# The evolution of abdominal microbiomes in fungus‐growing ants

**DOI:** 10.1111/mec.14931

**Published:** 2018-12-10

**Authors:** Panagiotis Sapountzis, David R. Nash, Morten Schiøtt, Jacobus J. Boomsma

**Affiliations:** ^1^ Centre for Social Evolution, Department of Biology University of Copenhagen 2100 Copenhagen Denmark

**Keywords:** 16S‐MiSeq sequencing, Actinobacteria, Attini, FISH confocal microscopy, microbiota, Mollicutes, α‐Proteobacteria

## Abstract

The attine ants are a monophyletic lineage that switched to fungus farming ca. 55–60 MYA. They have become a model for the study of complex symbioses after additional fungal and bacterial symbionts were discovered, but their abdominal endosymbiotic bacteria remain largely unknown. Here, we present a comparative microbiome analysis of endosymbiotic bacteria spanning the entire phylogenetic tree. We show that, across 17 representative sympatric species from eight genera sampled in Panama, abdominal microbiomes are dominated by Mollicutes, α‐ and γ‐Proteobacteria, and Actinobacteria. Bacterial abundances increase from basal to crown branches in the phylogeny reflecting a shift towards putative specialized and abundant abdominal microbiota after the ants domesticated gongylidia‐bearing cultivars, but before the origin of industrial‐scale farming based on leaf‐cutting herbivory. This transition coincided with the ancestral single colonization event of Central/North America ca. 20 MYA, documented in a recent phylogenomic study showing that almost the entire crown group of the higher attine ants, including the leaf‐cutting ants, evolved there and not in South America. Several bacterial species are located in gut tissues or abdominal organs of the evolutionarily derived, but not the basal attine ants. The composition of abdominal microbiomes appears to be affected by the presence/absence of defensive antibiotic‐producing actinobacterial biofilms on the worker ants' cuticle, but the significance of this association remains unclear. The patterns of diversity, abundance and sensitivity of the abdominal microbiomes that we obtained explore novel territory in the comparative analysis of attine fungus farming symbioses and raise new questions for further in‐depth research.

## Abstract 2

see also the Perspective by Pringle

## 1 INTRODUCTION

Several insect lineages have evolved farming symbioses with fungi. The oldest farmers are the platyponine ambrosia beetles (Smith, Kent, Boomsma, & Stow, 2018), which domesticated fungi ca. 90 MYA and later appear to have passed cultivars on to other clades of weevils (Vanderpool, Bracewell, & McCutcheon, 2018). The attine ants and macrotermitine termites began to rear fungi considerably later, but have surpassed the ambrosia beetles by evolving large‐scale farming comparable to culturally evolved human agriculture at an industrial scale (Aanen et al., 2002; Mueller & Gerardo, 2002; Nygaard et al., 2016; Schultz & Brady, 2008). Feeding up to millions within each colony, the ecological footprints of the fungus‐growing ants and termites in, respectively, new and old world (sub)tropical ecosystems are substantial (Mueller & Gerardo, 2002; Wilson, 1971). Many details of these farming symbioses differ, however. Ants and termites are very different insects, they rear unrelated basidiomycete cultivar lineages, their founding colonies acquire crop symbionts in fundamentally different ways, and their fungus gardens are very differently structured (Mueller, Gerardo, Aanen, Six, & Schultz, 2005). Convergent evolution transformed feeding and foraging habits, and both systems independently evolved ways to upscale provisioning of fungus gardens with vegetable matter (Aanen & Boomsma, 2005; Schultz, Mueller, Currie, & Rehner, 2005). In doing so, the fungus‐growing termites continued ancestral ingestion of dead plant material and ended up creating substrates for their fungus gardens from their primary faeces (Poulsen, 2015). However, the ancestors of the fungus‐growing ants were at least partly predacious and thus had to convert to foraging for leaf‐litter fragments and subsequently functional herbivory when the leaf‐cutting ants arose (Branstetter et al., 2017; Schultz & Brady, 2008).

Recent research has increasingly focused on characterizing the symbiotic, usually gut‐associated microbiomes of the fungus‐growing ants and termites. The litter‐feeding workers and minor soldiers of the Macrotermitinae have diverse bacterial gut microbiomes with decomposition functions complementary to those offered by the fungal cultivar, but royal pairs and soldiers have very low diversity gut microbiomes consistent with an exclusively fungal diet (Otani et al., 2014; Poulsen et al., 2014). Workers of the evolutionarily derived *Acromyrmex* leaf‐cutting ants appear to have microbiomes in their guts and associated abdominal organs of comparable simplicity to those of fungus‐growing termite royal pairs, consistent with all colony members having a specialized fungal diet (Sapountzis et al., 2015). These similarities suggest that food specialization reduces microbiome diversity, consistent with other ants and bees with specialized diets also having low diversity microbiomes (Anderson et al., 2012; Koch & Schmid‐Hempel, 2011; Martinson et al., 2011). As such, endosymbiotic social insect microbiomes appear to be more distinct than those of vertebrates, where even specialized food is still related to very diverse microbiomes as, for example, in the Giant Panda (Xue et al., 2015), and in a study of fish which suggested a negative relationship between diet diversity and microbiome complexity (Bolnick et al., 2014). This overall impression is reinforced by two meta‐studies in insects, which showed that hosts with more specialized diets tend to have less diverse gut microbiomes (Colman, Toolson, & Takacs‐Vesbach, 2012; Yun et al., 2014).

The last two decades have seen a significant general research effort to elucidate the intricacies of the fungus‐growing ant symbiosis. This work has documented that ant fungus farming evolved ca. 55–60 MYA and that the symbiosis underwent two subsequent transitions: one ca. 25–30 MYA, starting with the permanent domestication of a single fungal lineage that produced specialized hyphal tips (gongylidia) to feed the ants, and another ca. 15–20 MYA when the leaf‐cutting ants arose as active herbivores and evolved industrial‐scale farming (Currie, Poulsen, Mendenhall, Boomsma, & Billen, 2006; Kooij, Aanen, Schiøtt, & Boomsma, 2015; Mueller et al., 2005; Mueller, Rehner, & Schultz, 1998; Nygaard et al., 2016; Schultz & Brady, 2008; Shik et al., 2014; Villesen, Murakami, Schultz, & Boomsma, 2002). It was also discovered that ant fungus farms have suffered from a single specialized genus of *Escovopsis* mycopathogens throughout their evolutionary history (Currie, 2001; Currie et al., 2006), which arose shortly after the attine ants started to maintain their crop fungi in dense garden aggregations (de Man et al., 2016; Gerardo, Jacobs, Currie, & Mueller, 2006; Yek, Poulsen, & Boomsma, 2012). This specific disease pressure is relevant because the attine ants evolved cuticular cultures of antibiotic‐producing Actinobacteria to suppress *Escovopsis* and possibly other pathogens (Barke et al., 2010; Currie et al., 2006; Currie, Scott, Summerbell, & Malloch, 1999; Haeder, Wirth, Herz, & Spiteller, 2009; Mattoso, Moreira, & Samuels, 2012; Seipke et al., 2011). Cuticular actinobacterial biofilms have been maintained by many, but not all attine genera, so that attine ants are exceptional in often having both an external (cuticular) microbiome and an internal abdominal microbiome (Sapountzis et al., 2015). Considering that the cuticular actinomycetes produce a range of antibiotics and antifungals (Barke et al., 2010; Holmes et al., 2016; Oh, Poulsen, Currie, & Clardy, 2009; Seipke et al., 2011; Seipke, Grüschow, Goss, & Hutchings, 2012) that are applied to fungus gardens that the ants ingest, it is conceivable that cuticular Actinobacteria may affect the composition and diversity of microbiomes in the guts and associated organs, but this hypothesis has remained untested.

Here, we present the first large‐scale comparative analysis of abdominal microbiomes of attine fungus‐growing ants, covering 16 Panamanian species, including data from a previous study focusing on three *Acromyrmex* species (Sapountzis et al., 2015). Our study encompasses eight of the 17 currently recognized attine ant genera (Sosa‐Calvo et al., 2013; Sosa‐Calvo, Schultz, Ješovnik, Dahan, & Rabeling, 2018) and spans the two stepwise domestication transitions in fungus farming, the first one from small‐scale subsistence farming to specialized crop domestication and the final one that allowed industrial‐scale herbivorous farming to emerge. We define abdominal microbiomes as the bacterial communities associated with the intestinal system consisting of both the digestive tract and the associated endocrine and excretory organs such as the fat bodies and Malpighian tubules. Strictly speaking, the abdomen of myrmicine ants (the subfamily to which the attine ants belong) contains only the terminal segments because the first abdominal segment is merged with the thorax and the second and third segments are constricted into the narrow petiole and postpetiole. We will nonetheless use the abdomen connotation here to mostly avoid the little‐known ant‐specific term “gaster.” We characterize the abdominal bacterial communities and examine their abundance, variability and stability across species, samples and abdominal organs to examine whether and to what extent: (a) Evolutionarily derived forms of fungus farming led to changes in the overall abundance of endosymbiotic bacteria and the degree of dominance of specific symbionts, and (b) the composition and temporal stability of abdominal microbiomes is affected by the presence/absence of visible actinobacterial biofilms on the cuticle of worker ants.

A recent phylogenetic study (Branstetter et al., 2017) based on a powerful set of 1510 ultraconserved genomic elements has added considerable detail to our understanding of the biogeographic vicariance events that accompanied the transitions to more advanced forms of attine ant fungus farming. Ancestral state reconstructions showed that the evolution and domestication of gongylidia‐bearing cultivars happened in the dry habitats of South America away from the rainforests where fungus farming evolved, which helps to explain how the new cultivar lineage became reproductively isolated and started to co‐evolve with the farming ants. The study also revealed that most of the evolutionarily derived generic lineages of the higher attines including the *Acromyrmex* and *Atta* leaf‐cutting ants, evolved in Central/North America from a single colonizing lineage that reached the northern subcontinent ca. 20 MYA, that is, well before the Isthmus of Panama closed. Our present study was essentially completed before Branstetter et al. (2017) was published, but we explicitly analyse, interpret and discuss our results in the light of this new biogeographical frame of reference.

## 2 METHODS

### 2.1 Collections, dissections, DNA extractions and 16S‐MiSeq sequencing

We used a total of 63 colonies from 14 fungus‐growing ant species (this number increased to 76 colonies and 17 species after including data from 13 colonies of three *Acromyrmex* spp. previously published by Sapountzis et al. (2015), see also below). For the rare *Trachymyrmex *sp. 3 and *Cyphomyrmex rimosus*, we only found two and three colonies, respectively, and we only managed to obtain a single colony each of *Apterostigma cf. collare* and *Acromyrmex volcanus*. For all other ant species, we used four to six colonies, including two or three field colonies (for abdominal dissections or obtaining homogenates of entire abdomens) within a week of collection. These colonies were resampled after three months rearing in the laboratory, and similar samples were obtained from one to three colonies of the same species that had been collected on previous field trips and been maintained in the laboratory for more than two years (Supporting information Table S1). Ant species maintained in the laboratory were either fed with bramble leaves, dry rice and pieces of fruit (leaf‐cutting ants), polenta (lower attines), or a combination of polenta and bramble leaves (*Trachymyrmex* and *Sericomyrmex*). To restrict variation in environmentally acquired bacteria, and to allow unbiased comparison between long‐term laboratory colonies and a previous study (Sapountzis et al., 2015), we only used colonies collected in and around Gamboa, Republic of Panama, that were transferred to the same laboratory rearing rooms in Copenhagen and were maintained at ca. 25˚C and 70% RH.

Our analysis focused on the bacterial communities associated with the digestive tract, the endocrine organs and the excretory systems of the attine ants. To this end, we used dissected tissues (midguts, hindguts, Malpighian tubules and fat bodies) for most ant species and surface‐cleaned entire abdomen (gaster) samples for species with workers that were too small to dissect. The latter samples also included cuticle material, some additional glandular tissue, some nervous tissue and the (normally inactive) ovaries, which only marginally affected our estimates of bacterial abundance (see below). Ant workers were anesthetized on ice, surface‐cleaned by submerging them in absolute ethanol for 60 s and then rinsed with sterilized distilled water. We are aware that this external cleaning procedure will not have achieved 100% surface sterilization, but decided not to use more aggressive methods that could also degrade bacteria associated with internal tissues. This procedure also appeared reasonable in the light of a recent study (Birer, Tysklind, Zinger, & Duplais, 2017) showing that workers of *Atta* leaf‐cutting ants have very few cuticular bacteria, a result that we confirmed for several other attine species. In this context, it is important to note that cuticular biofilms of Actinobacteria on mature workers occur on the propleural (chest) plates and sometimes on surrounding thorax or leg body parts (Currie et al., 2006), so they will not have had direct effects on the abdominal microbiomes that we obtained. Dissections (only feasible for species with workers of at least ca. 5 mm body length) were done in sterile phosphate‐buffered saline (PBS), pH 7.4 under a stereo microscope, after which tissues were stored at −80˚C for later DNA extraction.

To make sure that our use of two sampling methods would not significantly affect our results, we also obtained a separate set of entire abdomen (gaster) samples from long‐term laboratory colonies of the larger‐bodied species. This allowed us to directly compare qPCR analyses of bacterial titres between entire ants and dissected abdominal samples for the species where dissections were feasible, that is, all species of *Acromyrmex*, *Atta*, *Sericomyrmex* and *Trachymyrmex* and *Apterostigma dentigerum*. To obtain these directly comparable samples, we collected abdomens from five workers from three colonies for the same species and partly the same colonies (Supporting information Table S1) and used the same qPCR methods to compare overall bacterial abundances (titres). For the remaining small‐bodied species (*Cyphomyrmex *spp., *Mycocepurus smithii* and *Myrmicocrypta ednaella*), we used the already collected surface‐cleaned worker abdomens as described above (Supporting information Table S1). We will henceforth use “dissected abdomens” when referring to dissected tissues and “entire abdomens” when referring to entire surface‐cleaned body parts also known as ant gasters. This two‐stage extra procedure allowed us to show that bacterial titres for entire abdomen samples were only slightly higher than for dissected samples so that biasing effects emerging from dissection constraints in small‐bodied species have remained limited. We will further discuss these issues when presenting our results.

DNA was extracted from the frozen samples using the Qiagen Blood and Tissue kit following the manufacturer's instructions, and all samples were re‐eluted in 150 µl AE buffer (Qiagen). We always included 1–2 blank “water” DNA extraction samples and quantified sample concentration using an ND‐1000 spectrophotometer (NanoDrop Technologies, Wilmington, DE, USA) before performing PCR reactions using 16S universal primers (Kozich, Westcott, Baxter, Highlander, & Schloss, 2013) to identify the optimal templates for library preparation and to confirm there was no detectable 16S amplification in the blank DNA extractions. Extracted DNA was sent to the Microbial Systems Laboratory at the University of Michigan for library preparation (multiplexed samples) and 16S‐MiSeq paired‐end sequencing, using the Kozich et al. (2013) protocol to amplify the V4 region including three blank “water” samples in the same MiSeq run.

The PCR mix contained 2.0 µl 10× AccuPrime™ PCR Buffer II (15 mM MgCl_2_), 0.15 µl AccuPrime™ Taq DNA Polymerase (2 units/µl, Life Technologies), 1.0 µl of each primer (10 µM), 1.0 µl diluted template and autoclaved MilliQ water (the same water used for the blank controls) to a total of 20 µl. PCR incubation conditions included an initial activation of the hotstart polymerase at 95°C for 2 min, followed by 30 cycles of 95°C for 20 s, 55°C for 15 s, 72°C for 5 min, and final extension at 72°C for 5 min. For samples that did not amplify with this protocol (for a detailed list see Supporting information Table S1), we used an initial activation of the hotstart polymerase at 95°C for 2 min, followed by 20 cycles of 95°C for 20 s, 60°C for 15 s with a decrease of 0.3°C per cycle, and 72°C for 5 min, then 20 cycles of 95°C for 20 s, 55°C for 15 s and 72°C for 5 min, and a final extension at 72°C for 10 min (touchdown PCR). PCR products were purified using a magnetic bead capture kit (Ampure; Agencourt) and quantified using a fluorometric kit (QuantIT PicoGreen; Invitrogen). The purified amplicons were then pooled in equimolar concentrations and sequenced.

### 2.2 Raw data analyses and OTU assignments

The MiSeq data were analysed using mothur v1.33.3 (Schloss et al., 2009) after several rounds of filtering as described in the standard operating procedure (SOP; page accessed July 2014) with a few modifications (Kozich et al., 2013). We also included data that were part of a previous publication (Sapountzis et al., 2015), which had been sequenced in the same MiSeq run. Contigs were created using the *make.contigs* command and were subsequently trimmed to remove sequences exceeding 275 bp or having ambiguous base pairs. We then identified unique sequences and aligned them against a customized database originating from a silva nonredundant database (version 111, accessed July 2014), which had only the V4 region of the 16S rRNA gene. Sequences that did not cover positions 13,862–23,444 of the full‐length silva alignment (V4 hypervariable region) or had a homopolymer length above 8 were removed. Overhangs on the sequences were also removed using the *filter.seqs* command and all sequences were screened again in order to retain only the unique ones. Chimeric OTUs were removed before sequences were assigned to taxonomic groups using the Bayesian classifier implemented in mothur with a confidence threshold of 80% against the same silva database used for the alignment, after which any reads belonging to Archaea, Eukaryota, chloroplasts or mitochondria were removed.

OTUs were identified at 97% similarity and all singletons were retained for further analysis, since there is no objective way to distinguish between rare but important OTUs representing true diversity and contaminants or sequencing errors. Rarefaction curves were visualized (Supporting information Figure S1) using the *R* package inext (Hsieh, Ma, & Chao, 2016), and rarefaction tables were constructed with mothur using pseudoreplicate OTU data sets containing between 1 and 122,998 sequences, with 1,000 iterations per pseudoreplicate. The final OTU table was rarefied at 8,900 reads, which reduced the number of OTUs from 3,334 to 2,303 and was used for any downstream analyses that did not exclusively consider the most abundant OTUs having prevalences (relative abundance) of >10% in at least two samples across the entire data set (Supporting information Figure S2). Following Hu et al. (2017), we identified the OTUs present in the blank “water” samples that were sequenced along with the real samples, and removed OTUs as contaminants if their relative abundance produced an average blank/sample ratio ≥0.2. We also performed an extra set of analyses focusing on the unique sequences in our data set, using a variety of filtering procedures to validate that the relatively high bacterial diversity that we obtained for many of the lower attine abdominal samples was not an artefact associated with having low amounts of bacterial DNA (available at https://doi.org/10.5061/dryad.tj30d), a known possible complication of MiSeq sequencing studies (Salter et al., 2014). This further reduced the number of OTUs to 3,103 (unrarefied) and 2,099 (rarefied).

For the maximum‐likelihood phylogenetic tree, the 16S sequences were extracted from the MiSeq data using the get.oturep command in mothur. Sequences were aligned using muscle v3.8.31, the alignments were checked with jmodeltest v2.1.7, and the phylogenies were built with raxml v8.2.11 using the “rapid bootstrap and best‐scoring ML” algorithm with 1,000 bootstraps after further modification in figtree v1.4.2.

### 2.3 Comparisons across phylogenetic farming transitions, sampling categories and presence/absence of cuticular Actinobacteria

The richness, diversity and composition of the specific abdominal bacterial communities were compared based on phylogenetic position of the ants and two additional partitions. Phylogenetic signals in the data set were examined based on a modified version of the tree presented by Schultz and Brady (2008), incorporating also data from Mehdiabadi, Mueller, Brady, Himler, and Schultz (2012), Ješovnik, González, and Schultz (2016) and Nygaard et al. (2016), and assuming that *S. amabilis* is a close relative of *Sericomyrmex cf. parvulus*. Branch lengths were based on the divergence times estimated by Schultz and Brady (2008), and between‐colony variation within each species was entered as a polytomy at 1 MYA. This within‐species divergence time represents approximately half the observed divergence time of the most closely related sister species in the tree and was considered to be a reasonable compromise between likely overestimating the divergence time between colonies and likely underestimating random variation between colonies not due to common ancestry. The tree was then used to obtain phylogenetic signals in overall bacterial abundances (qPCR data, see below) and alpha diversity (species richness, diversity and inequality) and to produce a matrix of pairwise divergence times between ant species to examine beta diversity.

Additionally, we partitioned samples based on the environmental conditions that colonies experienced at the time of sampling, that is, whether workers were collected in the field, from colonies ca. 3 months after establishment in the laboratory (short‐term laboratory) or from colonies that had been kept in the laboratory for >2 years (long‐term laboratory). We refer to this partitioning as sampling environment. The final partitioning that we used was between those species whose workers have clearly visible cuticular Actinobacteria on at least some body parts, and those that do not, since these Actinobacteria produce antibiotics that can potentially influence the composition and diversity of endosymbiotic microbiomes. The effects of these two partitions on measures of alpha and beta diversities were always examined while statistically removing any phylogenetic signal present.

### 2.4 Diversity analyses

Many different measures of alpha diversity for samples of communities have been proposed. The simplest of these is richness—the number of species (or in our case, OTUs) observed in a sample, *S*_obs_—but this does not take into account differences in prevalence of OTUs within samples. Richness can be decomposed into independent “true diversity” and “inequality” components (Jost, 2010), with a common family of measures of “true” diversity putting increasing emphasis on common rather than rare OTUs based on what is referred to as Hill numbers (Hill, 1973). A Hill number of 0 gives *S*_obs_ *,* a Hill number of 1 is the exponential of Shannon entropy (*e^H^*), and a Hill number of 2 is the inverse Simpson diversity index, etc. We therefore calculated both *S*_obs_ and *e^H^* for each sample as measures of diversity in mothur (Schloss et al., 2009), together with their related measures of inequality (*S*_obs_ */e^H^*; equivalent to the exponential of Theil entropy (Theil, 1967), *e^T^*), and compared these across samples. To avoid confusion between the two entropy terms, we will refer to Shannon entropy as Shannon diversity and to Theil entropy as Theil inequality in the remainder of this study. In addition, we also examined phylogenetic diversity (Faith, 1992) of bacterial OTUs across samples, to weight OTU richness by phylogenetic distance between bacterial OTUs. For analyses, each index was log‐transformed to normalize its distribution and reduce heteroscedasticity (i.e., the indices used were *log_e_S*_obs_, *H*, *T *and *log_e_PD*). The *phylosig* command within the *R* package phytools (Revell, 2012) was used to test whether there was any significant phylogenetic signal in these indices. This procedure computes a measure of phylogenetic signal, K (Blomberg, Garland, & Ives, 2003), and tests the probability that it could arise by chance with the selected data set using 100,000 randomizations across the phylogeny. Changes in the rate of evolutionary change in each index across the phylogeny were modelled using a Bayesian Markov chain Monte Carlo (MCMC) approach, using a Brownian model of character evolution, as implemented in the *evol.rate.mcmc* command in phytools, which identifies the phylogenetic location of shifts (splits) in the evolutionary rate through time (Revell, Mahler, Peres‐Neto, & Redelings, 2012). Default values were used for this command, including uniform priors, but with the number of MCMC generations increased to 100,000 to ensure model convergence. The split representing the position of the largest transition in evolutionary rate in the tree was then identified using the *MinSplit* command on the model output. Using the same phylogeny and diversity indices, we also identified the most pronounced additional splits using the surface package in R, which identifies all significant transitions across the phylogeny based on an Ornstein–Uhlenbeck model of character evolution and stepwise Akaike information criteria (Ingram & Mahler, 2013). The explanatory power of the intermediate split that we obtained empirically (referred to as “A‐transition”), relative to the “traditional” transitions from lower‐to‐higher and higher‐to‐leaf‐cutting attines, was evaluated by comparing the Akaike weights of linear mixed models in which the maximally supported split and the two traditional splits were included as explanatory variables, with ant species nested as a random factor within the resultant groups. We then used the evidence ratios of the Akaike weights of each combination of two models as estimates of how many times better the maximally supported split performed relative to the alternative traditional splits.

Differences in bacterial richness, diversity and inequality between sampling environments and the presence/absence of cuticular Actinobacteria were examined using a Bayesian (MCMC based) generalized linear mixed model as implemented in the *R* package mcmcglmm (Hadfield, 2010). This approach allows a phylogenetic tree to be included in the model as a random effect, so that the fixed effects can be examined independent of any phylogenetic signal (Hadfield & Nakagawa, 2010). For each index, five models were constructed, including all possible combinations of sampling environment (E) and the presence or absence of Actinobacteria (A) as main effects, and their possible interactions (i.e., E + A + E × A; E + A; E; A; and only random effects), after which the best model based on the AICc values was selected with the *R* package mumin (Bartón, 2017). mcmcglmm estimates a significance level for the comparison between a reference category and each other category within categorical main effects, so for sampling environment the short‐term laboratory category was chosen as the reference level, as it allowed direct comparison of both changes associated with transfer from the field to the laboratory and changes associated with long‐term laboratory culture.

To examine the differential representation of particular OTUs between different types of partitioning of samples, we used the deseq2 program in R (Love, Huber, & Anders, 2014). We also examined similarities and differences in OTU community (beta diversity) between pairs of samples to validate whether: (a) Any significant transition in overall bacterial abundance that we uncovered did in fact concern largely the same OTU composition or involved a significant discontinuous shift in OTU identities, and (b) whether transfer of colonies to the laboratory might have caused general shifts of this kind in overall OTU compositions because there might exist typical laboratory‐invasive strains that could become more generally abundant while they were absent or rare in the field.

We used three different measures to quantify pairwise bacterial community dissimilarity between samples: Bray–Curtis dissimilarity and weighted and unweighted UniFrac distances (Lozupone & Knight, 2005). To visualize differences in beta diversity (changes in community composition), and to reduce the number of variables for subsequent discriminant analyses, the matrices of pairwise dissimilarities between samples were ordinated using two‐dimensional nonmetric multidimensional scaling (NMDS) with the *metaMDS* command, and plotted using the *ordiplot* command in the *R* package *vegan*. The NMDS stress, which shows how well the samples are represented by two axes, was calculated using the “stressplot” command and is given in each ordination plot. All but one of these stress values were ≤0.2 suggesting good representation of the variation by 2 axes and the remaining one was only slightly higher. We carried out linear discriminant analyses based on the NMDS scores to evaluate how well ant species could be separated based on their bacterial OTU communities retrieved from sampling replicate colonies, within the two partitions of phylogeny and presence/absence of cuticular Actinobacteria that we used. This produced estimates of the percentage of samples assigned to the correct ant species, which were further evaluated by the associated Entropy *R^2^* value, a measure of discriminatory power with higher values indicating better discrimination. To examine differences in the average similarity of abdominal bacterial community samples across partitions (basal vs. evolutionarily derived attines; cuticular Actinobacteria present or absent; field vs. short‐term laboratory vs. long‐term laboratory collections), we used PERMANOVA (permutational multivariate analysis of variance) based on the pairwise dissimilarity matrices with the *adonis* command in *vegan*, using 9,999 permutations. Finally, we explicitly assessed differences in the variability in bacterial communities between ants with or without cuticular Actinobacteria and between the best‐supported phylogenetic split (“A‐transition”) identified in our analysis, because these differences remain implicit in discriminant analysis and are potentially relevant if phylogenetic transitions or shifts in the presence/absence of cuticular Actinobacteria would imply that abdominal bacterial communities become specialized on not only different but also fewer OTUs. For this analysis, we used the HOMOVA command in mothur based on the pairwise dissimilarity matrices, comparing homogeneity of variance between pairs of partitions of the complete set of samples, and applying Bonferroni‐adjusted alpha‐values because of multiple tests across the same data matrix.

### 2.5 Fluorescent in situ hybridization (FISH) microscopy

To start understanding what putative functions the most abundant bacterial endosymbionts may have, it is important to visualize them in the organs they are primarily associated with. We therefore used probes from a previous study (Sapountzis et al., 2015) and designed several more (Supporting information Table S2) so we could target most of the abundant OTUs (according to our double >10% presence criterion) and use FISH microscopy to identify their main locations along the intestinal system and its associated organs. Four of these abundant OTUs (*Rhizo19*, *Solir27*, *Rhizo30* and *Rhizo35*) were only detected in field samples and thus had to be excluded for FISH microscopy. We chose this specific FISH approach instead of using 16S EUB probes that target all bacteria, because it allowed us to use: (a) two specific types of probe (OTU‐ or class‐specific) and (b) DAPI staining as a positive control to detect all bacteria in the same sample. The procedure for designing probes was that OTU sequences were retrieved using the *get.oturep* command implemented in mothur and aligned using the Geneious Custom Assembly algorithm, after which probes could be designed using geneious R7 v7.0.6 (Kearse et al., 2012). OTU representative sequences were then compared with each other and with the newly designed probes using the blast algorithm (Altschul, Gish, Miller, Myers, & Lipman, 1990) against the nonredundant silva database using the web interface, and against a local database that was created using the *makeblastdb* command with a fasta file containing all the representative OTU sequences identified in this study. Probes were further tested with an online tool (OligoAnalyzer 3.1, Integrated Data Technologies) to check for hairpins and self‐dimers (Supporting information Table S2).

Six‐to‐ten ant workers from several short‐term and long‐term laboratory colonies belonging to 10 of the 17 attine species used for 16S‐MiSeq analysis (*At. colombica*, *At. cephalotes*, *T.cornetzi*, *T. zeteki*, *S. amabilis*, *C. costatus*, *C. longiscapus*, *Myc. smithii*, *Myr. ednaella *and *Ap. dentigerum*) were dissected in PBS and their gut, endocrine and excretory tissues (midgut, fat bodies, Malpighian tubules, ileum and rectum) were placed in 4% paraformaldehyde for at least 24 hr (see Supporting information Table S1 for details of colonies used). The localizations of the OTUs of interest in *Ac. echinatior* and *Ac. octospinosus* have been characterized in a previous study (Sapountzis et al., 2015) and are therefore not presented here. Half of the worker tissue samples were used for hybridization with OTU‐specific or class‐specific probes, and the other half for hybridization with antisense probes (reversed sequences) serving as negative controls. A single microscopy sample always consisted of tissues from three to five ant workers from the same colony. For the permeabilization, deproteinization and hybridization (using 0.75 µg/µl of our 16S‐specific labelled probes; Supporting information Table S2), we followed a previously established protocol (Sapountzis et al., 2015). All slides were observed and photographed using a Zeiss LSM 710 laser‐scanning confocal microscope equipped with ZEN 2009 software and a Leica TCS SP2 microscope. Images were processed with Adobe Photoshop CS5 for publication to merge the three channels and add scale bars. Merged RGB images were subsequently processed with ImageJ to evaluate the morphology of the suspected positive bacterial signals (granulometric filtering) and with a customized JavaScript to create frames showing special regions of interest (ROIs).

Scoring microscope image signals against background autofluorescence is a semiquantitative manual procedure that precludes formal statistical comparisons. Following Sapountzis et al. (2015), we used very strict criteria for accepting positive signals as genuine. We only considered signals that colocalized with DAPI signals of bacterial size (ca. 2 µm) and which were visible in only one of the probe channels. This is a reliable signal quality check because we never stained the same bacterial OTUs with different fluorochromes, so signals appearing in both probe channels would imply either autofluorescence or unspecific staining. We confirmed that our experimental protocol did not allow for unspecific binding of probes by using same‐tissue images stained with matching antisense probes, which showed no specific staining of the 16S antiprobes but only DAPI signals. The same process was used in an earlier study, which confirmed high probe specificities with electron microscopy (Sapountzis et al., 2015).

### 2.6 Validating FISH observations on overall bacterial abundances with quantitative PCR

To quantify the relative bacterial 16S abundance levels for each of the ant species, qPCR was carried out on samples of pooled entire abdomens (gasters) from each of the species used for FISH (Supporting information Table S1). The primers used for the MiSeq library construction (515F/806R; obtained from Kozich et al. (2013)) were also used for the qPCR reactions, which were performed as described by Sapountzis et al. (2015). All qPCR reactions were replicated twice, and the Ct (cycle threshold) mean across replications was used as a measure of amplicon abundance relative to two negative controls of no added template. Cell count data were normalized relative to the ant nuclear gene *elongation factor 1 alpha* (*EF‐1α*) (Andersen, Boye, Nash, & Boomsma, 2012), and standard curves with PCR products in tenfold dilution series of known concentration were used for both 16S and *EF‐1a*. The Ct values of the standards were then used to calculate PCR efficiency using the software rest v3.04 (Pfaffl, Horgan, & Dempfle, 2002), after which data were expressed as fold changes using a previously described formula for relative quantification (Pfaffl, 2001), with the mean of the three *Ap. dentigerum* samples (the most basal branch in the phylogeny) as reference (Adent_f1, Adent_3m1 and Adent_3m2: supporting information Table S1). The phylogenetic signal in overall bacterial abundances and how phylogenetically independent abundances differed between laboratory and field‐collected samples, and between species with and without cuticular Actinobacteria, were analysed using the same procedures as for alpha diversity.

## 3 RESULTS

### 3.1 Identification of abdominal bacteria across the phylogeny of fungus‐growing ants

**Figure 1:**
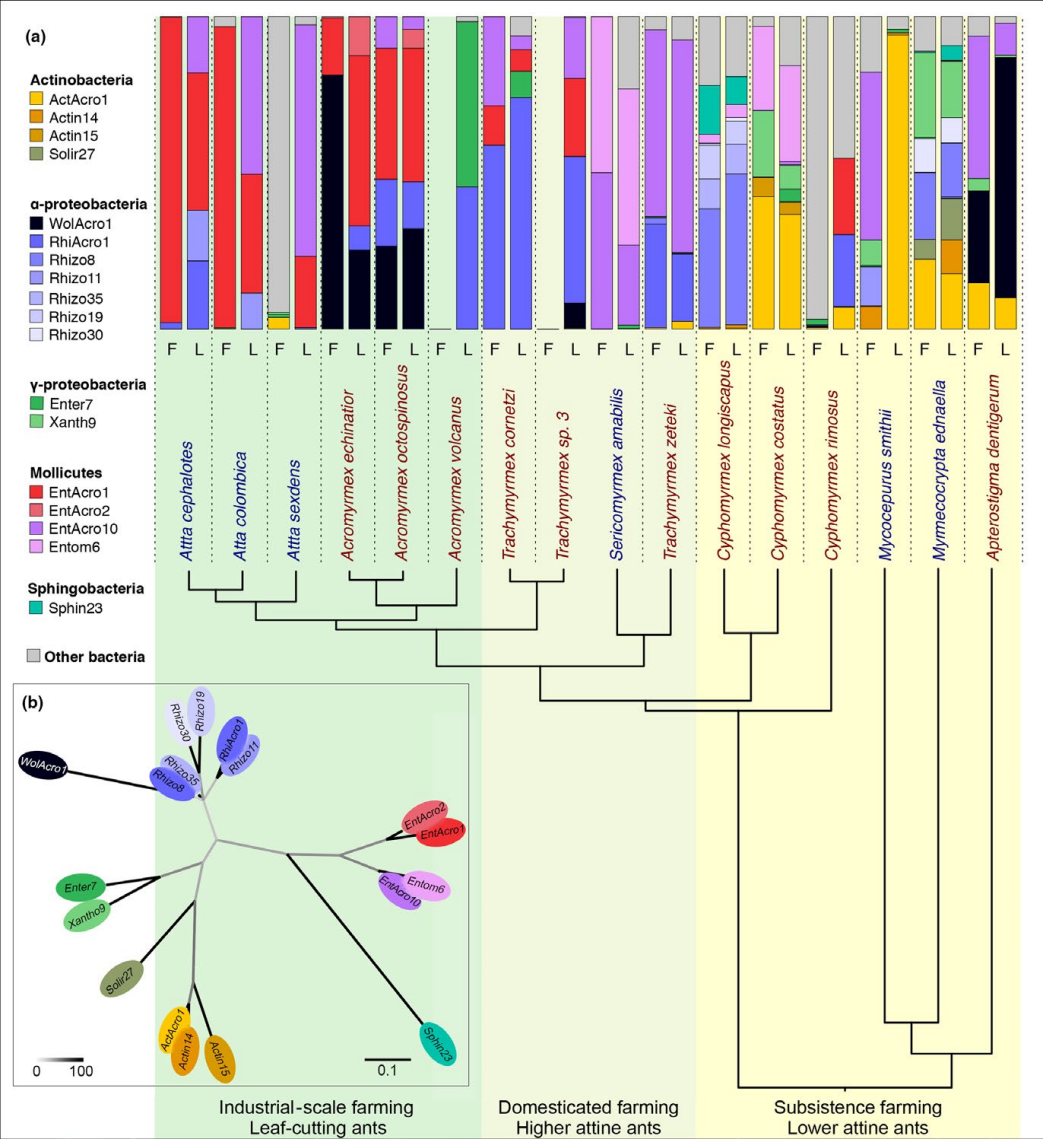
Relative abundances (prevalences) of the 18 abundant abdominal bacterial OTUs across 16 Panamanian fungus‐growing ants based on 84 samples for which libraries were generated after amplification with standard PCR. (a) The host phylogenetic tree has been modified from (Branstetter et al., 2017; Schultz & Brady, 2008)—see text and Supporting information Figure S4. Known farming transitions are highlighted by background colour shifts from yellow to light green to darker green. Vertical bars only include the 18 OTUs that reached ≥10% in at least two ant samples (see colour legend) and a single pooled prevalence of all other OTUs (grey). The left bar for each species refers to field‐collected samples (F) and the right bar to ants sampled after colonies were maintained in the laboratory (L), pooling short‐term and long‐term laboratory colonies. Relative abundances were calculated using all 2099 OTUs after rarefaction and averaged across all field or laboratory samples for each species. Species names are coloured according to whether worker ants have visible cuticular Actinobacteria (red) or not (blue). The phylogenetic relationships of the 18 abundant OTUs can be seen in Supporting information Figure S7. (b) A maximum‐likelihood nonrooted phylogeny showing the relationships of the 18 abundant OTUs identified in our study (following the >10% prevalence criterion in at least two of the 84 samples amplified with standard PCR) with ellipses in the same colours as the bars in the A‐panel. The scale bar at the bottom illustrates how branch lengths reflect substitution rates, and the heatmap bar represents degree of bootstrap support

Our analyses of 107 samples from 76 Panamanian colonies (including 19 *Acromyrmex* samples from 13 colonies from Sapountzis et al., 2015) retrieved 3,334 bacterial OTUs (operational taxonomic units = Putative species), including singletons, at the 97% sequence identity cut‐off, across 48 bacterial classes. After rarefaction and filtering of “water” OTUs (removed when present in the negative controls; see Methods), this number was reduced to 2099 (Supporting information Figure S1 and Table S3). The most prevalent of these were Mollicutes (334,358 hits; >35% of all reads; 16 OTUs), α‐Proteobacteria (306,740 hits; >32%; 252 OTUs), Actinobacteria (134,031 hits, >14%, 240 OTUs) and γ‐Proteobacteria (80,281 hits, >8%; 143 OTUs) (Figure 1 and Supporting information Table S3). Rarefaction curves showed that sampling had been adequate except for a single field sample of *C. rimosus* (Supporting information Figure S1), which we nonetheless included in the analyses as the effect of undersampling was generally conservative (i.e., differences would have been higher if more OTUs had been sampled). To facilitate reading and interpretation, we have named the 98 most abundant OTUs (OTUs0001–0100 based on Mothur's relative abundance ranking, using five letters that refer to their bacterial order followed by a specific number that identifies their overall rank (Supporting information Figures S2 and S3; Table S3). This produced a list of 98 OTUs rather than 100, because rarefication at 8,900 reads eliminated two OTUs.

Before using these comparative data to evaluate diversity patterns, we examined potential confounding effects in our sample preparation methods that could have emanated from: (a) the initial analyses having used both standard PCR (84 samples) and touchdown PCR (23 samples) to generate the libraries, (b) pools of five ant workers (most species) and pools of 10–15 workers (some very small lower attine ant species) having to be compared, (c) the presence of potential laboratory‐borne contaminants and (d) the unavoidable lower biomass of some samples (Salter et al., 2014). Ordination and matching statistical tests showed that we could not reliably exclude that the number of individuals sampled and particularly the PCR method used might have affected our bacterial richness scores and alpha diversity estimates (Supporting information Results S1). These complications, together with the unknown likelihood that some contaminations might not have been filtered out because we did not run a high number of blank samples, imply that we present our alpha diversity estimates with some precaution, and that we based our remaining analyses on the 84 samples that were amplified with standard PCR. This meant that a single species (*Apt. collare*) for which we only had a single sample that had been amplified with touchdown PCR has not been considered further, so the number of species dropped from 17 to 16 in most analyses.

To ensure that our emphasis would focus on bacteria with the highest likelihood of having functional significance for ant metabolism and performance of the fungus farming symbioses as a whole, we identified the limited set of OTUs with prevalences of more than 10% in at least two samples—a criterion that emphasizes relative abundance while avoiding that single occurrences are decisive. Our initial analyses based on 107 samples identified 21 OTUs that met this criterion, which accounted for more than 83% of the total reads, a number that was reduced to 18 OTUs accounting for more than 90% of the total reads when we only considered the 84 samples amplified with standard PCR (Supporting information Figure S3 and Table S3) because touchdown PCR tends to generate more spurious sequences. Six of these 18 or 21 abundant OTUs have previously been identified in the guts and associated organs of three species of *Acromyrmex* leaf‐cutting ants from the same sampling site, and retained their names from that study (*ActAcro1,* *EntAcro1*, *EntAcro2, EntAcro10*, *RhiAcro1* and *WolAcro1*: Sapountzis et al., 2015).

Distribution patterns of OTUs across the phylogenetic tree are given in Figure 1 and Supporting information Figure S3, arranged according to the phylogenetic relationships between the 16 attine species. The four dominant bacterial classes were all represented in the lower attine ants, but the higher attine ants had generally endosymbiotic microbiomes dominated by fewer OTUs that belong more predominantly to the Entomoplasmatales (Mollicutes) and Rhizobiales (α‐Proteobacteria). In contrast, Actinobacteria were prevalent abdominal endosymbionts in many lower attines, but were essentially lacking in the higher attines and leaf‐cutting ants. The basal higher attine ant species *Trachymyrmex* (but not the *Sericomyrmex*) have a Rhizobiales symbiont (*RhiAcro1*) not found in the lower attines (with the exception of *C. rimosus*). Shifts in endosymbiotic abdominal OTUs continued in the leaf‐cutting ants, with the genus *Acromyrmex* having Entomoplasmataceae (*EntAcro1* & *EntAcro2*) rather than the typical Spiroplasmataceae (*Entom6* & *EntAcro10*) of *Trachymyrmex* and *Sericomyrmex*, and abundant *Wolbachia *(*WolAcro1*; α‐Proteobacteria) otherwise only found in the most basal attine *Apt. dentigerum*. Enterobacteriaceae (*Enter7*; class: γ‐Proteobacteria) also appear here in a single species (*Ac. volcanus*; the same OTU as in *T. cornetzi *and *C. costatus*). Finally, across the *Atta *species, we found the same Spiroplasmataceae (*EntAcro1* & *EntAcro2*; class Mollicutes) as in *Acromyrmex* but a different common Rhizobiales OTU (*Rhizo11*) because *RhiAcro1* was largely absent, with the exception of most colonies of *Ac. cephalotes*. *Wolbachia *was only found in traces across *Atta* species, except for a single *Atta sexdens* field colony where *Wolbachia* reached higher prevalence.

**Figure 2:**
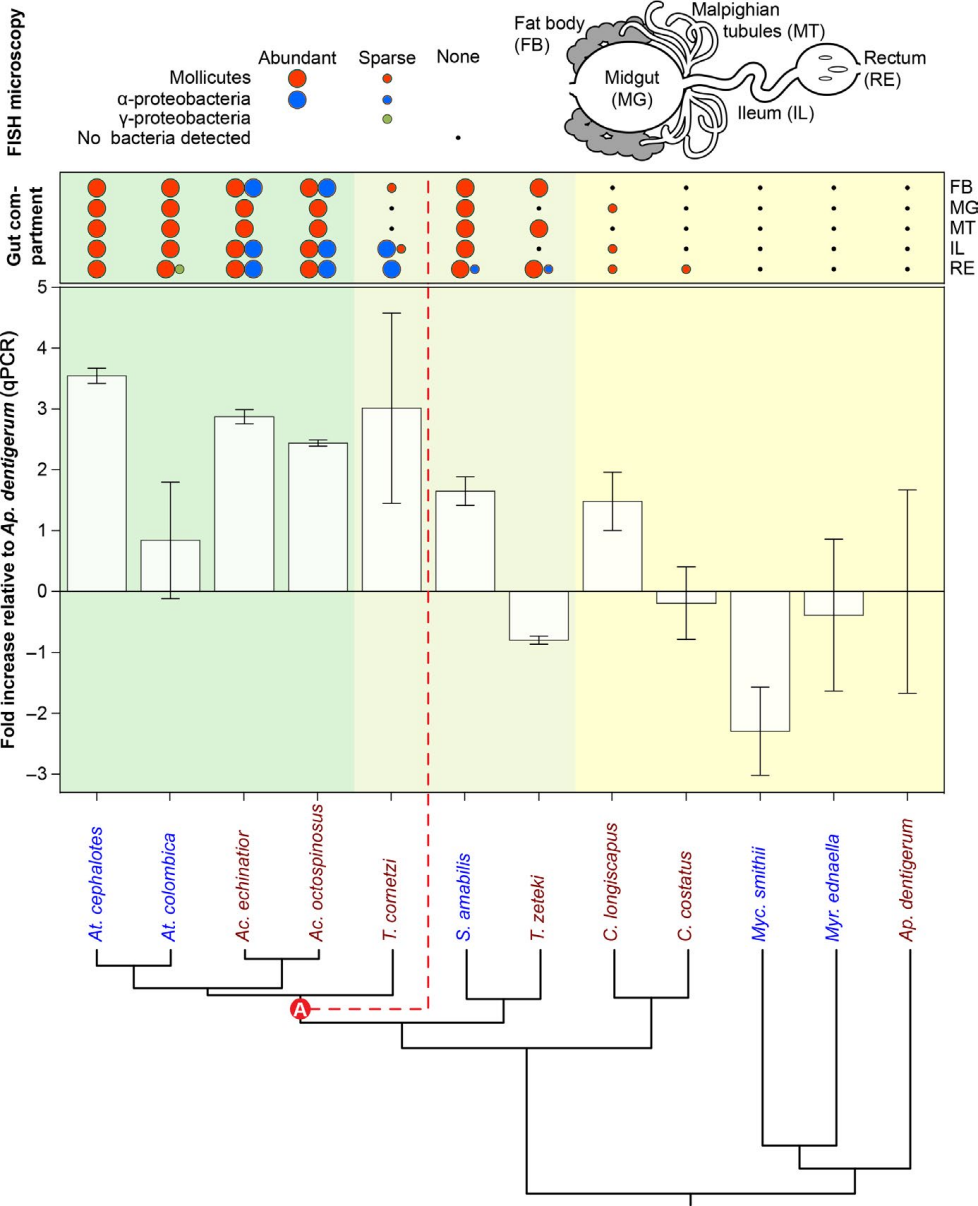
Summary of gut partitions where bacterial OTUs could be localized with fluorescence in situ hybridization (FISH) microscopy and overall bacterial abundances measured as 16S rRNA copies in homogenized abdomens of 12 attine ants using qPCR, ordered from crown to base of the attine phylogenetic tree. QPCR data are presented as the ratio of the total number of bacterial 16S rRNA gene copies relative to the number of ant *EF‐1a* gene copies per sample and plotted relative to the mean value for the phylogenetically basal *Ap. dentigerum *samples as calculated by the method of Pfaffl (2001). Results are shown as means ± *SE*, based on three colony‐level DNA samples for each ant species. The phylogeny is a 12‐branch extract of the tree shown in Figure 1. The steepest observed transition in overall (total 16S copies) abundance is marked as transition “A.” A similar analysis using qPCR data without normalization comparing entire and dissected abdominal samples produced essentially the same results (supporting information Results S3). Above each histogram bar, the presences or absences of 13 of the 18 abundant OTUs for which FISH probes were available are plotted in two qualitative abundance categories reflecting whether FISH signals were consistently detected or rarely present. The 16S‐FISH confocal microscopy was done separately for fat body cells, midgut, Malpighian tubules, ileum and rectum (see schematic diagram at the top right and Supporting information Results S2 for details)

Our analyses of alpha diversity across the 84 samples indicated that OTU richness (*log_e_S*_obs_), diversity (Shannon diversity *H*), inequality (Theil inequality *T*) and phylogenetic diversity (*log_e_PD*) of abdominal bacteria all showed a significant phylogenetic signal (*log_e_S*_obs_: *K* = 0.0847, *p* < 0.0001; *H*: *K* = 0.0515, *p* = 0.0022; *T*: *K* = 0.0588, *p* < 0.0001; *log_e_PD*: *K* = 0.0829, *p* < 0.0001) (Supporting information Figure S4 and Table S4). However, the branch in the phylogenetic tree at which the rate of change was highest differed between the different indices and types of analyses: The *minsplit* analysis identified the largest split within *S. amabilis* (Shannon diversity *H*), between the paleo‐attines (*Myrmicocripta*, *Mycocepurus*, *Apterostigma*) and the remaining (*Cyphomyrmex*, *Trachymyrmex*, *Sericomyrmex*) lower attines (Theil inequality *T*) and between *C. rimosus* and the other *Cyphomyrmex* spp. (*log_e_S*_obs,_ *log_e_PD*), while “surface” analysis suggested an important transition within *S. amabilis* (Shannon diversity *H*), between higher and lower attines (*log_e_S*_obs_) and within the *Trachymyrmex *grade (Theil inequality *T*, *log_e_PD*). We will refer to the latter as transition “A” (see Supporting information Figure S4 and qPCR results given below and in Figure 2). Although there is substantial variation across the phylogenetic branches, the overall statistics indicate that there is a trend towards lower alpha diversity when going from the base to the crown of the attine ant tree. However, we do not consider this to be a hard result because our validations indicated that our alpha diversity estimates were likely influenced by a number of confounding effects (Supporting information Results S1).

### 3.2 OTU localization by FISH and estimates of overall bacterial abundances using qPCR

FISH microscopy showed that abundant Mollicutes OTUs were widespread across many abdominal tissues in both *Acromyrmex *and *Atta* leaf‐cutting ants (Supporting information Results S2; Sapountzis et al., 2015), but that the abundant Rhizobiales OTU (*RhiAcro1*) in *Acromyrmex, *often co‐occurring with *Wolbachia* (Andersen et al., 2012; Sapountzis et al., 2015), was never detected with specific probes in worker tissues of the *Atta *colonies that we examined (Figure 2), consistent with the MiSeq data plotted in Figure 1 and Supporting information Figure S3 showing that *RhiAcro1 *is rare in *Atta* colonies. However, the *RhiAcro1* OTU was as highly abundant in the hindgut of *T. cornetzi* as in *Acromyrmex* species (Sapountzis et al., 2015)*,* but Mollicutes signals in *T. cornetzi* were sparse and restricted to the fat body and ileum (Supporting information Results S2; Figure 2). In both *S. amabilis *and *T. zeteki, *which belong to a phylogenetically more basal lineage of higher attine ants (Branstetter et al., 2017; Schultz & Brady, 2008; Sosa‐Calvo et al., 2018), there were very few *RhiAcro1 *signals, but Mollicutes OTUs could be stained in most of the tissues examined (Figure 2). It is important to note, however, that these Mollicutes were a different OTU (*EntAcro10*) than the *EntAcro1* bacteria that were exclusively found in *Atta*, *Acromyrmex* and *T. cornetzi* (Figure 1; Supporting information Figure S3 and Table S3). We detected no Actinobacteria signals in the abdominal tissues of *S. amabilis* and *T. zeteki* even though the MiSeq sequencing showed a low relative abundance of *ActAcro1* across the 15 colonies of *S. amabilis* and *T. zeteki* examined (usually <3%–4% but sometimes as high as 14% in a single laboratory colony; Tz_l1; Table S3; using all 107 samples as this result is independent of confounding effects). We never detected any endosymbiotic Actinobacteria in the abdomens of the evolutionarily more derived *T. cornetzi,* *Acromyrmex* and *Atta* with FISH, consistent with them being absent or sparse (Table S3) in spite of *T. cornetzi* and all three *Acromyrmex* species maintaining cuticular biofilms in which *ActAcro1* is the dominant OTU (Andersen, Hansen, Sapountzis, Sørensen, & Boomsma, 2013).

Our FISH survey only rarely detected general bacterial signals in abdominal tissues of *Cyphomyrmex* and *Myc. smithii *workers and we never observed specific signals in abdominal tissues of *Myr. ednaella* or *Ap. dentigerum* (Figure 2, Supporting information Results S2). These absences were confirmed using a wide range of specific FISH probes, and neither could bacterial DNA be stained with DAPI in replicated samples of multiple colonies of these species (Figure 2, Supporting information Results S2). The qPCR analyses of pooled entire abdomens (gasters) across the attine ants for which we obtained FISH data (Table S5) confirmed that overall bacterial titres in lower attines were low and part of a strong phylogenetic signal of increasing bacterial titres towards the crown of the phylogenetic tree (*K* = 0.113, *p* = 0.001; Figure 2). This change in overall bacterial abundances was not a smooth gradient, but had an unusually steep increase at the “A” transition within the *Trachymyrmex *grade that we defined above based on two alpha diversity measures also highlighting this transition (see previous section and Supporting information Figure S4). The fold‐increase in bacterial 16S DNA relative to that found in *Ap. dentigerum* (standardized to the ant housekeeping gene EF‐1α) was 2.54 ± 0.56 above the “A”‐transition and −0.08 ± 0.48 below, indicating that bacterial titres essentially remain unchanged until they realize a stepwise increase to higher abundance at the “A” transition. This transition explained differences in bacterial titre much better than the traditional transitions from lower attine subsistence farming to higher attine domesticated farming and to industrial‐scale herbivorous leaf‐cutting farming (evidence ratio = 6.00).

**Figure 3:**
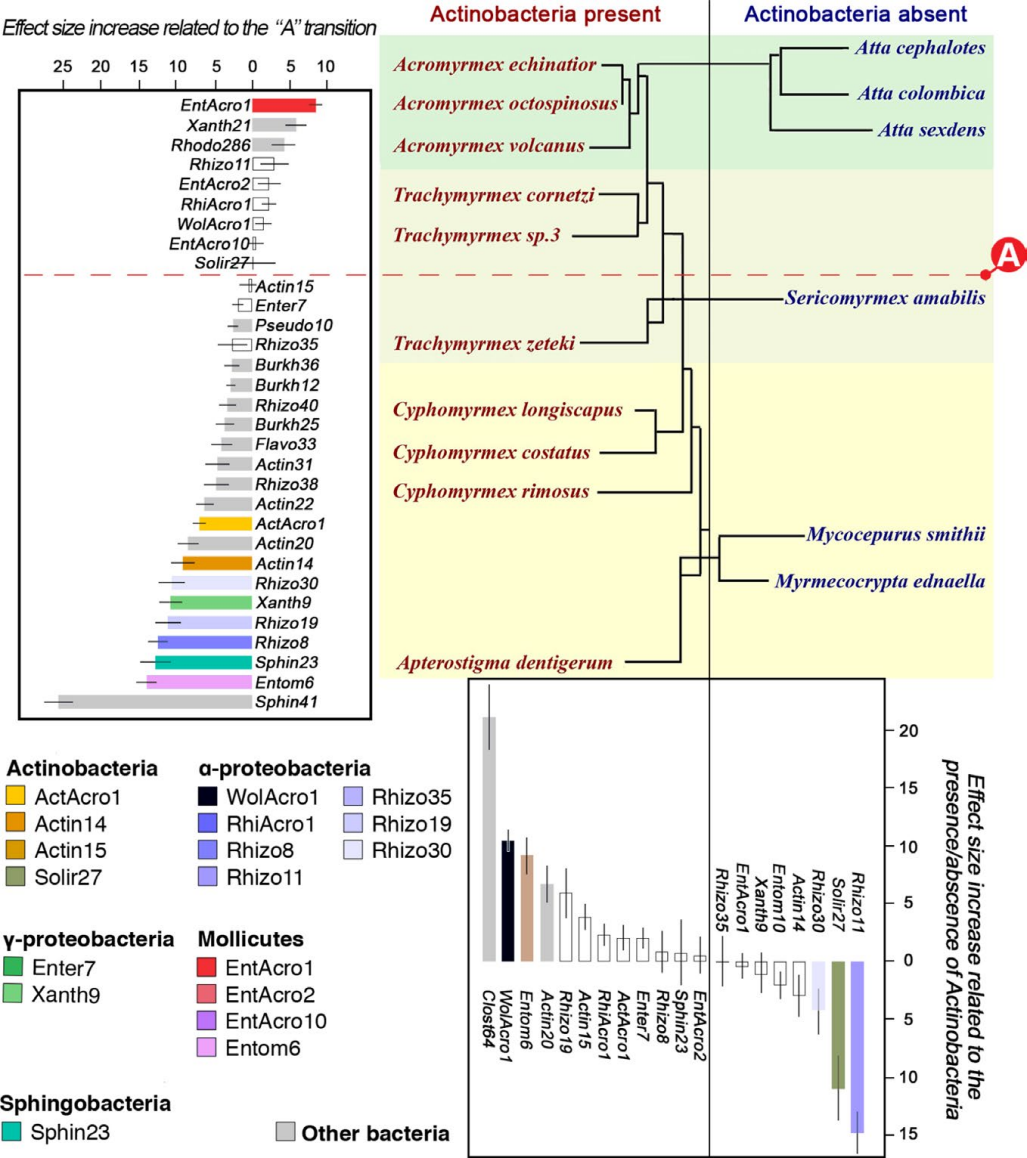
Patterns of differential representation of abdominal bacterial OTUs across sixteen Panamanian fungus‐growing ant species, based on all 2099 OTUs. The ant phylogeny of Figure 1 is redrawn here in such a way that species with and without visible covers of cuticular Actinobacteria are separated into left and right columns, with the traditional farming transitions highlighted by the same background colour shifts as in Figures 1 and 2 and Supporting information Figure S4, and the “A” shift identified in Figure 2 added as a red dashed horizontal line contrasting with the vertical black line highlighting the presence/absence of cuticular actinobacterial biofilms. deseq2 analyses (Supporting information Table S6) showed that both partitions affected the proportional representation of a series of OTUs, of which we plotted all 18 abundant OTUs and the 14 remaining less common OTUs for which differential representation was significant at *p* < 0.05. Bars show effect size differences ± *SE* for each OTU as identified by the deseq2 analysis (Supporting information Table S6). Bar colours for specific OTUs are the same as in Figure 1 (legend bottom left), and grey bars present the 14 nonabundant OTUs across both histograms, which are not plotted in Figure 1 but for which representations were significantly different in the present analysis. Open white bars represent abundant OTUs whose differential representation was not affected by either of the two focal partitions

The apparent “A” transition in overall abundance of abdominal endosymbiotic bacteria prompted us to further evaluate the extent to which the composition and distribution of bacterial communities might also reflect this transition. Using our original data set of 3,103 unrarefied OTUs belonging to 48 bacterial classes and the deseq software for analysis (Love et al., 2014), it appeared that, at the level of bacterial classes, Actinobacteria, Betaproteobacteria, Clostridia and Sphingobacteria were significantly more prevalent in the abdomens of the attine species basal to the “A” transition, while Alphaproteobacteria were more prevalent beyond the “A” transition (deseq adjusted *p* < 0.001 in all contrasts). At the OTU level, *EntAcro1* (Mollicutes) and the nonabundant *Xanth21 *(Gammaproteobacteria) and *Rhodo286* (Alphaproteobacteria) were significantly more prevalent in the evolutionarily derived attine lineages (deseq adjusted *p* < 0.001, *p* < 0.001 and *p* = 0.03, respectively; Figure 3 and Supporting information Table S6), while 19 other OTUs belonging to diverse bacterial classes were significantly more prevalent in the basal attine lineages (Figure 3 and Supporting information Table S6). Of the 22 OTUs that showed significant differences in proportional representation (Supporting information Table S6), nine were among the 18 most abundant OTUs previously identified with our double >10% criterion. Remarkably, *ActAcro1*, the dominant Actinobacteria on the cuticle of *Acromyrmex* species in Gamboa (Andersen et al., 2013), was one of the significantly underrepresented OTUs in the abdominal tissues of this evolutionarily derived group of higher attine ants, indicating that this OTU is only a relatively common endosymbiont associated with guts and abdominal organs throughout the basal attine lineages (lower attines plus *T. zeteki* and *S. amabilis*) where the overall bacterial abundances tend to be low (Figure 2 and Supporting information Table S6).

### 3.3 Sensitivity of abdominal microbiomes to cuticular Actinobacteria and sampling environment

After phylogenetic effects had been removed, there was no overall association between the presence/absence of Actinobacteria and bacterial OTU richness (mcmcglmm: *p* = 0.873; Supporting information Table S7A), but colonies that had been kept in the laboratory for 3 months generally had abdominal microbiomes with lower richness than field colonies (*p* = 0.007), although they did not differ in richness from those kept in the laboratory for several years (*p* = 0.222; Supporting information Table S7A). PD (Faith's Phylogenetic Diversity) followed the same pattern (Actinobacteria presence: *p* = 0.274; short‐term laboratory vs. field: *p* = 0.0109; short‐term laboratory vs. long‐term laboratory: *p* = 0.66; Supporting information Table S7B). For the other alpha diversity indices, there were no significant associations with either the presence/absence of cuticular Actinobacteria or sampling environment (Supporting information Table S7C,D).

We subsequently examined changes in specific OTUs between field and laboratory colonies, to see whether transfers systematically favoured the prevalence of some OTUs over others. This showed that *Atta *leaf‐cutting ant species tended to gain the dominant OTUs *EntAcro10*, *RhiAcro1* and *Rhizo11* that were mostly absent in the field (Figure 1), but only the *Rhizo11* changes were statistically significant (deseq2: effect size* *= 0.203, *p* = 0.956; effect size* *= 0.586, *p* = 0.823; effect size* *= 5.807, *p* = 0.033, respectively). Among the remaining 15 abundant OTUs, *Entom6*, *Enter7* and *EntAcro2* significantly increased in laboratory colonies as well (deseq2: effect size* *= 3.810, *p* = 0.045; effect size* *= 2.663, *p* = 0.033; effect size* *= 4.706, *p* = 0.033, respectively), but these significances also barely passed the 5% threshold, so may not be meaningful because we did not correct for repeated testing. The abundant OTUs *EntAcro1* in *Atta* and *Acromyrmex*, *RhiAcro1* in *Ac. octospinosus* and *T. cornetzi*, *EntAcro10* in *T. zeteki,* *ActAcro1* in *C. costatus*, *Rhizo8* in *C. longiscapus*, and *WolAcro1* in *Acromyrmex* and *Ap. dentigerum *were hardly affected by transfers of colonies from the field to the laboratory (Figure 1, all deseq *p*‐values ≥0.2). We therefore conclude that, in spite of the suggestive differences between field and laboratory colonies for some of the ant species emanating from Figure 1, few of these represented statistically meaningful shifts in bacterial community.

While we could not recover a general association with alpha diversity of abdominal bacterial communities, the presence/absence of cuticular Actinobacteria did reveal shifts in specific representations of abdominal OTUs. We found that *WolAcro1*, *Entom6*, *Actin20* and *Clost64* were present in significantly higher prevalences in abdominal tissues of ants carrying cuticular Actinobacteria (Figure 3 and Supporting information Table S6; deseq2, adjusted *p* values for *Actin20 p* = 0.01, for the other three OTUs *p* < 0.001). Two other OTUs were largely specific for ants that lack cuticular Actinobacteria: a Rhizobiales (α‐Proteobacteria) OTU (*Rhizo11*) (*p* < 0.001) and a Solirubrobacterales (Actinobacteria) OTU (*Solir27*) (*p* = 0.03). These results suggest that the presence/absence of cuticular Actinobacteria on the propleural thorax plates of worker ants may encourage the growth of certain abdominal endosymbiont OTUs while inhibiting the growth of others.

Finally, we checked whether overall bacterial abundances in ant abdomens, as measured by qPCR, were affected by the presence/absence of cuticular Actinobacteria or by transfer of colonies to the laboratory. Our analysis based on entire abdomens revealed no association between bacterial titres and either sampling environment (mcmcglmm: *p* = 0.209; Supporting information Table S7E) or the presence/absence of Actinobacteria (*p* = 0.980; Supporting information Table S7E), nor for the statistical interaction term between these two main predictor variables (*p* = 0.513; Supporting information Table S7E). This was also the case when we used the uncorrected qPCR results from our mixed set of dissected abdomen samples and entire abdomen samples (Supporting information Results S3).

### 3.4 Phylogenetic and environmental effects on OTU community composition

We performed beta diversity analyses to evaluate whether there were any discontinuous shifts in the composition of endosymbiotic abdominal communities across the attine phylogeny, and whether any such putative shifts were affected by the presence/absence of cuticular Actinobacteria. At the same time, such analyses allowed us to evaluate whether or not transfer of colonies to a common laboratory environment tends to homogenize bacterial communities, that is, reduce the variance in OTU communities between colonies. Before doing these analyses, we checked whether confounding effects might have affected our beta diversity estimates (Supporting information Results S1). We found evidence for similar PCR method effects (justifying exclusion of the touchdown PCR samples also here) and for the number of individuals used for DNA extraction having affected the community of OTUs retrieved. However, we concluded that the latter confounding effect (explaining <9% of the variance in bacterial community composition) was minor relative to the much more pronounced between‐species effect that explained almost 40% of the variance (Supporting information Results S1).

**Figure 4:**
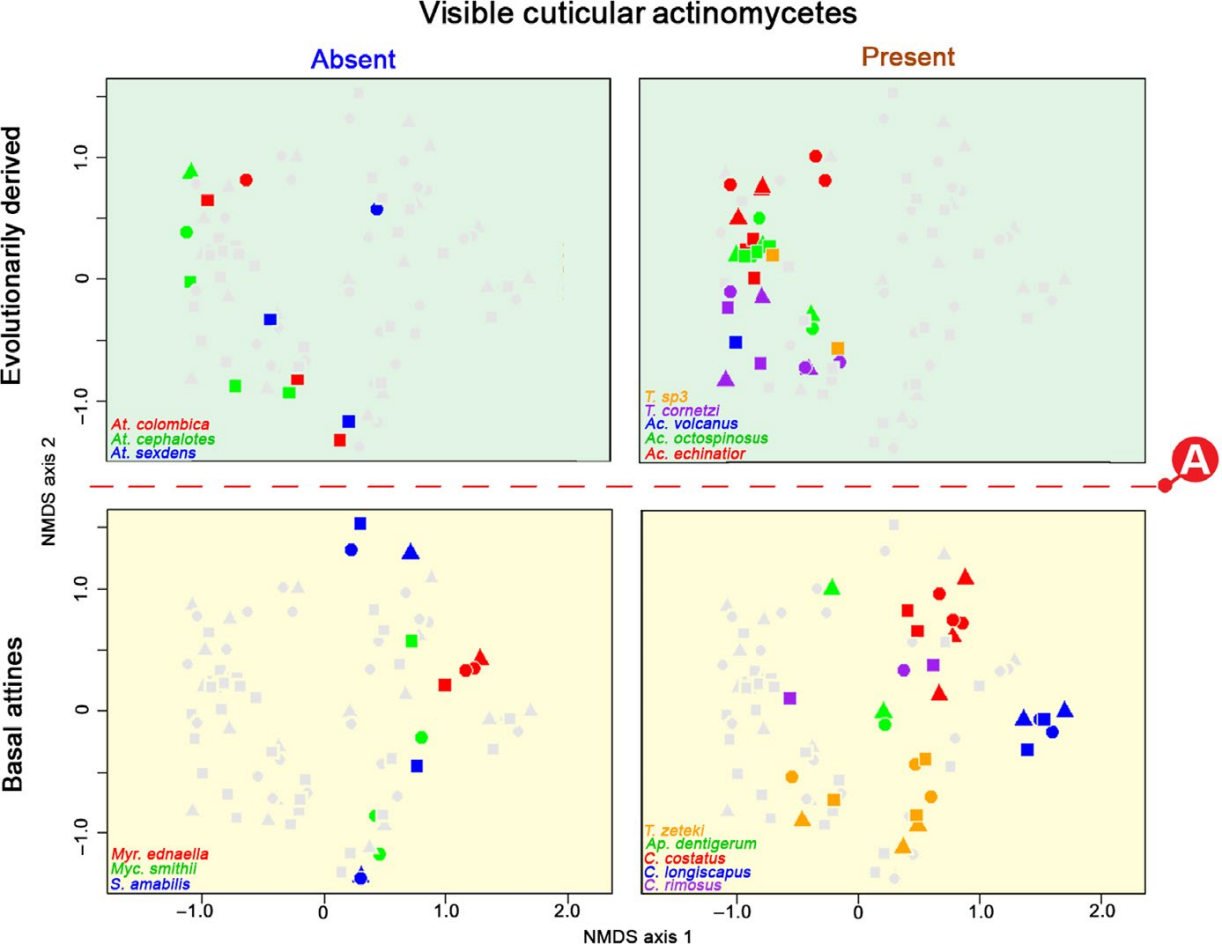
Composition of abdominal bacterial communities (beta diversity) partitioned according to phylogeny and presence/absence of cuticular Actinobacteria across 16 species of Panamanian fungus‐growing ants, based on 84 colony samples and using only the 18 most abundant OTUs. The ordination is based on nonmetric multidimensional scaling ordination of pairwise Bray–Curtis dissimilarities between all 84 colony samples amplified with standard PCR and had an overall stress factor of 0.208, which implies that the representation of total variation by just two axes was acceptable. The closer two samples are on the ordination plot, the more similar their bacterial communities. The three types of samples for each ant species were as follows: field colonies (circles), short‐term laboratory colonies (triangles) and long‐term laboratory colonies (squares). The four panels each have all 84 colony samples plotted, with samples from the focal quadrant coloured by species, and the remaining samples in grey

PERMANOVA showed that both the phylogenetic transition “A” and the presence of cuticular Actinobacteria had a significant effect on the mean compositions of abdominal bacterial communities (Figure 4) but that the former had a higher impact (*Pseudo‐F_1,78_* = 14.69, partial *R^2^* = 0.148, *p* < 0.001 and *Pseudo‐F_1,78_* = 3.747, partial *R^2^* = 0.038, *p* = 0.001, respectively). There was also an interaction between phylogenetic transition and the presence of Actinobacteria that just fell short of significance (*Pseudo‐F_1,78_* = 1.81, partial *R^2^* = 0.018, *p* = 0.082), while sampling environment had no overall impact (*Pseudo‐F_2,78_* = 0.530, partial *R^2^* = 0.011, *p* = 0.927). As these analyses focused on shifts in bacterial communities rather than on anonymous OTU richness or alpha diversity, the results suggest that transition “A” also carries a signature of change in beta diversity.

These changes in mean bacterial communities were also supported by our linear discriminant analysis (DA) results. Almost all (90.5%) of the colony‐level samples could be correctly assigned to above or below this transition based on their bacterial community composition (entropy *R^2^ *= 0.634), while the presence/absence of cuticular Actinobacteria could not be deduced from these community compositions (57.2% correctly assigned, *R^2^* = 0.101). Particularly, the evolutionarily derived attine species carrying Actinobacteria had very similar abdominal bacterial communities and could not be readily distinguished in discriminant analysis (58.7% correctly assigned, *R^2^* = 0.372), while all other quadrants of species were more distinct in their bacterial communities (evolutionarily derived ants without Actinobacteria: 66.7%, *R^2^* = 0.405; basal ants without Actinobacteria: 92.9%, *R^2^* = 0.593; basal ants bearing Actinobacteria: 82.8%, *R^2^* = 0.805). These discriminant analysis results did not change in any appreciable way when we used alternative weighted or unweighted UniFrac distance measures, or when we used all 2099 OTUs instead of only the 18 abundant ones (Supporting information Figure S5 and Table S9).

Finally, we used HOMOVAs to explicitly evaluate the changes in between‐sample variance in bacterial communities for the 18 abundant OTUs across the “A” transition and the possible codependence of such shifts on the presence/absence of cuticular Actinobacteria (Figure 4 and Supporting information Table S8). Using only the phylogenetic contrast, this confirmed that the evolutionarily derived attine ants (beyond transition “A” in Figure 2) had a significant reduction in the variability of their abdominal bacterial communities compared to the basal attines (*B* = 1.2494, *p* < 0.001 for Bray–Curtis distance; *B* = 1.805, *p* < 0.001 for weighted UniFrac distances; *B* = 0.988, *p* = 0.018 for unweighted UniFrac distances; Supporting information Table S8). The first two distance measures also indicated that the transition “A” effect on variability of OTU communities across samples was driven by the low variance in bacterial communities of the evolutionarily derived attine ants that carry cuticular Actinobacteria (*B* = 2.2194, *p* < 0.001 for Bray–Curtis distances and *B* = 2.9458, *p* < 0.001 for weighted UniFrac distances; Table S8). Using the full data set of 2099 OTUs produced a comparable result and this time the evolutionarily derived attine contrast between species with and without cuticular Actinobacteria was recovered for all three distance measures, in addition to unanimous support for the “A” transition contrast (Supporting information Table S8, Figure S5). Sampling environment was never a significant copredictor in any of the HOMOVA tests.

## 4 DISCUSSION

Our study is the first large‐scale comparative analysis of abdominal endosymbionts spanning the entire phylogenetic diversity of the ant tribe Attini. After the ancestor of these ants specialized on fungus farming ca. 55–60 MYA, the symbiosis was elaborated in two steps—first by fully domesticating a single lineage of crop fungi which allowed the ants to abandon less specific subsistence farming, and second by developing industrial‐scale leaf‐cutting farming in tighter co‐evolution with increasingly multinucleate and polyploid crop fungi that gradually adapted to decompose more nutritious fresh plant material (De Fine Licht et al., 2013; Kooij et al., 2015; Nygaard et al., 2016; Schultz & Brady, 2008; Shik et al., 2016). The attine ant symbiosis with fungus gardens became significantly enriched by cuticular Actinobacteria, which were acquired shortly after the origin of fungus farming and persisted in some, but not all later‐evolving attine genera where their main function appears to be the control of *Escovopsis* infections or other pathogens in fungus gardens (Barke et al., 2010; Currie et al., 2006; Fernandez‐Marin et al., 2013; Fernández‐Marín, Zimmerman, Nash, Boomsma, & Wcislo, 2009; Fernández‐Marín, Zimmerman, Rehner, & Wcislo, 2006; Gerardo et al., 2006; Haeder et al., 2009; Mattoso et al., 2012; Seipke et al., 2011). Our comparative survey of the composition, variation and robustness of the attine microbiomes associated with the gut and associated organs now adds a third layer of intriguing complexity to this symbiotic model system and strongly suggests that these abdominal microbiomes evolved in direct interaction with the attine ant hosts, the fungal cultivars and the presence/absence of actinobacterial microbiomes on the cuticle of the ants. We identified a clear transition in overall abundances of abdominal microbiota in between the domestication of gongylidia‐bearing cultivars and the origin of the *Atta *and *Acromyrmex* leaf‐cutting ants. This “A” transition (Figure 2), which showed significant contrasts in: (a) Our analyses of beta diversity (Figure 4; Supporting information Figure S5), (b) our OTU‐specific analyses (Figure 3) and (c) some of our alpha diversity analyses (Fig. S4) coincides with the colonization of Central/North America by the first attine ant lineage (Branstetter et al., 2017).

### 4.1 Less is more when fungus farming increases in scale and specialization

The overall diversity of the abdominal microbiomes across the attine ants is low. Although we obtained thousands of OTUs, only 21 of these were abundant across 107 samples (76 colonies) from 17 Panamanian ant species, a number that was reduced to 18 OTUs in the 84 samples from 16 ant species that we used for most of the analyses (representing >90% of all original reads). These findings are consistent with a previous study showing that more than 97% of the bacterial sequence reads obtained from the abdominal tissues of Panamanian *Acromyrmex* species is captured by just four of these OTUs (Sapountzis et al., 2015). The overall trends that we uncovered were that: (a) Abdominal bacteria have low or even negligible abundances in lower attine ants and their general abundance increases sharply at transition “A” (Figure 2), (b) This shift in abundance was reflected in patterns of beta diversity, indicating a discontinuous change in abdominal community composition, but whether alpha diversity decreased when abdominal bacterial titres increased remained unclear. Our correlative evidence suggested that multiple aspects of abdominal microbiome composition are affected by the presence/absence of cuticular Actinobacteria, particularly for the evolutionarily derived attines beyond transition “A.” We concentrated our sampling efforts on a single Panamanian site of ca. 25 km^2^, where it could reasonably be assumed that all ant species were exposed to the same environmental bacteria, so that noise from this source across genera and species of attine ants would be minimal. The extent to which our results are representative for other Latin American regions remains to be demonstrated and we hope that such studies will be pursued in the years to come.

We obtained several lines of evidence indicating that the most important transition in overall bacterial abundance and community composition (beta diversity) of abdominal microbiomes occurred after the origin of higher attine agriculture but well before the origin of industrial‐scale leaf‐cutting agriculture (Schultz & Brady, 2008). This transition seemed puzzling initially, but turned out to match a recent study showing that the ancestor of the crown group starting with the *T. cornetzi* branch (“A” in Figure 2) colonized the Central/North American subcontinent long before the Isthmus of Panama closed (Branstetter et al., 2017). This finding is intriguing because the founding queens of *T. cornetzi* are very small, which may have facilitated this unique event of long‐distance airborne dispersal. These undoubtedly severely bottlenecked colonizers were the first fungus growers in an entire “northern” subcontinent and started an adaptive radiation that ultimately produced the *Acromyrmex* and *Atta* leafcutter ants, which thus appear to have evolved in Central/North America rather than in South America as has previously been assumed (Branstetter et al., 2017). Only much later (presumably when the Isthmus was about to close) did representatives of *Acromyrmex* and *Atta* (whose queens are much heavier) colonize South America. The phylogenomic ancestral state reconstructions by Branstetter et al. (2017) imply that the “A” transition in abdominal microbiomes uncovered in our study coincides with what must have been the most significant vicariance event in the adaptive radiation of the attine ants. If our results would prove to have more general validity across Latin America, they could imply that obligate crop domestication, that is, the emergence of gongylidia‐bearing cultivars, affected gut microbiota less than the subsequent colonization of the Central/North American subcontinent. This is because bacterial species composition in the abdomens of Panamanian *T. zeteki* and *S. amabilis* remained rather similar to those of the lower attine ants included in our study. It is also striking that the common Mollicutes OTU *EntAcro10* was found in all genus‐level branches of the attine tree, but that the abundant leaf‐cutting ant OTU *EntAcro1 *was also found in *T. cornetzi* (Figure 1; see also Sapountzis, Zhukova, Shik, Schiott, & Boomsma, 2018). Also, the α‐Proteobacteria (mostly Rhizobiales and *Wolbachia* in *Acromyrmex*) begin to rise to abundance from the *T. cornetzi* lineage onwards only to be reduced to minor significance again in *Atta*.

### 4.2 Putative functions of abundant abdominal endosymbionts

As new attine genera emerged over evolutionary time, a few specific OTUs became dominant (Figure 1), suggesting that these eclectic higher attine symbionts came to provide specific mutualistic services when ant fungus farming became more advanced. Rhizobiales are particularly abundant in higher attine guts beyond the “A”‐transition, where they are largely restricted to the hindgut (rectum and ileum) in *T. cornetzi*, similar to *Acromyrmex* species where an earlier study recovered both *nifH* sequences and NifH proteins from the hindgut biofilms of *RhiAcro1* bacteria. This suggested that at least some strains encompassed by the *RhiAcro1* OTU are able to retrieve nitrogen for the farming symbiosis (Sapountzis et al., 2015), but alternative functions are also possible (Bradley & Philips, 1977; Donini & Odonnell, 2005; Engel & Moran, 2013; Russell et al., 2009; Sapountzis et al., 2015). The exclusive presence of *RhiAcro1* in Panamanian *Ac. echinatior*, *Ac. octospinosus*, *T. cornetzi* and *T. sp. 3* makes it of interest to find out whether similar patterns and putative functions can be found in other *Acromyrmex* species and representatives of the *T. cornetzi* clade that colonized South America, and in *T. septentrionalis* which is the sister lineage of the leaf‐cutting ants and occurs only in North America.

The other occasionally abundant α‐Proteobacterium associated with the abdominal organs was *Wolbachia*, but this symbiont (*WolAcro1*) was only found in consistent prevalences in *Acromyrmex* species and, to a lesser extent, *Ap. dentigerum* (Figure 1), confirming previous findings (Andersen et al., 2012; Frost, Fernandez‐Marin, Smith, & Hughes, 2010; Sapountzis et al., 2015). These bacteria primarily colonize nonreproductive tissues in *Acromyrmex *(Andersen et al., 2012; Frost, Pollock, Smith, & Hughes, 2014) where they can also occur extracellularly in the gut lumen, although it remains unknown how many bacterial cells may occur in this form. Our FISH location evidence suggests that *Wolbachia* cells are almost absent in somatic tissues of *Ap. dentigerum* but more abundant in the ovaries, whereas this appears to be the other way around in *Ac. echinatior* (P. Sapountzis, *unpublished observations*). This may be consistent with a putative mutualistic function of this symbiont in evolutionarily derived *Acromyrmex* leaf‐cutting ants (see Andersen et al. (2012) for a more detailed discussion) that did not evolve in the phylogenetically basal *Apterostigma*, but further work will be needed to clarify this.

The functional significance of the few but occasionally highly abundant Mollicutes OTUs remains enigmatic. It is very unlikely that they are pathogens (Anderson et al., 2012; Funaro et al., 2011; Kautz, Rubin, Russell, & Moreau, 2013; Meirelles et al., 2016; Sapountzis et al., 2015) and rather suggests that the high prevalences of *EntAcro1* in the leaf‐cutting ants may be related to actively herbivorous foraging habits (De Fine Licht & Boomsma, 2010) which also implies the ingestion of plant sap and fruit juices (Littledyke & Cherrett, 1976). Their consistent localization in the fat body suggests that beneficial interactions with energy and nutrient decomposition processes are likely (Arrese & Soulages, 2010), particularly because some close relatives of the two most common *EntAcro1* and *EntAcro10* OTUs are associated with other insects with specialized diets where they may be mutualists (Lo, Ku, Chen, Chang, & Kuo, 2013). Detoxification and immune defence functions are also a possibility when these bacteria occur in the Malpighian tubules as is commonly observed (Beyenbach, Skaer, & Dow, 2010; Dow, 2009). A possible connection with chitin monomer degradation has been suggested by Sapountzis et al., (2015), with recent reinforcement from an extensive comparative genomics analysis across the crown group of the same Panamanian attine ants as covered in our present study. That study revealed consistent signatures of positive selection for novel chitinase decomposition functions in ant genomes, likely in response to positive selection for chitin synthases in the fungal cultivars (Nygaard et al., 2016). The chitin metabolism hypothesis is also, albeit indirectly, supported by our 16S‐sequencing results showing that several *Chitinophaga* OTUs (order Sphingobacteriales, class Sphingobacteria) occur in the abdomens of some *C. longiscapus* and *Myr. ednaella* workers (Supporting information Table S3). These two species happen to be the only attines where Mollicutes were always sparse (<4% across 107 samples; see Supporting information Table S3), but any such inferences remain provisional and will need explicit validation. We address the functional significance of *EntAcro1* and *EntAcro10* in a parallel study (Sapountzis et al., 2018).

The most abundant actinobacterial OTU associated with the abdomens of attine ants was *ActAcro1*, a *Pseudonocardia* that has previously been found in minor (<1%) prevalences in the abdomens of some Panamanian *Acromyrmex* workers (Sapountzis et al., 2015), where it is primarily an obligate cuticular ectosymbiont producing antibiotics to help control *Escovopsis* infections (Currie, Bot, & Boomsma, 2003). The same OTU now appears to be relatively prevalent in the sparse endosymbiotic microbiomes of lower attine ants (Figure 1; mean prevalence per species 5%–37%) with the exception of *C. longiscapus* where it is very sparse (mean prevalence <0.3%). These numbers changed only slightly when separately considering the abdominal microbiomes of workers in field colonies, because prevalences of *ActAcro1 *in the abdomens of lower attines tended to remain similar (6%–36%) after transfer of colonies to the laboratory. The prevalences of *ActAcro1* in *Myr. ednaella* were in fact higher in field colonies (16%–24%) than in laboratory colonies (2%–5%). These consistent presences of *Pseudonocardia *in lower attine abdomens can therefore not be explained by contamination of the gut after ingesting cuticular *Pseudonocardia*, also because *ActAcro1 *was present in lower attines that lack cuticular Actinobacteria (the *Myrmicocrypta* and *Mycocepurus* used in our study), in *Cyphomyrmex* lower attines where cuticular *Pseudonocardia* is absent or rare (Andersen et al., 2013; Innocent et al., unpublished results), and in higher attines such as *T. zeteki* that have cuticular actinobacterial biofilms with other bacterial species than *Pseudonocardia *(Andersen et al., 2013; Sen et al., 2009). This could imply that *ActAcro1 *was initially a gut or abdominal‐organ symbiont, whose antibiotic functions were later co‐opted for external hygienic use in the evolutionarily derived higher attine ants. This hypothesis deserves further testing because it may shed novel light on the ongoing discussion of whether and to what extent *Pseudonocardia* strains co‐evolved with the attine ant symbiosis and its *Escovopsis *mycopathogens (Cafaro et al., 2011; de Man et al., 2016; Heine et al., 2018; Holmes et al., 2016; Sen et al., 2009).

Members of γ‐Proteobacteria were found in the attine ant abdomens but only in traces and then primarily in the rectum (Figure 2). This finding is of interest because representatives of γ‐Proteobacteria have repeatedly been reported as being abundant in the fungus gardens of attine ants (Aylward et al., 2014, 2012; Kellner, Ishak, Linksvayer, & Mueller, 2015; Pinto‐Tomás et al., 2009), although it remains unclear whether these were the same OTUs or not. These previously reported γ‐Proteobacteria are mostly Enterobacteriaceae with abilities to fix nitrogen in laboratory fungus gardens of *Atta* leaf‐cutting ants (Pinto‐Tomás et al., 2009). They not only occur abundantly in fungus gardens (Aylward et al., 2014) but also have a substantial presence in the abdominal microbiomes of leaf‐cutting ant larvae who only ingest fungal food, suggesting they may have important metabolic roles during development (Zhukova, Sapountzis, Schiøtt, & Boomsma, 2017). However, the scattered pieces of evidence for nitrogen supplementation by bacterial symbionts, suggested for the hindgut of *Acromyrmex *workers (Sapountzis et al., 2015) and shown for *Atta *fungus garden (Pinto‐Tomás et al., 2009), are as yet insufficient to understand the bigger picture of nitrogen turnover in attine ants (Shik et al., 2016). Nitrogen supplementation by bacterial symbionts has been suggested to also be important in other ant species with nitrogen limited diets (Russell et al., 2009; van Borm, Buschinger, Boomsma, & Billen, 2002), but more work is needed to resolve when and why during attine ant evolution nitrogen became a limiting factor for mutualistic productivity and whether nitrogen supplementation in the hindgut and fungus garden would tend to complement or exclude each other.

### 4.3 Interactions between thoracic cuticular Actinobacteria and abdominal microbiomes

We obtained multiple pieces of correlative evidence suggesting that the presence or absence of cuticular Actinobacteria affects the composition of abdominal microbiomes (Figures 3 and 4; Supporting information Figure S5; Tables S8 and S9), but not their overall abundances which seem to be largely dependent on the phylogenetic position of ant species and genera (Figure 2; Supporting information Results S3). These effects appear to be complex, to probably interact with the “A” transition, and to be mostly reflected in distributional shifts among rarer OTUs rather than the abundant ones (Figure 3). Given the known production of antibacterials by the *ActAcro1* cuticular *Pseudonocardia* symbiont (Holmes et al., 2016), these interactive effects do not come as a surprise, but they will require functional experiments to assess whether and to what extent specific bacterial OTUs benefit from the presence of cuticular actinobacterial antibiotics or are inhibited.

Our quantitative comparisons of shifts in community composition of abdominal bacteria and the general robustness of these communities across sampling environments showed that ant species that carry cuticular Actinobacteria tend to have more similar abdominal microbiomes than those without, particularly in the evolutionarily derived attine lineages (Figure 4; Supporting information Figure S5). This implies that several attine ant species may have abdominal microbiota that could be actively protected by cuticular antibiotic‐producing biofilms against inadvertent colonizers that may compete with “native” abdominal endosymbionts. This hypothesis will also need to be tested experimentally.

### 4.4 Caveats and future perspectives

Our comparative 16S‐metagenomic study adds significant insights into the complexity of the attine ant fungus farming symbiosis across their phylogenetic tree, but surveys of this kind cannot resolve hidden diversity that may exist between closely related symbionts that share identical or very similar (>97%) 16S sequences (Andersen et al., 2012; Engel, Stepanauskas, & Moran, 2014; Kuo, 2015). They also do not provide direct information on the function of symbiotic OTUs, so the correlative trends and inferences obtained in our present study need to be followed up by more extensive microscopy of bacteria from field‐collected attines (see e.g., Sapountzis et al., 2015) and by functional genomic studies of the handful of abundant bacterial symbionts that we identified and mapped onto abdominal tissues and organs after FISH (e.g. Sapountzis et al., 2018). Finally, our survey remained restricted to central Panama and needs to be repeated in South America where extant diversity of attine ants is higher. Comparisons between attine ant communities in Central and South America have become relevant in a novel way after Branstetter et al. (2017) showed (in sharp contrast to previous belief; e.g., Kusnezov, 1963) that most of the crown group of the higher attine ants (including the leaf‐cutting ants) evolved in Central/North America. If the discontinuous change in abdominal microbiomes that we identified as the “A”‐transition does indeed represent a fundamental reorganization of the abdominal microbiome upon colonization of a new American subcontinent, our results would predict that extant representatives of the *Trachymyrmex*, *Acromyrmex* and *Atta* lineages that independently colonized South America without having evolved there should have retained phylogenetic signatures of their Central/North American origin in their extant abdominal microbiomes, and that those signatures should be similar to what can be found in exclusively North American species of *Trachymyrmex*, *Acromyrmex* and *Atta*.

## CONFLICT OF INTEREST

The authors declare that they have no competing interests.

## AUTHOR CONTRIBUTION

P.S. performed the experiments. P.S., M.S. and J.J.B. designed the study and wrote the manuscript. P.S. and D.R.N. analysed the data and designed the different types of statistical analysis, D.R.N. helped evaluate the results, conceived and edited part of the figures and contributed to the final stages of the writing. All authors read and approved the final version of the manuscript.

## Data Availability

Sequences produced for this study are available via the Sequence Reads Archive (https://www.ncbi.nlm.nih.gov/sra) under SRA study accessions: SAMN03460089–SAMN03460167 and SAMN03460169–SAMN03460177. OTU tables (Supporting information Table S3) and Results S2 are available on dryad: https://doi.org/10.5061/dryad.tj30d.
