# Supplementary material for: The evolution of abdominal microbiomes in fungus‐growing ants
Source: Mol Ecol. 2018 Dec 10;28(4):879–99. doi: 10.1111/mec.14931 (PMC6446810; doi:10.1111/mec.14931)
Supplement: Supplementary file 1 [file MEC-28-879-s001.docx]

**Supplementary Results 1 – Validation of assumptions that samples were fully comparable for alpha- and beta-diversity analyses**

Confounding effects due to unintended differences among samples are a common problem in comparative 16S-microbiome studies. Such differences can be due to sample preparation (different number of individuals collected, different amplification methods for sequencing library construction, and different DNA extraction methods), but they can also be generated by random contaminants (during field collection, lab processing, lab airconditioning draft, or contaminated reagents) or by differences related to the origin of samples (differences in biomass, tissue identity, and presence/absence of PCR inhibitors). An earlier study (Salter et al., 2014) has also shown that samples with low microbial biomass are more sensitive to contamination from bacterial DNA present in lab reagents. Our initial analyses showed that confounding effects may exist because comparisons of overall bacterial abundances (qPCR data) in the bodies of different species (Fig. 2 and Supplementary Results 3) showed that several lower attine ant samples (the genera *Cyphomyrmex*, *Myrmicocrypta*, *Mycocepurus* and *Apterostigma*) had almost 2.5 orders of magnitude lower abdominal bacterial titers than the remaining ant samples, while the alpha diversity of their abdominal microbial communities was elevated compared to the attine ant samples with higher bacterial titers (Fig. S4).

We thus performed a set of extra analyses to examine whether potential confounding effects were related to: i) the low biomass available in several of these lower attine ant samples, ii) the presence of lab-borne contaminant sequences, iii) the different PCR amplification methods that were used in the sequencing center that handled our samples (standard and touchdown PCR; see methods), or iv) the higher number of individual ants homogenized for some of our lower attine samples.

*Effects of low biomass on alpha-diversity measures*

The qPCR analyses (Fig. 2) gave us a measure of bacterial density (titer) that is independent of our measures of alpha diversity from a subset of the same samples that were used for MiSeq. Hence we could directly examine the relationship between bacterial density and alpha diversity. Using the 35 samples for which we had both qPCR and MiSeq data, we repeatedly found a negative relationship between measures of alpha diversity and bacterial density (log_e_ OTU Richness: r = -0.64, F_1,33_=22.3, p < 0.0001; Shannon diversity: r = -0.37, F_1,33_=5.35, p = 0.027; log_e_PD: r = -0.60, F_1,33_=18.7, p < 0.0001; Theil inequality: r = -0.37, F_1,33_=5.31, p = 0.027). This trend is expected both under the hypothesis of transition from low to high bacterial titers being accompanied by a shift to lower diversity microbiomes, and under the alternative hypothesis that the relationship is an unintended artefact due to contamination. However, in the former case we would expect to see no association between bacterial density and diversity within ant species, while in the latter case (if the relationship was driven by contamination) bacterial diversity and bacterial density should be negatively correlated to similar degrees as within species (c.f. Hu et al., 2017). When we examined the within-species and between-species patterns, there was no consistent within-species relationship between density and diversity, both for richness (7 positive slopes versus 5 negative slopes) and for Shannon diversity (6 positive slopes versus 6 negative slopes). There was also no indication of slopes towards the left in the plot being more likely to be negative than those towards the right (Figure SR1.1). These results suggest that the low biomass in the lower attine ant samples had not substantially affected the alpha-diversity estimates obtained for the lower attine ant samples.


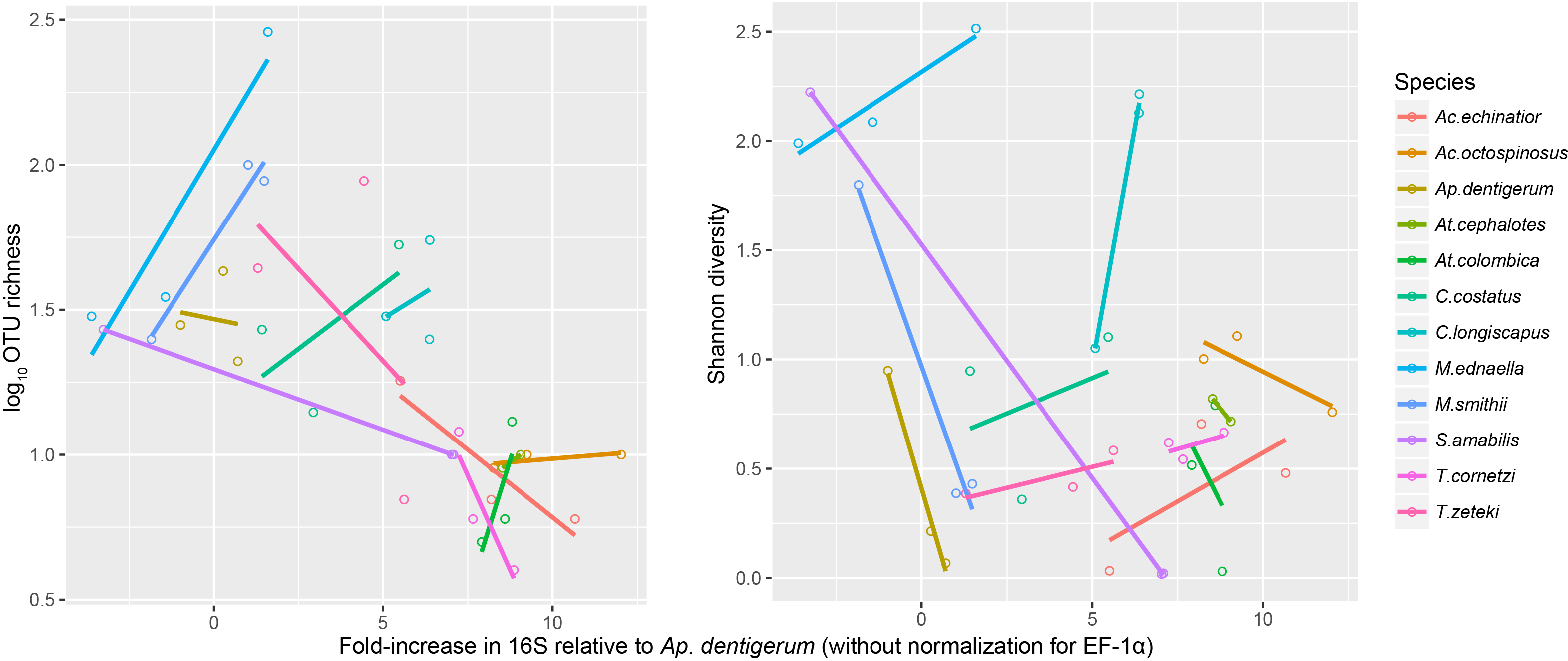


**Figure SR1.1: Correlations between bacterial titers and OTU richness (left) and Shannon diversity (right).** Samples and slopes are color-coded as indicated in the legend.

*Possible presence of lab-borne contaminants in our reagents and water samples*

Laboratory-borne contaminants in 16S-microbiome studies can be a common issue and are often visible as positive 16S-PCR signals in water samples and blank extractions (e.g. qPCR negative controls in Sanders et al., 2017). In the following pictures we present several of the 16S-PCR electrophoresis images of our lab-processed samples. In each image the blank extractions and/or water samples (negative PCR) are presented next to DNA samples extracted from attine ant abdomens or dissected tissues used to construct the 16S sequencing libraries (dates shown at the top). With the exception of the first PCR image from June07_2011 (AmpliTaq Gold, ThermoFischer Scientific) all other PCRs were performed either with Phusion High-Fidelity DNA Polymerase or with the AccuPrime Taq DNA Polymerase, high fidelity.


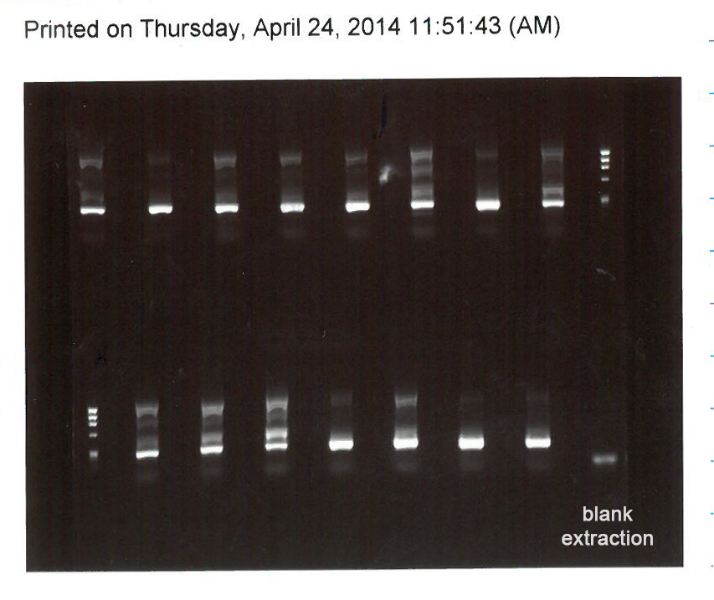

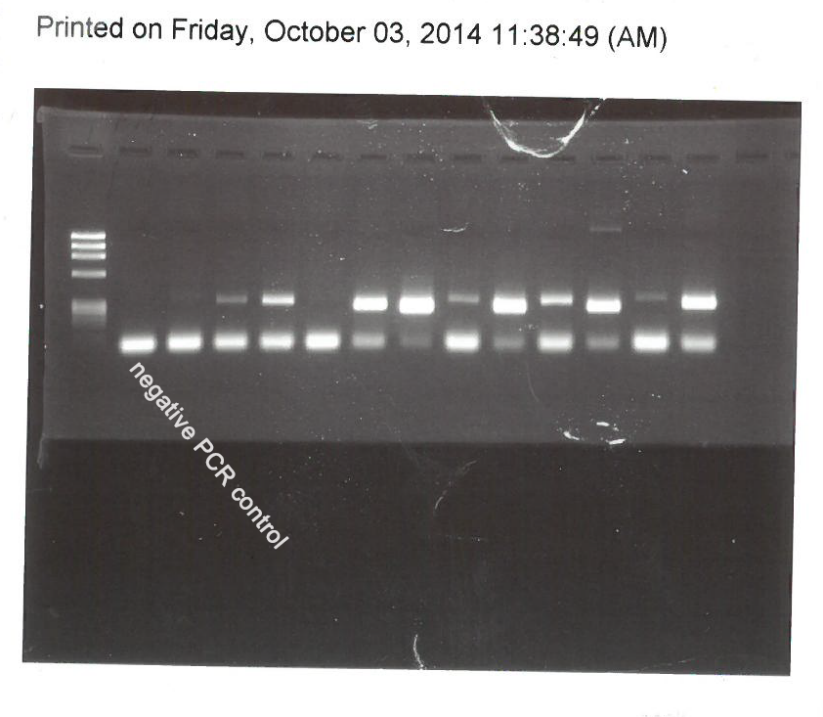

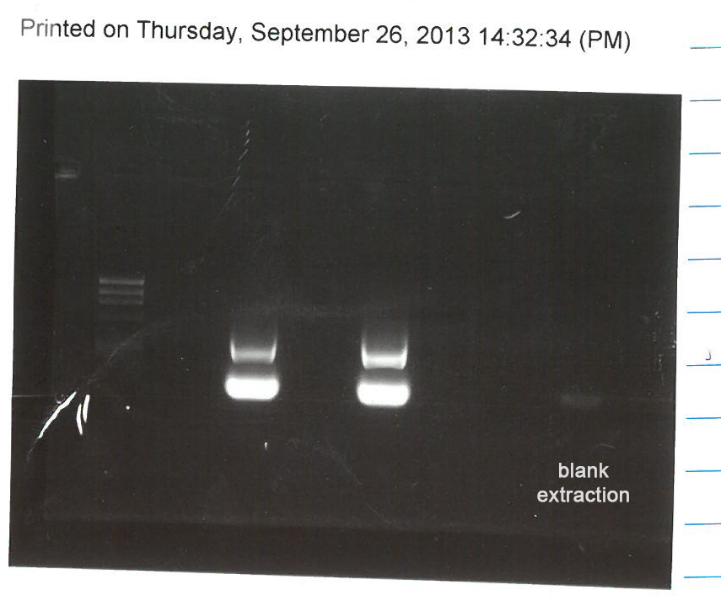

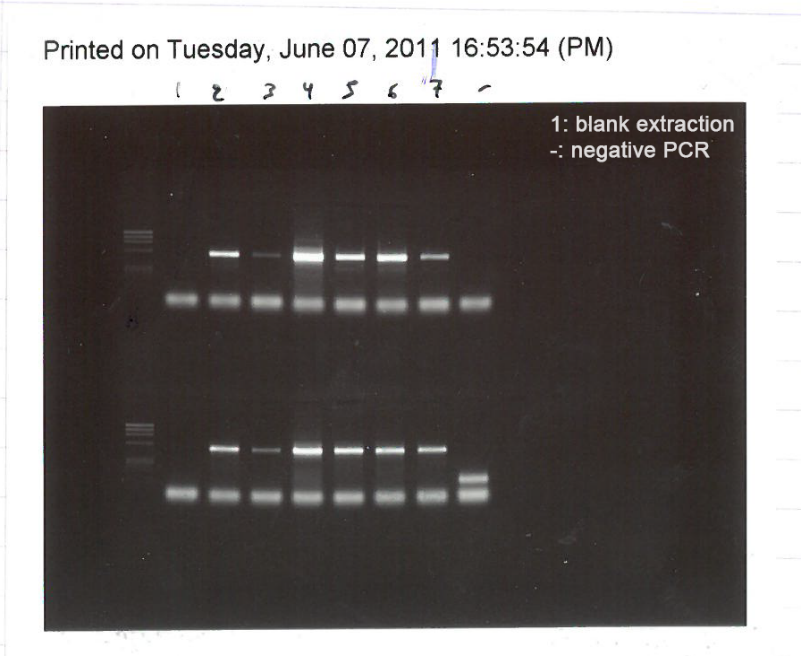


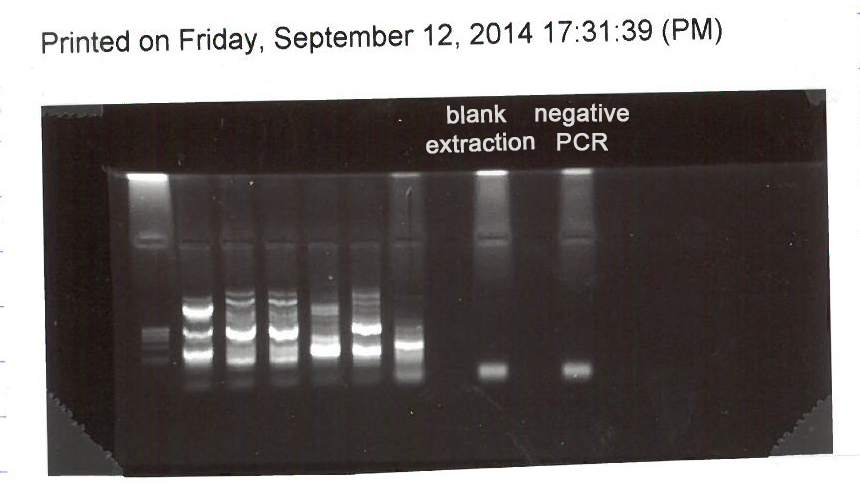


**Figure SR1.2: 16S-PCR electrophoresis images of the ant samples sent for MiSeq sequencing as well as several water samples and blank extractions samples.** Dates at which pictures were taken are presented above each image. All images have both blank and/or negative (water) control samples and samples of extracted DNA from ant tissues which produced positive signals as expected.

The absence of positive signals in the ‘blank extraction’ and the ‘negative PCR’ (water) samples suggested that potential lab-borne contaminants should have been infrequent and, if present, that they were below the PCR detection limit.

*Possible effects of lab-borne contaminants on estimates of alpha-diversity*

Even though the blank DNA extractions (Figure SR1.2) did not produce any detectable PCR products, sparse and under-detection-limit contaminants may still have been present in our samples. Assuming that some lab-borne contaminants might have been present in our samples, as suggested by both Hu et al., (2017) and Salter et al., (2014), we expected that low-density samples affected by contamination would have similar bacterial communities to those of blank water samples analyzed in the same lab (Fig. S1 of Hu et al. and Fig. 1 of Salter et al.), and that samples with lower density of target bacteria should yield bacterial communities more similar to the contaminant communities of blank controls. Hence we carried out an NMDS ordination (based on Bray-Curtis dissimilarities) of all 107 ant-samples, while also including the water blanks. In this analysis the water blanks showed up peripherally in the NMDS plot, indicating that the vast majority of ant samples did not show similarity to the OTU community of bacteria found in the water blanks (Figure S1.3).

However, this analysis did alert us to one sample that was very close in bacterial composition to the water blanks, which we then examined further. This sample had, indeed, a very low bacterial titer as measured by 16S amplification in qPCR – but it also had a very low EF-1α titer, suggesting that DNA extraction in general had not worked well. In such a case it is not surprising that contaminants are being amplified along with (or instead of) the target DNA. This was the same field sample of *Atta cephalotes* (Ace_f3) that was already excluded from the qPCR analysis (see Supplementary Results 3) for this reason.

In general, at the ordinal level, two of the three water blanks were dominated by single OTUs in the Pseudomonadales, and the third by an OTU belonging to the Caulobacteriales. These are orders (especially the Caulobacteriales) that were only minor components of the ant samples (Table S3), but were also common in the blanks of Hu et al., (2017). However, it is also true that several recent studies (e.g. Hu et al., 2017) have shown that more contaminant sequences are found when more blank/water samples are sequenced, so we realized that we would likely have missed some contaminants by having only three ‘blank’ water samples.

**
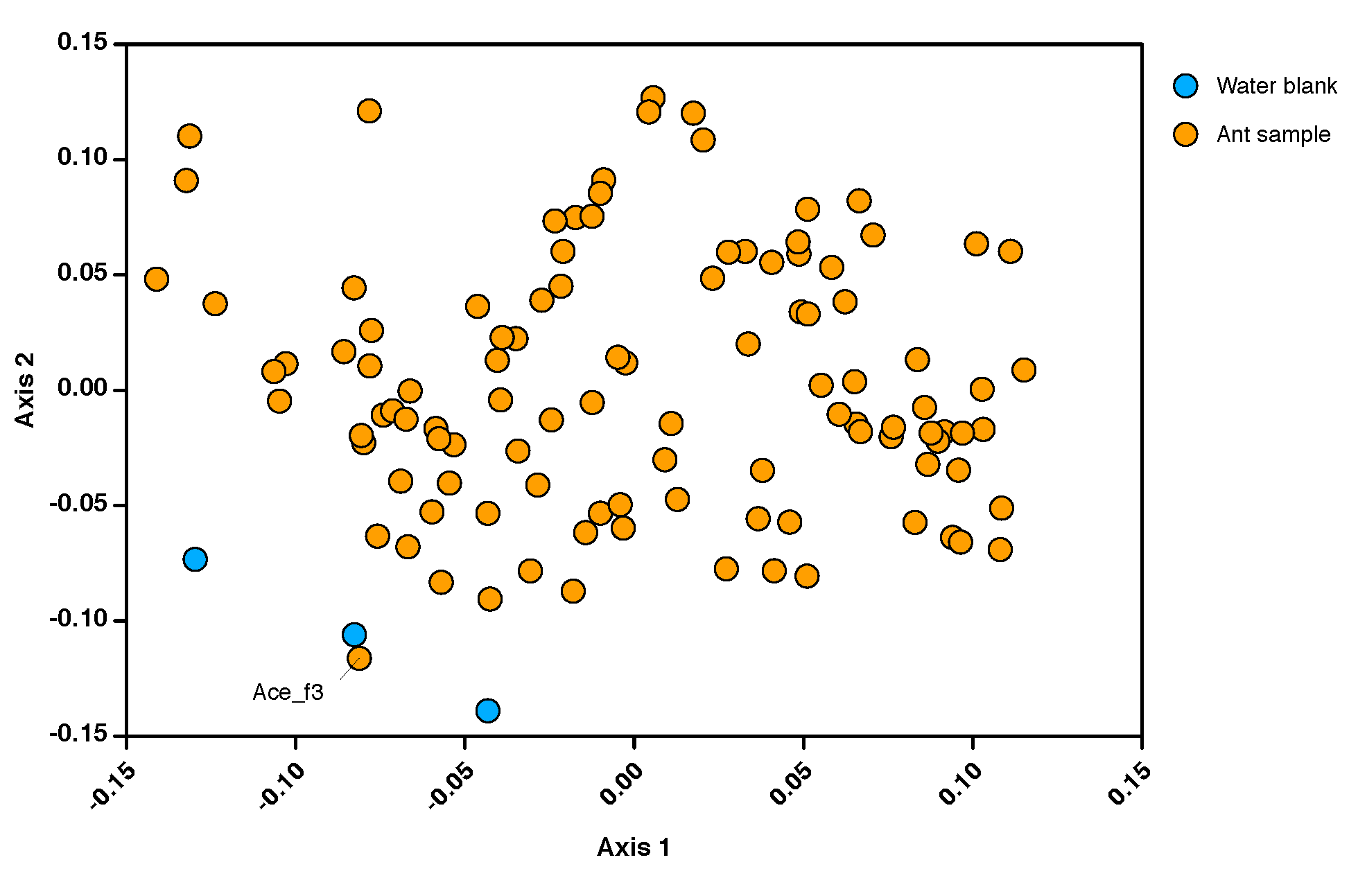
Figure SR1.3: Non-metric dimensional (NMDS) ordination of Bray-Curtis dissimilarity distances of all 107 samples using all 2099 OTUs after rarefication.** Samples are color-coded depending on whether they were ant samples or water blanks (see legend).

Therefore, even though we took all necessary precautions when preparing our samples (aseptic workflow, sterile reagents, 16S PCR using water samples and blank extractions), we cannot exclude that some of the sparse and low prevalence sequences identified in our dataset may have been lab-borne contaminants. We therefore: i) filtered our dataset using the three blank libraries that were included along with our samples as controls (see methods and below), and ii) used only the 18 most abundant OTUs for most of our analyses.

*Filtering of lab-borne contaminant sequences using OTUs or unique sequences*

Considering the above results which showed that even if lab-borne contaminants were not abundantly present in our dataset, they could have been among the OTUs in our data set, we identified the common OTUs between ant samples and blank samples (>97% identical, see methods) and excluded any OTUs for which prevalences produced an average blank/sample ratio ≥ 0.2 (see methods). However, our initial method was different from the original procedure proposed by Hu et al., (2017) who used unique sequences instead of OTUs, so we also extracted the unique sequences between the ant and the blank samples (instead of merely creating OTUs based on 97% similarity), compared them and identified the sequences which were common between the ant and the blank samples as potential contaminants. We then calculated the ratio of the prevalence of these common sequences in the blank/ant samples and considered all sequences where that ratio exceeded 0.2 as contaminants (henceforth referred to as filtering method 0.2). A table containing all unique sequences and information about their prevalence and distribution has been deposited at dryad (DOI: <https://doi.org/10.5061/dryad.tj30d>).

Using the filtered dataset we created a new OTU table (also at DOI: <https://doi.org/10.5061/dryad.tj30d>) and calculated the Sobs (number of Taxa = Richness), the Shannon diversity, the Theil inequality and the Faith’s phylogenetic diversity (PD). We then evaluated the degree to which our OTU-filtering method produced similar or identical alpha diversity indices to the more stringent unique-sequences filtering method using simple regression analysis, which produced the four plots below.

**Sobs**

Ac_f2

**Figure SR1.4: OTU** **Richness (Sobs) based on our original measurements (using a 0.2 filtering method directly on the OTUs) on the x-axis and the measurements that were produced after applying the Hu et al. (2017) procedure based on unique sequences (y-axis).** Only one sample showed some deviation in the predicted direction – the marked *Atta colombica* field sample (Ac_f2).

**Shannon**

Ac_f2

Adent_l1

Msmi_l1

**Figure SR1.5:** **OTU** **Shannon index values based on our original measurements (using a 0.2 filtering method directly on the OTUs) on the x-axis and the new measurements produced after applying the Hu et al. (2017) procedure based on unique sequences (y-axis).** Here scatter is slightly higher, but only three samples deviated in the direction predicted. One of these is the same Ac_f2 field sample (at x = ca. 3.4). The other two are a *Mycocepurus* sample (at x = ca. 1.8) and an *Apterostigma* sample (at x=ca. 2.5), both from lab colonies.

Tcorn_3m3

**Figure SR1.6: OTU** **Theil inequality** **values based on our original measurements (using a 0.2 filtering method directly on the OTUs) on the x-axis and the new measurements produced after applying the Hu et al. (2017) procedure based on unique sequences (y-axis)**. Here scatter is a bit higher, for one sample also in the unpredicted direction. The samples that seemed worth tracking came from two *Atta* species and a *T. cornetzi*, which had generally low diversity values and high bacterial titers. In addition, both *Atta* samples had been amplified with touchdown PCR (Table S1) and these samples were already removed from our analyses for reasons explained below (Figures SR1.8-SR1.10).

**PD**

As_l1

Ac_f2

Ace_f3

As_f1

**Figure SR1.7: OTU** **Faith’s phylogenetic diversity** **(PD)** **values based on our original measurements (using a 0.2 filtering method directly on the OTUs) on the x-axis and the new measurements produced after applying the Hu et al. (2017) procedure based on unique sequences (y-axis)**. Here scatter is low again, for one sample also in the unpredicted direction. In light of that apparent noise, only four samples seemed worth tracking - all four of these were from *Atta* species, which had generally low diversity values and high bacterial titers except for the *A. cephalotes* field sample Ace_f3, which had been amplified with touchdown PCR, similar to the *Atta colombica* field sample Ac_f2 (Table S1), and these samples were already removed from our analyses.

Considering that *R^2^* values were 0.99, 0.96, 0.86 and 0.93 and that almost all of the samples that showed a deviation were excluded from the main analyses in the manuscript (samples Ac_f2, Ace_f1, Ace_f2, Ace_f3, As_f1, Adent_l1, Msmi_l1 were amplified with touchdown PCR and therefore excluded), we inferred from these checks that the unique sequence-filtration method by Hu and our OTU-filtration method based on water sample-specific OTUs produced essentially the same results.

*Effects of PCR method and number of individuals homogenized on alpha diversity*

We also examined whether the use of two different PCR amplification methods (standard and touchdown) as well as the use of a different number of homogenized individual abdomens for some of the lower attine samples may have impacted the diversity measures that we obtained. We present these results in Figures SR1.8 and SR1.9, addressing Sobs and Shannon diversity (e^H^) estimates in the first figure and Theil inequality and PD in the second figure.


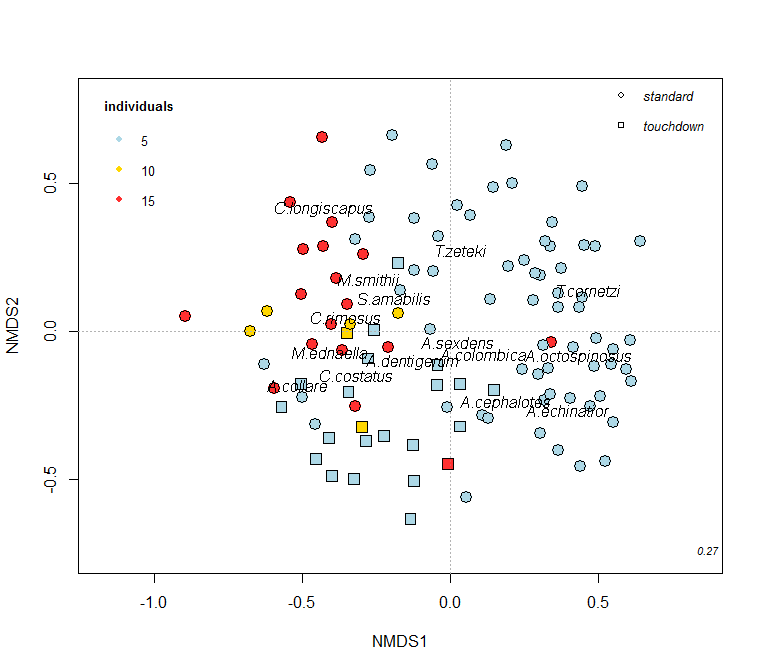

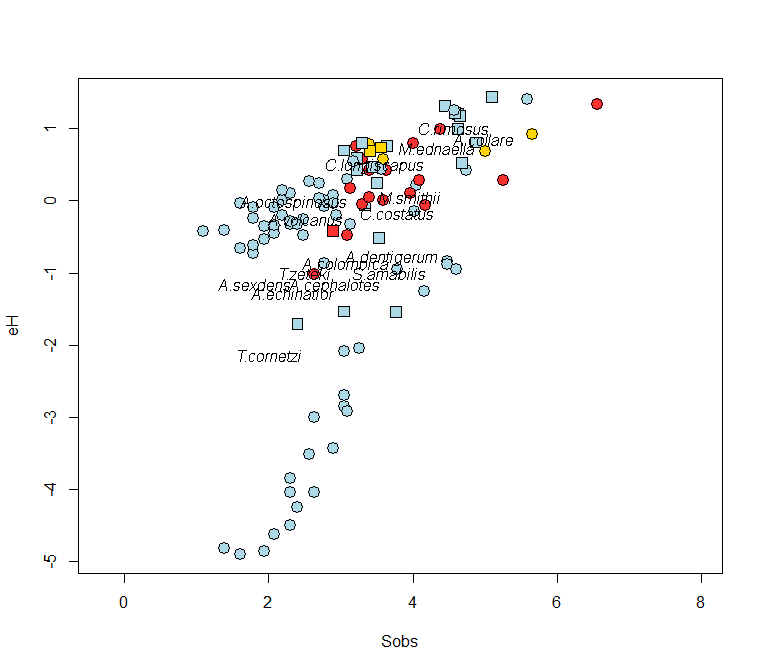


e^H^

**Figure SR1.8: Alpha diversity Sobs and Shannon values (logSobs and e*^H^*) of all 107 samples amplified by either standard or touchdown PCR.** Out of the 107 samples, 23 libraries were prepared with touchdown PCR (squares) because these particular samples could not be amplified with standard PCR (circles). These 23 samples were randomly distributed across the attine ant phylogeny: 3 *A.colombica*, 3 *A.cephalotes*, 2 *A.sexdens*, 3 *S.amabilis*, 1. *C.costatus*, 4 *M.smithii*, 2 *M.ednaella*, 4 *A.deptigerum* and a single *A.collare* sample. However, the number of individual abdomens homogenized for each sample covaried with diversity estimates across ant species. For *C. costatus*, *C. longiscapus*, *C. rimosus* and *M. ednaella* we collected 15, 15, 15 and 10 individuals per sample (red and yellow samples at the top) while estimates for all other species were based on five homogenized abdomens per sample (blue). After examining normality of the data distribution (which gave no indications of heteroscedasticity or deviations from normality) we used linear models in R, producing alpha diversity indices as response variables with PCR method, number of homogenized abdomens, and ant species as predictor variables. ANOVA tests showed that both PCR method and number of individuals collected had significantly affected the Sobs index (F=13.40, d.f=1, p<0.001 and F=11.38, d.f.=2, p<0.001, respectively) and the Shannon diversity (F=11.77, d.f=1, p<0.001 and F=7.73, d.f.=2, p<0.001, respectively) while the effect of ant species was borderline significant for Sobs (F=2.14, d.f=15, p=0.016) and not significant for Shannon (F=1.39, d.f.=15, p=0.174).


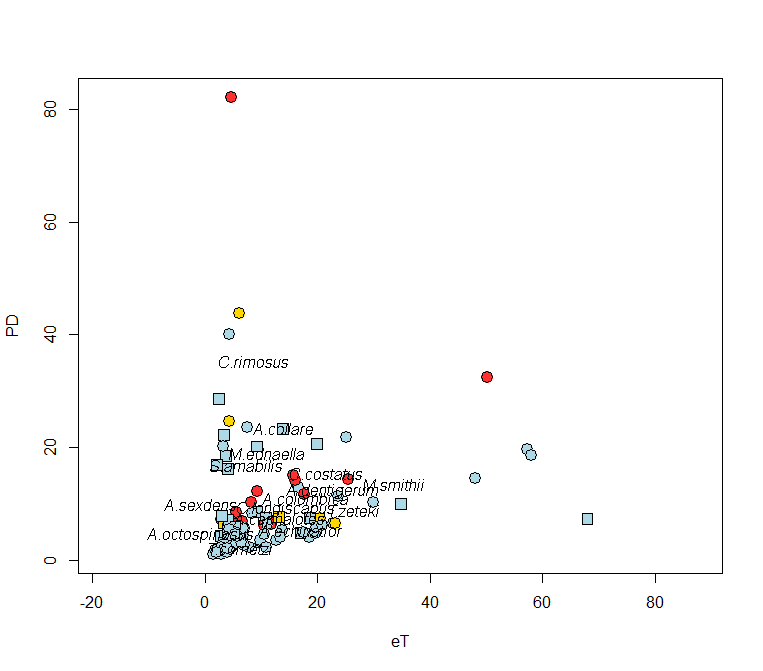


e^T^

**Figure SR1.9: Alpha diversity as estimated by Theil inequality (*e^T^*) and PD values for all 107 samples.** Color coding and symbols are the same as in Figure SR1.8. To examine the possible confounding effects of PCR method and the number of individual abdomens homogenized on these other two alpha diversity indices we used linear models in R, which showed that PCR method did not significantly affect these diversity estimates (F=0.01, d.f=1, p=0.920 and F=1.94, d.f.=1, p=0.166, for e^T^ and PD, respectively) and that the number of individual abdomens homogenized only had a significant effect on PD (F=5.46, d.f.=2, p=0.005) and not on e^T^ (F=0.14, d.f=2, p=0.864). However, in this analysis the effect of the ant species was significant regardless of the diversity index used (F=2.06, d.f=15, p=0.021 and F=2.07, d.f.=15, p=0.020 for e^T^ and PD, respectively).

Based on the combined validation checks that we performed, we concluded that our alpha diversity estimates remained liable based on possibly undetected contaminants, different numbers of homogenized abdomens of individual ants and PCR method, the latter of which was beyond our control. We therefore present our alpha diversity estimates only in the Supplementary Figures and Tables and refrain from making strong inferences on possible phylogenetic trends in alpha diversity across the attine ant phylogenetic tree.

***Possible confounding effects on estimates of beta diversity***


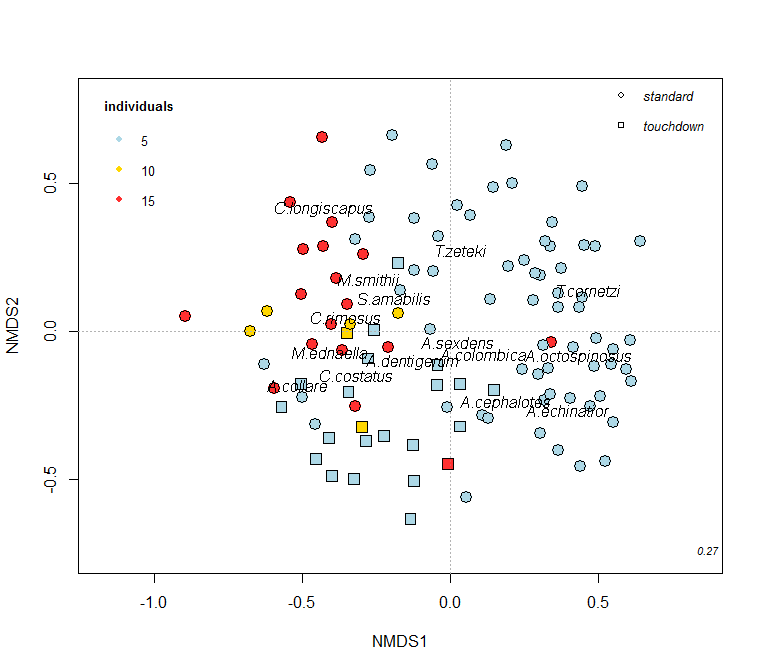
We also evaluated the extent to which our estimates of beta diversity might have been confounded by the same factors that we considered when validating the reliability of our alpha diversity estimates. For this analysis we used non-metric dimensional (NMDS) ordination of Bray-Curtis dissimilarity distances including all 107 samples used in our study encompassing 2099 OTUs after rarefication (Figure SR1.10). PERMANOVA tests showed that PCR method and number of individuals both had a significant effect on beta diversity (Pseudo-F=9.21, *R^2^*=0.048, p<0.001 and Pseudo-F=16.45, *R^2^*=0.087, p<0.001), but that the species (phylogeny) effect was at least five times more powerful in explaining the overall variability in the data (Pseudo-F=4.90, *R^2^*=0.391, p<0.001) in contrast to the alpha diversity analyses above where species effects were rather weak. We therefore decided to remove the samples whose libraries were generated with touchdown PCR from the beta diversity analyses, but to keep all 84 remaining samples because the species (phylogeny) effect appeared to be largely parallel to the weaker number of individuals effect.

**Figure SR1.10: Non-metric dimensional (NMDS) ordination of Bray-Curtis dissimilarity distances of all 107 samples using all 2099 OTUs after rarefication.** Samples are color-coded based on the number of individuals collected (top left), while the PCR method used to generate the libraries is highlighted with different symbols for each sample (top right). The NMDS stress factor is given at the bottom right.

**References**

Hu, Y., Holway, D. A., Łukasik, P., Chau, L., Kay, A. D., LeBrun, E. G., … Russell, J. A. (2017). By their own devices: invasive Argentine ants have shifted diet without clear aid from symbiotic microbes. *Molecular Ecology*, *26*(6), 1608–1630. doi:10.1111/mec.13991

Salter, S. J., Cox, M. J., Turek, E. M., Calus, S. T., Cookson, W. O., Moffatt, M. F., … Walker, A. W. (2014). Reagent and laboratory contamination can critically impact sequence-based microbiome analyses. *BMC Biology*, *12*, 87. doi:10.1186/s12915-014-0087-z

Sanders, J. G., Lukasik, P., Frederickson, M. E., Russell, J. A., Koga, R., Knight, R., & Pierce, N. E. (2017). Dramatic Differences in Gut Bacterial Densities Correlate with Diet and Habitat in Rainforest Ants. *Integrative and Comparative Biology*, *57*(4), 705–722. doi:10.1093/icb/icx088
