# Supplementary material for: The evolution of abdominal microbiomes in fungus‐growing ants
Source: Mol Ecol. 2018 Dec 10;28(4):879–99. doi: 10.1111/mec.14931 (PMC6446810; doi:10.1111/mec.14931)
Supplement: Supplementary file 2 [file MEC-28-879-s002.pdf]

Supplementary Results 2 – FISH Microscopy

Examples of bacteria detected in various abdominal tissues, gut lumen and fat bodies of attine ant workers using confocal laser scanning FISH microscopy. Staining was performed with 16S OTU-specific or genus-specific probes with either an attached alexa488 or a Cy3 fluorochrome, which appears as bright green spots when present) or a Cy5 flurochrome (which appears as bright red spots when present. DAPI staining of bacterial DNA was used as a positive control in each experiment (appears as bright blue spots). All channels (DAPI:blue, 16S_alexa488/Cy3:green, 16S_Cy5:red) were always analyzed separately. Frame triplets show examples of typical regions of interest (ROIs) for specific bacterial signals in black and white to maximize contrast. The order of the different channels is always the same: top (blue) DAPI, middle (green) alexa488, bottom (red) Cy5; all at 3× background magnification. We frequently detected bacterial cells in the green channel within which autofluorescence is more frequent. This urged us to be as critical as possible; we only accepted a signal as positive evidence if it had a clearly defined, congruent shape in i) both the blue and green channel but not in the red channel, ii) both the blue and the red channel (but not in the green), and iii) only in the blue channel. Cases in which a signal was present in all three different channels were rare and always dismissed as being due to autofluorescence since we never used two 16S probes targeting the same OTU or class (see also Methods). After applying these criteria, our microscopy observations suggested that the most widespread bacterial OTUs (among the ones we examined) were those belonging to the order of Entomoplasmatales and that their cells are likely scattered across multiple ant tissues.

For each example presented in this file the title shows the ant species and the tissue examined, after which the original image with ROIs and scale bar follows with the bottom image showing how we further evaluated the specificity of our probes by applying a granulometric filtering and only accepting signals that were circular and had a radius of ca. 1.5 – 2.5 µm as expected for bacterial cells. Each example also has a short legend providing details about the staining. All scale bars are always 20um, even if they may seem different because some pictures are zoomed or cropped versions while others are not.

At. colombica – Malpighian Tubules

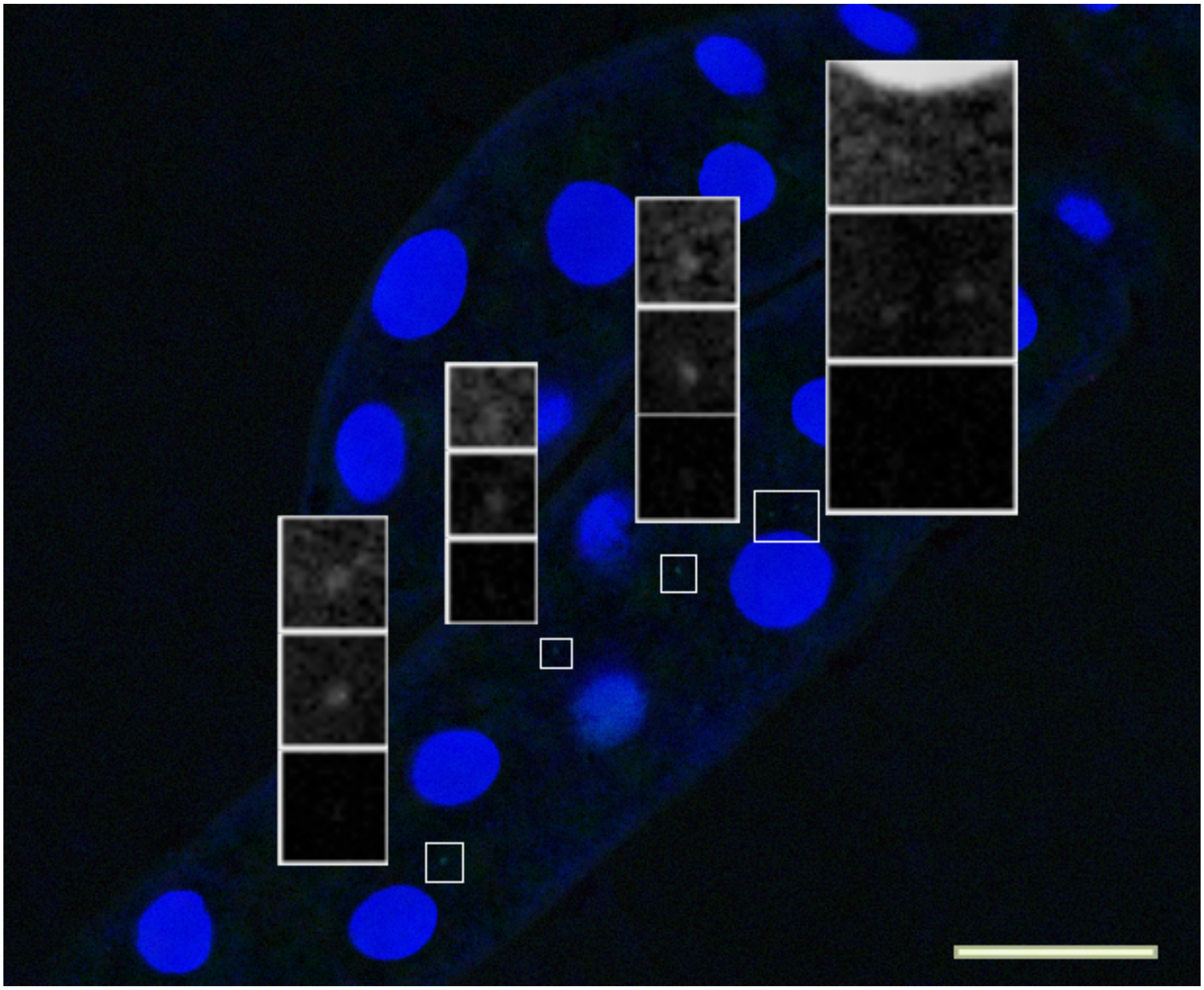

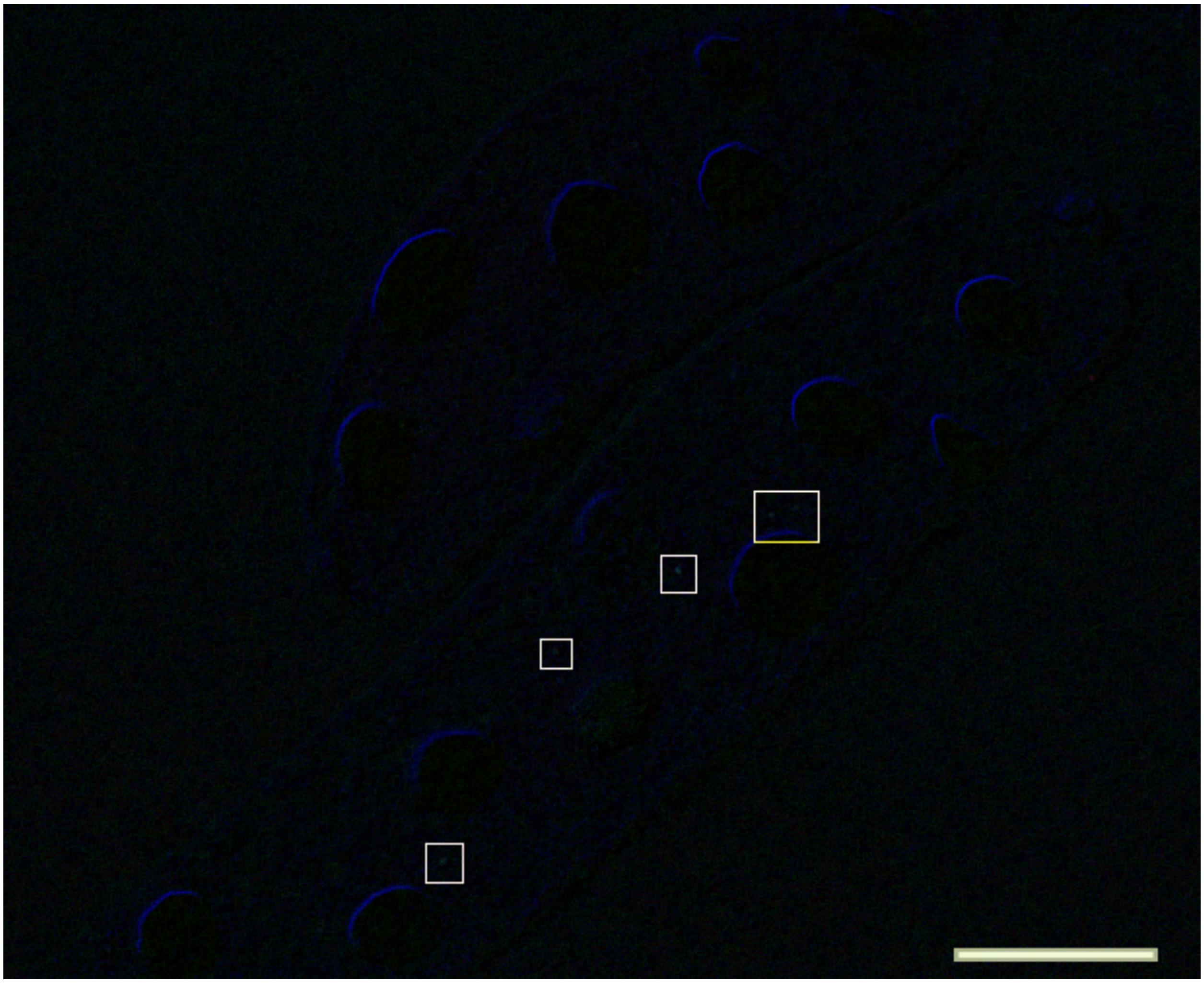

Figure SR2.1: Examples of Mollicutes bacteria detected in Malpighian tubules of *Atta colombica* workers. Staining was performed with a 16S Mollicutes-specific probe (Entom_A488; Table S2) with an attached alexa488 fluorochrome, which appears as bright green spots when present. DAPI staining appears as bright blue spots. The red channel shows an image taken at a wavelength >630nm where we would normally detect probes with an attached Cy5 fluorochrome but here they functioned as autofluorescence controls because we did not use any Cy5 probes. FISH images suggested that Mollicutes bacteria are abundantly present.

At. colombica – hindgut

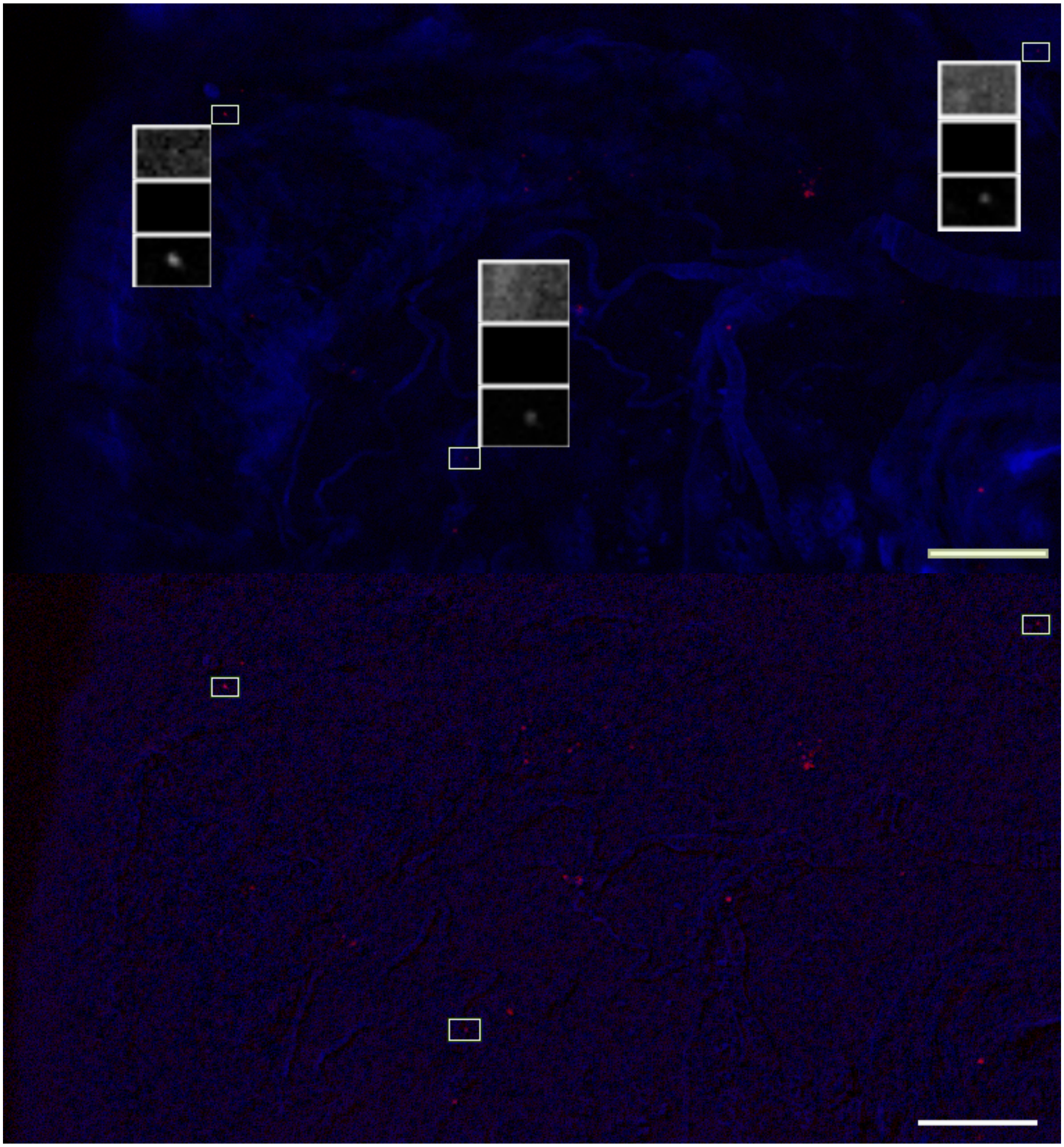

Figure SR2.2: Examples of Gamma-Proteobacteria detected in the rectum of *Atta colombica* workers. Staining was performed with a Gamma-Proteobacteria-specific probe (Xantho2; Table S2) with an attached Cy5 fluorochrome, which appears as red/green spots when present. DAPI staining of bacterial DNA appears as bright blue spots. The green channel shows an image taken at wavelength 400-500nm where we would normally detect probes with an attached alexa488 fluorochrome but here they functioned as autofluorescence controls because we did not use any probes that can be detected in that wavelength. FISH images suggested that Gamma-Proteobacteria are sparsely present.

At. cephalotes – fat bodies

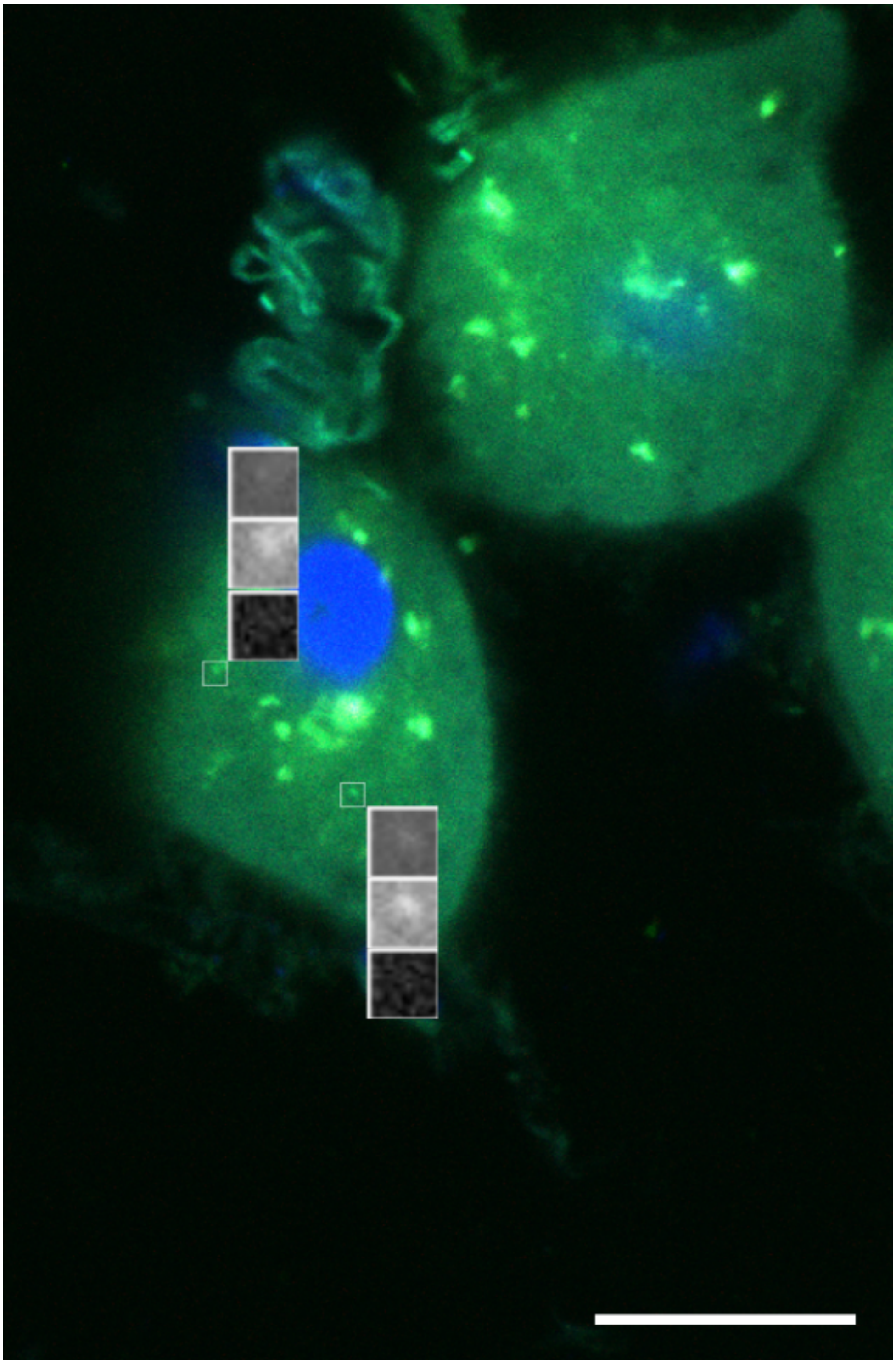

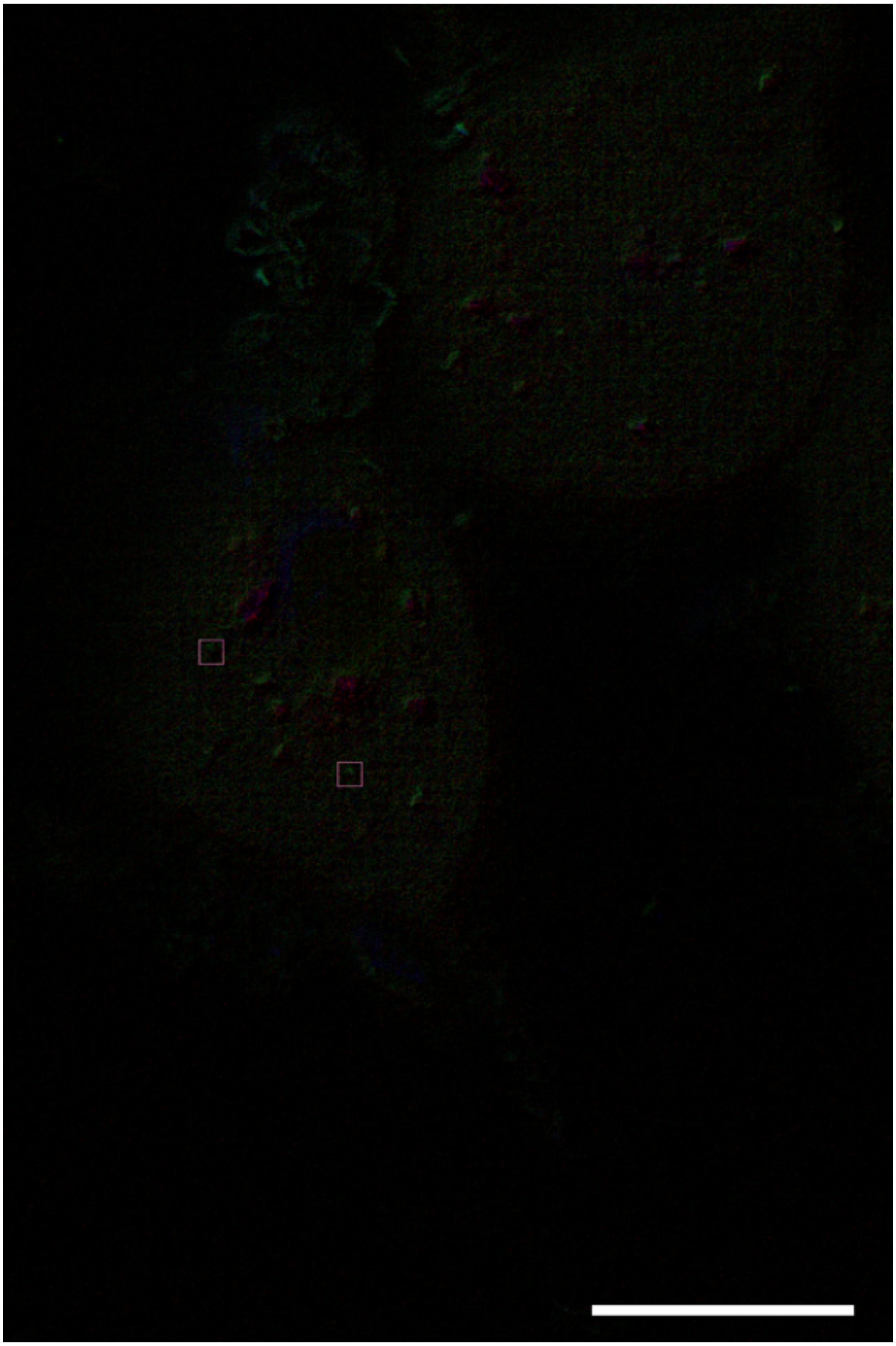

Figure SR2.3: Examples of Mollicutes bacteria detected in fat body cells of *Atta colombica* workers. Staining was performed with a 16S Mollicutes-specific probe (Entom_A488; Table S2) with an attached alexa488 fluorochrome, which appears as bright green spots when present. DAPI staining appears as bright blue spots. The red channel shows an image taken at a wavelength >630nm where we would normally detect probes with an attached Cy5 fluorochrome but here they functioned as autofluorescence controls because we did not use any Cy5 probes. FISH images suggested that Mollicutes bacteria are abundantly present.

At. colombica – hindgut (negative)

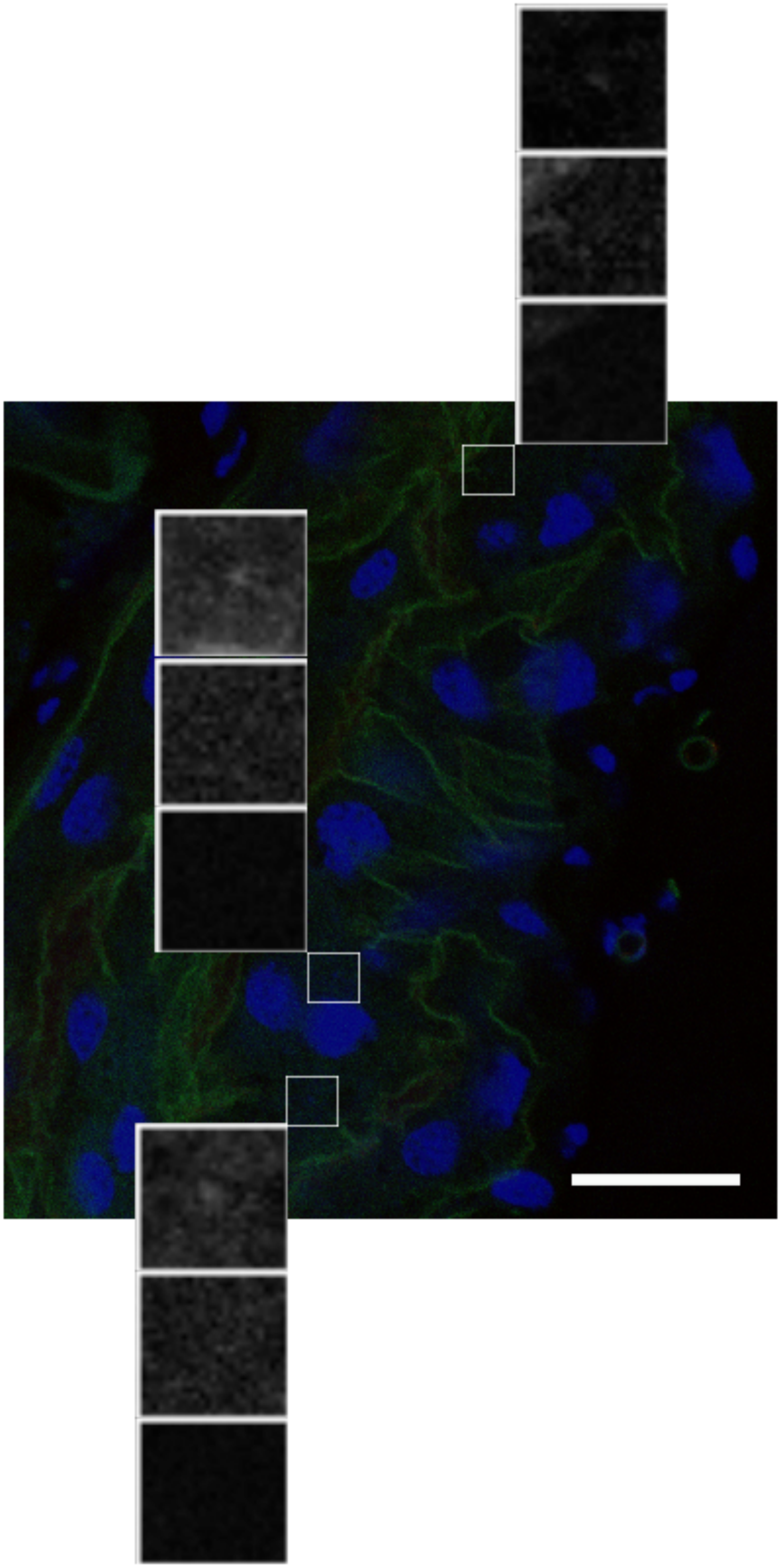

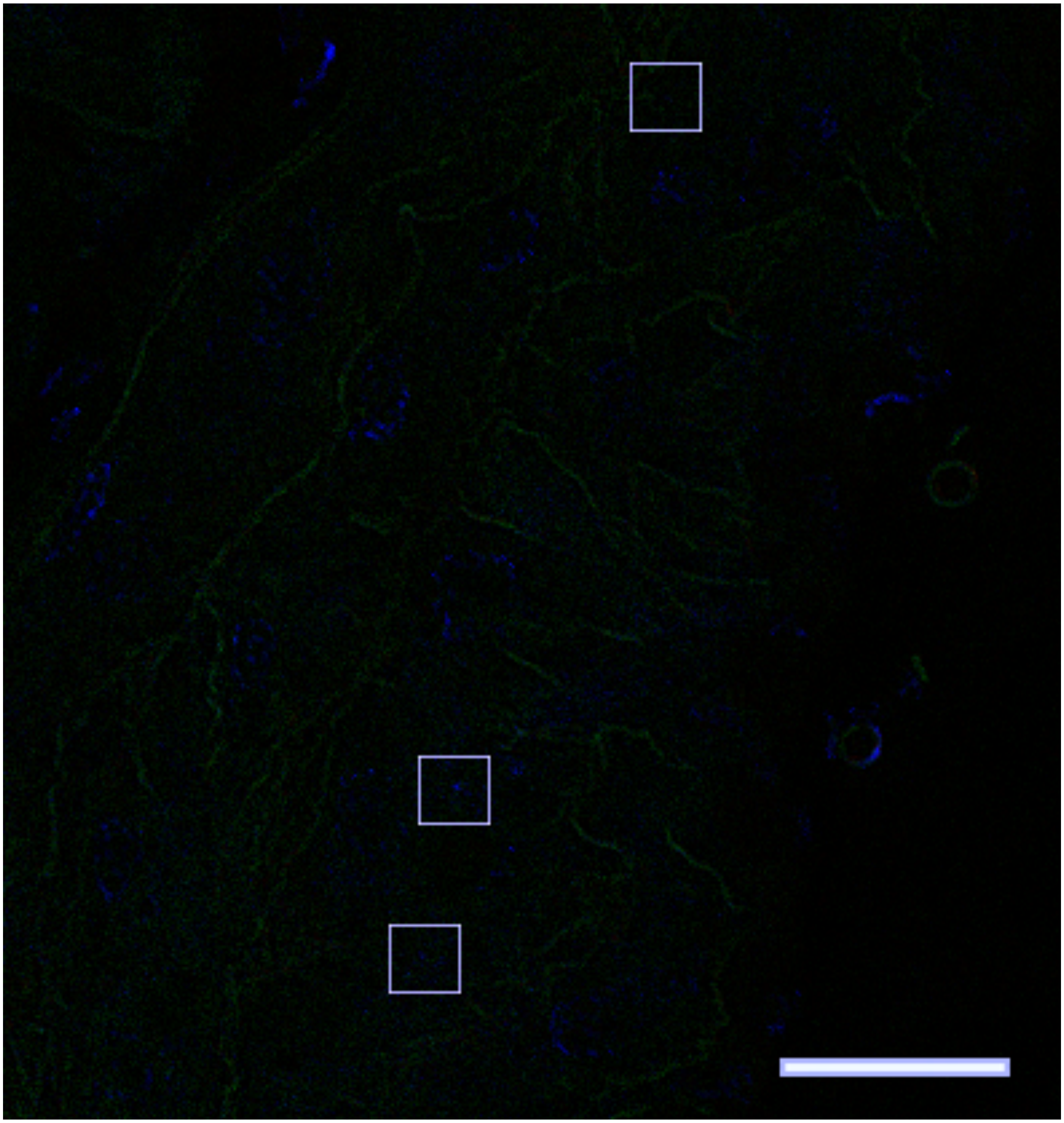

Figure SR2.4: Examples showing the specificity of our FISH protocol by staining with antisense probes, i.e. reversed sequences which have no sequence matches and therefore no probe-specific bacteria detection in the ileum of *Atta colombica* workers was expected. Staining was performed with a 16S anti-sense Mollicutes-specific probe with an attached alexa488 fluorochrome, which would have appeared as bright green spots if there was staining and a 16S anti-sense Alpha-Proteobacteria probe with an attached Cy5 fluorochrome, which would have appeared as bright red spots if there was staining. DAPI staining of bacterial DNA was used as a positive control and appears as bright blue spots. Only the DAPI signal could be detected, which shows that bacteria are present but that the anti-sense probes were not hybridizing to them (as expected). Probe sequences are shown in Table S2.

***At. colombica* – hindgut (negative)**

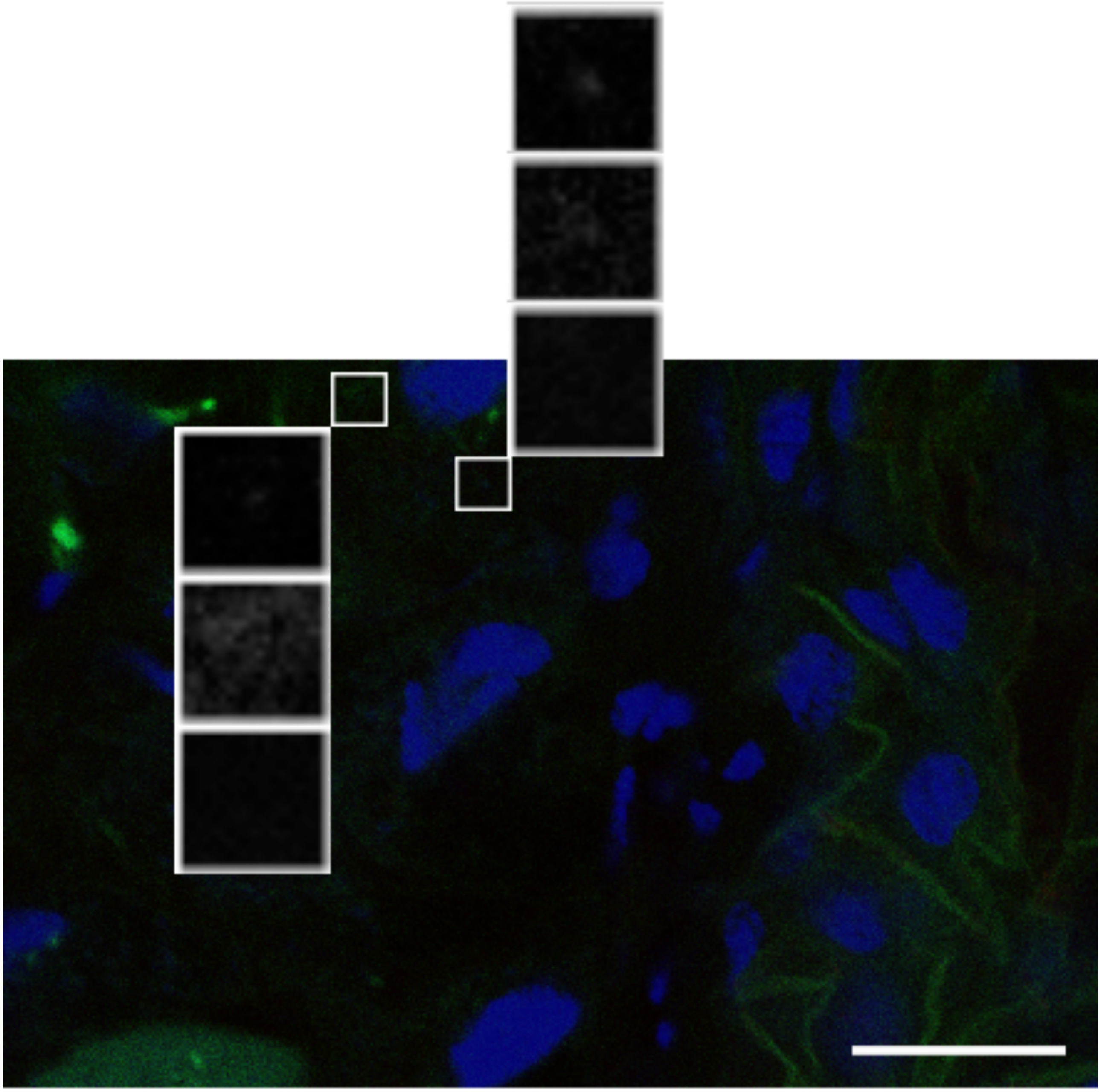

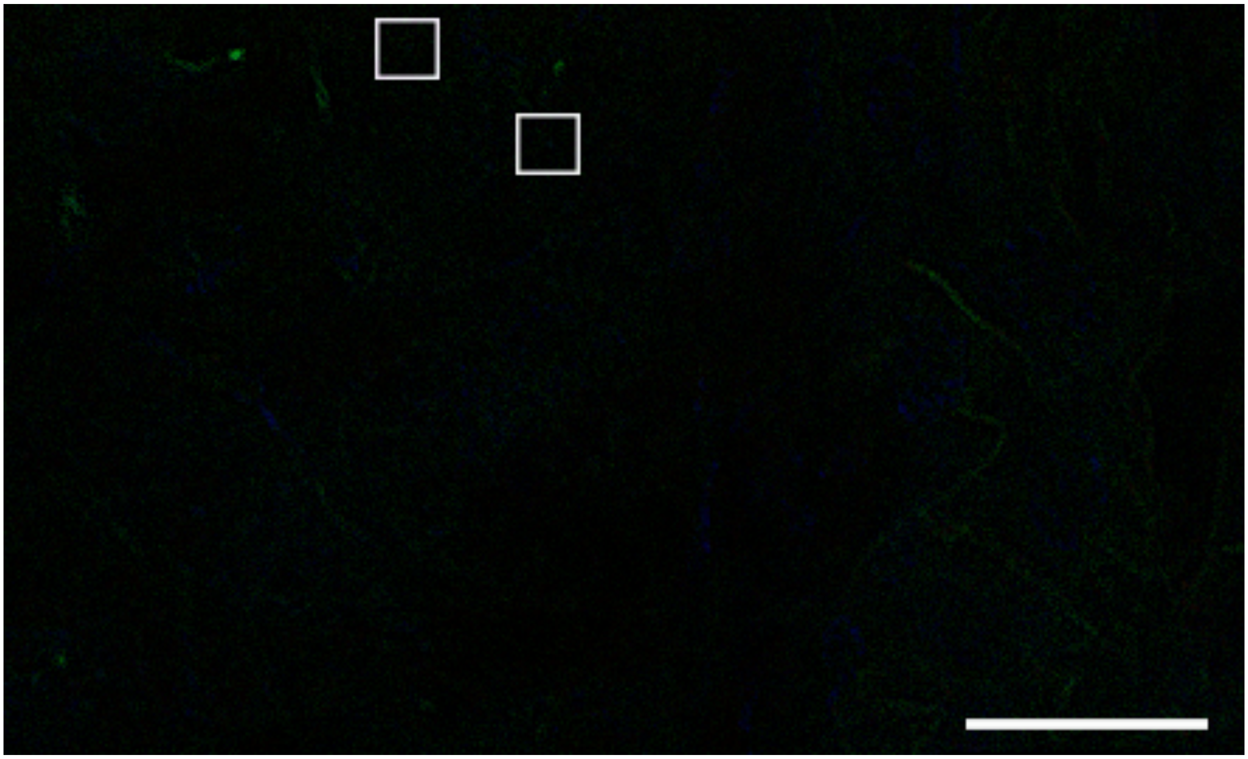

Figure SR2.5: Examples showing the specificity of our FISH protocol by staining with antisense probes, i.e. reversed sequences which have no sequence matches and therefore there was no probe-specific bacteria detected in the hindgut of *Atta colombica* workers. Staining was performed with a 16S anti-sense Mollicutes-specific probe with an attached alexa488 fluorochrome, which would have appeared as bright green spots if there was staining and a 16S anti-sense Alpha-Proteobacteria probe with an attached Cy5 fluorochrome, which would have appeared as bright red spots if there was staining. DAPI staining of bacterial DNA was used as a positive control and appears as bright blue spots. Only the DAPI signal could be detected which shows that bacteria were present but the anti-sense probes were not hybridizing to them (as expected). Probe sequences are shown in Table S2.

T. cornetzi – fat body

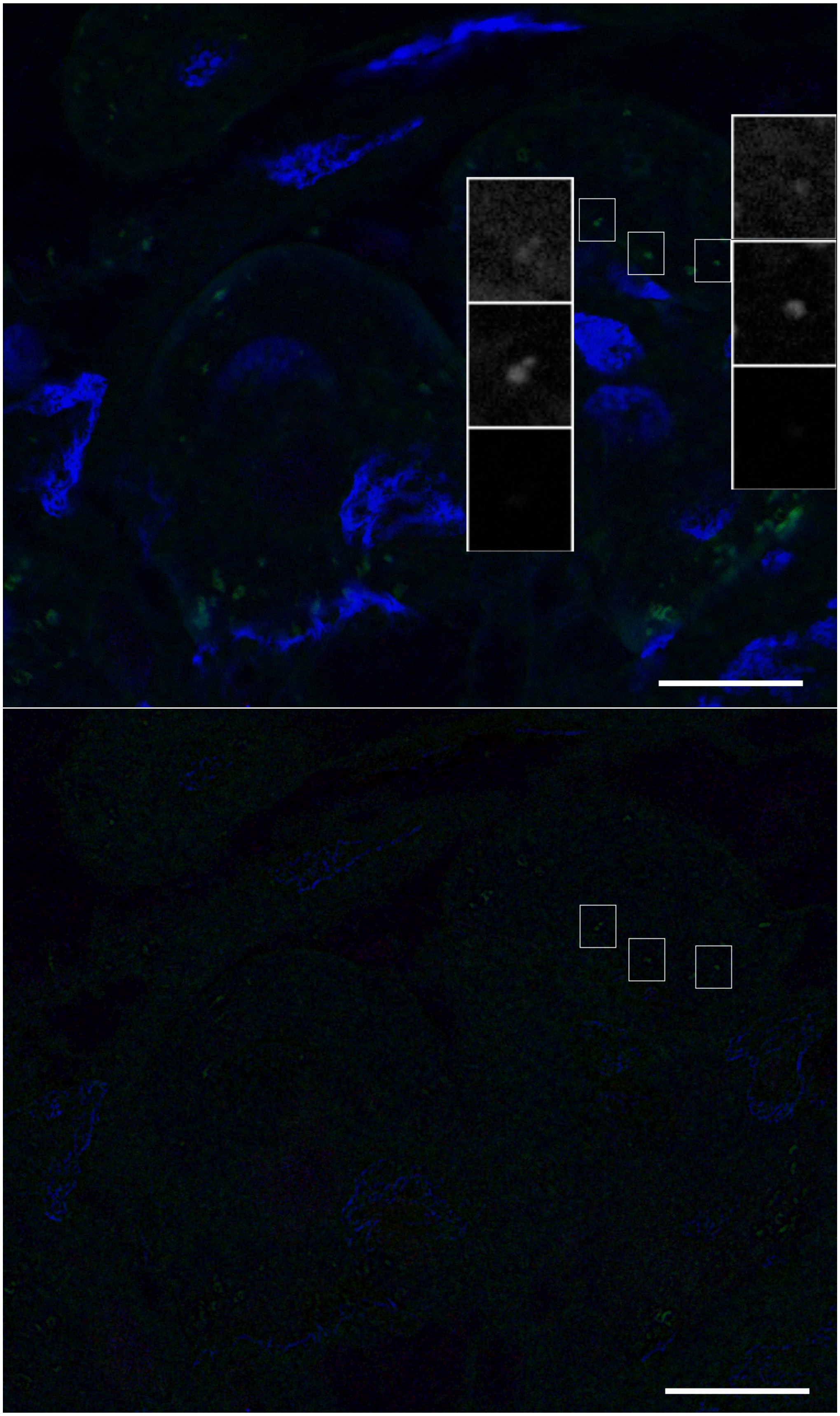

Figure SR2.6: Examples of Mollicutes bacteria detected in fat body cells of *T. cornetzi* workers. Staining was performed with a 16S Mollicutes-specific probe (Entom_A488; Table S2) with an attached alexa488 fluorochrome, which appears as bright green spots when present and a *RhiAcro1* specific probe (Phyllo_Cy5; Table S2) with an attached Cy5 fluorochrome, which would appear as bright red spots but it did not show up here because there are no *RhiAcro1* bacteria in the fat body cells. DAPI staining appears as bright blue spots. Each of the probe-specific channels (green and red) functions also as autofluorescence control for the other one, because both probes were specific to different OTUs and therefore there were no bacteria in common that they could both detect. FISH images suggested that Mollicutes bacteria are sparsely present and *RhiAcro1* bacteria absent.

T. cornetzi – ileum

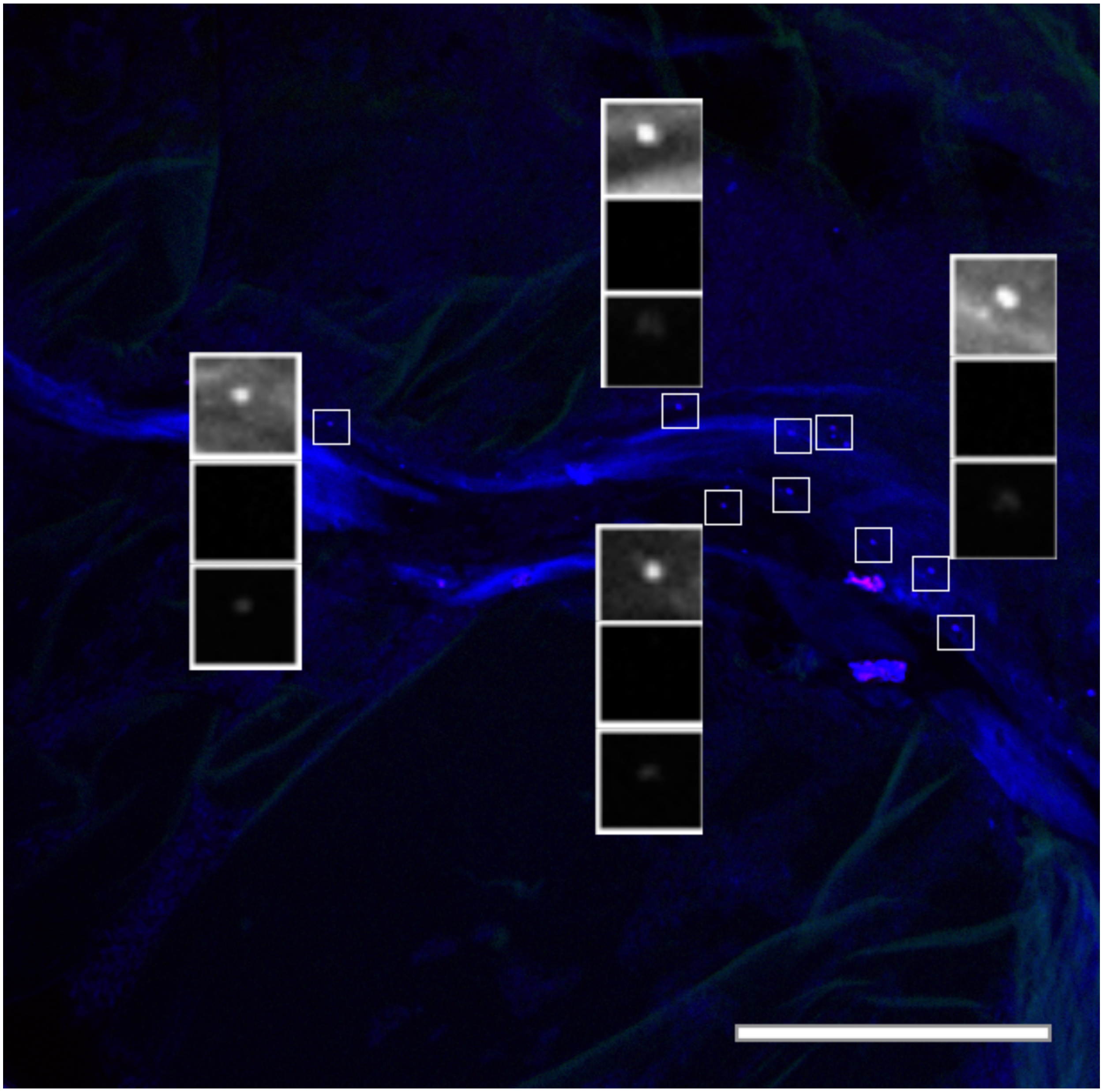

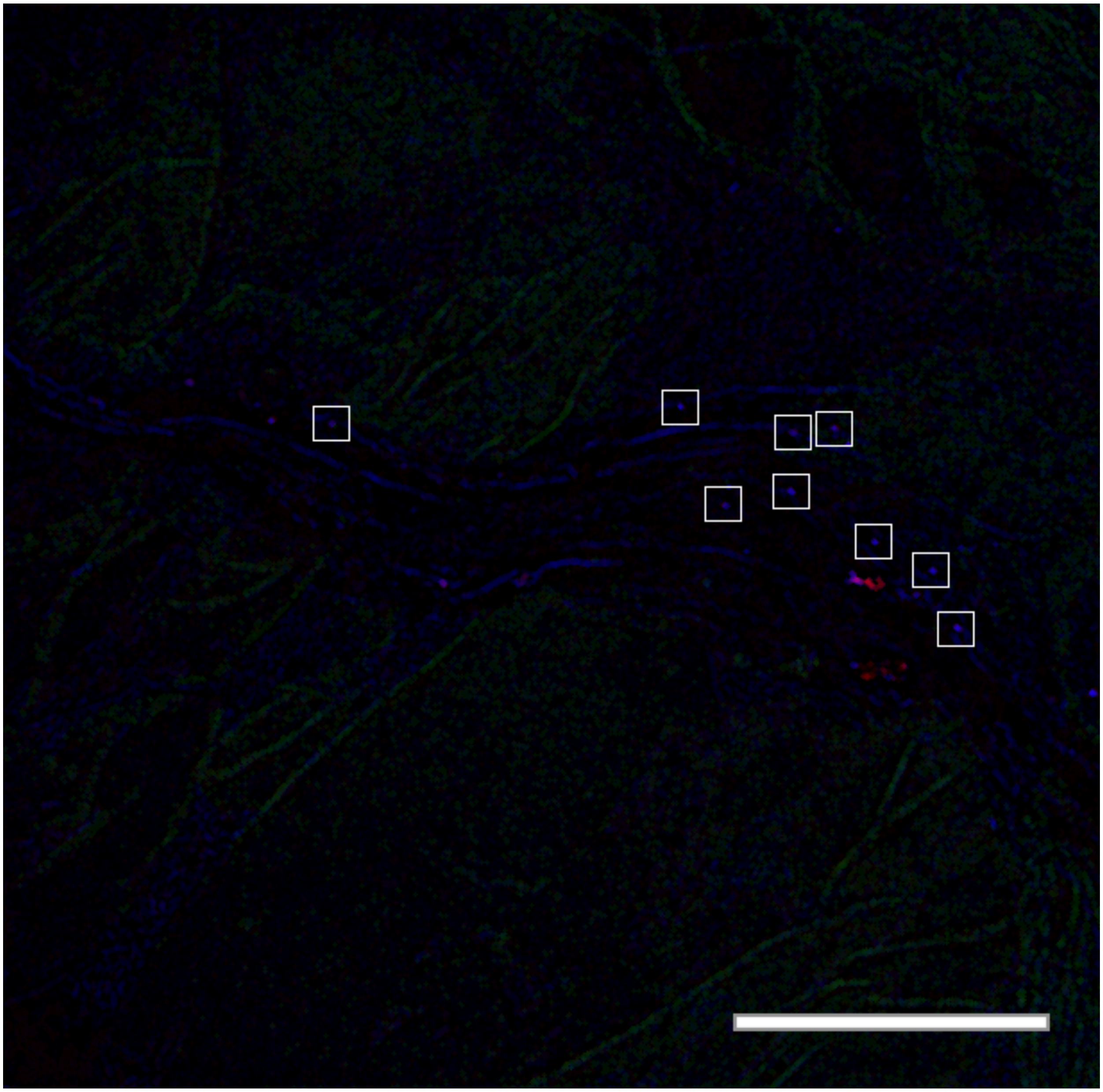

Figure SR2.7: Examples of *RhiAcro1* bacteria detected in the ileum of *T. cornetzi* workers. Staining was performed with a 16S Mollicutes-specific probe (Entom_A488; Table S2) with an attached alexa488 fluorochrome, which would appear as bright green spots if present and a *RhiAcro1* specific probe (Phyllo_Cy5; Table S2) with an attached Cy5 fluorochrome, which appears as bright red spots when present. DAPI staining appears as bright blue spots. Each of the probe-specific channels (green and red) functions also as autofluorescence control for the other one, because both probes were specific to different OTUs and therefore there were no bacteria in common that they could both detect. FISH images suggested that *RhiAcro1* bacteria are abundantly present and Mollicutes bacteria absent.

T. cornetzi – ileum (negative)

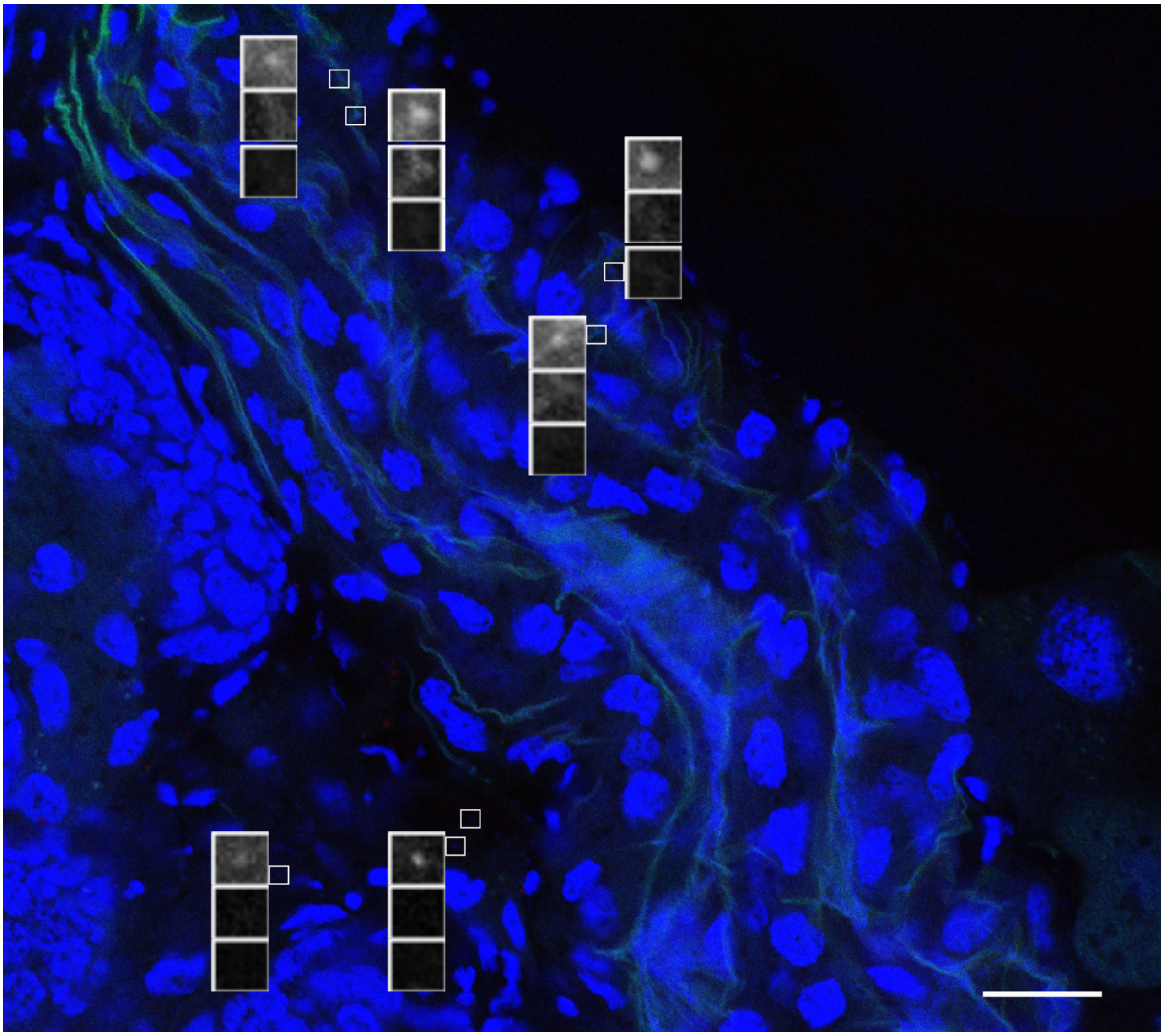

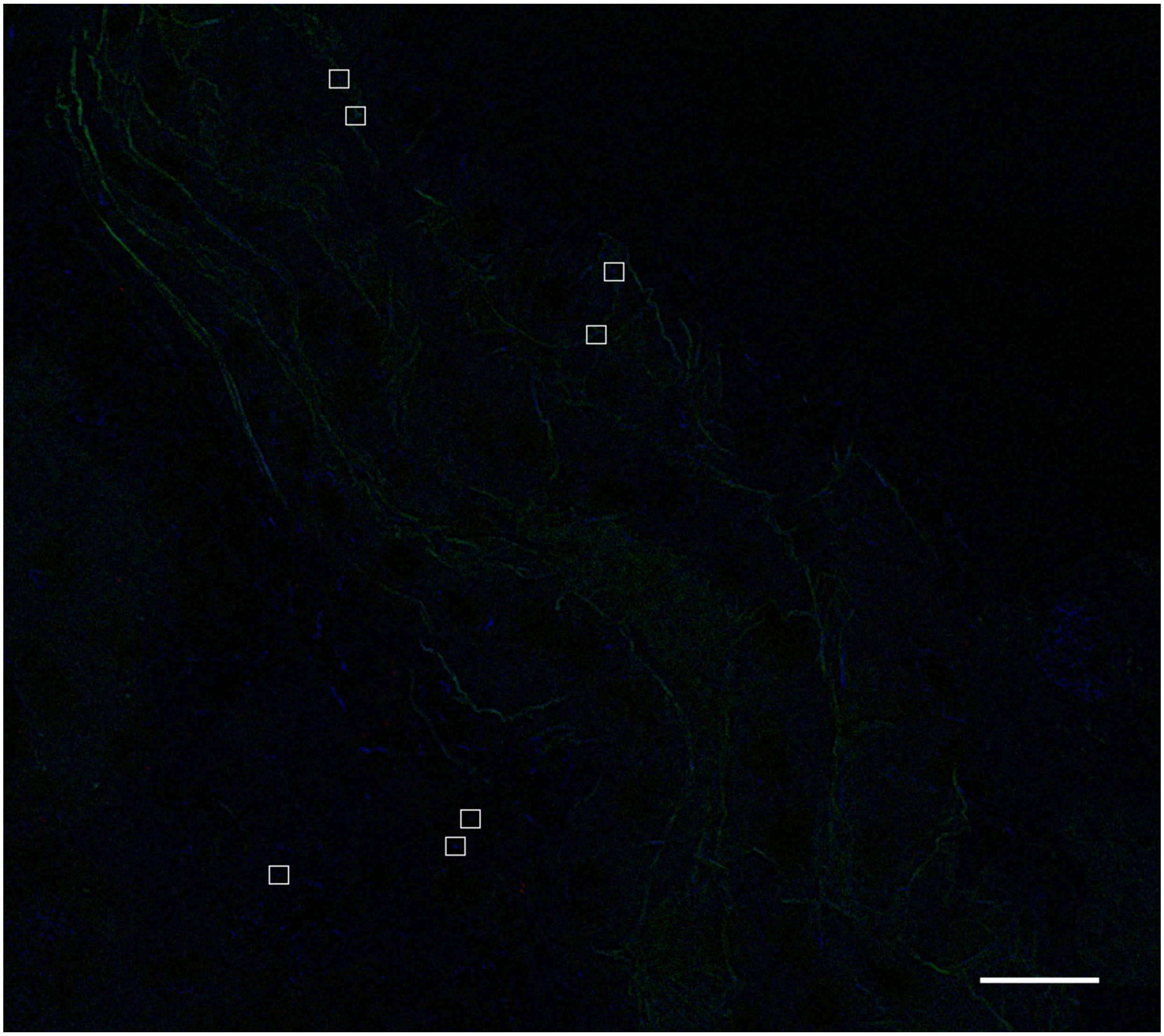

Figure SR2.8: Examples showing the specificity of our FISH protocol by performing staining with antisense probes (reversed sequences which have no sequence matches) and therefore there was no probe-specific bacteria detected in the ileum of *T. cornetzi* workers. Staining was performed with a 16S anti-sense Mollicutes-specific probe with an attached alexa488 fluorochrome, which would have appeared as bright green spots if there was staining and a 16S anti-sense Alpha-Proteobacteria probe with an attached Cy5 fluorochrome, which would have appeared as bright red spots if there was staining. DAPI staining of bacterial DNA was used as a positive control and appears as bright blue spots. Only the DAPI signal could be detected which shows that bacteria are present but the anti-sense probes are not hybridizing to them (as expected). Probe sequences are shown in Table S2.

S. amabilis – fat body cell

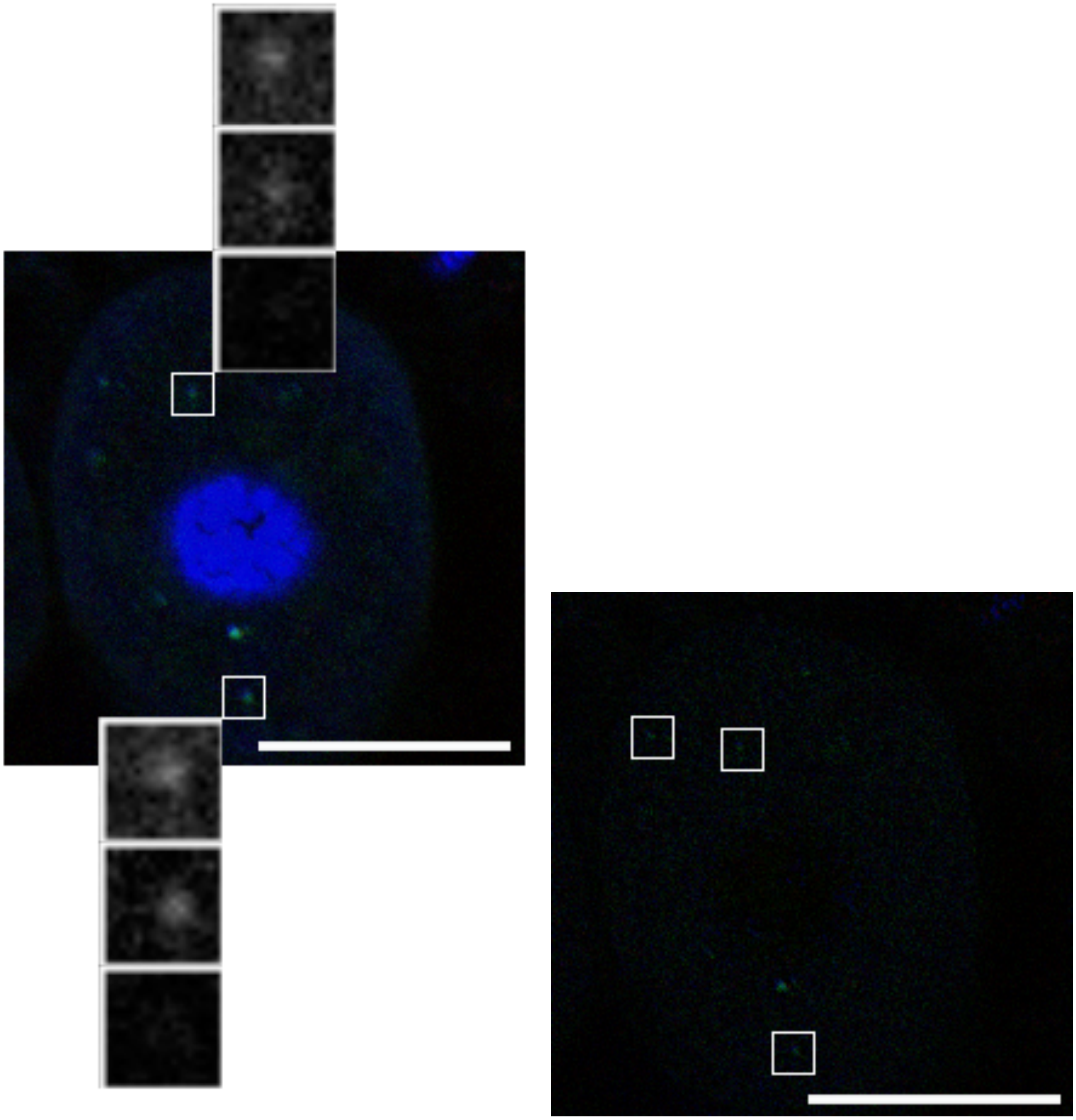

Figure SR2.9: Examples of Mollicutes bacteria detected in fat body cells of *S. amabilis* workers. Staining was performed with a 16S Mollicutes-specific probe (Entom_A488; Table S2) with an attached alexa488 fluorochrome, which appears as bright green spots when present and a *RhiAcro1* specific probe (Phyllo_Cy5; Table S2) with an attached Cy5 fluorochrome, which would appear as bright red spots but it does not show up here because there are no *RhiAcro1* bacteria in the fat body cells. DAPI staining appears as bright blue spots. Each of the probe-specific channels (green and red) works also as autofluorescence control for the other one, because both probes used were specific to different OTUs and therefore there were no bacteria in common that they could both detect. FISH images suggested that Mollicutes bacteria are abundantly present and *RhiAcro1* bacteria absent.

S. amabilis – ileum

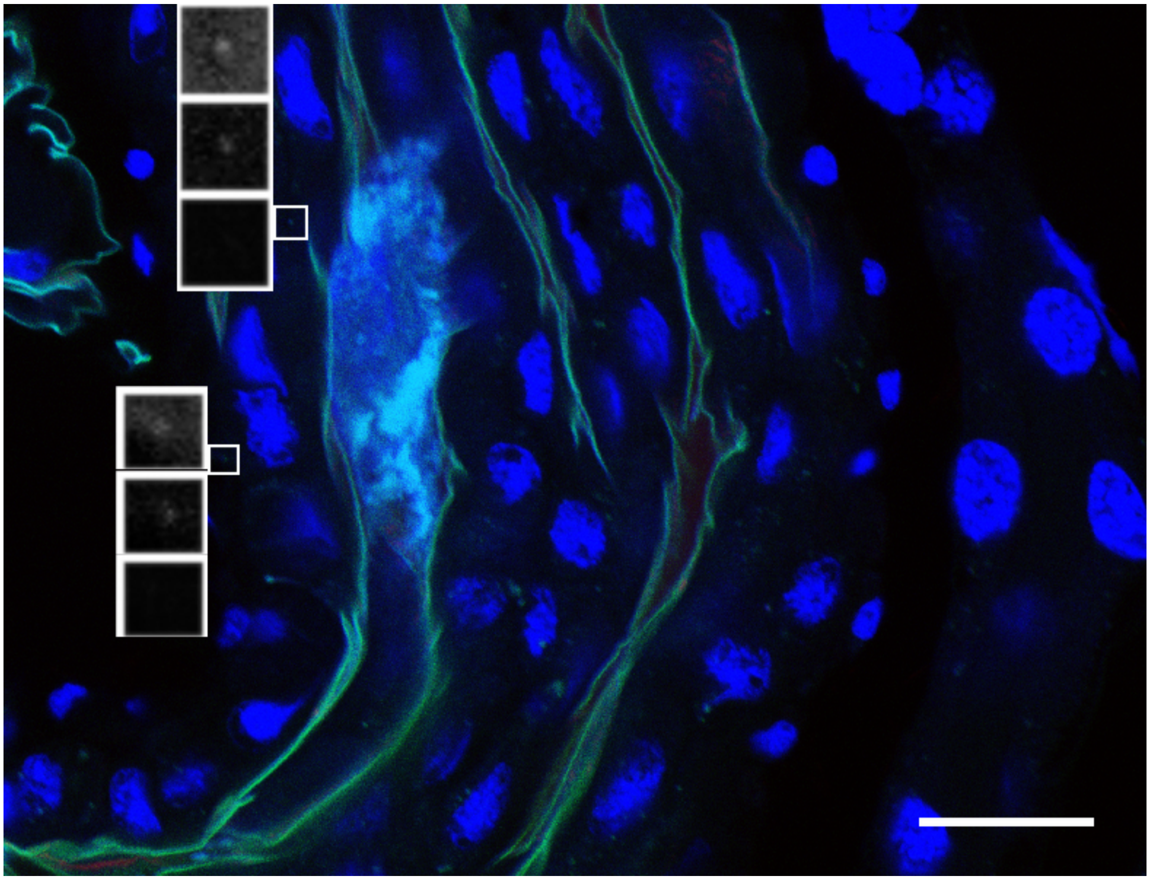

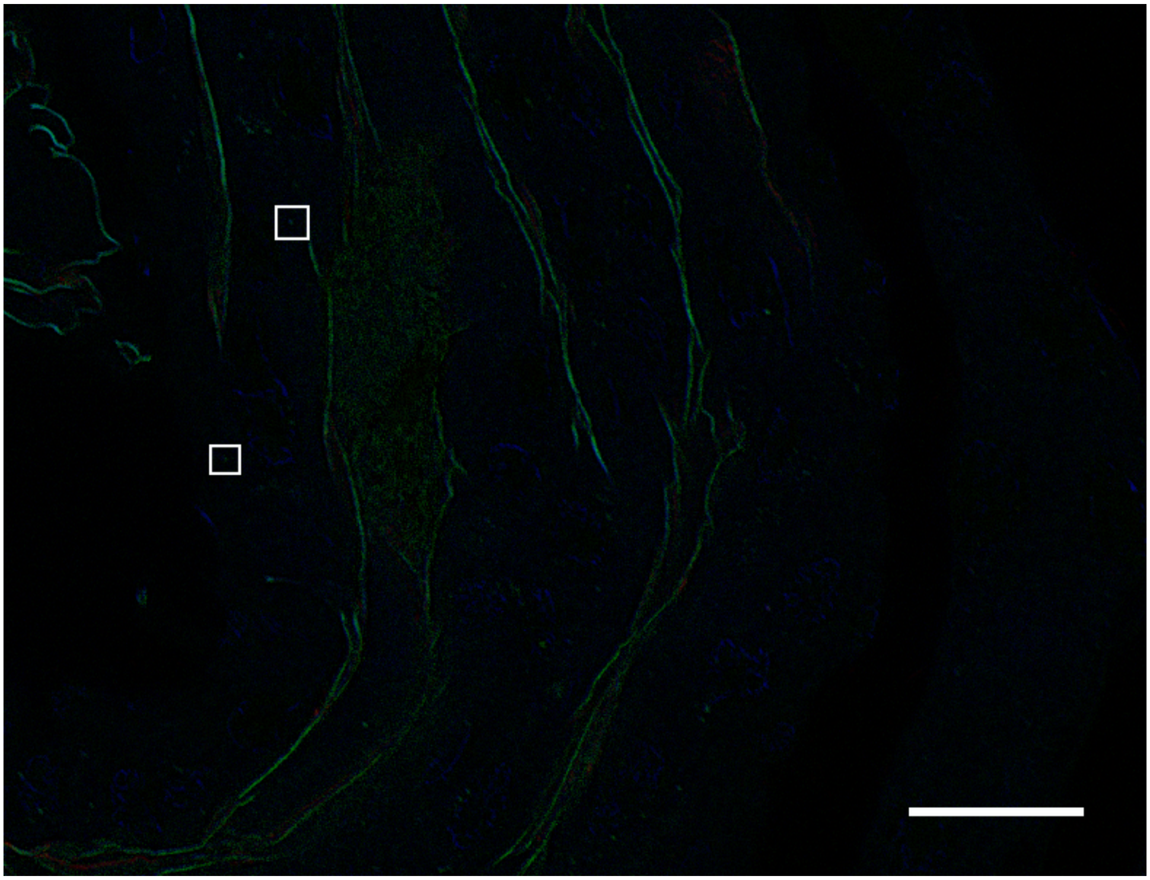

Figure SR2.10: Examples of Mollicutes bacteria detected in the ileum of *S. amabilis* workers. Staining was performed with a 16S Mollicutes-specific probe (Entom_A488; Table S2) with an attached alexa488 fluorochrome, which appears as bright green spots when present) and a *RhiAcro1* specific probe (Phyllo_Cy5; Table S2) with an attached Cy5 fluorochrome, which would appear as bright red spots but it does not show up here because there are no *RhiAcro1* bacteria present in this tissue. DAPI staining appears as bright blue spots. Each of the probe-specific channels (green and red) works also as autofluorescence control for the other one, because both probes used were specific to different OTUs and therefore there were no bacteria in common that they could both detect. FISH images suggested that Mollicutes bacteria are abundantly present and *RhiAcro1* bacteria absent.

S. amabilis – midgut

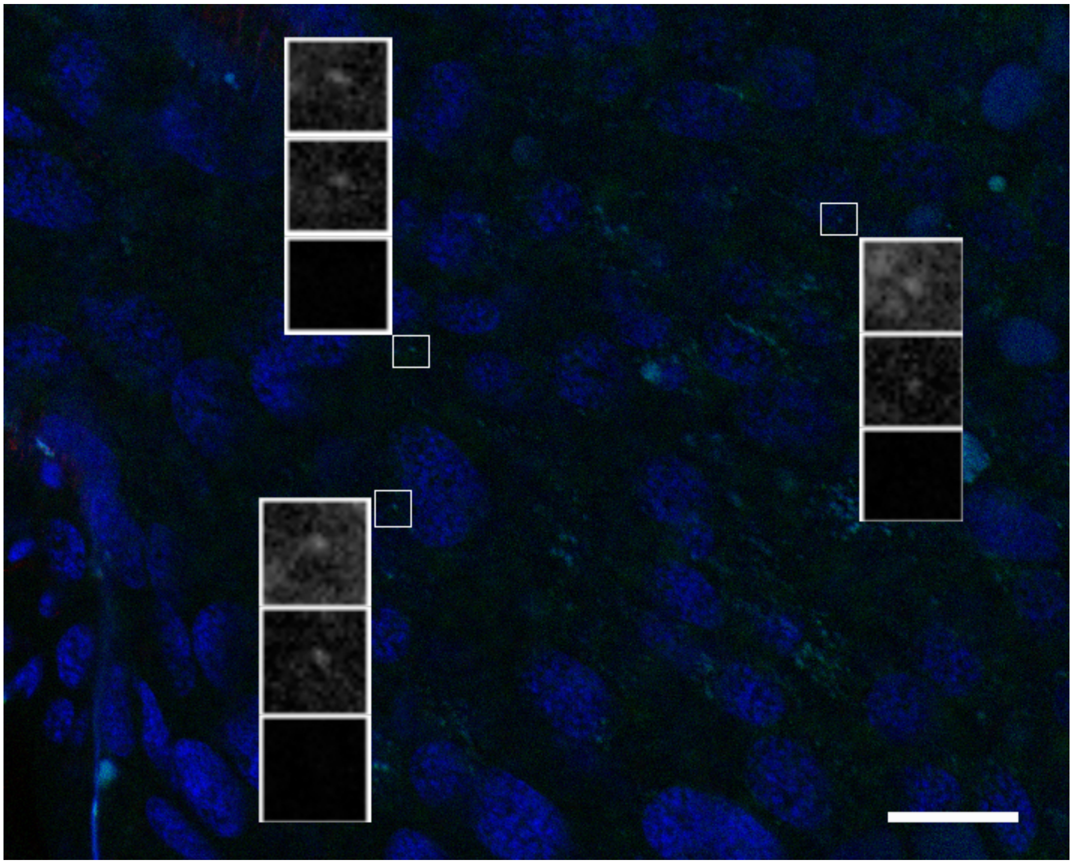

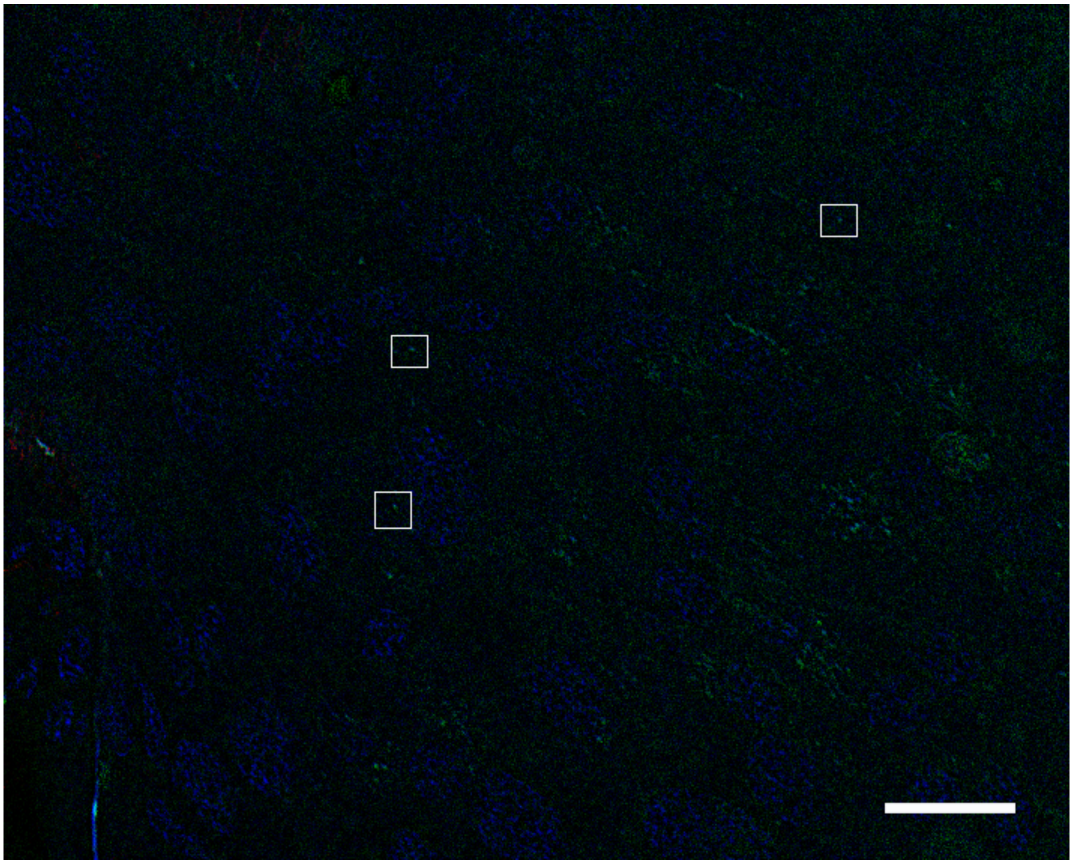

Figure SR2.11: Examples of Mollicutes bacteria detected in the midgut of *S. amabilis* workers. Staining was performed with a 16S Mollicutes-specific probe (Entom_A488; Table S2) with an attached alexa488 fluorochrome, which appears as bright green spots when present and a *RhiAcro1* specific probe (Phyllo_Cy5; Table S2) with an attached Cy5 fluorochrome, which would appear as bright red spots but it does not show up here because there are no *RhiAcro1* bacteria present in this tissue. DAPI staining appears as bright blue spots. Each of the probe-specific channels (green and red) works also as autofluorescence control for the other one, because both probes used were specific to different OTUs and therefore there were no bacteria in common that they could both detect. FISH images suggested that Mollicutes bacteria are abundantly present and *RhiAcro1* bacteria absent.

S. amabilis – ileum (negative)

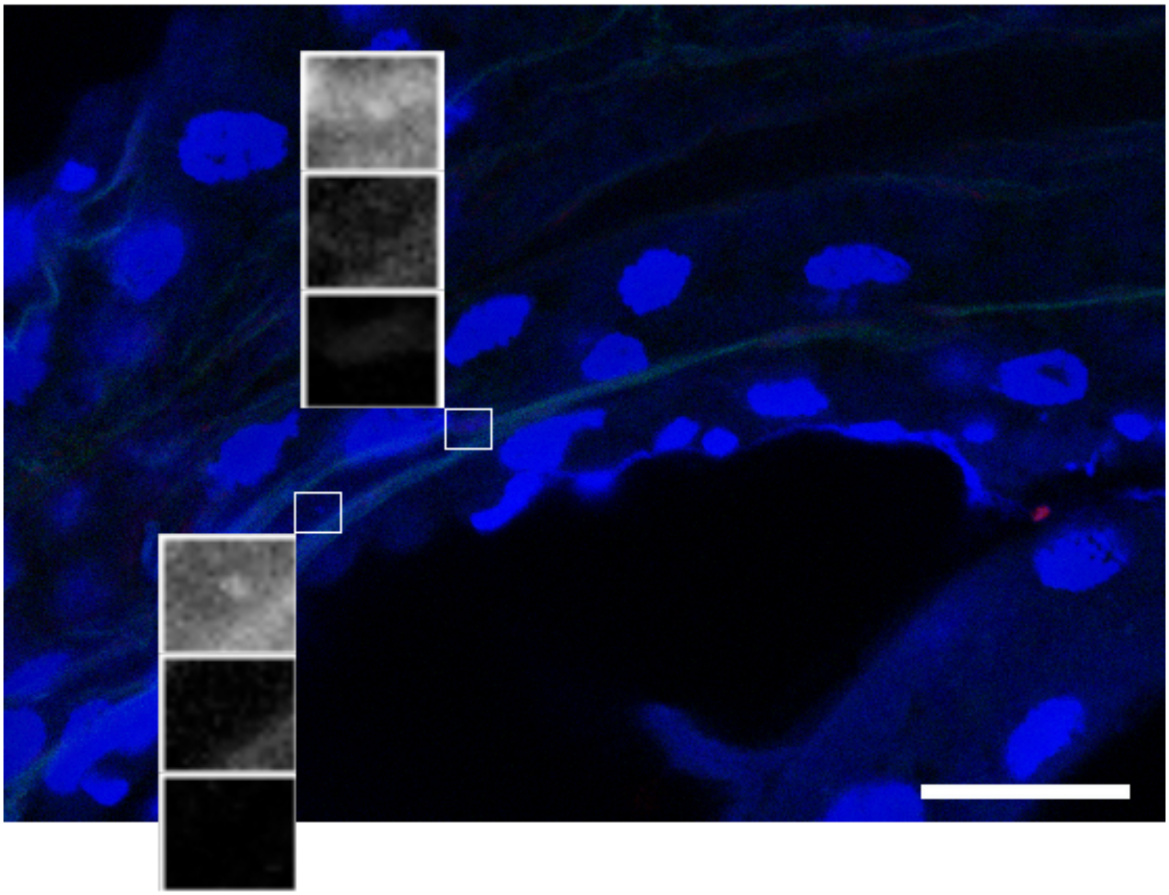

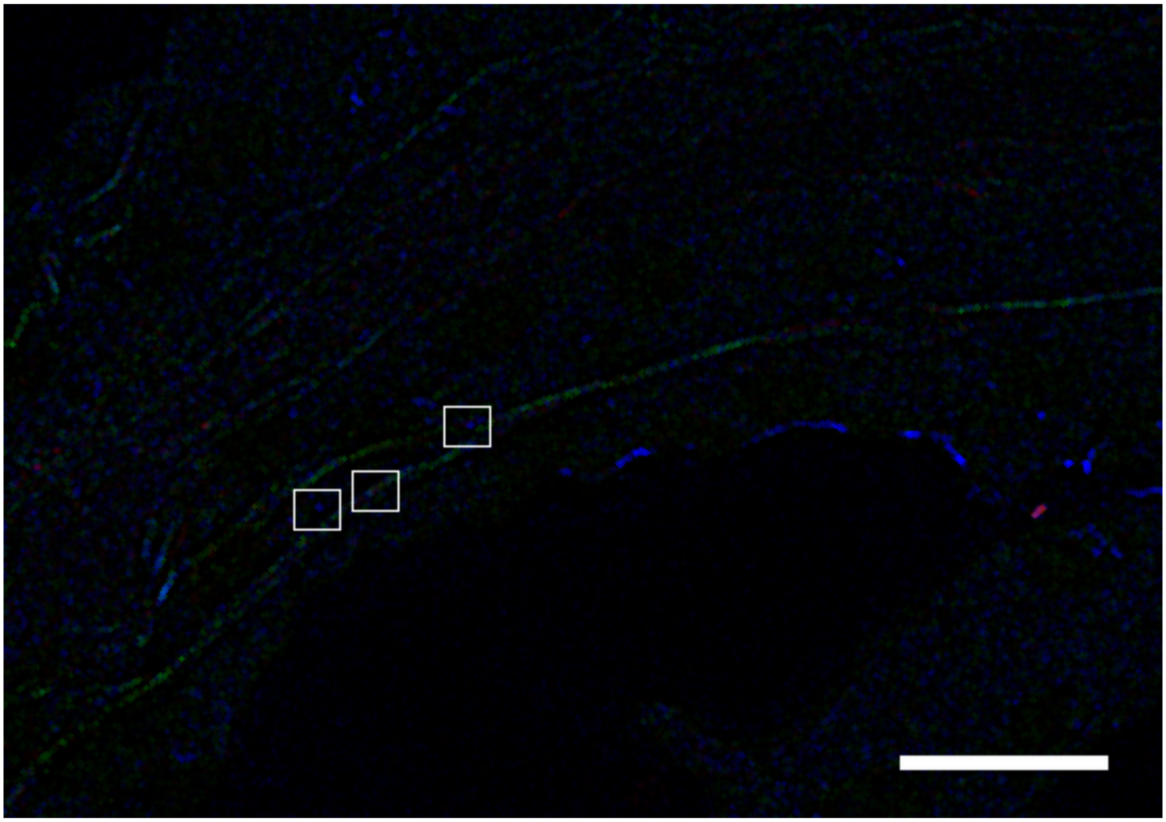

Figure SR2.12: Examples showing the specificity of our FISH protocol by performing staining with antisense probes, i.e. reversed sequences which have no sequence matches and therefore there was no probe-specific bacteria detected in the ileum of *S. amabilis* workers. Staining was performed with a 16S anti-sense Mollicutes-specific probe with an attached alexa488 fluorochrome, which would have appeared as bright green spots if there was staining and a 16S anti-sense Alpha-Proteobacteria probe with an attached Cy5 fluorochrome, which would have appeared as bright red spots if there was staining. DAPI staining of bacterial DNA was used as a positive control and appears as bright blue spots. Only the DAPI signal could be detected which shows that bacteria are present but the anti-sense probes are not hybridizing to them (as expected). Probe sequences are shown in Table S2.

T. zeteki – fat body

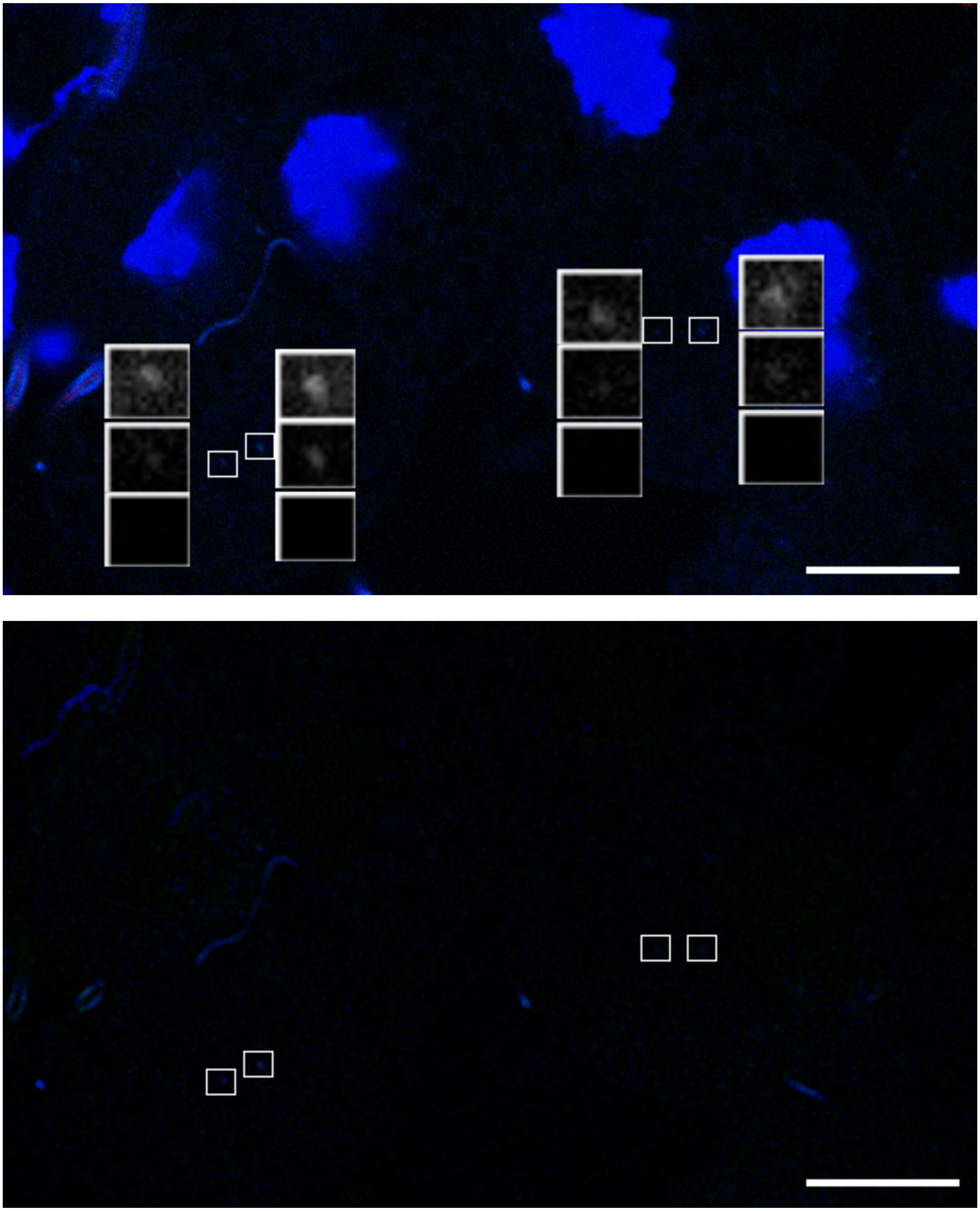

Figure SR2.13: Examples of Mollicutes bacteria detected in fat body cells of *T. zeteki* workers. Staining was performed with a 16S Mollicutes-specific probe (Entom_A488; Table S2) with an attached alexa488 fluorochrome, which appears as bright green spots when present and a *RhiAcro1* specific probe (Phyllo_Cy5; Table S2) with an attached Cy5 fluorochrome, which would appear as bright red spots but it does not show up here because there are no *RhiAcro1* bacteria in the fat body cells. DAPI staining

appears as bright blue spots. Each of the probe-specific channels (green and red) works also as autofluorescence control for the other one, because both probes used were specific to different OTUs and therefore there were no bacteria in common that they could both detect. FISH images suggested that Mollicutes bacteria are abundantly present and *RhiAcro1* bacteria absent.

T. zeteki – ileum (negative)

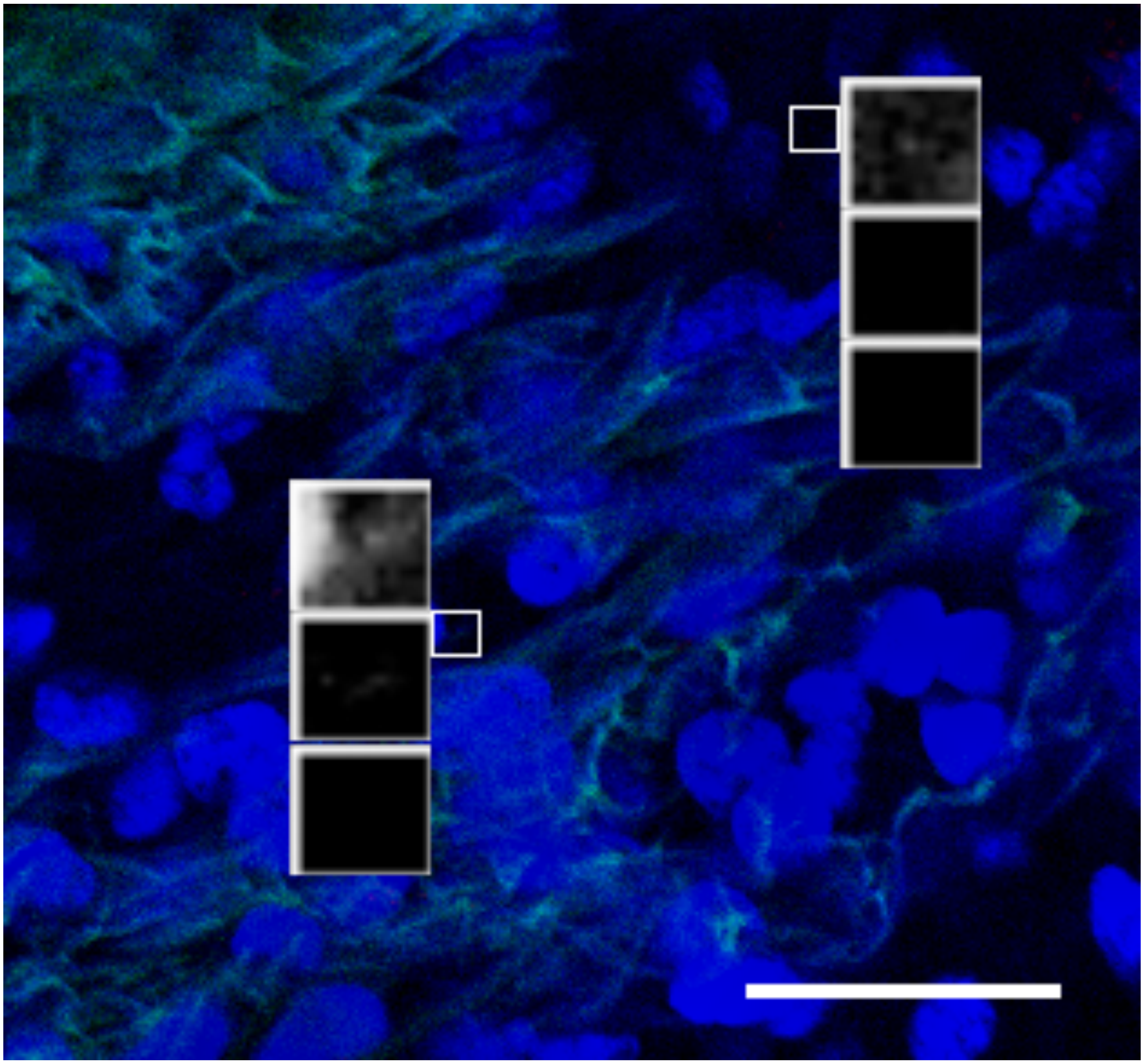

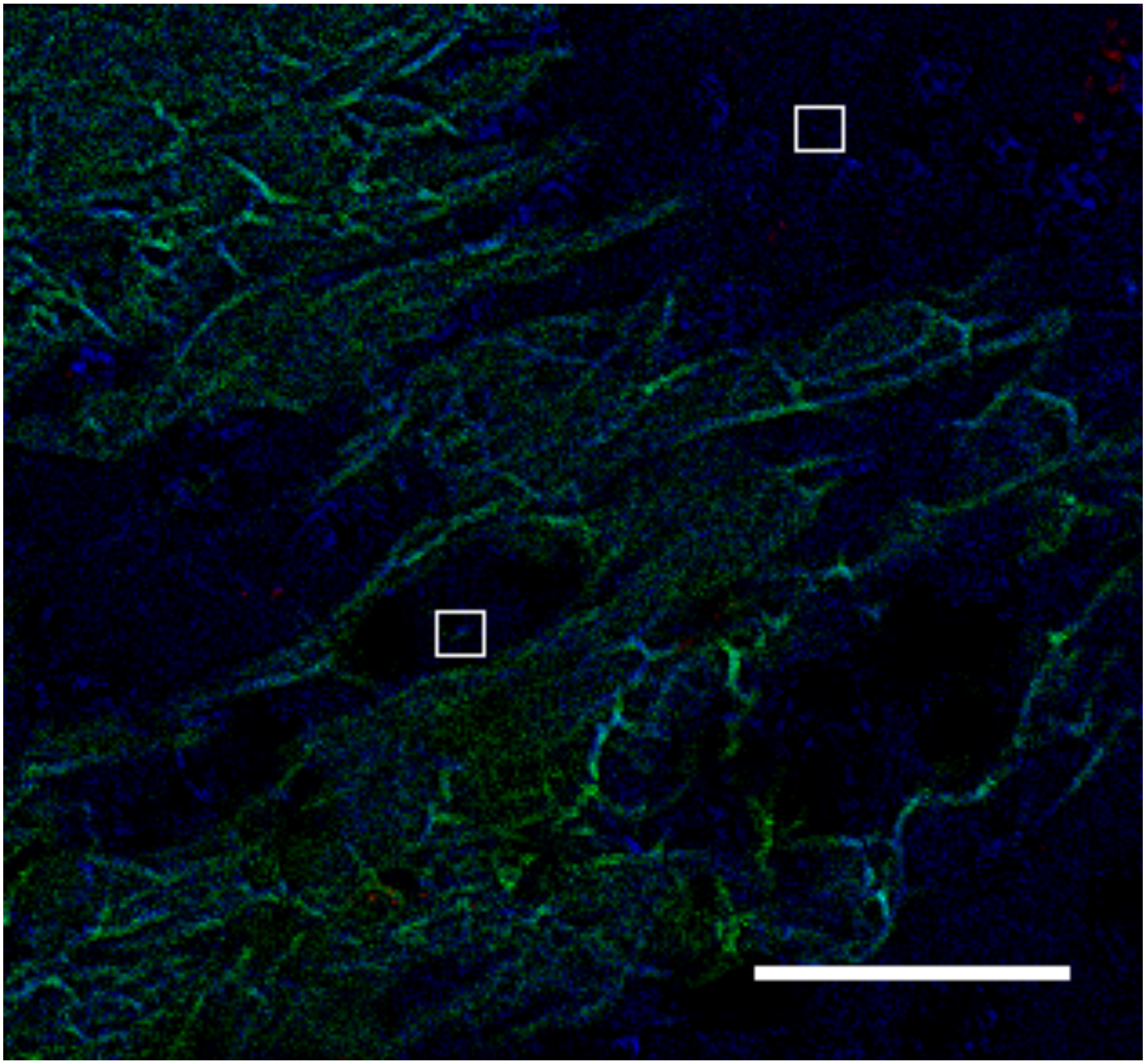

Figure SR2.14: Examples showing the specificity of our FISH protocol by performing staining with antisense probes, i.e. reversed sequences which have no sequence matches and therefore there was no probe-specific bacteria detected in the ileum of *T. zeteki* workers. Staining was performed with a 16S anti-sense Mollicutes-specific probe with an attached alexa488 fluorochrome, which would have appeared as bright green spots if there was staining and a 16S anti-sense Alpha-Proteobacteria probe with an attached Cy5 fluorochrome, which would have appeared as bright red spots if there was staining. DAPI staining of bacterial DNA was used as a positive control and appears as bright blue spots. Only the DAPI signal could be detected which shows that bacteria are present but the anti-sense probes are not hybridizing to them (as expected). Probe sequences are shown in Table S2.

C. longiscapus – rectum

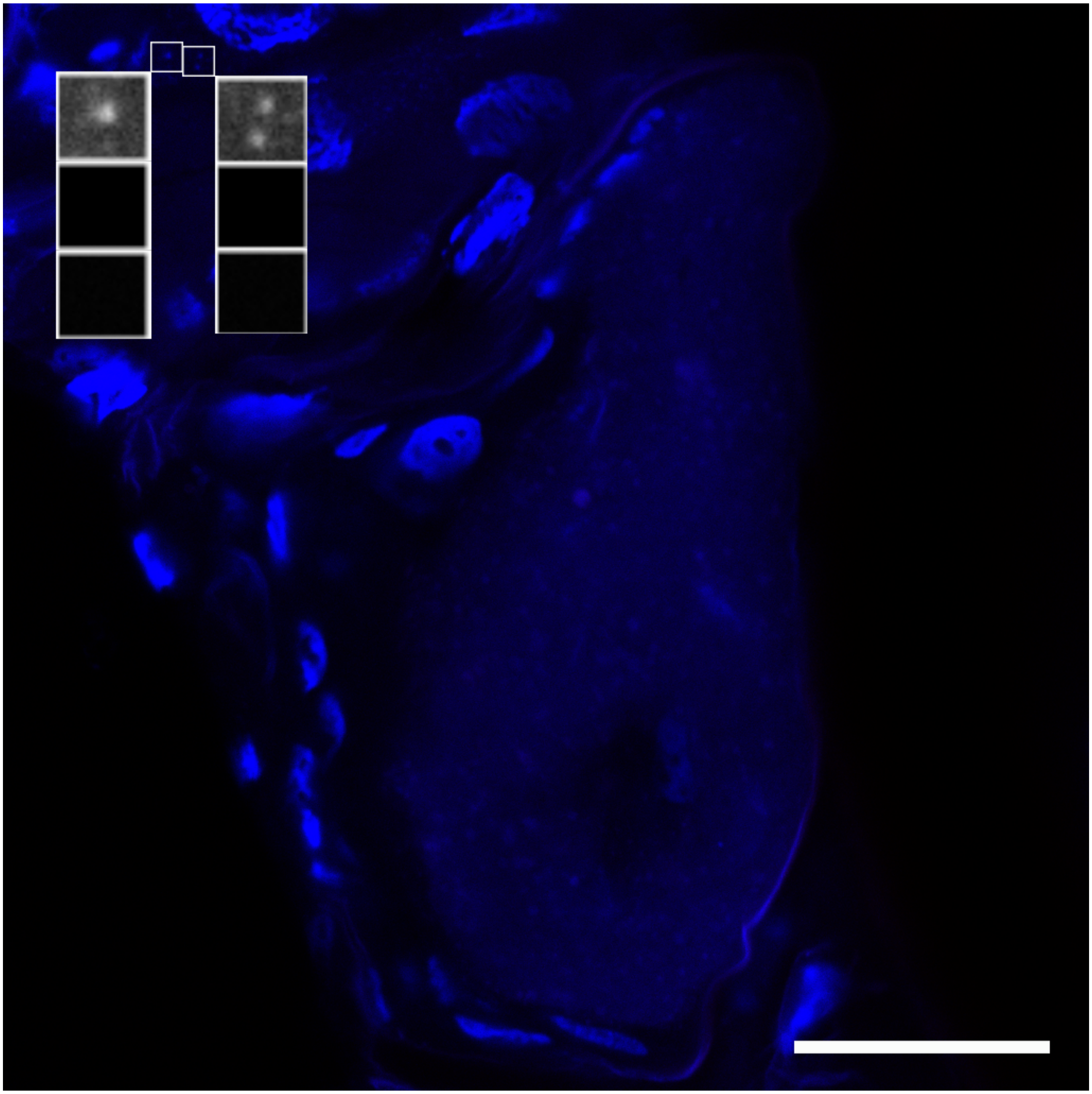

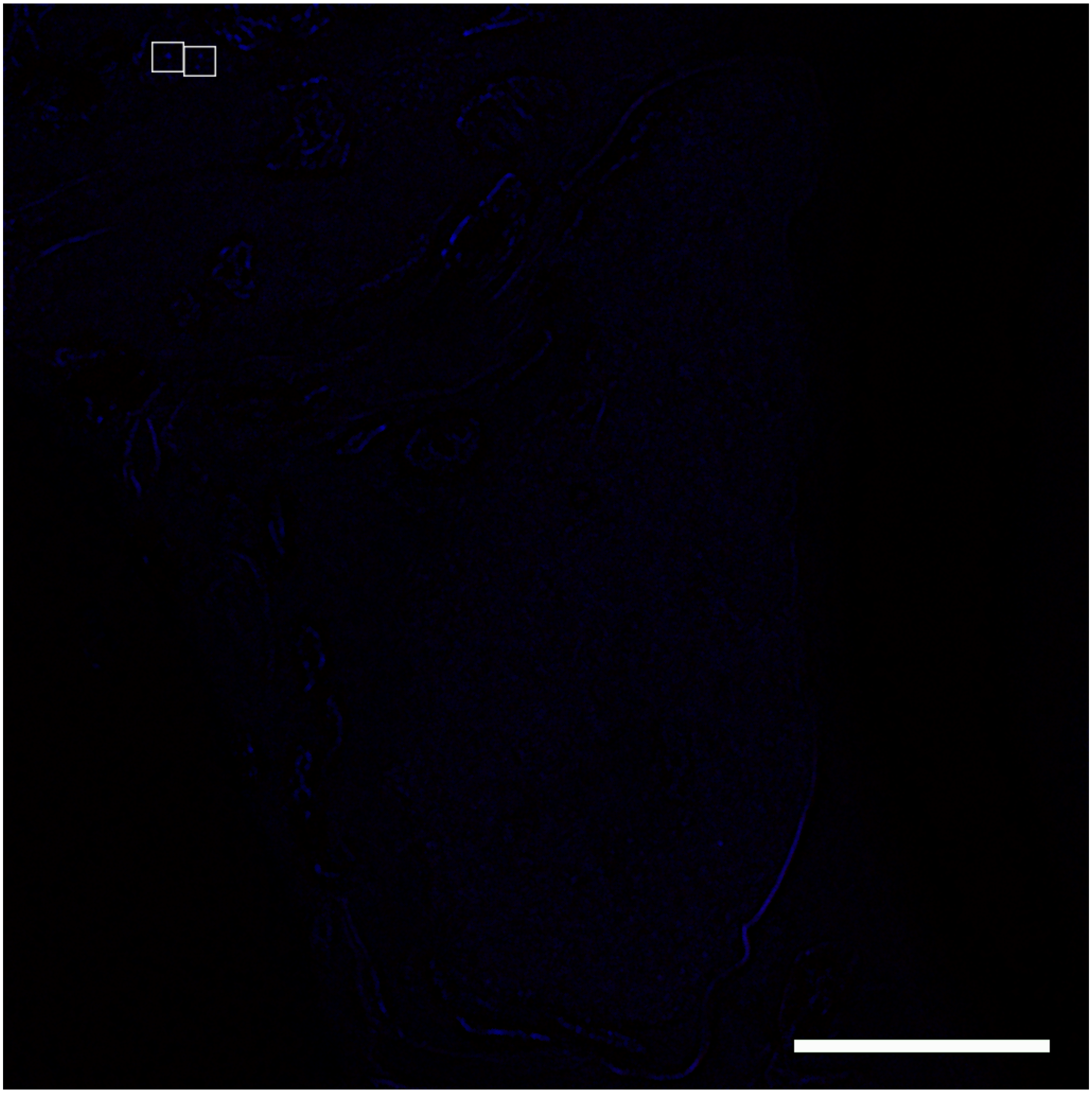

Figure SR2.15: Examples of no *Rhizo11* bacteria detected in the rectum of *C. longiscapus* workers. Staining was performed with a *Rhizo11* OTU-specific probe with an attached Cy3 fluorochrome, which would appear as bright green spots if present but the absence of signals shows no *Rhizo11* bacteria in this tissue. DAPI staining of bacterial DNA was used as a positive control in each experiment (appears as bright blue spots). The red channel shows an image taken at a wavelength >630nm where we would normally detect probes with an attached Cy5 fluorochrome but here they functioned as autofluorescence controls because we did not use any Cy5 probes. FISH images suggested that *Rhizo11* bacteria are absent.

C. longiscapus – ileum

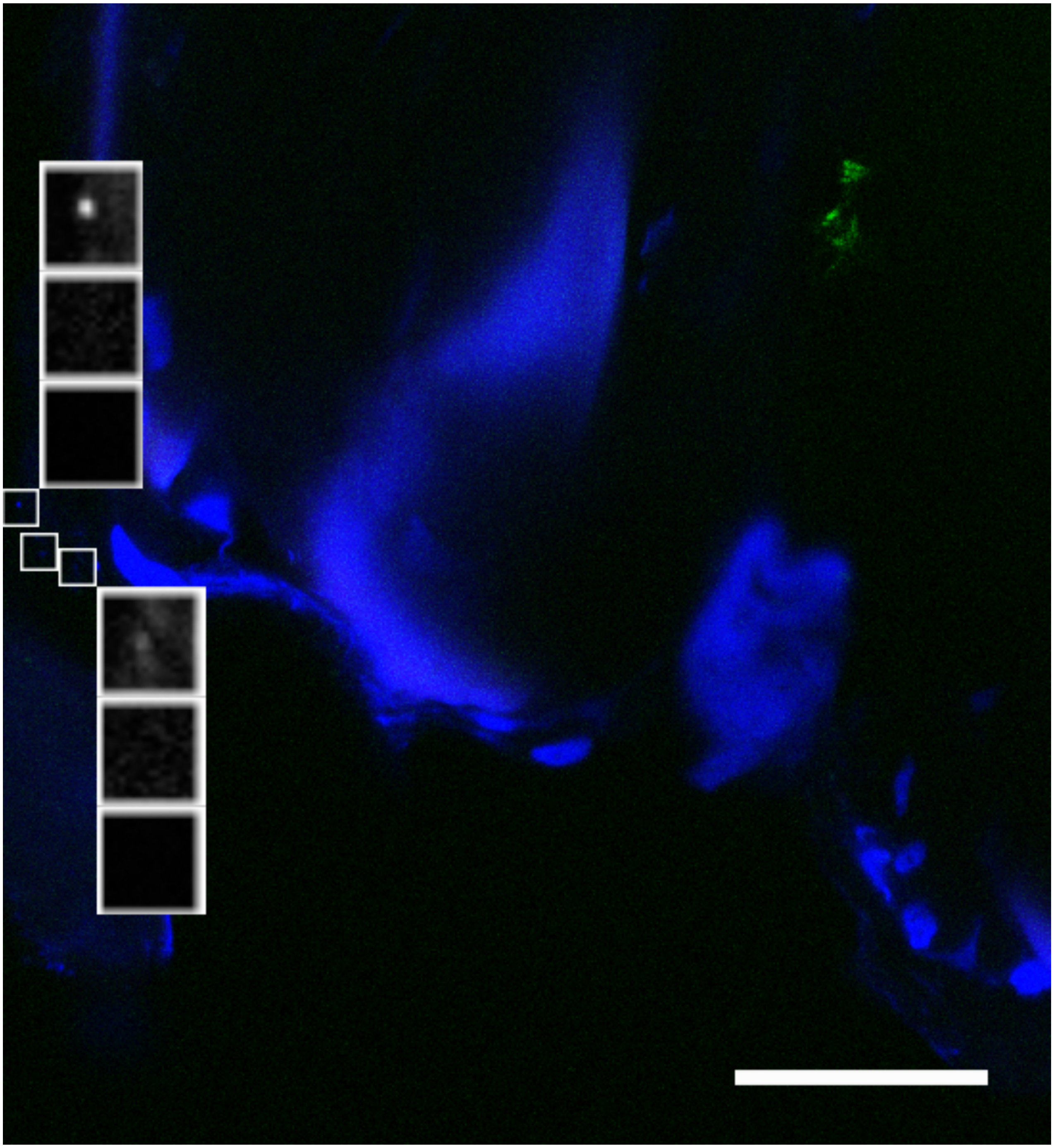

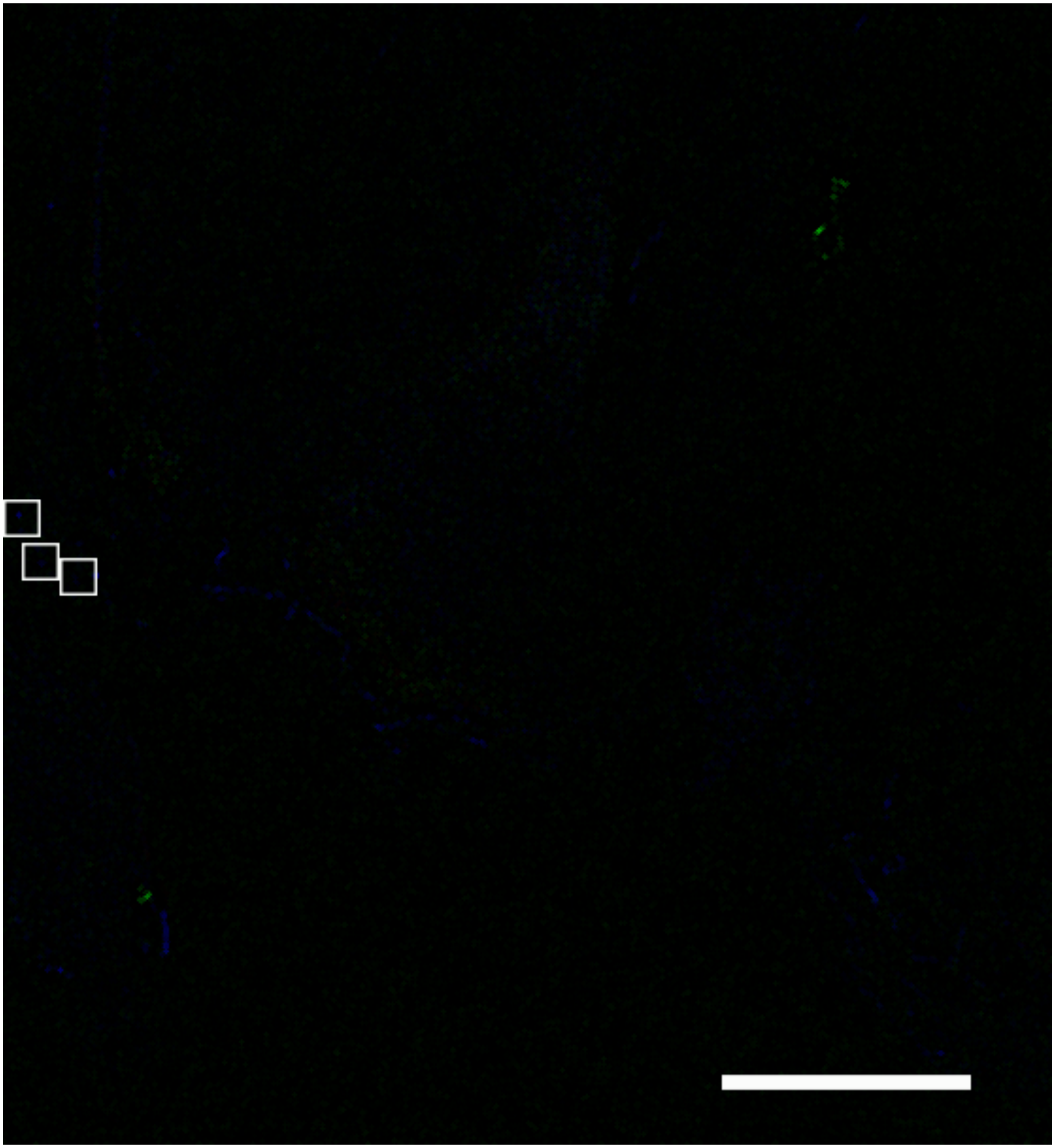

Figure SR2.16: Examples of no *Rhizo11* bacteria detected in the ileum of *C. longiscapus* workers. Staining was performed with a *Rhizo11* OTU-specific probe with an attached Cy3 fluorochrome, which would appear as bright green spots if present but the absence of signals shows no *Rhizo11* bacteria in this tissue. DAPI staining of bacterial DNA was used as a positive control in each experiment and it appears as bright blue spots. The red channel shows an image taken at a wavelength >630nm where we would normally detect probes with an attached Cy5 fluorochrome but here they functioned as autofluorescence controls because we did not use any Cy5 probes. FISH images suggested that *Rhizo11* bacteria are absent.

***C. longiscapus* – hindgut**

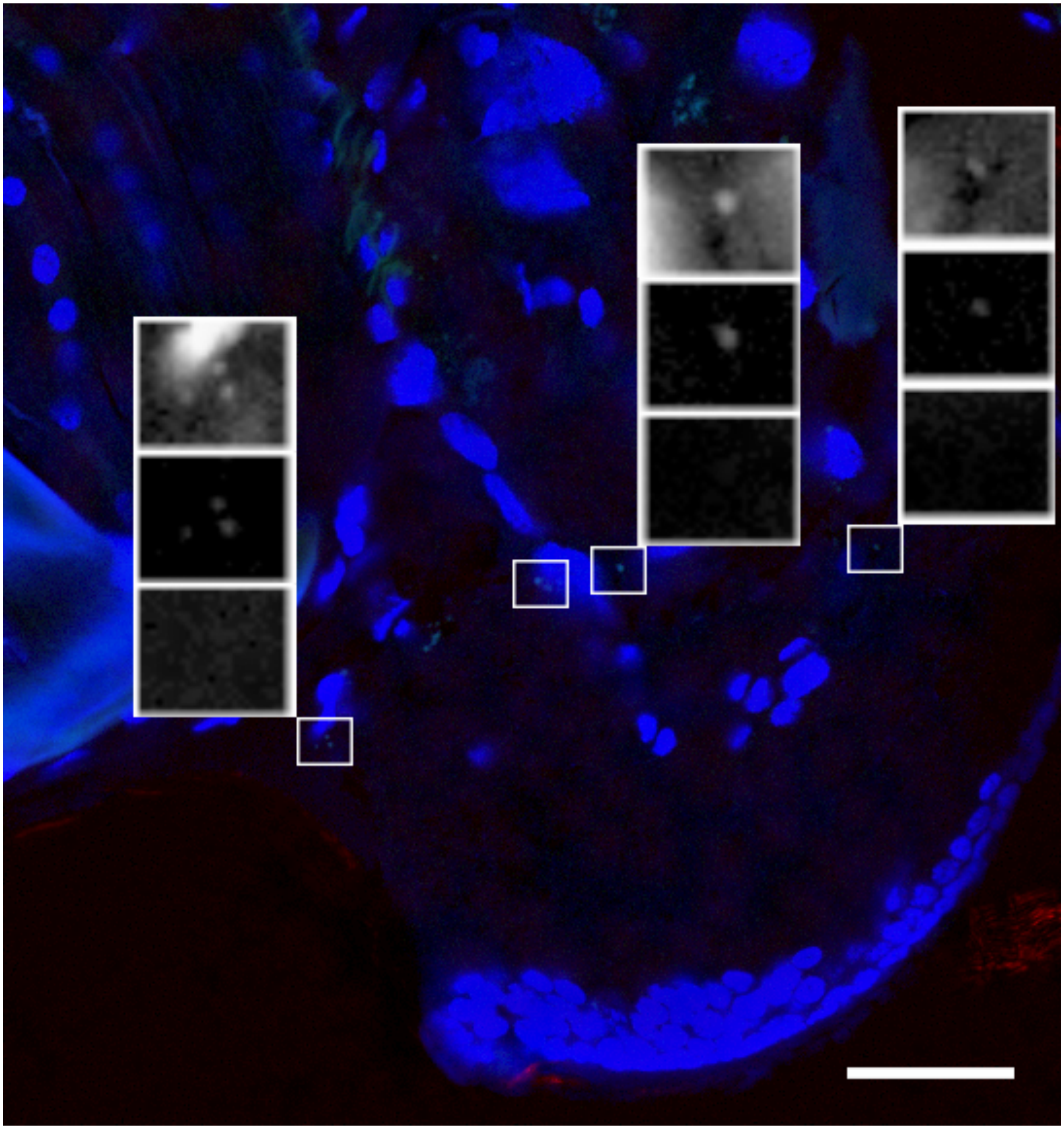

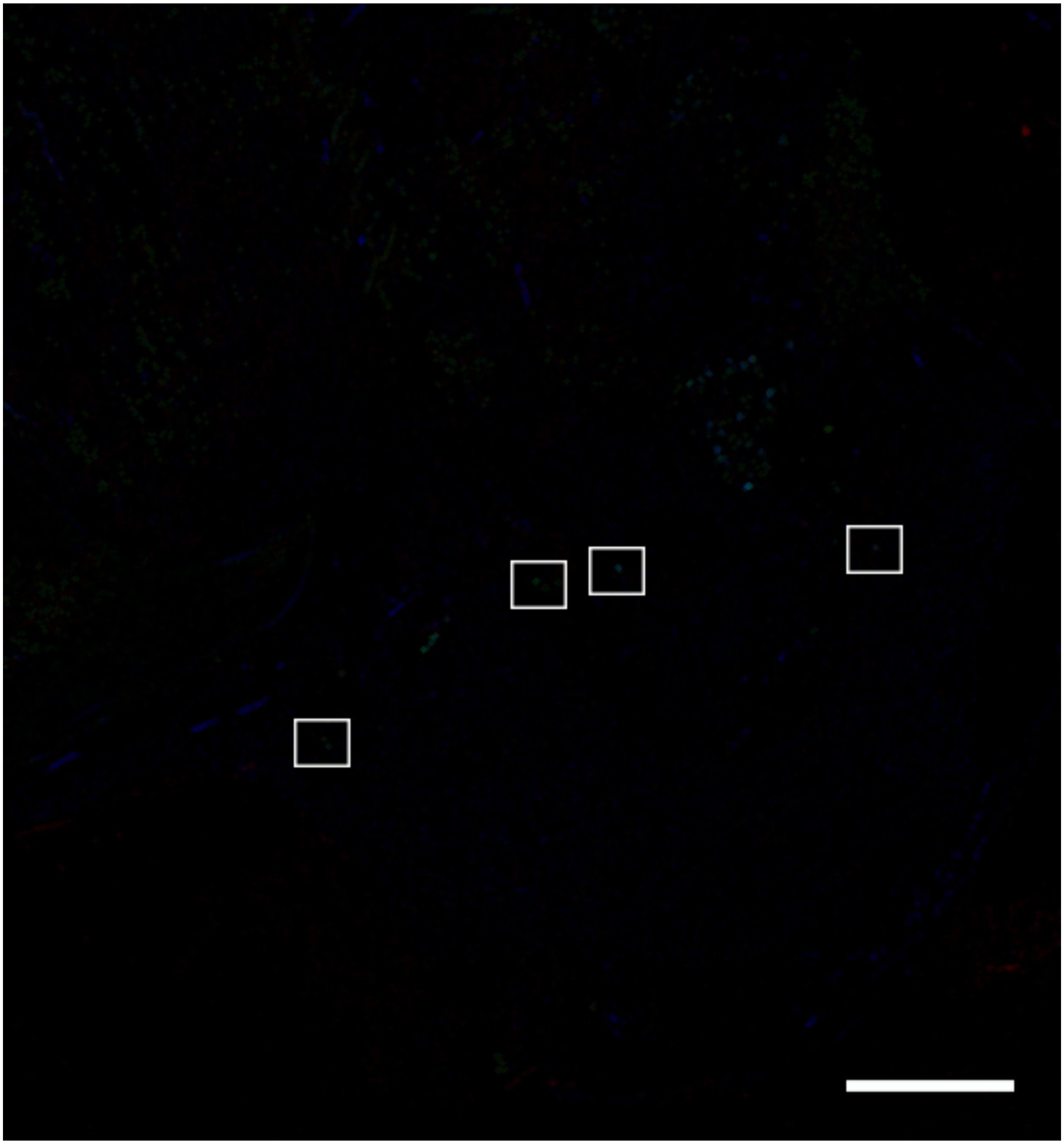

Figure SR2.17: Examples of Mollicutes bacteria detected in the hindgut of *C. longiscapus* workers. Staining was performed with a 16S Mollicutes-specific probe (Entom_A488; Table S2) with an attached alexa488 fluorochrome, which appears as bright green spots when present and a *RhiAcro1* specific probe (Phyllo_Cy5; Table S2) with an attached Cy5 fluorochrome, which would appear as bright red spots but it does not show up here because there are no *RhiAcro1* bacteria present in this tissue. DAPI staining appears as bright blue spots. Each of the probe-specific channels (green and red) functions also as autofluorescence control for the other one, because both probes used were specific to different OTUs and therefore there were no bacteria in common that they could both detect. FISH images suggested that Mollicutes bacteria are sparsely present and *RhiAcro1* bacteria absent.

C. longiscapus – ileum

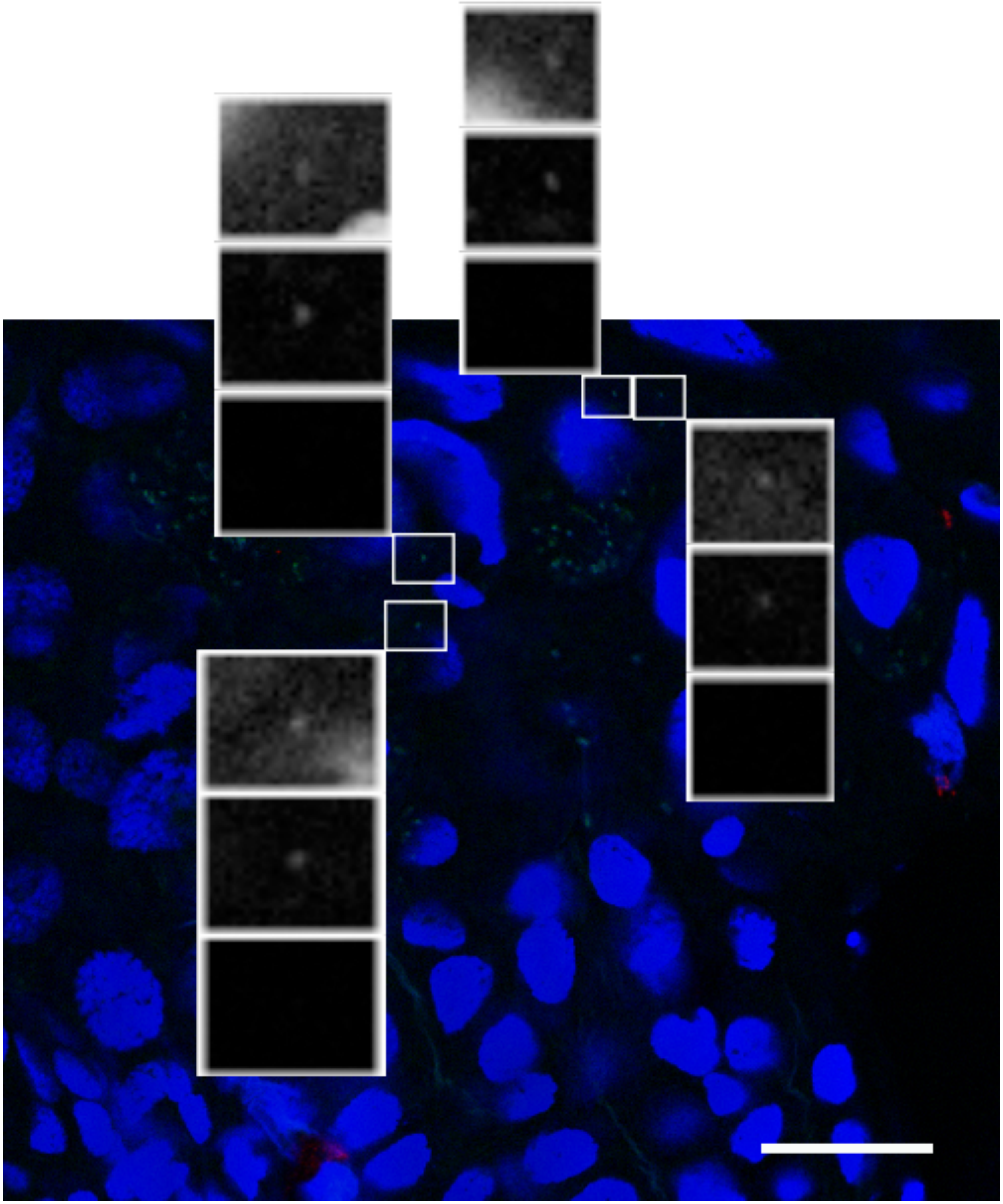

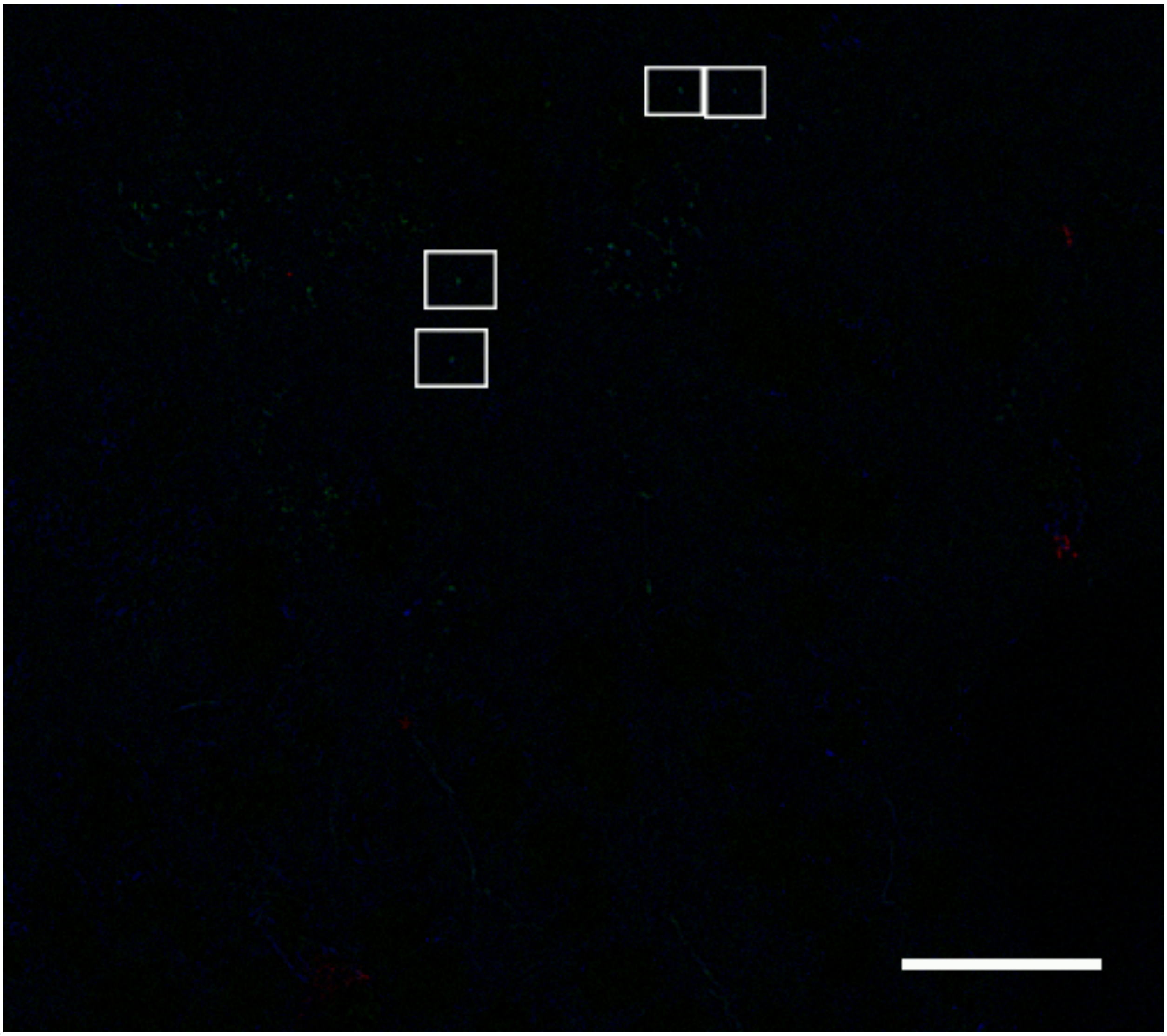

Figure SR2.18: Examples of Mollicutes bacteria detected in the ileum of *C. longiscapus* workers. Staining was performed with a 16S Mollicutes-specific probe (Entom_A488; Table S2) with an attached alexa488 fluorochrome, which appears as bright green spots when present and a *RhiAcro1* specific probe (Phyllo_Cy5; Table S2) with an attached Cy5 fluorochrome, which would appear as bright red spots but it does not show up here because there are no *RhiAcro1* bacteria present in this tissue. DAPI staining appears as bright blue spots. Each of the probe-specific channels (green and red) works also as autofluorescence control for the other one, because both probes used were specific to different OTUs and therefore there were no bacteria in common that they could both detect. FISH images suggested that Mollicutes bacteria are sparsely present and *RhiAcro1* bacteria absent.

C. costatus – ileum

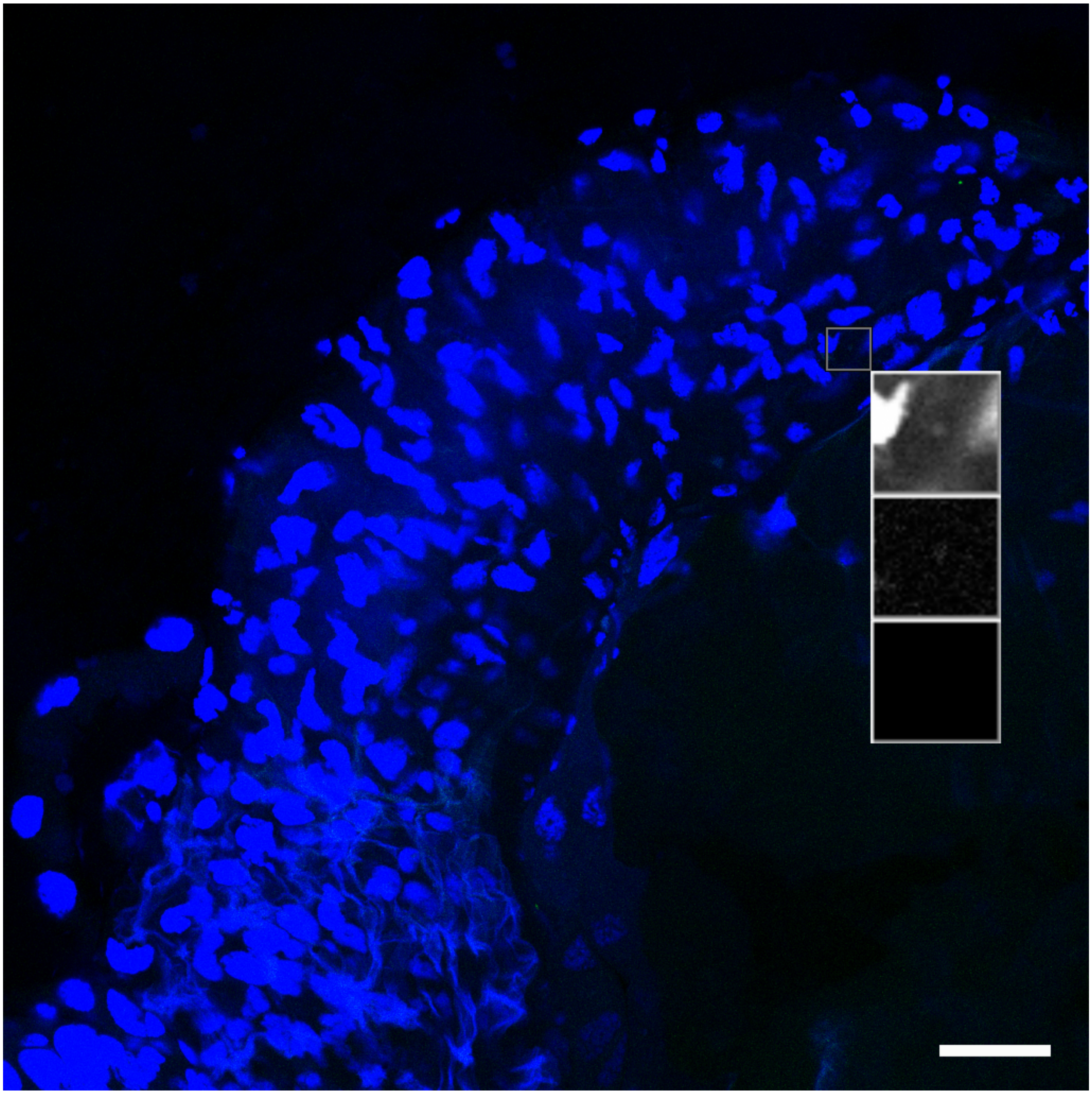

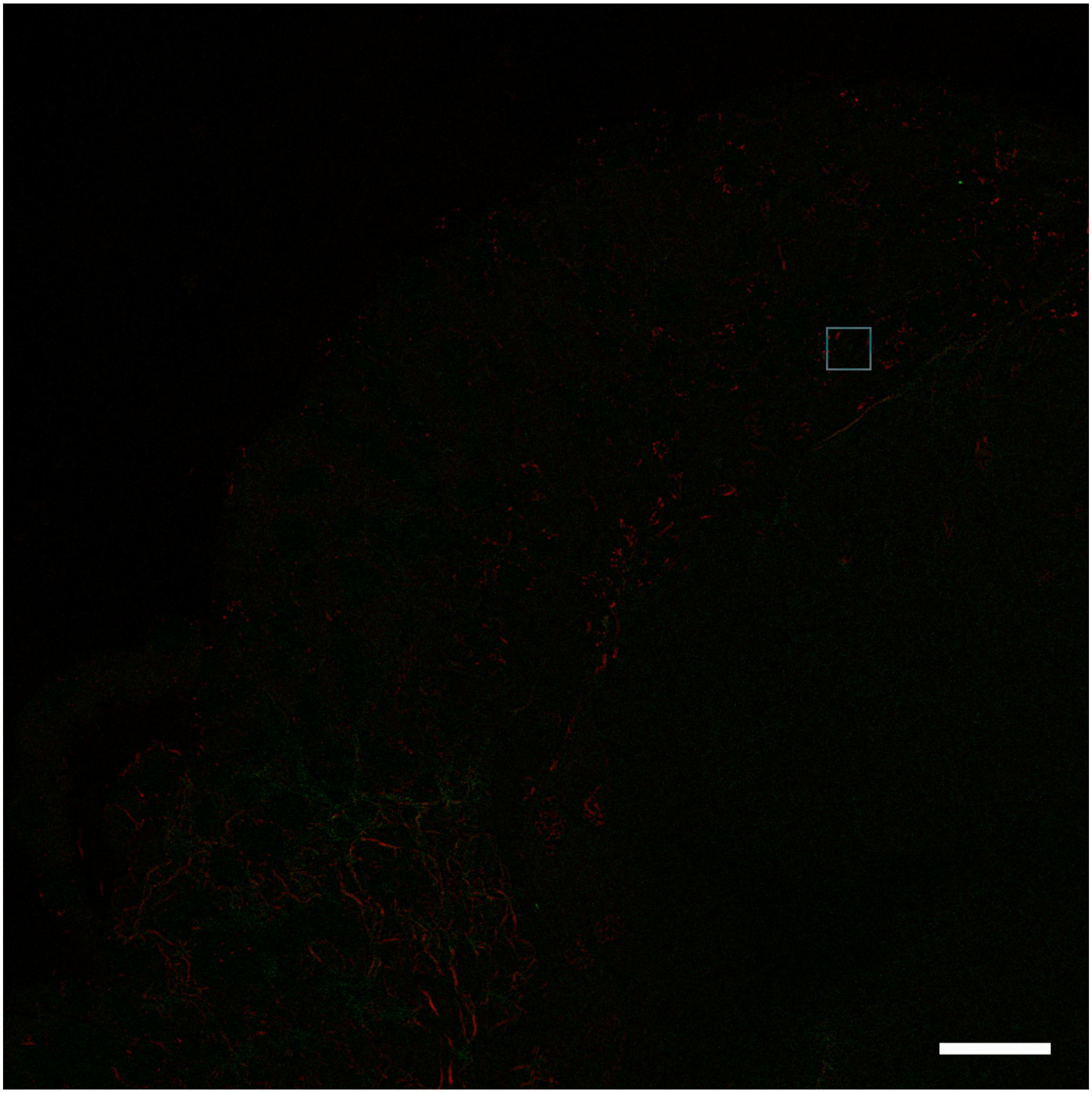

Figure SR2.19: Example of a Mollicutes bacterium detection in the ileum of *C. costatus* workers. Staining was performed with a 16S Mollicutes-specific probe (Entom_A488; Table S2) with an attached alexa488 fluorochrome, which appears as a bright green spot and an Alphaproteobacteria specific probe (Phyllo_Uni_Cy5; Table S2) with an attached Cy5 fluorochrome, which would appear as bright red spots but it does not show up here because there are no alpha-Proteobacteria present in this tissue. DAPI staining appears as bright blue spots. Each of the probe-specific channels (green and red) works also as autofluorescence control for the other one, because both probes used were specific to different OTUs and therefore there were no bacteria in common that they could both detect. FISH images suggested that Mollicutes bacteria are sparsely present and Alphaproteobacteria absent.

C. costatus – rectum

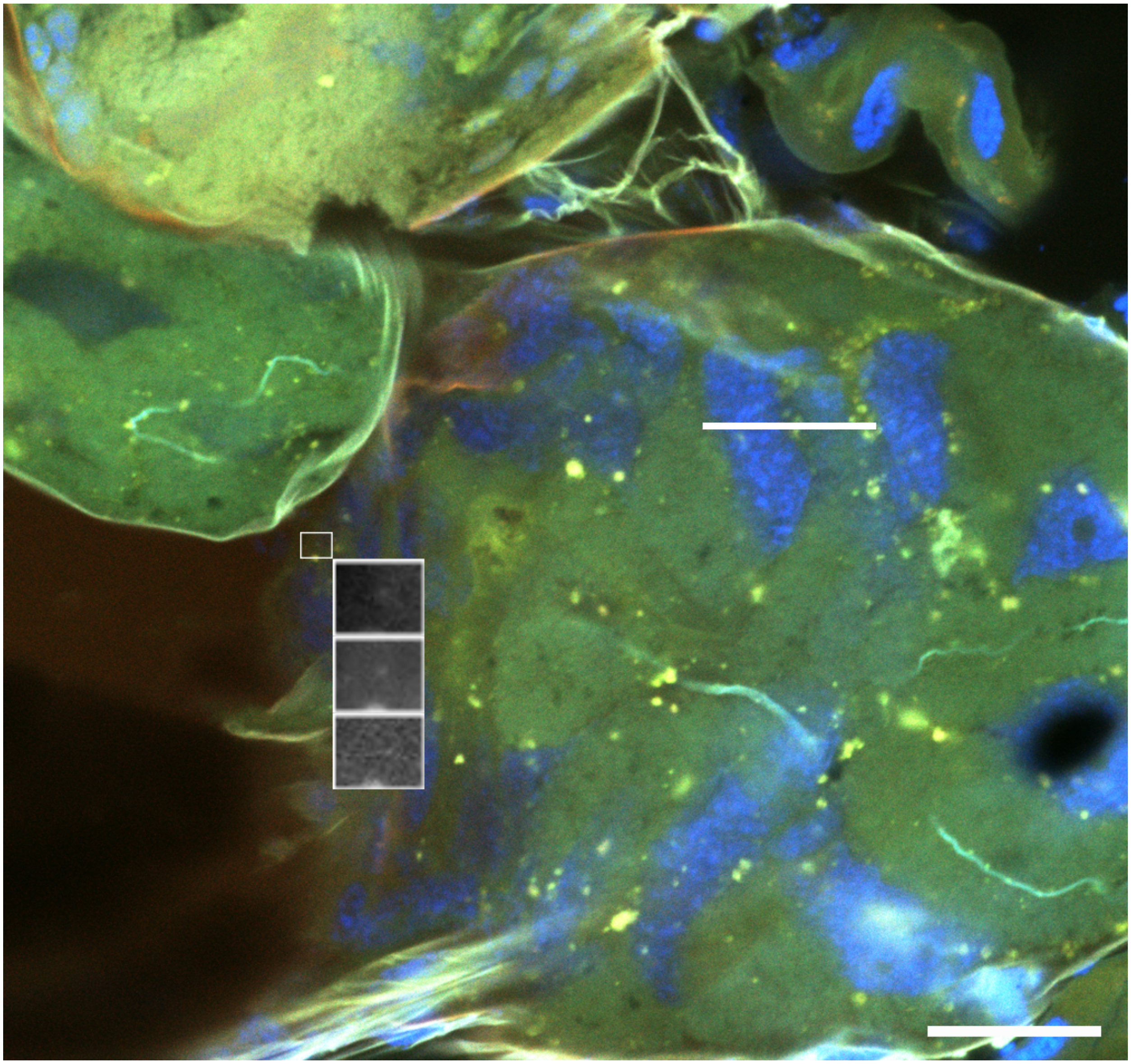

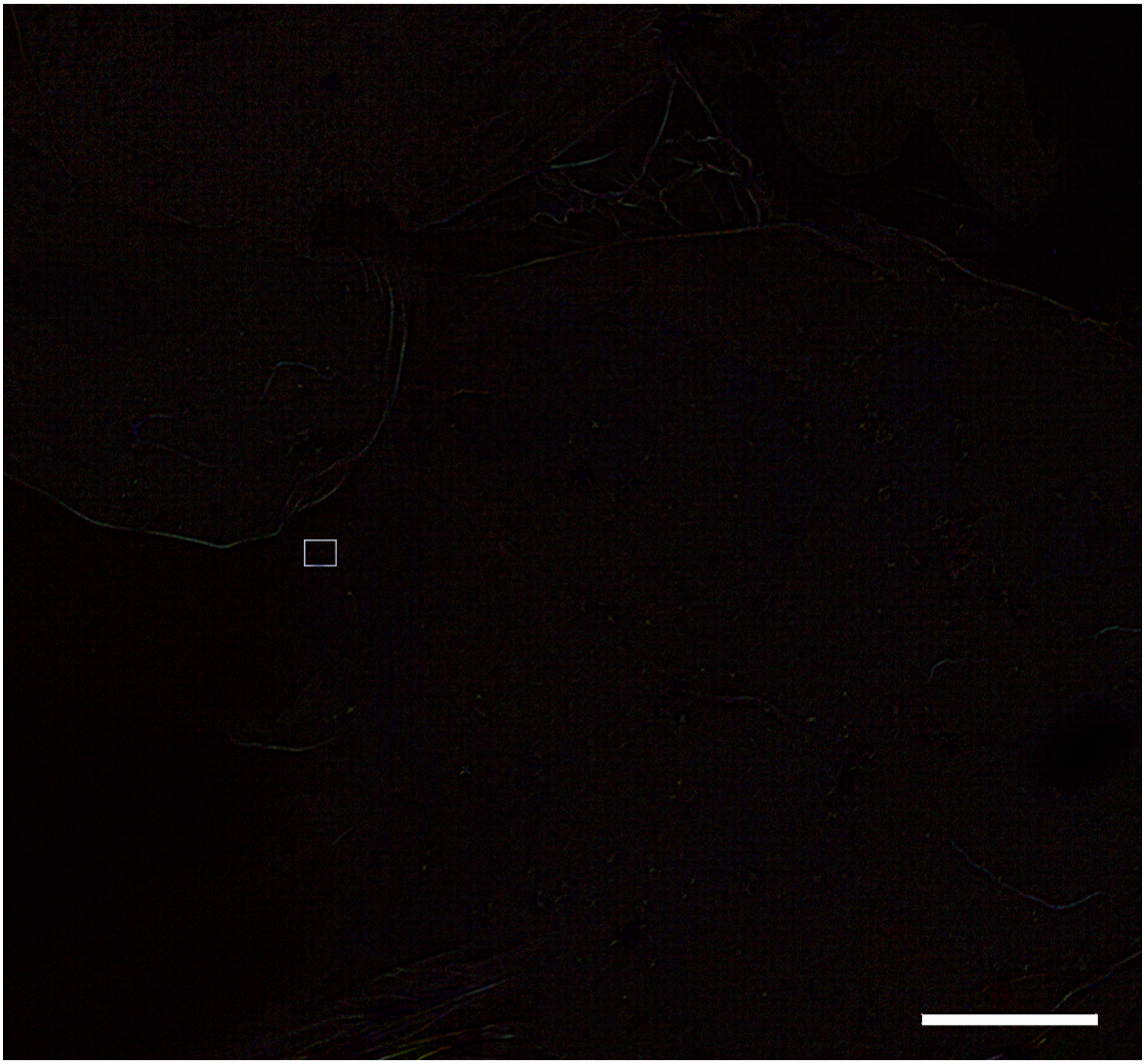

Figure SR2.20: Example of a Mollicutes bacterium detection in the rectum of *C. costatus* workers. Staining was performed with a 16S Mollicutes-specific probe (Entom_A488; Table S2) with an attached alexa488 fluorochrome, which appears as a bright green spot and an alpha-Proteobacteria specific probe (Phyllo_Uni_Cy5; Table S2) with an attached Cy5 fluorochrome, which would appear as bright red spots but it does not show up here because there are no alpha-Proteobacteria present in this tissue. DAPI staining appears as bright blue spots. Each of the probe-specific channels (green and red) works also as autofluorescence control for the other one, because both probes used were specific to different OTUs and therefore there were no bacteria in common that they could both detect. FISH images suggested that Mollicutes bacteria are sparsely present and Alphaproteobacteria absent.

M. smithii **– fat body cell**

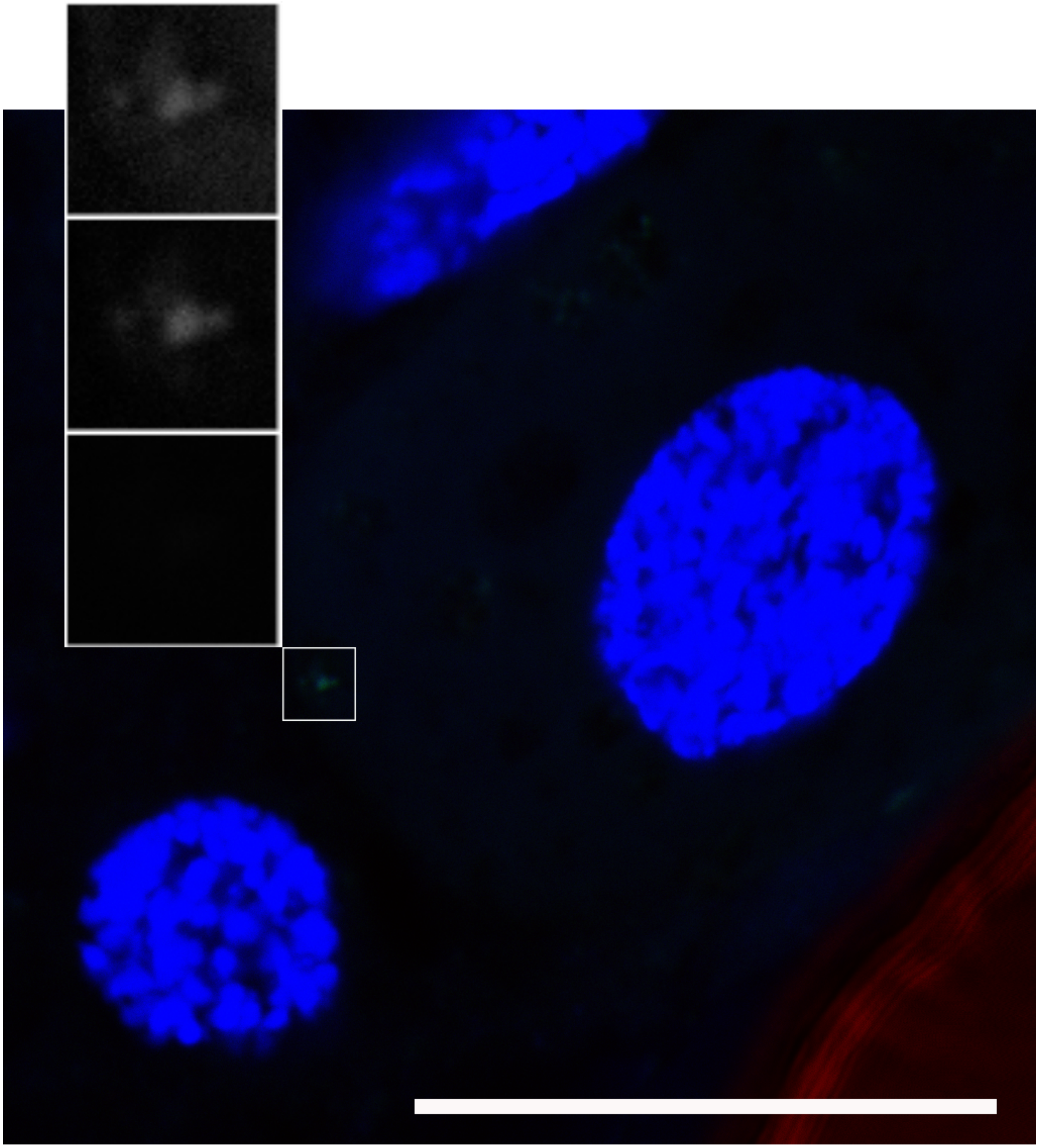

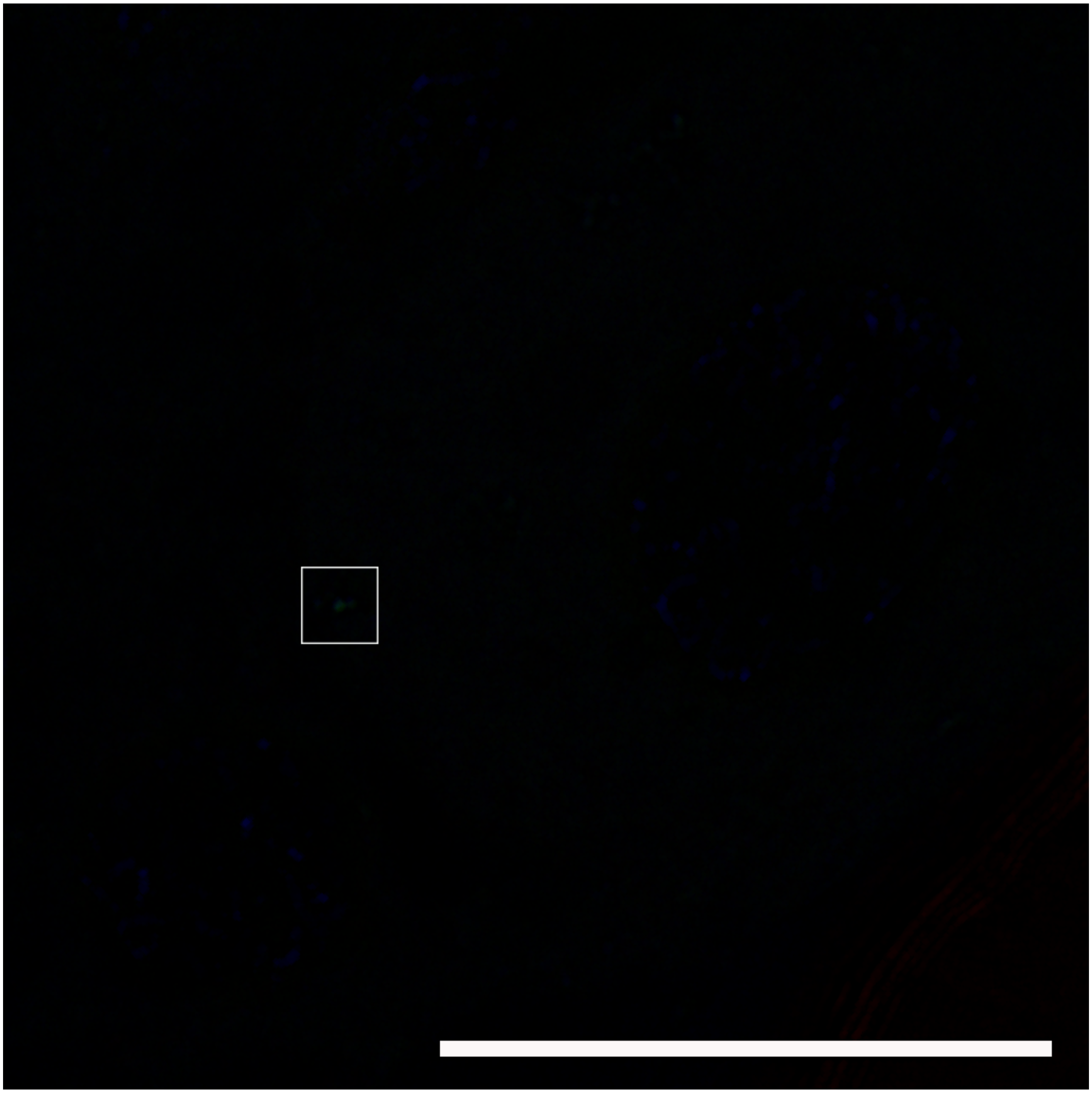

Figure SR2.21: Examples of a Mollicutes bacterium detection in a fat body cell of *M. smithii* workers. Staining was performed with a 16S Mollicutes-specific probe (Entom_A488; Table S2) with an attached alexa488 fluorochrome, which appears as a bright green spots and a *RhiAcro1* specific probe (Phyllo_Cy5; Table S2) with an attached Cy5 fluorochrome, which would appear as bright red spots but it does not show up here because there are no *RhiAcro1* bacteria in the fat body cells. DAPI staining appears as bright blue spots. Each of the probe-specific channels (green and red) works also as autofluorescence control for the other one, because both probes used were specific to different OTUs and therefore there were no bacteria in common that they could both detect. FISH images suggested that Mollicutes bacteria may be sparsely present but more likely are absent, similar to *RhiAcro1* bacteria.

M. smithii – ileum

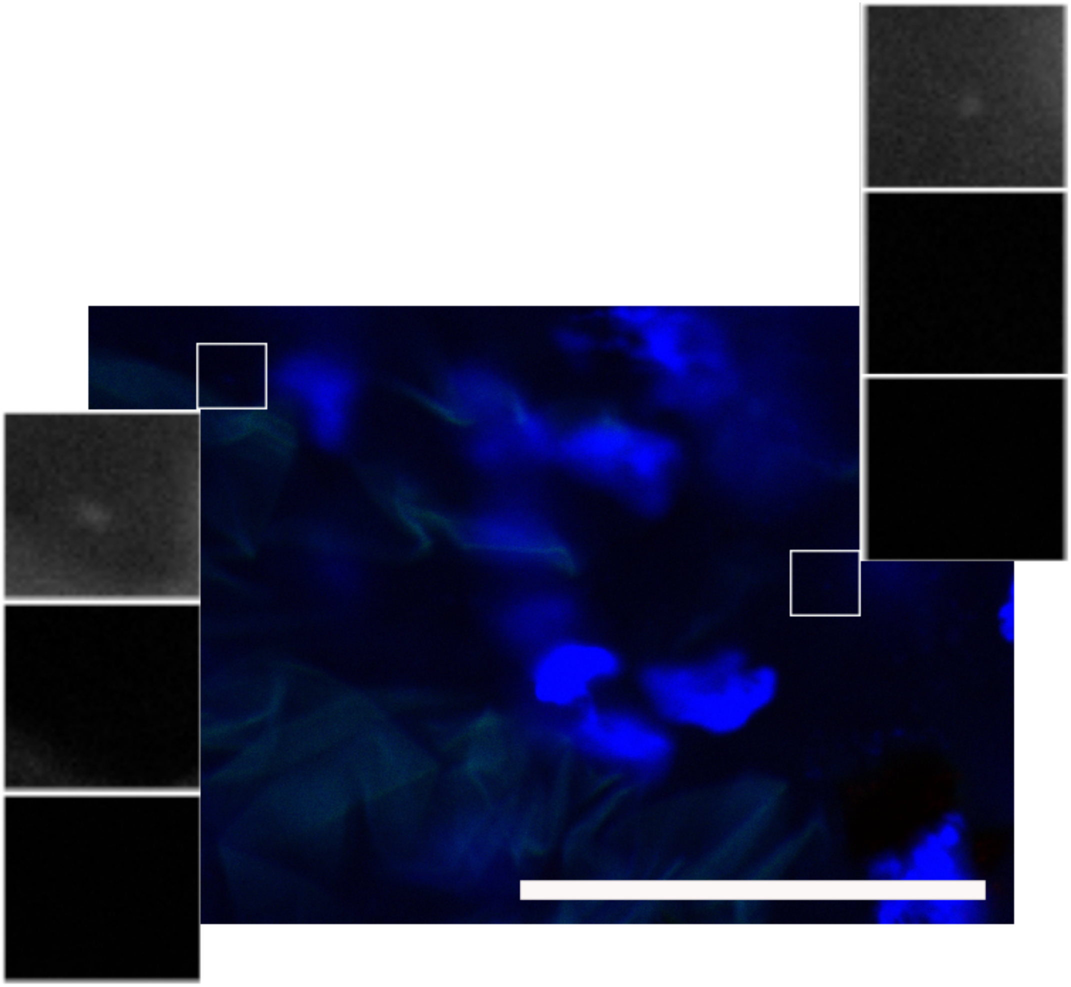

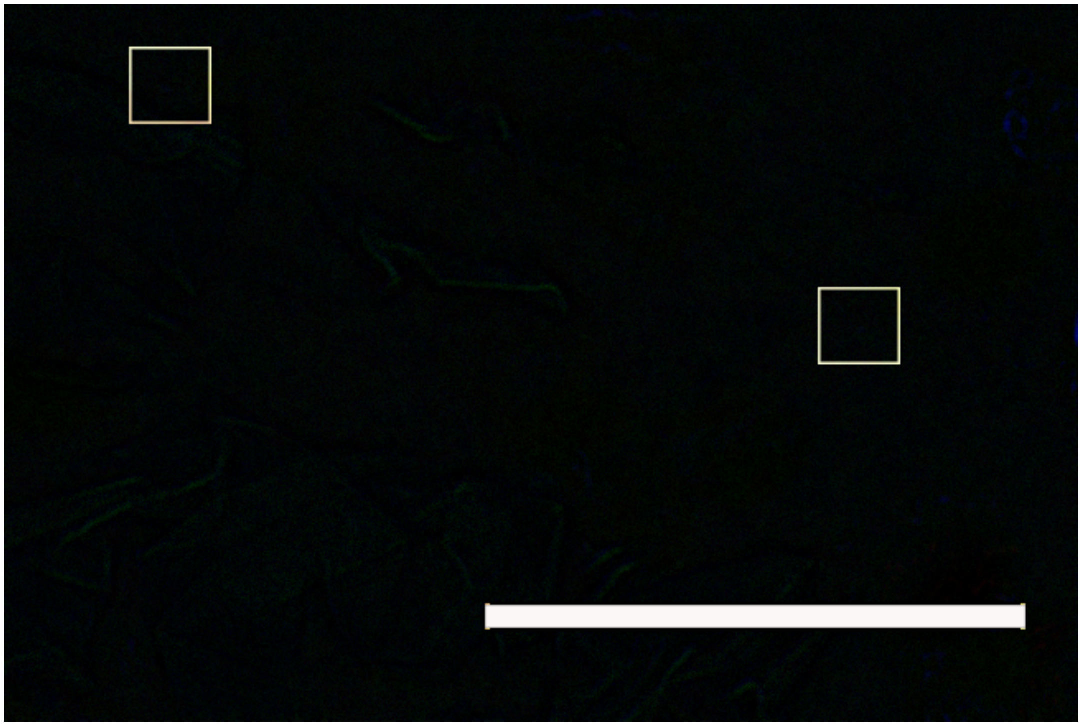

Figure SR2.22: Examples of no Mollicutes and no alpha-Proteobacteria detected in the ileum of *M. smithii* workers. Staining was performed with a 16S Mollicutes-specific probe (Entom_A488; Table S2) with an attached alexa488 fluorochrome, which would appear as bright green spots if present and an apha-Proteobacteria specific probe (Phyllo_Uni_Cy5; Table S2) with an attached Cy5 fluorochrome, which would appear as bright red spots if present. DAPI staining of bacterial DNA was used as a positive control in each experiment and it appears as the two bright blue spots which are neither Mollicutes nor alpha-Proteobacteria. FISH images suggested that Mollicutes bacteria and Alphaproteobacteria are absent.

***M. smithii* – ileum**

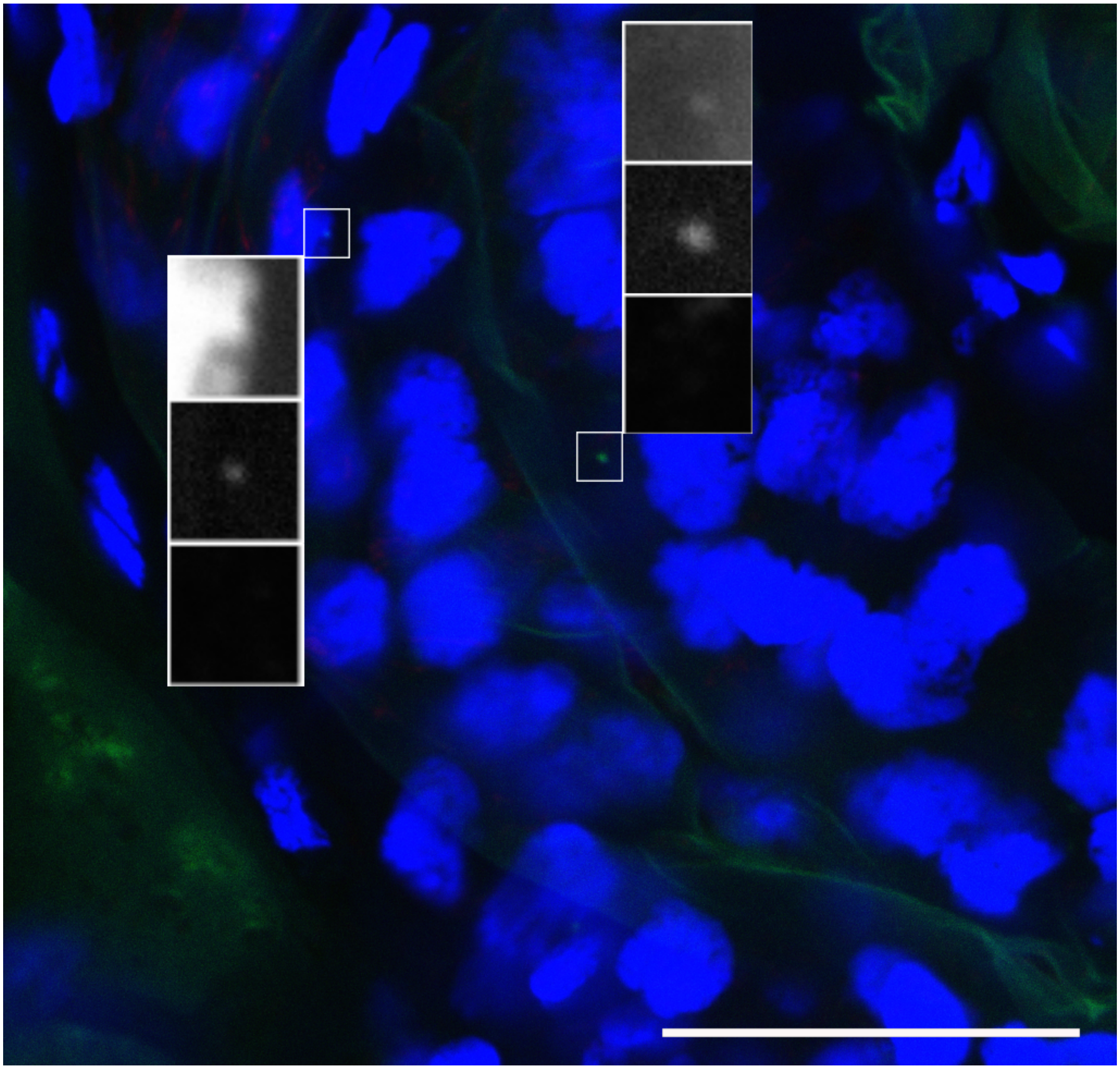

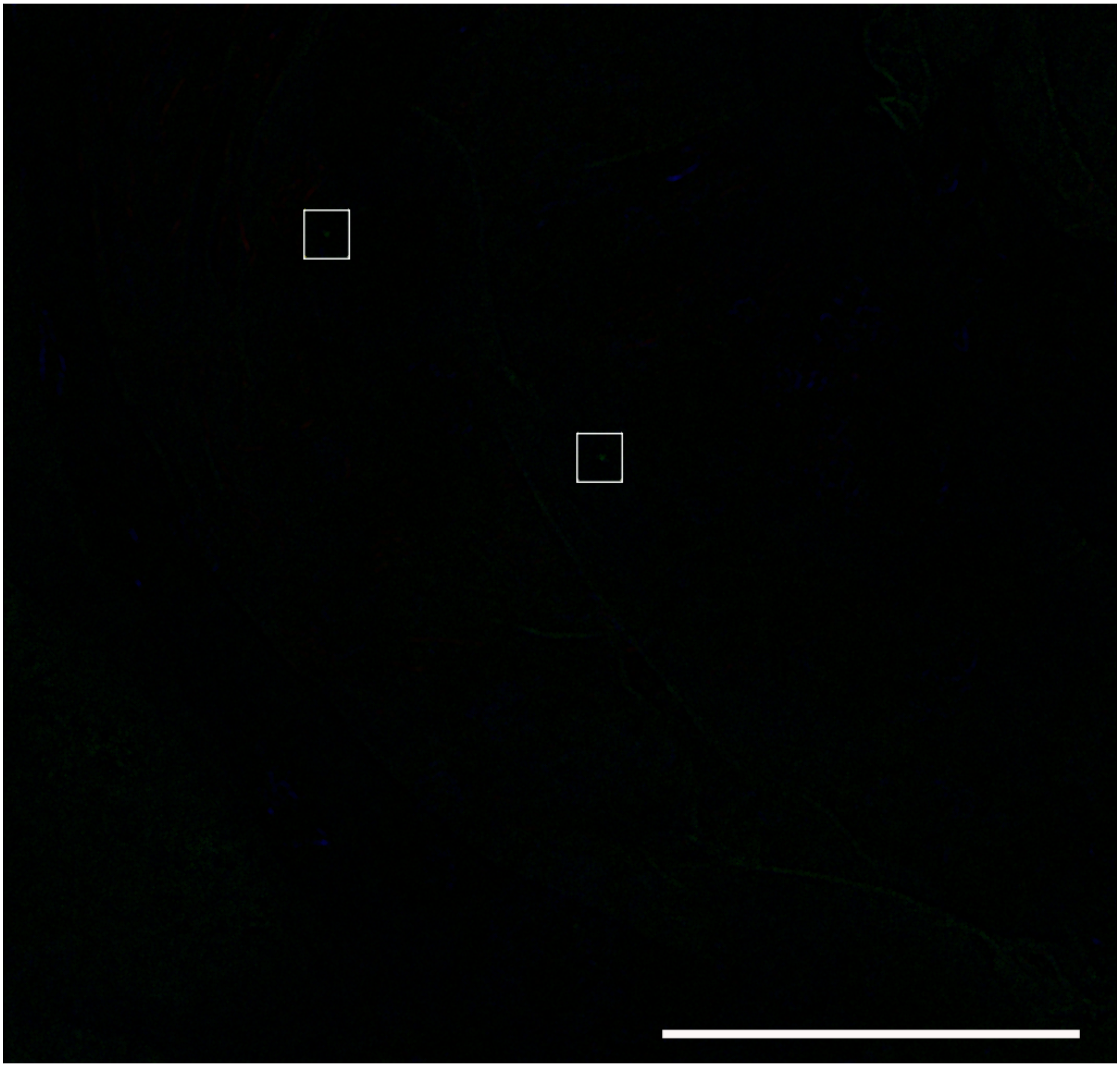

Figure SR2.23: Examples of Mollicutes bacteria detected in the ileum of *M. smithii* workers. Staining was performed with a 16S Mollicutes-specific probe (Entom_A488; Table S2) with an attached alexa488 fluorochrome, which appears as a bright green spots and a *RhiAcro1* specific probe (Phyllo_Cy5; Table S2) with an attached Cy5 fluorochrome, which would appear as bright red spots but it does not show up here because there were no *RhiAcro1* bacteria in the fat body cells. DAPI staining appears as bright blue spots. Each of the probe-specific channels (green and red) works also as autofluorescence control for the other one, because both probes used were specific to different OTUs and therefore there were no bacteria in common that they could both detect. FISH images suggested that Mollicutes bacteria may be sparsely present but since this was the only time they were detected, more likely absent, similar to *RhiAcro1* bacteria.

M. ednaella – fat body cells

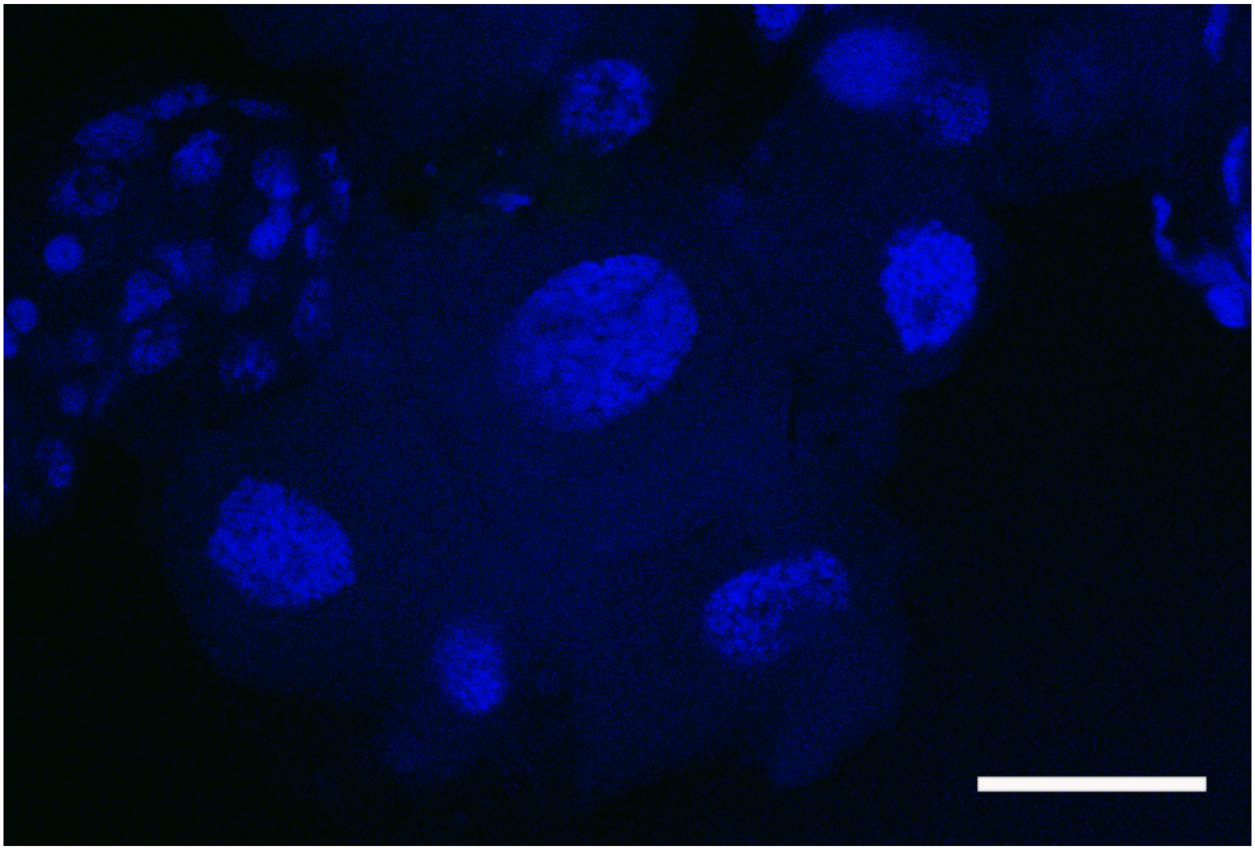

Figure SR2.24: Example of no bacteria detected in fat body cells of *M. ednaella* workers. Staining was performed with a 16S Mollicutes-specific probe (Entom_A488; Table S2) with an attached alexa488 fluorochrome, which would appear as bright green spots if present and an alpha-Proteobacteria-specific probe (Phyllo_Uni_Cy5; Table S2) with an attached Cy5 fluorochrome, which would appear as bright red spots if present. DAPI staining of bacterial DNA was used as a positive control in each experiment and it would appear as bright blue spots if there were any bacteria present. FISH images suggested that no bacteria were present.

M. ednaella – midgut

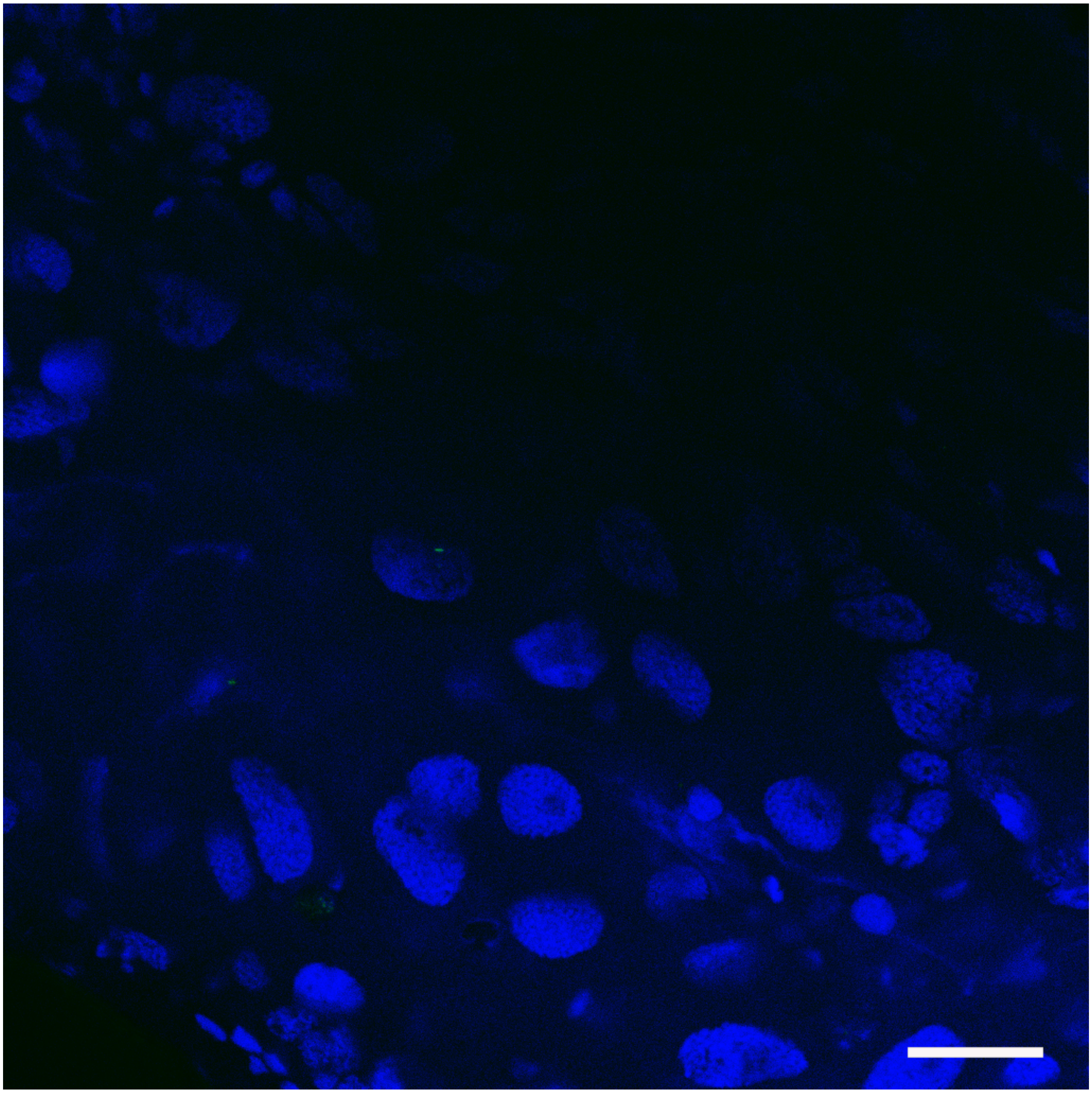

Figure SR2.25: Example of no bacteria detected in the midgut of *M. ednaella* workers. Staining was performed with a 16S Mollicutes-specific probe (Entom_A488; Table S2) with an attached alexa488 fluorochrome, which would appear as bright green spots if present and an alpha-Proteobacteria-specific probe (Phyllo_Uni_Cy5; Table S2) with an attached Cy5 fluorochrome, which would appear as bright red spots if present. DAPI staining of bacterial DNA was used as a positive control in each experiment and it would appear as bright blue spots if there were any bacteria present. FISH images suggested that no bacteria were present.

M. ednaella – rectum

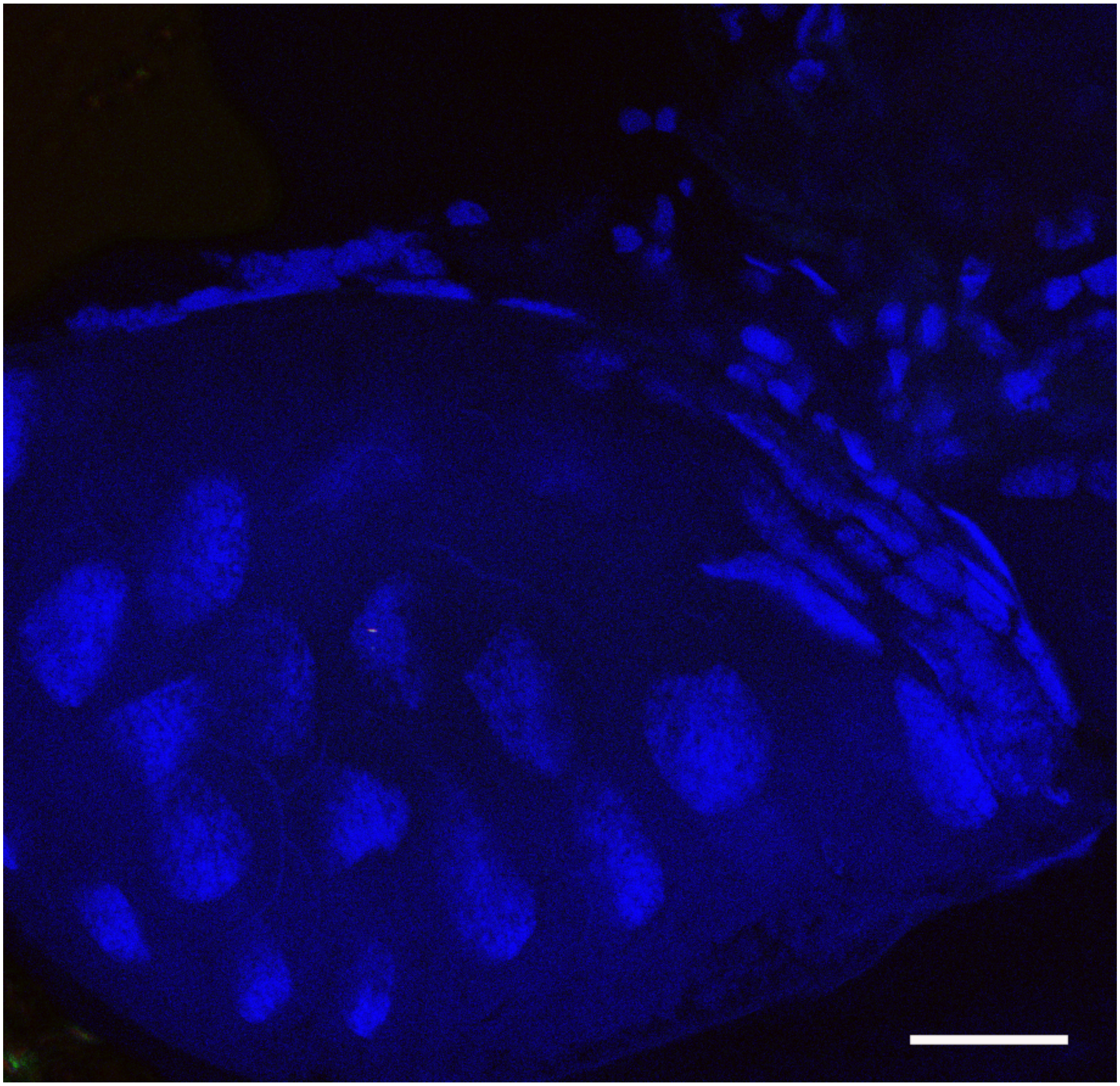

Figure SR2.26: Example of no bacteria detected in the rectum of *M. ednaella* workers. Staining was performed with a 16S Mollicutes-specific probe (Entom_A488; Table S2) with an attached alexa488 fluorochrome, which would appear as bright green spots if present and an alpha-Proteobacteria-specific probe (Phyllo_Uni_Cy5; Table S2) with an attached Cy5 fluorochrome, which would appear as bright red spots if present. DAPI staining of bacterial DNA was used as a positive control in each experiment and it would appear as bright blue spots if there were any bacteria present. FISH images suggested that no bacteria were present.

Ap. dentigerum – fat body cells

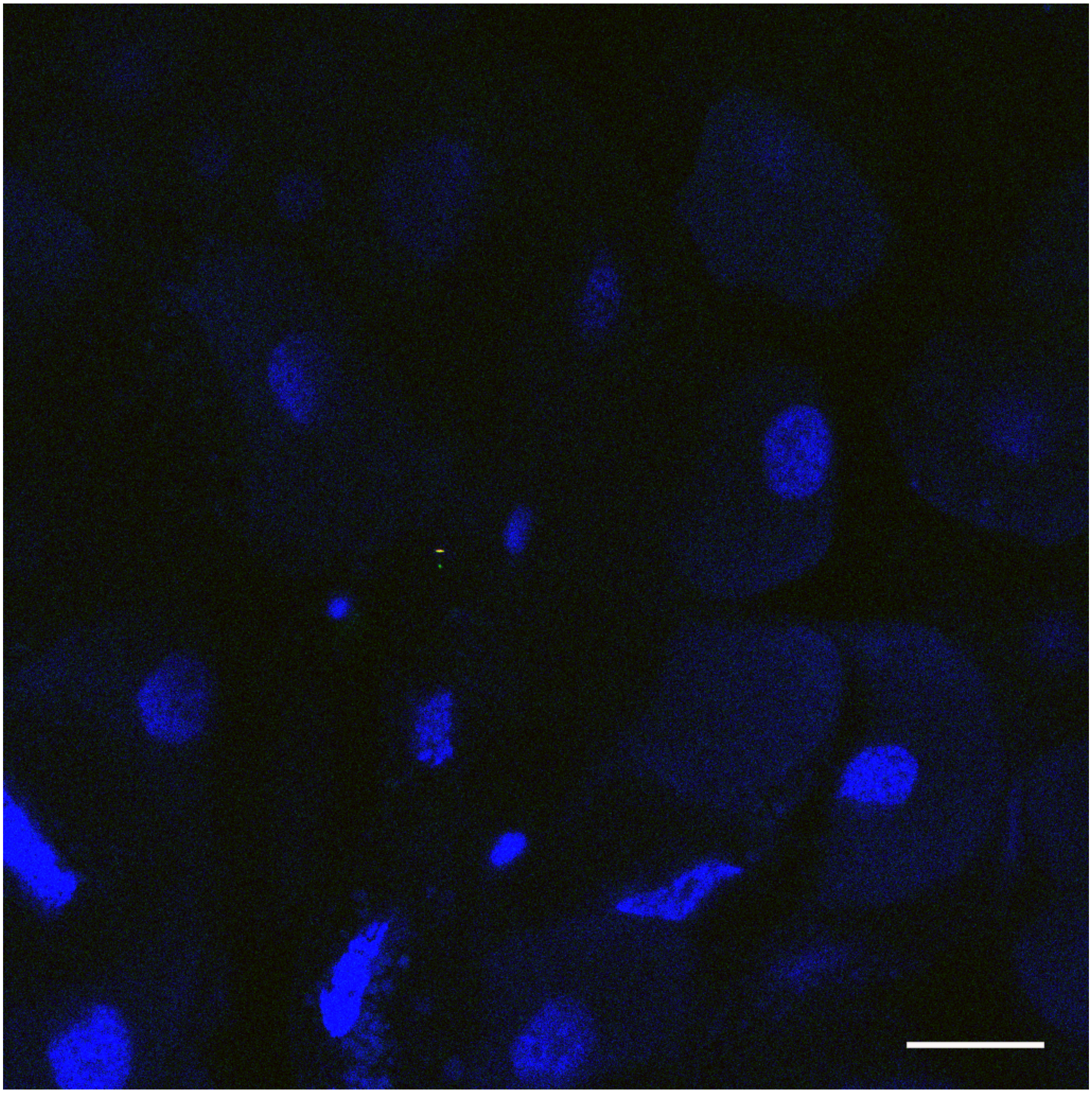

Figure SR2.27: Example of no bacteria detected in fat body cells of *Ap. dentigerum* workers. Staining was performed with a 16S Mollicutes-specific probe (Entom_A488; Table S2) with an attached alexa488 fluorochrome, which would appear as bright green spots if present and an alpha-Proteobacteria-specific probe (Phyllo_Uni_Cy5; Table S2) with an attached Cy5 fluorochrome, which would appear as bright red spots if present. DAPI staining of bacterial DNA was used as a positive control in each experiment and it would appear as bright blue spots if there were any bacteria present. FISH images suggested that no bacteria were present.

Ap. dentigerum – ileum

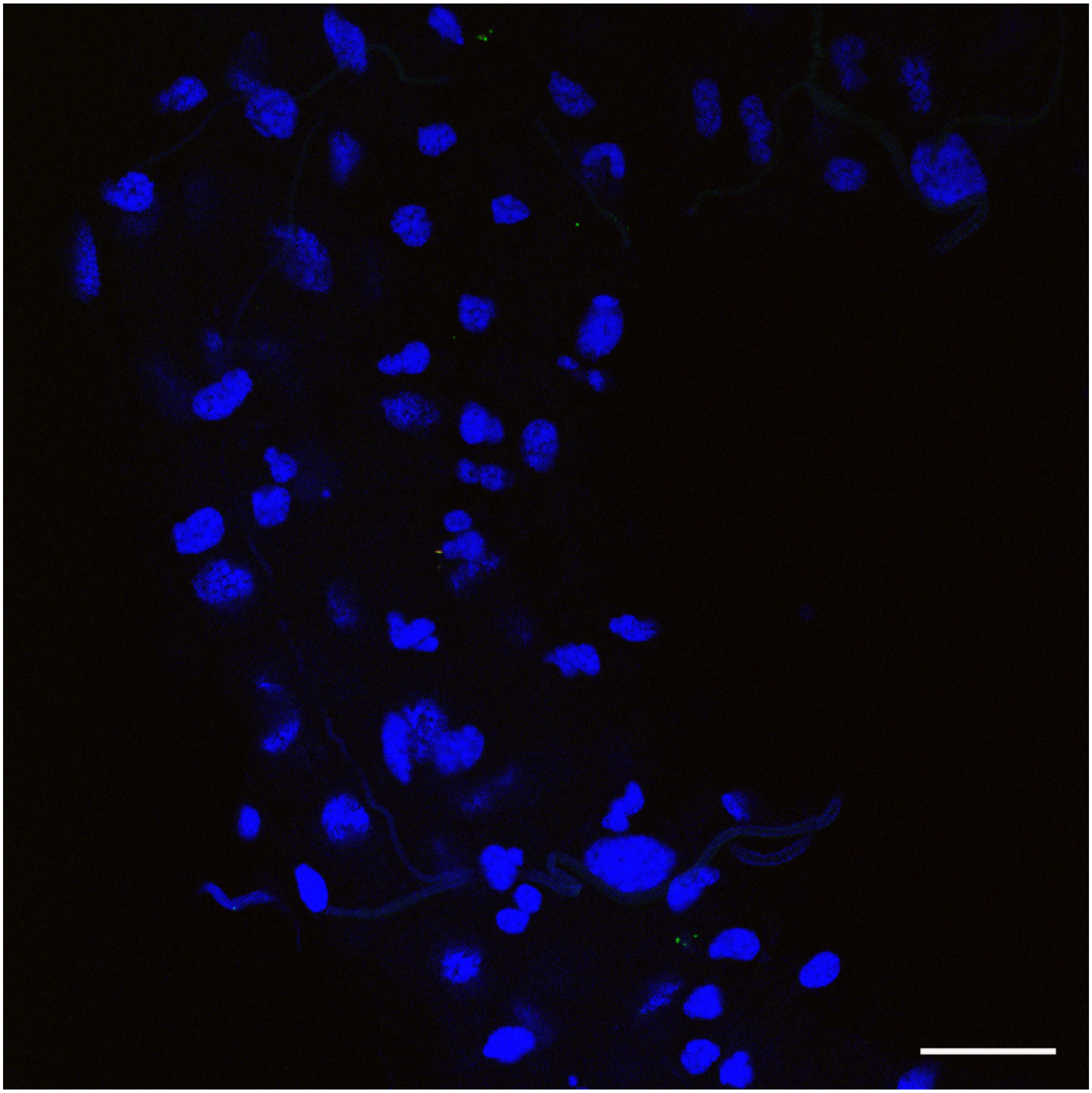

Figure SR2.28: Example of no bacteria detected in the ileum of *Ap. dentigerum* workers. Staining was performed with a 16S Mollicutes-specific probe (Entom_A488; Table S2) with an attached alexa488 fluorochrome, which would appear as bright green spots if present and an alpha-Proteobacteria-specific probe (Phyllo_Uni_Cy5; Table S2) with an attached Cy5 fluorochrome, which would appear as bright red spots if present. DAPI staining of bacterial DNA was used as a positive control in each experiment and it would appear as bright blue spots if there were any bacteria present. FISH images suggested that no bacteria were present.

Ap. dentigerum – rectum

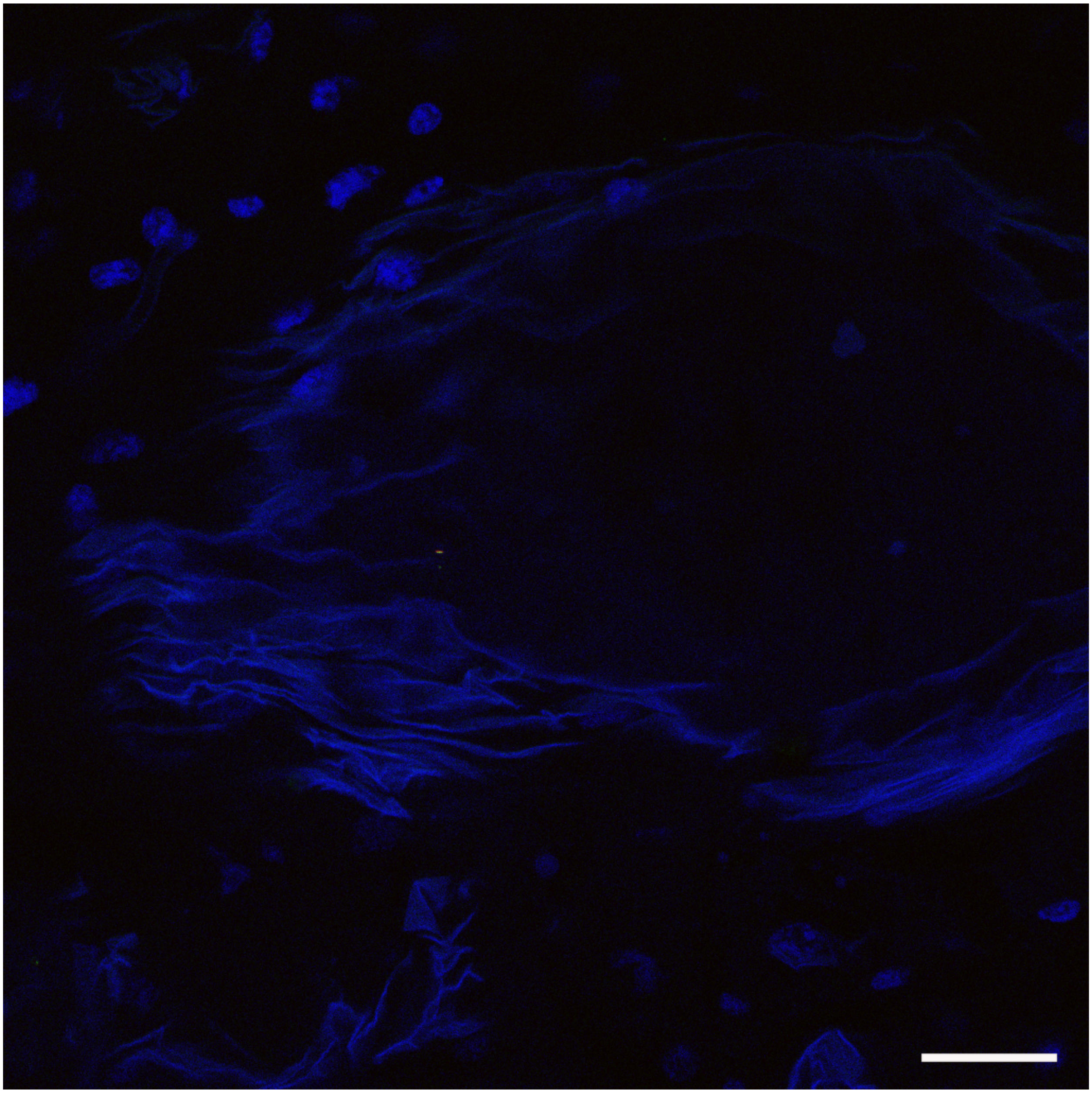

Figure SR2.29: Example of no bacteria detected in the rectum of *Ap. dentigerum* workers. Staining was performed with a 16S Mollicutes-specific probe (Entom_A488; Table S2) with an attached alexa488 fluorochrome (which would appear as bright green spots if present) and an alpha-Proteobacteria-specific probe (Phyllo_Uni_Cy5; Table S2) with an attached Cy5 fluorochrome (which would appear as bright red spots if present). DAPI staining of bacterial DNA was used as a positive control in each experiment and it would appear as bright blue spots if there were any bacteria present. FISH images suggested that no bacteria were present.
