# Supplementary material for: The evolution of abdominal microbiomes in fungus‐growing ants
Source: Mol Ecol. 2018 Dec 10;28(4):879–99. doi: 10.1111/mec.14931 (PMC6446810; doi:10.1111/mec.14931)
Supplement: Supplementary file 3 [file MEC-28-879-s003.docx]

**Supplementary Results 3 – Comparisons of absolute bacterial abundances in dissected and surface cleaned entire abdomens across eight attine ant species**

Because of the large size differences between workers of the attine ant species examined, it was necessary to treat large and small-bodied species in a different manner. For the main 16S MiSeq analysis, the dissected abdominal tissues (fat body cells, midgut, Malpighian tubules, ileum and rectum) of larger-bodied species (the genera *Atta*, *Acromyrmex*, *Sericomyrmex*, *Trachymyrmex* and *Apterostigma*) were dissected out, while for small bodied species (the genera *Cyphomyrmex*, *Mycocepurus* and *Myrmicocrypta*), entire surface cleaned abdomens were used. As a result, the samples from small-bodied ants may have included some bacteria present on the cuticle that survived our washes and potentially some other bacteria associated with glandular tissue, nervous tissue, and the (normally inactive) ovaries. Using different sampling procedures might thus have biased our results if these additional tissues harbored substantial numbers of bacteria not found in the intestinal tract and the associated organs that we dissected for the larger-bodies species.

While there is very little non-gut tissue present inside attine worker abdomens (i.e. gasters)(Dijkstra et al., 2005), a considerable part is made up by cuticular material. A recent study suggests that there are very few bacteria associated with cuticular material, at least in *Atta cephalotes* (Birer et al., 2017), but to examine the magnitude of potential bias directly, we compared dissected abdominal samples with entire abdomen samples. The analyses presented here thus examine both the potential bias introduced in our 16S-MiSeq sequencing by the presence of two types of tissues and complement the qPCR analyses given in Fig. 2 where we exclusively used data on whole abdomen homogenates to compare the overall bacterial abundances of 12 species. Working with two types of tissue homogenates implied that the number of host cells varied depending on extraction procedure, so that normalization with a host housekeeping gene such as EF-1α would not be appropriate. We therefore used non-normalized data here, assuming that there were no confounding effects due to the presence of tissue-specific PCR inhibitors, and directly compared the 16S ΔCt values across samples. We excluded one field sample of *Atta cephalotes* (Ace_f3) from the analysis because of poor amplification of both 16S and *EF-1α* (also identified as an outlier for both genes using Huber robust analysis).

A 2-way ANOVA was carried out using uncorrected fold-increases in 16S rRNA copies, relative to the most basal attine ant *Ap. dentigerum* as the dependent variable, and species and tissue type (dissected or entire abdomens) and their interaction term as independent variables.

The comparison confirmed (Figure SR3.1) that there was a highly significant difference in 16S bacterial titers between species (F_7,31_ = 10.69, p <0.0001), but that whole abdomens had only slightly higher bacterial titers than dissected abdomens, a difference that was not significant (F_1,31_ = 3.97, p = 0.055). This increase did not differ between species (species-tissue-type interaction: F_7,31_ = 1.06, p = 0.412) and a variance component decomposition analysis indicated that ant species accounted for 59.5% of the total variance, tissue type for 4.5% and the interaction term for 0.75%.

Hence, while there is some evidence that cuticular material and some other tissues in samples of entire abdomens before extraction increased the bacterial titers, this effect was minor compared to the differences in bacterial titers between the ant species. We therefore concluded that the practical necessity of using different extraction procedures for the larger and small- bodied attine species did not introduce an overall bias in our comparisons.


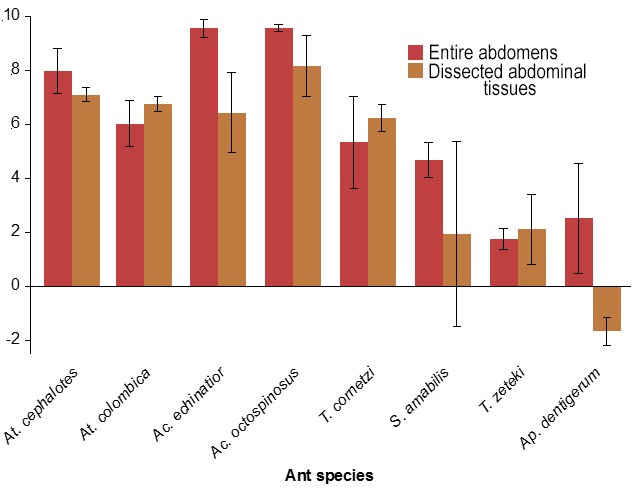
**Figure SR3.1: Comparisons of bacterial 16S rDNA copies for DNA samples extracted from pooled entire abdomens and from dissected tissues of the same ant species measured by Quantitative PCR.** Data were not normalized against ant housekeeping genes and results are presented as delta CT values calculated using the Pfaffl (2001) method. For each ant species three colony-level DNA samples were used, and all pools consisted of 5 individuals. Error bars are standard errors.

**Fold-change relative to *Ap. dentigerum***

**References**

Birer, C., Tysklind, N., Zinger, L., and Duplais, C. (2017). Comparative analysis of DNA extraction methods to study the body surface microbiota of insects: A case study with ant cuticular bacteria. *Mol. Ecol. Resour.* Online first.

Dijkstra, M.B., Nash, D.R., and Boomsma, J.J. (2005). Self-restraint and sterility in workers of *Acromyrmex* and *Atta* leafeutter ants. I*nsectes Sociaux* ***52***, 67–76.
