# Supplementary material for: The evolution of abdominal microbiomes in fungus‐growing ants
Source: Mol Ecol. 2018 Dec 10;28(4):879–99. doi: 10.1111/mec.14931 (PMC6446810; doi:10.1111/mec.14931)
Supplement: Supplementary file 4 [file MEC-28-879-s004.docx]

**Supporting Information Appendix**

***Supplementary figures***

**Fig. S1:** Rarefaction curves for each set of ant abdominal sample sequences.

**Fig. S2:** Highest and second-highest prevalences of bacterial OTUs across the 84 samples amplified with standard PCR based on the rarefied OTUs (Table S3).

**Fig. S3:** Heatmap showing the relative abundance (after rarefaction) of the 98 most abundant OTUs (OTU0001-OTU0100 based on Mothur’s relative abundance ranking) across 16 fungus-growing ant species based on 84 samples amplified with standard PCR (see Table S3 for details).

**Fig. S4:** Dated phylogeny (MYA) used for comparative analyses of changes in bacterial prevalences across the attine ant phylogeny based on four measures of alpha diversity and inequality, diversity of the abdominal bacterial communities associated with 16 attine ant species.

**Fig. S5**: Composition of abdominal bacterial communities (beta diversity) and their partitioning according to phylogeny and presence/absence of cuticular Actinobacteria across 16 species of Panamanian fungus-growing ants based on 84 colony-samples and all 2099 OTUs identified (after rarefaction).

***Supplementary tables***

**Table S1:** Sample IDs, sampling environments, colony IDs, types of sample, ant species, and accession numbers (Sequence Read Archives) of the samples included in this study.

**Table S2:** Probes used for FISH microscopy: probes names, targeted bacterial OTUs or taxa, types of fluorochrome labeling used, and nucleotide sequences.

**Table S3:** OTU tables for the MiSeq data deposited at dryad.

**Table S4:** OTU alpha diversity and inequality indices for the MiSeq data based on 84 samples.

**Table S5:** 16S-qPCR samples, their species and sampling characteristics, and the ΔCt values generated.

**Table S6:** Differential representation of bacterial classes and OTUs in the abdomens of fungus-growing ants with and without cuticular Actinobacteria and on either side of the phylogenetic ‘A’ transition, using the 84 samples generated with standard PCR producing 2099 OTUs after rarefaction.

**Table S7:** Results of applying Bayesian Markov chain Monte Carlo generalized linear mixed models to examine the effects of sampling environment and the presence/absence of cuticular Actinobacteria on the four measures of alpha diversity and inequality of the abdominal bacterial communities associated with 16 attine ant species (A-D) and a similar analysis for overall bacterial abundance (E).

**Table S8:** HOMOVAs examining differences in the overall variability of abdominal bacterial communities (beta- diversity variance) of Panamanian attine ants across the phylogenetic ‘A’ partition and the presence/absence of cuticular Actinobacteria categories, using either the 2099 OTUs (A) or the 18 abundant OTUs (B) in analyses based on three different distance methods (Bray-Curtis, weighted Unifrac, and unweighted Unifrac).

**Table S9:** Results of a linear Discriminant Analysis between attine species based on 2-dimensional NMDS scores of pairwise distances between samples, subdivided according to the quadrants in Figure 4.

***Supplementary results***

**Supplementary results 1**: Validation of assumptions that all samples were fully comparable for estimating diversity indices.

**Supplementary results 2**: Examples of bacteria detected in various abdominal tissues, the gut lumen and the fat bodies of attine ant workers using confocal laser scanning FISH microscopy. This file has also been deposited at dryad: [https://doi.org/10.5061/dryad.tj30d](https://webmail.ku.dk/OWA/redir.aspx?C=E5pW_XwGwXhtdILh4Du7unl2z_IZGOz2gXPB-EDnhYMujRvzty7WCA..&URL=https%3a%2f%2fdoi.org%2f10.5061%2fdryad.tj30d)

**Supplementary results 3**: Comparisons of bacteria in dissected and surface cleaned entire abdomens across eight Panamanian attine ant species**.**

**Fig. S1: Rarefaction curves for each set of ant abdominal sample sequences**. Curves represent the number of OTUs as a function of the number of sequences obtained from each of the 107 samples initially included while not discriminating between touchdown PCR and standard PCR amplifications, which yielded pseudoreplicate OTU datasets containing between 1 and 122,998 sequences with 1,000 iterations per pseudoreplicate. The inset log frequency histogram shows the completeness distribution for each sample, measured as the proportion of overall Shannon diversity estimated by extrapolation to an infinite number of sequences (Hsieh et al., 2016).

**
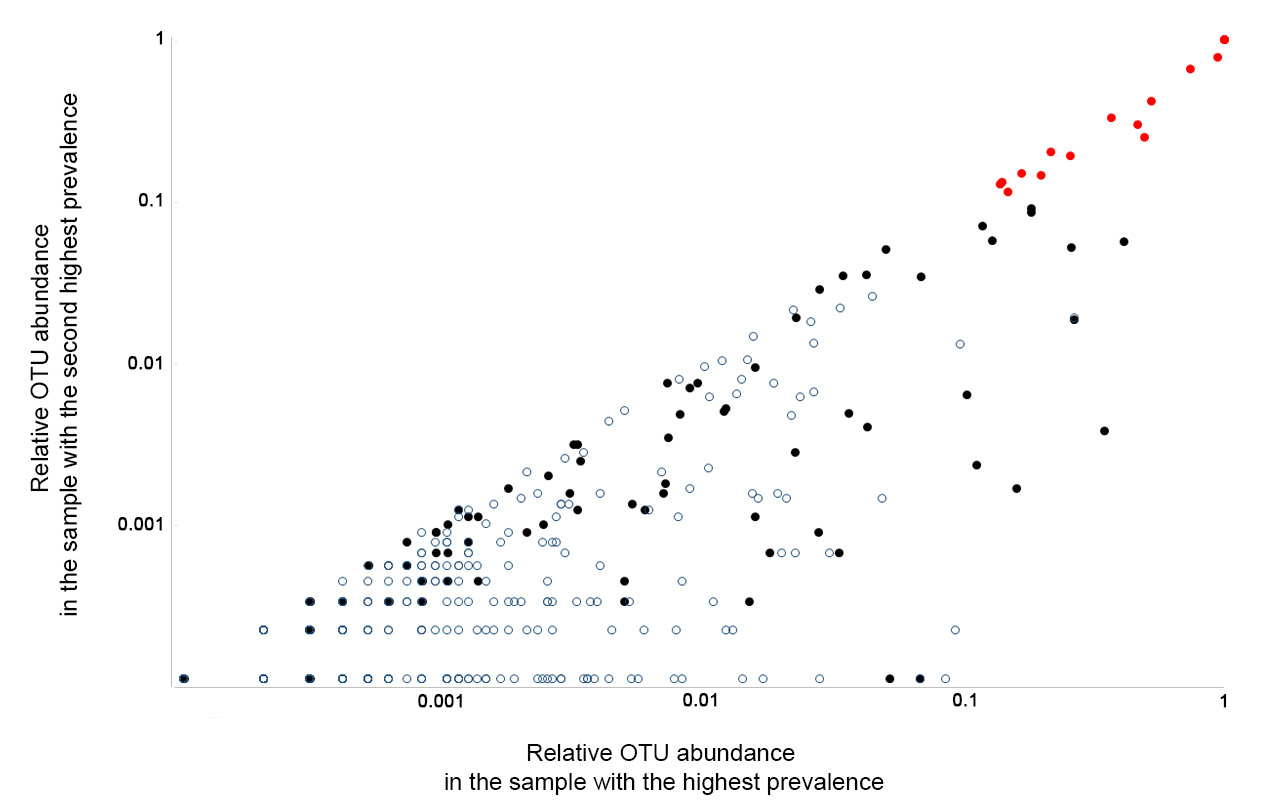
**

**Fig. S2: Highest and second-highest prevalences of bacterial OTUs across the 84 samples amplified with standard PCR based on the rarefied OTUs (Table S3).** The 18 abundant OTUs are shown in red, the remaining 80 OTUs (also presented as OTU0001-OTU0100 in Fig. S3) in black, and all remaining OTUs as open blue circles. OTUs *EntAcro1*, *WolAcro1*, *EntAcro10*, *RhiAcro1* and *Entom6* had relative abundances of 0.99 both on the X- and Y-axis and thus fall on top of each other. Only 67 black dots (instead of 80) and 136 open dots (instead of 2001) can be seen as many of them fell on top of each other as well.


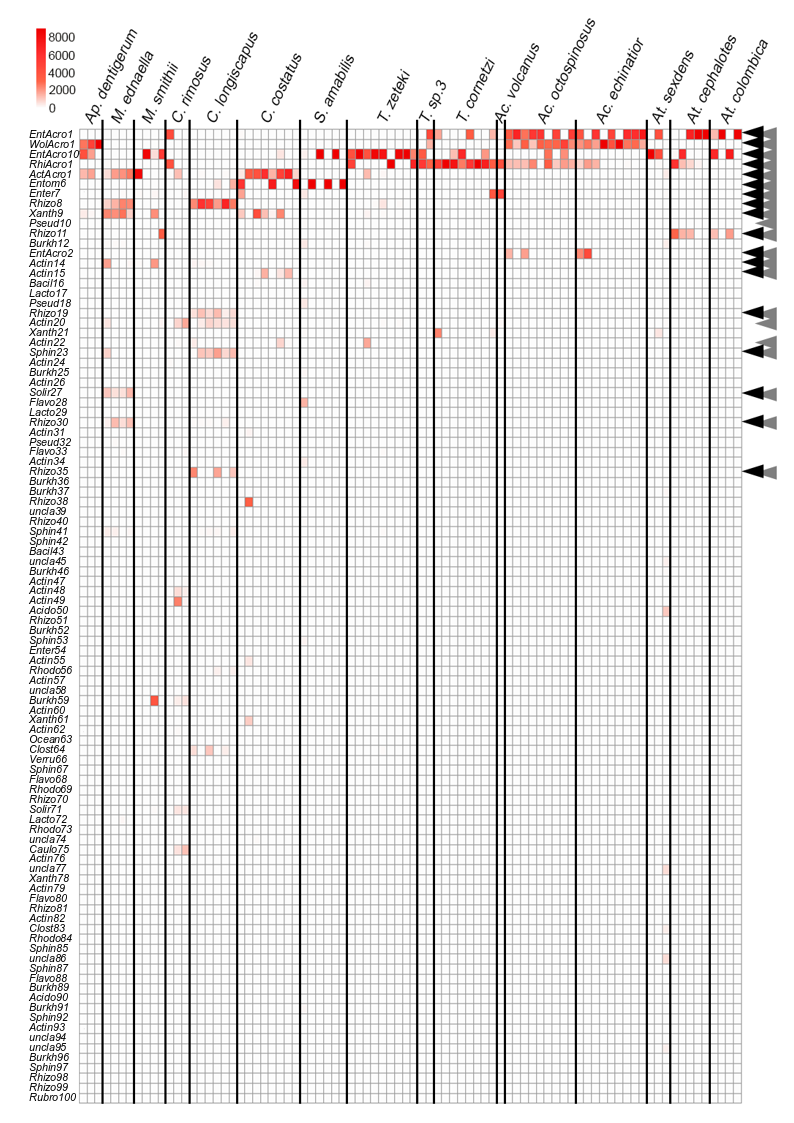
**Fig. S3: Heatmap showing the relative abundance (after rarefaction) of the 98 most abundant OTUs (OTU0001-OTU0100 based on Mothur’s relative abundance ranking) across 16 fungus-growing ant species based on 84 samples amplified with standard PCR (see Table S3 for details).** Samples consisted of five pooled dissected worker abdomens, except for *Cyphomyrmex*, *Mycocepurus* and *Myrmicocrypta* species where entire pooled abdomens (instead of dissected tissues) were used (see text for details). OTU names are presented on the left with the first five letters representing their bacterial order and an additional number identifying relative abundance rank. The 98 OTUs jointly accounted for more than 91% of the total rarefied reads and the 18 most abundant OTUs (marked with horizontal black arrows towards the right) accounted for more than 90% of the reads. When applying the same 10% prevalence criterion in at least two samples to all 107 samples (i.e. including also samples amplified with touchdown PCR) we identified 21 abundant OTUs, the same 18 and three additional ones (grey arrows). Solid vertical black lines separate the 16 ant species. Two of the first hundred OTUs (OTU0001-OTU0100) were eliminated at rarefaction, producing a final total of 98. The display includes 16 rather than 17 species because the single *Ap. collare* sample amplified with touchdown PCR was omitted.

**
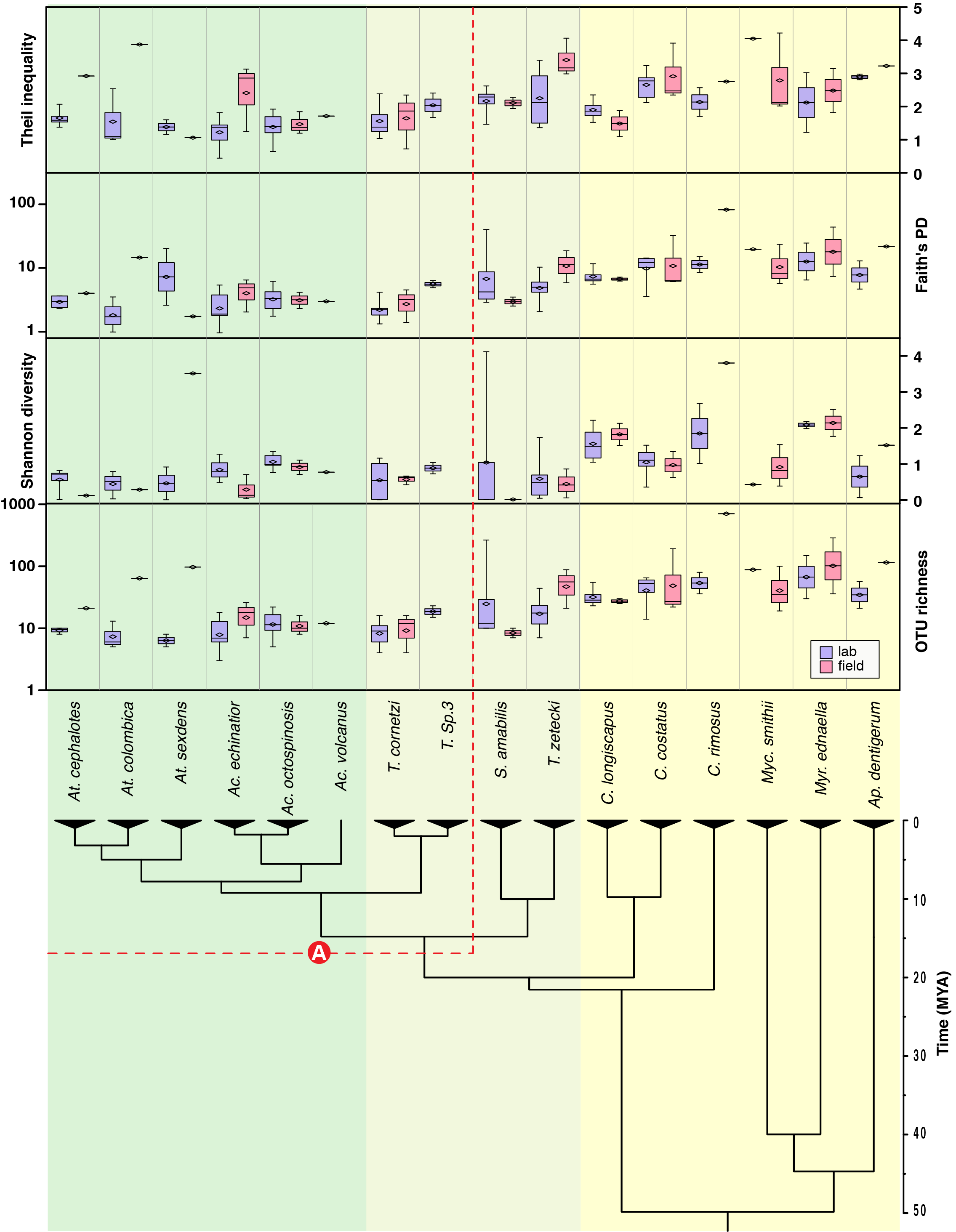
**

**Fig. S4: Dated phylogeny (MYA) used for comparative analyses of changes in bacterial prevalences across the attine ant phylogeny based on four measures of alpha diversity and inequality of the abdominal bacterial communities associated with 16 attine ant species.** Box-plots refer to OTU richness, Shannon Diversity, Faith’s phylogenetic diversity (PD), and Theil inequality separately for field and lab colonies. Central horizontal lines are medians, with the boxes extending from the lower to upper quartile, and the whiskers indicating the limits of the data. Mean values are marked with a diamond symbol. For phylogenetically controlled analyses, intraspecific variation was included by assuming that between-sample variation resulted from within-species polytomies 1MYA (black triangles; see methods for further details). As in Fig. 1, the known fungus-farming transitions are highlighted by background color shifts from yellow to light green to darker green. The red dashed lines mark the empirically obtained microbiome transition ‘A’ for two alpha diversity measures, which was also recovered in our analyses of bacterial titers (Fig. 2; Supplementary Results 3).

**
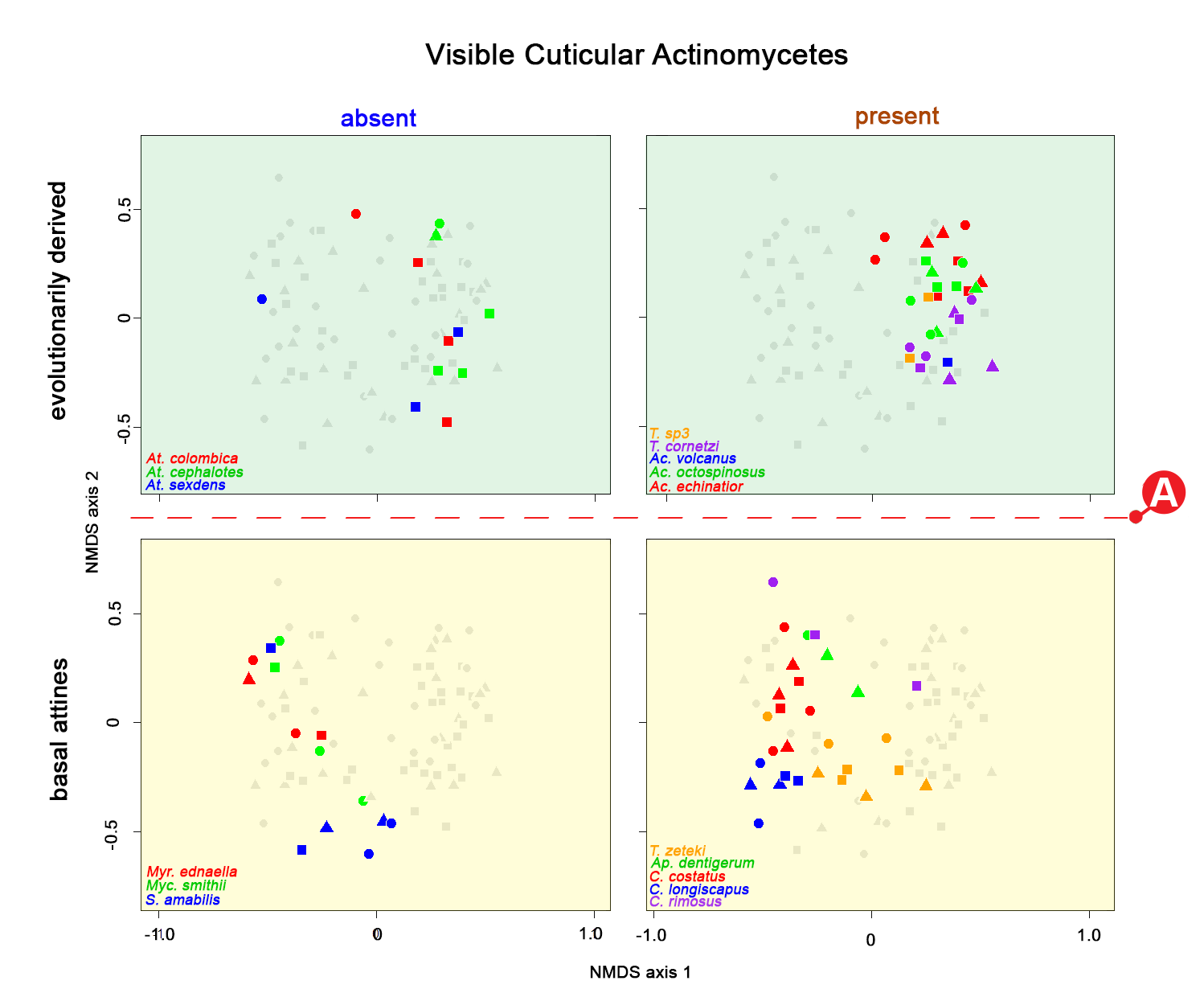
**

**Fig. S5: Composition of abdominal bacterial communities (beta diversity) and their partitioning according to phylogeny and presence/absence of cuticular Actinobacteria across 16 species of Panamanian fungus-growing ants based on 84 colony-samples and all 2099 OTUs identified (after rarefaction).** Layout is identical to Fig. 4, but the ordination resulted in mirror images of the plots presented there. The analysis is based on Non-Metric Multidimensional Scaling ordination of pairwise Bray-Curtis dissimilarities between all 84 samples amplified with standard PCR, and had an overall stress-factor of 0.2545. The three types of samples for each ant species were: field colonies (circles), short-term lab colonies (triangles), and long-term lab colonies (squares). The four panels each have all 84 colony samples plotted, with samples from the focal (quadrant-specific) ant colored by ant species, and the remaining samples in grey. HOMOVA results comparing the variances for bacterial community composition among samples across the two contrasts are given in table S8. Results of matching quadrant-specific linear discriminant analyses between ant species are given in table S9. Both support the results found for the 18 dominant OTUs (Figure 4) showing that OTU community variability and discrimination between attine species is lowest for the evolutionarily derived attines with visible cuticular Actinobacteria (see text for further details).

**Table S1: Sample IDs, sampling environments, colony IDs, types of sample, ant species, and accession numbers (Sequence Read Archives) of the samples included in this study.** Dagger symbols (†) next to sample IDs mark library construction amplifications with touchdown PCR, which were removed from most of the analyses (see text), while the remaining samples were amplified by standard PCR (see methods for details). Asterisks next to colony IDs mark samples from colonies that were used for FISH microscopy. The § symbols indicate samples that were used for Real-Time qPCR and §§ symbols indicate additional samples that were prepared from pooled abdomens of ant workers and used for Real-Time qPCR in an independent analysis to validate bacterial abundances (Supplementary Results 3) showing that evolutionarily derived higher attine ants have more 16S rRNA copies in their guts and associated organs than the more basal attine ants. Similar information on the *Acromyrmex* samples included in our study is given by Sapountzis et al. (2015).

| **Sample ID** | ***Sampling Environment*** | **Colony ID** | **Type / Sample** | **Ant species** | **SRA** |
| --- | --- | --- | --- | --- | --- |
| **Ac_3m2 †** | short-term lab | Ac2 * | 16S-MiSeq sequencing from five pooled dissected abdomens of an *At. colombica* lab colony | *Atta colombica* | SAMN03460111 |
| **Ac_f1 †** | field | Ac1 | 16S-MiSeq sequencing from five pooled dissected abdomens of an *At. colombica* field colony | *Atta colombica* | SAMN03460107 |
| **Ac_f2 †** | field | Ac2 §§ | 16S-MiSeq sequencing from five pooled dissected abdomens of an *At. colombica* field colony | *Atta colombica* | SAMN03460108 |
| **Ac_f3** | field | Ac3 | 16S-MiSeq sequencing from five pooled dissected abdomens of an *At. colombica* field colony | *Atta colombica* | SAMN03460112 |
| **Ac_l1** | long-term lab | Ac4 § §§ | 16S-MiSeq sequencing from five pooled dissected abdomens of an *At. colombica* lab colony | *Atta colombica* | SAMN03460106 |
| **Ac_l2** | long-term lab | Ac5 § | 16S-MiSeq sequencing from five pooled dissected abdomens of an *At. colombica* lab colony | *Atta colombica* | SAMN03460109 |
| **Ac_l3** | long-term lab | Ac6 § | 16S-MiSeq sequencing from five pooled dissected abdomens of an *At. colombica* lab colony | *Atta colombica* | SAMN03460110 |
| **Ace_3m2** | short-term lab | Ace2 | 16S-MiSeq sequencing from five pooled dissected abdomens of an *At. cephalotes* lab colony | *Atta cephalotes* | SAMN03460098 |
| **Ace_3m3 †** | short-term lab | Ace3 §§ | 16S-MiSeq sequencing from five pooled dissected abdomens of an *At. cephalotes* lab colony | *Atta cephalotes* | SAMN03460103 |
| **Ace_R1** | field | Ace1 | 16S-MiSeq sequencing from five pooled dissected abdomens of an *At. cephalotes* field colony | *Atta cephalotes* | SAMN03460104 |
| **Ace_f2 †** | field | Ace2 | 16S-MiSeq sequencing from five pooled dissected abdomens of an *At. cephalotes* field colony | *Atta cephalotes* | SAMN03460102 |
| **Ace_f3 †** | field | Ace3 § | 16S-MiSeq sequencing from five pooled dissected abdomens of an *At. cephalotes* field colony | *Atta cephalotes* | SAMN03460099 |
| **Ace_l1** | long-term lab | Ace4 * § | 16S-MiSeq sequencing from five pooled dissected abdomens of an *At. cephalotes* lab colony | *Atta cephalotes* | SAMN03460100 |
| **Ace_l2** | long-term lab | Ace5 § | 16S-MiSeq sequencing from five pooled dissected abdomens of an *At. cephalotes* lab colony | *Atta cephalotes* | SAMN03460101 |
| **Ace_l3** | long-term lab | Ace6 * | 16S-MiSeq sequencing from five pooled dissected abdomens of an *At. cephalotes* lab colony | *Atta cephalotes* | SAMN03460105 |
| **Acol_f1 †** | field | Acol1 | 16S-MiSeq sequencing from five pooled dissected abdomens of an *Ap. cf collare* field colony | *Apterostigma cf. collare* | SAMN03460090 |
| **Adent_3m1 †** | short-term lab | Adent1 § | 16S-MiSeq sequencing from five pooled dissected abdomens of an *Ap. dentigerum* lab colony | *Apterostigma dentigerum* | SAMN03460094 |
| **Adent_3m2** | short-term lab | Adent2 §* | 16S-MiSeq sequencing from five pooled dissected abdomens of an *Ap. dentigerum* lab colony | *Apterostigma dentigerum* | SAMN03460095 |
| **Adent_3m3** | short-term lab | Adent3 | 16S-MiSeq sequencing from five pooled dissected abdomens of an *Ap. dentigerum* lab colony | *Apterostigma dentigerum* | SAMN03460096 |
| **Adent_f1 †** | field | Adent1 § | 16S-MiSeq sequencing from five pooled dissected abdomens of an *Ap. dentigerum* field colony | *Apterostigma dentigerum* | SAMN03460091 |
| **Adent_f2 †** | field | Adent2 | 16S-MiSeq sequencing from five pooled dissected abdomens of an *Ap. dentigerum* field colony | *Apterostigma dentigerum* | SAMN03460092 |
| **Adent_f3** | field | Adent3 | 16S-MiSeq sequencing from five pooled dissected abdomens of an *Ap. dentigerum* field colony | *Apterostigma dentigerum* | SAMN03460093 |
| **Adent_l1 †** | long-term lab | Adent4 * | 16S-MiSeq sequencing from five pooled dissected abdomens of an *Ap. dentigerum* lab colony | *Apterostigma dentigerum* | SAMN03460097 |
| **As_R1** | field | As3 | 16S-MiSeq sequencing from five pooled dissected abdomens of an *At. sexdens* field colony | *Atta sexdens* | SAMN03460114 |
| **As_f1 †** | field | As1 | 16S-MiSeq sequencing from five pooled dissected abdomens of an *At. sexdens* field colony | *Atta sexdens* | SAMN03460116 |
| **As_f2 †** | field | As2 | 16S-MiSeq sequencing from five pooled dissected abdomens of an *At. sexdens* field colony | *Atta sexdens* | SAMN03460113 |
| **As_l1** | long-term lab | As4 | 16S-MiSeq sequencing from five pooled dissected abdomens of an *At. sexdens* lab colony | *Atta sexdens* | SAMN03460115 |
| **As_l2** | long-term lab | As5 | 16S-MiSeq sequencing from five pooled dissected abdomens of an *At. sexdens* lab colony | *Atta sexdens* | SAMN03460117 |
| **Tra_l1** | long-term lab | Tra1 | 16S-MiSeq sequencing from five pooled dissected abdomens of a *T. sp. 3* lab colony | *Trachymyrmex sp. 3* | SAMN03460089 |
| **Ccost_3m1** | short-term lab | Ccost1 § | 16S-MiSeq sequencing from fifteen pooled abdomens of a C. *costatus* lab colony | *Cyphomyrmex costatus* | SAMN03460123 |
| **Ccost_3m2** | short-term lab | Ccost2 * | 16S-MiSeq sequencing from fifteen pooled abdomens of a C. *costatus* lab colony | *Cyphomyrmex costatus* | SAMN03460122 |
| **Ccost_3m3** | short-term lab | Ccost3 | 16S-MiSeq sequencing from fifteen pooled abdomens of a C. *costatus* lab colony | *Cyphomyrmex costatus* | SAMN03460125 |
| **Ccost_f1** | field | Ccost1 | 16S-MiSeq sequencing from fifteen pooled abdomens of a C. *costatus* field colony | *Cyphomyrmex costatus* | SAMN03460119 |
| **Ccost_f2** | field | Ccost2 § | 16S-MiSeq sequencing from fifteen pooled abdomens of a C. *costatus* field colony | *Cyphomyrmex costatus* | SAMN03460120 |
| **Ccost_f3** | field | Ccost3 | 16S-MiSeq sequencing from fifteen pooled abdomens of a C. *costatus* field colony | *Cyphomyrmex costatus* | SAMN03460124 |
| **Ccost_l1** | long-term lab | Ccost4 | 16S-MiSeq sequencing from fifteen pooled abdomens of a C. *costatus* lab colony | *Cyphomyrmex costatus* | SAMN03460121 |
| **Ccost_l2 †** | long-term lab | Ccost5 | 16S-MiSeq sequencing from fifteen pooled abdomens of a C. *costatus* lab colony | *Cyphomyrmex costatus* | SAMN03460126 |
| **Ccost_l3** | long-term lab | Ccost6 §* | 16S-MiSeq sequencing from fifteen pooled abdomens of a C. *costatus* lab colony | *Cyphomyrmex costatus* | SAMN03460118 |
| **Clong_3m1** | short-term lab | Clong1 § * | 16S-MiSeq sequencing from fifteen pooled abdomens of a C. *longiscapus* lab colony | *Cyphomyrmex longiscapus* | SAMN03460131 |
| **Clong_3m2** | short-term lab | Clong2 § | 16S-MiSeq sequencing from fifteen pooled abdomens of a C. *longiscapus* lab colony | *Cyphomyrmex longiscapus* | SAMN03460132 |
| **Clong_f1** | field | Clong1 § | 16S-MiSeq sequencing from fifteen pooled abdomens of a C. *longiscapus* field colony | *Cyphomyrmex longiscapus* | SAMN03460127 |
| **Clong_f2** | field | Clong2 | 16S-MiSeq sequencing from fifteen pooled abdomens of a C. *longiscapus* field colony | *Cyphomyrmex longiscapus* | SAMN03460128 |
| **Clong_l1** | long-term lab | Clong3 | 16S-MiSeq sequencing from fifteen pooled abdomens of a C. *longiscapus* lab colony | *Cyphomyrmex longiscapus* | SAMN03460129 |
| **Clong_l2** | long-term lab | Clong4 | 16S-MiSeq sequencing from fifteen pooled abdomens of a C. *longiscapus* lab colony | *Cyphomyrmex longiscapus* | SAMN03460130 |
| **Cri_f1** | field | Cri1 | 16S-MiSeq sequencing from fifteen pooled abdomens of a C. *rimosus* field colony | *Cyphomyrmex rimosus* | SAMN03460133 |
| **Cri_l1** | long-term lab | Cri2 | 16S-MiSeq sequencing from fifteen pooled abdomens of a C. *rimosus* lab colony | *Cyphomyrmex rimosus* | SAMN03460134 |
| **Cri_l2** | long-term lab | Cri3 | 16S-MiSeq sequencing from fifteen pooled abdomens of a C. *rimosus* lab colony | *Cyphomyrmex rimosus* | SAMN03460135 |
| **Medn_3m1 †** | short-term lab | Medn1 §* | 16S-MiSeq sequencing from ten pooled abdomens of a My*r. ednaella* lab colony | *Myrmicocrypta ednaella* | SAMN03460147 |
| **Medn_3m3** | short-term lab | Medn3 * | 16S-MiSeq sequencing from ten pooled abdomens of a My*r. ednaella* lab colony | *Myrmicocrypta ednaella* | SAMN03460148 |
| **Medn_f1** | field | Medn1 § | 16S-MiSeq sequencing from ten pooled abdomens of a My*r. ednaella* field colony | *Myrmicocrypta ednaella* | SAMN03460144 |
| **Medn_f3** | field | Medn3 | 16S-MiSeq sequencing from ten pooled abdomens of a My*r. ednaella* field colony | *Myrmicocrypta ednaella* | SAMN03460146 |
| **Medn_l1** | long-term lab | Medn4 | 16S-MiSeq sequencing from ten pooled abdomens of a My*r. ednaella* lab colony | *Myrmicocrypta ednaella* | SAMN03460145 |
| **Medn_l2 †** | long-term lab | Medn5 § | 16S-MiSeq sequencing from ten pooled abdomens of a My*r. ednaella* lab colony | *Myrmicocrypta ednaella* | SAMN03460149 |
| **Msmi_3m1 †** | short-term lab | Msmi1 §* | 16S-MiSeq sequencing from five pooled abdomens of a My*c. smithii* lab colony | *Mycocepurus smithii* | SAMN03460140 |
| **Msmi_3m2 †** | short-term lab | Msmi2 * | 16S-MiSeq sequencing from five pooled abdomens of a My*c. smithii* lab colony | *Mycocepurus smithii* | SAMN03460141 |
| **Msmi_3m3 †** | short-term lab | Msmi3 | 16S-MiSeq sequencing from five pooled abdomens of a My*c. smithii* lab colony | *Mycocepurus smithii* | SAMN03460142 |
| **Msmi_f1** | field | Msmi1 | 16S-MiSeq sequencing from five pooled abdomens of a My*c. smithii* field colony | *Mycocepurus smithii* | SAMN03460137 |
| **Msmi_f2** | field | Msmi2 | 16S-MiSeq sequencing from five pooled abdomens of a My*c. smithii* field colony | *Mycocepurus smithii* | SAMN03460138 |
| **Msmi_f3** | field | Msmi3 § | 16S-MiSeq sequencing from five pooled abdomens of a My*c. smithii* field colony | *Mycocepurus smithii* | SAMN03460143 |
| **Msmi_l1 †** | long-term lab | Msmi4 * | 16S-MiSeq sequencing from five pooled abdomens of a My*c. smithii* lab colony | *Mycocepurus smithii* | SAMN03460139 |
| **Msmi_l2** | long-term lab | Msmi5 §* | 16S-MiSeq sequencing from five pooled abdomens of a My*c. smithii* lab colony | *Mycocepurus smithii* | SAMN03460136 |
| **Sam_3m1** | short-term lab | Sam1 § | 16S-MiSeq sequencing from five pooled dissected abdomens of a S. *amabilis* lab colony | *Sericomyrmex amabilis* | SAMN03460155 |
| **Sam_3m2** | short-term lab | Sam2 | 16S-MiSeq sequencing from five pooled dissected abdomens of a S. *amabilis* lab colony | *Sericomyrmex amabilis* | SAMN03460156 |
| **Sam_3m3 †** | short-term lab | Sam3 | 16S-MiSeq sequencing from five pooled dissected abdomens of a S. *amabilis* lab colony | *Sericomyrmex amabilis* | SAMN03460157 |
| **Sam_f1** | field | Sam1 § | 16S-MiSeq sequencing from five pooled dissected abdomens of a S. *amabilis* field colony | *Sericomyrmex amabilis* | SAMN03460151 |
| **Sam_f2** | field | Sam2 | 16S-MiSeq sequencing from five pooled dissected abdomens of a S. *amabilis* field colony | *Sericomyrmex amabilis* | SAMN03460152 |
| **Sam_f3 †** | field | Sam3 | 16S-MiSeq sequencing from five pooled dissected abdomens of a S. *amabilis* field colony | *Sericomyrmex amabilis* | SAMN03460150 |
| **Sam_l1** | long-term lab | Sam4 | 16S-MiSeq sequencing from five pooled dissected abdomens of a S. *amabilis* lab colony | *Sericomyrmex amabilis* | SAMN03460153 |
| **Sam_l2 †** | long-term lab | Sam5 § * | 16S-MiSeq sequencing from five pooled dissected abdomens of a S. *amabilis* lab colony | *Sericomyrmex amabilis* | SAMN03460154 |
| **Sam_l3** | long-term lab | Sam6 * | 16S-MiSeq sequencing from five pooled dissected abdomens of a S. *amabilis* lab colony | *Sericomyrmex amabilis* | SAMN03460158 |
| **Tcorn_3m1** | short-term lab | Tcorn1 * | 16S-MiSeq sequencing from five pooled dissected abdomens of a T. *cornetzi* lab colony | *Trachymyrmex cornetzi* | SAMN03460164 |
| **Tcorn_3m2** | short-term lab | Tcorn2 § | 16S-MiSeq sequencing from five pooled dissected abdomens of a T. *cornetzi* lab colony | *Trachymyrmex cornetzi* | SAMN03460165 |
| **Tcorn_3m3** | short-term lab | Tcorn3 | 16S-MiSeq sequencing from five pooled dissected abdomens of a T. *cornetzi* lab colony | *Trachymyrmex cornetzi* | SAMN03460166 |
| **Tcorn_f1** | field | Tcorn1 § | 16S-MiSeq sequencing from five pooled dissected abdomens of a T. *cornetzi* field colony | *Trachymyrmex cornetzi* | SAMN03460160 |
| **Tcorn_f2** | field | Tcorn2 § | 16S-MiSeq sequencing from five pooled dissected abdomens of a T. *cornetzi* field colony | *Trachymyrmex cornetzi* | SAMN03460161 |
| **Tcorn_f3** | field | Tcorn3 | 16S-MiSeq sequencing from five pooled dissected abdomens of a T. *cornetzi* field colony | *Trachymyrmex cornetzi* | SAMN03460163 |
| **Tcorn_l1** | long-term lab | Tcorn4 §§ | 16S-MiSeq sequencing from five pooled dissected abdomens of a T. *cornetzi* lab colony | *Trachymyrmex cornetzi* | SAMN03460162 |
| **Tcorn_l2** | long-term lab | Tcorn5 | 16S-MiSeq sequencing from five pooled dissected abdomens of a T. *cornetzi* lab colony | *Trachymyrmex cornetzi* | SAMN03460159 |
| **Tra_l2** | long-term lab | Tra2 | 16S-MiSeq sequencing from five pooled dissected abdomens of a *T. sp. 3* lab colony | *Trachymyrmex sp. 3* | SAMN03460167 |
| **Tz_3m1** | short-term lab | Tz1 § | 16S-MiSeq sequencing from five pooled dissected abdomens of a T. *zeteki* lab colony | *Trachymyrmex zeteki* | SAMN03460175 |
| **Tz_3m2** | short-term lab | Tz2 | 16S-MiSeq sequencing from five pooled dissected abdomens of a T. *zeteki* lab colony | *Trachymyrmex zeteki* | SAMN03460174 |
| **Tz_3m3** | short-term lab | Tz3 § | 16S-MiSeq sequencing from five pooled dissected abdomens of a T. *zeteki* lab colony | *Trachymyrmex zeteki* | SAMN03460176 |
| **Tz_f1** | field | Tz1 | 16S-MiSeq sequencing from five pooled dissected abdomens of a T. *zeteki* field colony | *Trachymyrmex zeteki* | SAMN03460170 |
| **Tz_f2** | field | Tz2 | 16S-MiSeq sequencing from five pooled dissected abdomens of a T. *zeteki* field colony | *Trachymyrmex zeteki* | SAMN03460171 |
| **Tz_f3** | field | Tz3 § | 16S-MiSeq sequencing from five pooled dissected abdomens of a T. *zeteki* field colony | *Trachymyrmex zeteki* | SAMN03460173 |
| **Tz_l1** | long-term lab | Tz4 * §§ | 16S-MiSeq sequencing from five pooled dissected abdomens of a T. *zeteki* lab colony | *Trachymyrmex zeteki* | SAMN03460177 |
| **Tz_l2** | long-term lab | Tz5 §§ | 16S-MiSeq sequencing from five pooled dissected abdomens of a T. *zeteki* lab colony | *Trachymyrmex zeteki* | SAMN03460172 |
| **Tz_l3** | long-term lab | Tz6 | 16S-MiSeq sequencing from five pooled dissected abdomens of a T. *zeteki* lab colony | *Trachymyrmex zeteki* | SAMN03460169 |
| **Ac-2012-1** | long-term lab | Ac4 §§ | additional qPCR using DNA from five pooled abdomens of an *At. colombica*  lab colony | *Atta colombica* | NA |
| **Ace-2012-2** | long-term lab | Ace4 §§ | additional qPCR using DNA from five pooled abdomens of an *At. cephalotes*  lab colony | *Atta cephalotes* | NA |
| **Ace-2012-4** | long-term lab | Ace4 §§ | additional qPCR using DNA from five pooled abdomens of an *At. cephalotes*  lab colony | *Atta cephalotes* | NA |
| **Ae150** | long-term lab | NA §§ | additional qPCR using DNA from five pooled abdomens of an *Ac.echinatior*  lab colony | *Acromyrmex echinatior* | NA |
| **Ae484** | long-term lab | NA §§ | additional qPCR using DNA from five pooled abdomens of an *Ac.echinatior*  lab colony | *Acromyrmex echinatior* | NA |
| **Ae331** | long-term lab | Aech_l1 §§ | additional qPCR using DNA from five pooled abdomens of an *Ac.echinatior*  lab colony | *Acromyrmex echinatior* | NA |
| **Ao482** | long-term lab | NA §§ | additional qPCR using DNA from five pooled abdomens of an *Ac.octospinosus*  lab colony | *Acromyrmex octospinosus* | NA |
| **Ao616** | long-term lab | Aoct_3m3 §§ | additional qPCR using DNA from five pooled abdomens of an *Ac.octospinosus*  lab colony | *Acromyrmex octospinosus* | NA |
| **Ao492** | long-term lab | Aoct_l1 §§ | additional qPCR using DNA from five pooled abdomens of an *Ac.octospinosus*  lab colony | *Acromyrmex octospinosus* | NA |
| **177613** | long-term lab | NA §§ | additional qPCR using DNA from five pooled abdomens of a *T.cornetzi*  lab colony | *Trachymyrmex cornetzi* | NA |
| **May-15** | long-term lab | NA §§ | additional qPCR using DNA from five pooled abdomens of a *T.cornetzi*  lab colony | *Trachymyrmex cornetzi* | NA |
| **TMI140519-13** | long-term lab | NA §§ | additional qPCR using DNA from five pooled abdomens of a *S.amabilis*  lab colony | *Sericomyrmex amabilis* | NA |
| **J130504-04** | long-term lab | NA §§ | additional qPCR using DNA from five pooled abdomens of a *S.amabilis*  lab colony | *Sericomyrmex amabilis* | NA |
| **140515-01** | long-term lab | NA §§ | additional qPCR using DNA from five pooled abdomens of a *T.zeteki*  lab colony | *Trachymyrmex zeteki* | NA |
| **190523-07** | long-term lab | NA §§ | additional qPCR using DNA from five pooled abdomens of an *Ap.dentigerum*  lab colony | *Apterostigma dentigerum* | NA |
| **150517-03** | long-term lab | NA §§ | additional qPCR using DNA from five pooled abdomens of an *Ap.dentigerum*  lab colony | *Apterostigma dentigerum* | NA |

**Table S2: Probes used for FISH microscopy: probes names, targeted bacterial OTUs or taxa, type of fluorochrome labeling used, and nucleotide sequences.** The * symbols indicate probes that were designed in a previous study (Sapountzis *et al.* 2015).

| \| Name \| Target \| Specificity \| Fluorochrome \| Sequence 5’ to 3’ \| \| --- \| --- \| --- \| --- \| --- \| \| Entom_A * \| *EntAcro1* \| OTU specific \| Cy3 \| CTG TCA CAT TCT AGT CGT AT \| \| Entom_B * \| *EntAcro2* \| OTU specific \| Cy3 \| TCT CTT GTA TTC TAG TGA TGT \| \| Phyllo_R * \| *RhiAcro1* \| OTU specific \| Cy3 \| CTT CTG CAC TCA AGA TAA AC \| \| Phyllo_Cy5 * \| *RhiAcro1* \| OTU specific \| Cy5 \| CTT CTG CAC TCA AGA TAA AC \| \| Xantho2 \| *Xanth9, Xanth21, Pseud18* \| OTU specific \| Cy5 \| ATC CAA TGC AAT TCC CAG \| \| Propio \| *Actin15* \| OTU specific \| Cy3 \| GGT AAT GCC CAG AGA AC \| \| Chitino \| *Sphin23* \| OTU specific \| Cy3 \| ATG ACA TAT TCC GCT AAC \| \| Actino \| *ActAcro1, Actin14, Actin20* \| OTU specific \| Cy3 \| GTA TCG GCC CAG AGA C \| \| Entero-pseudo \| *Enter7, Pseud10* \| OTU specific \| Cy5 \| TTT TGA ATG CAG TTC CCA G \| \| W2 * \| *WolAcro1* \| OTU specific \| Cy3 \| CTT CTG TGA GTA CCG TCA TTA TC \| \| Wolbachia16SR * \| *WolAcro1* \| OTU specific \| Cy3 \| CAG ATT TGA ACC AGA TAG A \| \| Phylo_220 \| *Rhizo11* \| OTU specific \| Cy3 \| CTT CTG TAC TCT AGA TAC CC \| \| Entom_A488 * \| Mollicutes  (*EntAcro1*, *EntAcro2*,  *EntAcro10, Entom6*) \| class specific \| Alexa488 \| TGT TGT AAG GGA AGA A \| \| Phyllo_Uni_Cy5 * \| α-Proteobacteria (*RhiAcro1*, *WolAcro1,  Rhizo8, Rhizo11*) \| class specific \| Cy5 \| CCG GTG AAG ATA ATG ACG GTA \| \| non-Entom488 \| - \| antisense,  non-specific \| Alexa488 \| TTC TTC CCT TAC AAC AGA C \| \| non-EntomA * \| - \| antisense,  non-specific \| Cy3 \| TAT GCT GAT CTT ACA CTG TC \| \| non-Phyllo_R * \| - \| antisense,  non-specific \| Cy5 \| CAA ATA GAA CTC ACG TCT TC \| |  |  |  |
| --- | --- | --- | --- | --- | --- | --- | --- | --- | --- | --- | --- | --- | --- | --- | --- | --- | --- | --- | --- | --- | --- | --- | --- | --- | --- | --- | --- | --- | --- | --- | --- | --- | --- | --- | --- | --- | --- | --- | --- | --- | --- | --- | --- | --- | --- | --- | --- | --- | --- | --- | --- | --- | --- | --- | --- | --- | --- | --- | --- | --- | --- | --- | --- | --- | --- | --- | --- | --- | --- | --- | --- | --- | --- | --- | --- | --- | --- | --- | --- | --- | --- | --- | --- | --- | --- | --- | --- | --- | --- | --- | --- | --- | --- |

**Table S3: OTU tables for the MiSeq data deposited at dryad:** [https://doi.org/10.5061/dryad.tj30d](https://webmail.ku.dk/OWA/redir.aspx?C=E5pW_XwGwXhtdILh4Du7unl2z_IZGOz2gXPB-EDnhYMujRvzty7WCA..&URL=https%3a%2f%2fdoi.org%2f10.5061%2fdryad.tj30d)

**File 1: OTU97 table with pre-filtration blanks_included.** **A**: Table showing the 3334 OTUs identified in MiSeq analysis of the original 107 samples analyzed by clustering 97% identical sequences in Mothur before filtering. **B**: The same table rarefied at 8900 reads. **C**: Table showing the 65 OTUs identified in the three ‘blank’/water samples included in our MiSeq sequencing. To identify common OTUs between ‘blank’ and ant samples we used local blast analyses and for the OTUs that were present in both ant and ‘blank’ samples we provide the corresponding OTU numbers (IDs) in the ant sample table at the bottom of the table.

**File 2: OTU97 table main ms filteredOTU97. A**: Table showing the 2099 OTUs identified in the MiSeq analysis of the original 107 samples analyzed by clustering 97% identical sequences in Mothur rarefied at 8900 reads after removing potential water contaminants. We identified the OTUs (97% clustering) present in the blank ‘water’ samples that were sequenced along with the real samples, and removed these OTUs if their relative abundances produced an average blank/sample ratio ≥ 0.2. **B**: The same OTU table presenting only the 21 abundant OTUs (based on ≥10% prevalence in at least 2 of the 107 samples).

**File 3: OTU97_table_alternative_filtering0.2.unique.** A rarefied OTU table (similar to the main OTU table in File 2) using the same dataset as above but curated with an alternative filtering approach. Here we used unique sequences found in the blank ‘water’ samples to remove potential contaminants instead of 97% OTUs, using the same criterion of an average blank/sample ratio ≥ 0.2 being required for removal.

**File 4: Unique seqs pre-filtration blank_included.** **A**: Unique sequence table showing the distribution of sequences identified in the MiSeq analysis of the original 107 samples before filtering. **B**: Unique sequence table showing the sequences identified in the three ‘blank’/water samples that were included in the MiSeq sequencing. To identify common sequences between ‘blank’ and ant samples we used local blast analyses. For the unique sequences that were present in both ant and ‘blank’ samples we also show the corresponding unique sequence numbers (IDs) in the ant sample table towards the right.

**File 5: Unique table post-filtration.** Unique sequence table showing the OTUs (after removing water contaminants identified in the MiSeq analysis of the original 107 samples analyzed by clustering 100% identical sequences in Mothur.

**File 6: Unique_sequence_tabular_dryad.** A table presenting all unique sequences identified in the 107 original samples in both ant and ‘blank’ ‘water’ samples. From left to right (separated with tabs): a unique identifier, description of whether the sequence was interpreted as a contaminant or as being ant-specific (based on the average blank/sample ratio ≥ 0.2), the number of times the sequence was present, the samples in which the sequence was present (based on Table S1 in Supporting Information), and the nucleotide sequence.

**File 7: OTU97 table main ms filteredOTU97_84samples. A**: OTU table showing the 2099 OTUs after rarefaction and removing ‘water’ contaminants identified in the MiSeq analysis of the 84 samples that were amplified with standard PCR and analyzed by clustering 97% identical sequences in Mothur. As before, the table was rarefied at 8900 reads. The 18 abundant OTUs according to our criterion of ≥10% prevalence in at least two samples, are highlighted with bold-face print. **B**: The same OTU table before rarefaction. To facilitate reading and interpretation we have named the 98 OTUs of highest overall abundance (Fig. S3) by using five letters that refer to their bacterial order followed by a specific number that identifies their overall prevalence rank. Six of them had previously been identified in the guts of three species of *Acromyrmex* leaf-cutting ants from the same sampling site, and retained their names from that study: *ActAcro1*, *EntAcro1*, *EntAcro2*, *EntAcro10*, *RhiAcro1* and *WolAcro1* (Sapountzis et al. 2015).

**Table S4: OTU alpha diversity and inequality indices for the MiSeq data based on 84 samples.** From left to right: Sample IDs, attine ant species, sampling environment (field, short-term lab, long-term lab), presence or absence of visible cuticular Actinobacteria, OTU richness per sample (*Sobs*), exponential Shannon diversity (*e^H’^*), exponential Theil inequality (*e^T^*) and Faith’s phylogenetic diversity (*PD*).

| **Sample ID** | **Species** | **Sampling environment** | **Actino- bacteria** | ***S_obs_*** | ***e^H’^*** | ***e^T^*** | ***PD*** |
| --- | --- | --- | --- | --- | --- | --- | --- |
| Ace_R1 | *At. cephalotes* | Field | no | 21 | 1.13 | 18.57 | 4.02 |
| Ace_3m2 | *At. cephalotes* | Lab (short term) | no | 8 | 1.01 | 7.92 | 2.32 |
| Ace_l1 | *At. cephalotes* | Lab (long term) | no | 10 | 2.05 | 4.89 | 3.64 |
| Ace_l2 | *At. cephalotes* | Lab (long term) | no | 9 | 2.27 | 3.97 | 3.65 |
| Ace_l3 | *At. cephalotes* | Lab (long term) | no | 10 | 2.05 | 4.88 | 2.44 |
| Ac_f3_rerun | *At. colombica* | Field | no | 64 | 1.33 | 48.03 | 14.57 |
| Ac_l1 | *At. colombica* | Lab (long term) | no | 5 | 1.68 | 2.98 | 0.99 |
| Ac_l2 | *At. colombica* | Lab (long term) | no | 13 | 1.03 | 12.62 | 3.52 |
| Ac_l3 | *At. colombica* | Lab (long term) | no | 6 | 2.20 | 2.72 | 1.72 |
| As_R1 | *At. sexdens* | Field | no | 97 | 33.58 | 2.89 | 1.74 |
| As_l1 | *At. sexdens* | Lab (long term) | no | 8 | 2.50 | 3.20 | 20.26 |
| As_l2 | *At. sexdens* | Lab (long term) | no | 5 | 1.01 | 4.96 | 2.60 |
| Aech_f1 | *Ac. echinatior* | Field | yes | 18 | 1.03 | 17.42 | 4.90 |
| Aech_f2 | *Ac. echinatior* | Field | yes | 7 | 2.02 | 3.46 | 2.04 |
| Aech_f3 | *Ac. echinatior* | Field | yes | 26 | 1.14 | 22.84 | 6.49 |
| Aech_3m1 | *Ac. echinatior* | Lab (short term) | yes | 6 | 1.62 | 3.71 | 1.79 |
| Aech_3m2 | *Ac. echinatior* | Lab (short term) | yes | 8 | 1.89 | 4.24 | 1.87 |
| Aech_3m3 | *Ac. echinatior* | Lab (short term) | yes | 3 | 1.93 | 1.56 | 0.96 |
| Aech_l1 | *Ac. echinatior* | Lab (long term) | yes | 6 | 2.49 | 2.41 | 1.92 |
| Aech_l2 | *Ac. echinatior* | Lab (long term) | yes | 18 | 2.92 | 6.16 | 5.40 |
| Aech_l3 | *Ac. echinatior* | Lab (long term) | yes | 15 | 3.57 | 4.20 | 4.73 |
| Aoct_f1 | *Ac. octospinosis* | Field | yes | 10 | 3.03 | 3.31 | 3.17 |
| Aoct_f2 | *Ac. octospinosis* | Field | yes | 8 | 2.04 | 3.93 | 2.31 |
| Aoct_f3 | *Ac. octospinosis* | Field | yes | 16 | 2.53 | 6.33 | 4.16 |
| Aoct_3m1 | *Ac. octospinosis* | Lab (short term) | yes | 5 | 2.63 | 1.90 | 1.75 |
| Aoct_3m2 | *Ac. octospinosis* | Lab (short term) | yes | 9 | 2.72 | 3.30 | 2.19 |
| Aoct_3m3 | *Ac. octospinosis* | Lab (short term) | yes | 18 | 2.64 | 6.83 | 4.30 |
| Aoct_l1 | *Ac. octospinosis* | Lab (long term) | yes | 22 | 3.85 | 5.71 | 6.19 |
| Aoct_l2 | *Ac. octospinosis* | Lab (long term) | yes | 10 | 2.14 | 4.68 | 4.04 |
| Aoct_l3 | *Ac. octospinosis* | Lab (long term) | yes | 13 | 3.70 | 3.51 | 2.74 |
| Av_l | *Ac. volcanus* | Lab (long term) | yes | 12 | 2.17 | 5.54 | 3.00 |
| Tcorn_f1 | *T. cornetzi* | Field | yes | 4 | 1.94 | 2.06 | 1.40 |
| Tcorn_f2 | *T. cornetzi* | Field | yes | 12 | 1.86 | 6.46 | 3.18 |
| Tcorn_f3 | *T. cornetzi* | Field | yes | 16 | 1.53 | 10.47 | 4.53 |
| Tcorn_3m1 | *T. cornetzi* | Lab (short term) | yes | 9 | 3.19 | 2.82 | 2.32 |
| Tcorn_3m2 | *T. cornetzi* | Lab (short term) | yes | 6 | 1.72 | 3.48 | 1.83 |
| Tcorn_3m3 | *T. cornetzi* | Lab (short term) | yes | 4 | 1.01 | 3.97 | 1.33 |
| Tcorn_l1 | *T. cornetzi* | Lab (long term) | yes | 11 | 1.01 | 10.84 | 2.22 |
| Tcorn_l2 | *T. cornetzi* | Lab (long term) | yes | 16 | 2.76 | 5.80 | 4.17 |
| Tra_l1 | *T. Sp.3* | Lab (long term) | yes | 15 | 2.83 | 5.30 | 4.92 |
| Tra_l2 | *T. Sp.3* | Lab (long term) | yes | 23 | 2.07 | 11.12 | 6.31 |
| Sam_f1 | *S. amabilis* | Field | no | 10 | 1.02 | 9.79 | 3.50 |
| Sam_f2 | *S. amabilis* | Field | no | 7 | 1.01 | 6.95 | 2.53 |
| Sam_3m1 | *S. amabilis* | Lab (short term) | no | 10 | 1.02 | 9.83 | 2.91 |
| Sam_3m2 | *S. amabilis* | Lab (short term) | no | 10 | 1.01 | 9.89 | 3.39 |
| Sam_l1 | *S. amabilis* | Lab (long term) | no | 14 | 1.02 | 13.75 | 5.25 |
| Sam_l3_rerun | *S. amabilis* | Lab (long term) | no | 265 | 61.31 | 4.32 | 40.14 |
| Tz_f1 | *T. zetecki* | Field | yes | 21 | 1.06 | 19.81 | 5.88 |
| Tz_f2 | *T. zetecki* | Field | yes | 56 | 2.36 | 23.70 | 11.33 |
| Tz_f3 | *T. zetecki* | Field | yes | 88 | 1.52 | 58.05 | 18.68 |
| Tz_3m1 | *T. zetecki* | Lab (short term) | yes | 7 | 1.79 | 3.91 | 2.07 |
| Tz_3m2 | *T. zetecki* | Lab (short term) | yes | 14 | 1.05 | 13.32 | 4.03 |
| Tz_3m3 | *T. zetecki* | Lab (short term) | yes | 44 | 1.47 | 29.92 | 10.31 |
| Tz_l1_rerun | *T. zetecki* | Lab (long term) | yes | 24 | 5.66 | 4.24 | 5.38 |
| Tz_l2 | *T. zetecki* | Lab (long term) | yes | 22 | 1.06 | 20.85 | 6.24 |
| Tz_l3 | *T. zetecki* | Lab (long term) | yes | 11 | 2.07 | 5.30 | 4.62 |
| Clong_f1 | *C. longiscapus* | Field | yes | 25 | 8.40 | 2.98 | 6.24 |
| Clong_f2 | *C. longiscapus* | Field | yes | 30 | 4.56 | 6.58 | 7.18 |
| Clong_3m1 | *C. longiscapus* | Lab (short term) | yes | 55 | 9.15 | 6.01 | 11.70 |
| Clong_3m2 | *C. longiscapus* | Lab (short term) | yes | 30 | 2.86 | 10.49 | 6.55 |
| Clong_l1 | *C. longiscapus* | Lab (long term) | yes | 23 | 3.33 | 6.91 | 6.81 |
| Clong_l2 | *C. longiscapus* | Lab (long term) | yes | 27 | 5.89 | 4.59 | 5.60 |
| Ccost_f1 | *C. costatus* | Field | yes | 22 | 1.86 | 11.84 | 6.32 |
| Ccost_f2 | *C. costatus* | Field | yes | 27 | 2.58 | 10.48 | 6.12 |
| Ccost_f3 | *C. costatus* | Field | yes | 192 | 3.83 | 50.12 | 32.37 |
| Ccost_3m1 | *C. costatus* | Lab (short term) | yes | 14 | 1.43 | 9.78 | 3.57 |
| Ccost_3m2 | *C. costatus* | Lab (short term) | yes | 65 | 2.56 | 25.40 | 14.37 |
| Ccost_3m3 | *C. costatus* | Lab (short term) | yes | 60 | 3.74 | 16.03 | 14.08 |
| Ccost_l1 | *C. costatus* | Lab (long term) | yes | 38 | 4.58 | 8.30 | 10.26 |
| Ccost_l3 | *C. costatus* | Lab (long term) | yes | 53 | 3.01 | 17.61 | 12.20 |
| Cri_f1 | *C. rimosus* | Field | yes | 704 | 44.84 | 15.70 | 82.26 |
| Cri_l1 | *C. rimosus* | Lab (long term) | yes | 80 | 14.57 | 5.49 | 15.09 |
| Cri_l2 | *C. rimosus* | Lab (long term) | yes | 36 | 2.76 | 13.06 | 8.54 |
| Msmi_f1 | *Myc. smithii* | Field | no | 19 | 2.26 | 8.41 | 5.70 |
| Msmi_f2 | *Myc. smithii* | Field | no | 35 | 4.65 | 7.53 | 8.23 |
| Msmi_f3 | *Myc. smithii* | Field | no | 100 | 1.47 | 67.90 | 23.56 |
| Msmi_l2 | *Myc. smithii* | Lab (long term) | no | 88 | 1.54 | 57.23 | 19.67 |
| Medn_f1 | *Myr. ednaella* | Field | no | 36 | 5.84 | 6.16 | 7.37 |
| Medn_f3 | *Myr. ednaella* | Field | no | 287 | 12.35 | 23.23 | 43.80 |
| Medn_3m3 | *Myr. ednaella* | Lab (short term) | no | 149 | 7.26 | 20.53 | 24.64 |
| Medn_l1 | *Myr. ednaella* | Lab (long term) | no | 30 | 8.86 | 3.38 | 6.49 |
| Adent_f3 | *Ap. dentigerum* | Field | yes | 115 | 4.57 | 25.15 | 21.77 |
| Adent_3m2 | *Ap. dentigerum* | Lab (short term) | yes | 21 | 1.07 | 19.63 | 4.68 |
| Adent_3m3 | *Ap. dentigerum* | Lab (short term) | yes | 57 | 3.42 | 16.64 | 12.93 |

**Table S5: 16S-qPCR samples, their species and sampling characteristics, and the ΔC_t_ values generated.** From left to right: Sample IDs, ant species, sampling environment (Env), tissue or ant body parts that DNA was extracted from, the mean Ct values of two replicates and the delta-C_t_ values calculated using the Pfaffl (2001) method. Data were used for generating Supplementary results 3 and Fig. 2.

| **Sample** | **Species** | **Env** | **Tissue** | **Mean 16S** | **Mean Ef-1a** | **ΔCt 16S** | **ΔCt Ef-1a** |
| --- | --- | --- | --- | --- | --- | --- | --- |
| Ac_l1 | *At. colombica* | lab | abdomens | 22.05 | 23.56 | 148.42 | 3.51 |
| Ac2012-1 | *At. colombica* | lab | abdomens | 22.79 | 22.52 | 94.52 | 8.68 |
| Ac_f2 | *At. colombica* | lab | abdomens | 19.58 | 22.89 | 669.28 | 6.29 |
| Ace-2012-2 | *At. cephalotes* | lab | abdomens | 17.77 | 22.47 | 2018.02 | 9.06 |
| Ace_3m3 | *At. cephalotes* | lab | abdomens | 18.99 | 23.6 | 959.07 | 3.39 |
| Ace-2012-4 | *At. cephalotes* | lab | abdomens | 21.01 | 24.7 | 279.84 | 1.30 |
| Ae150 | *Ac. echinatior* | lab | abdomens | 17.09 | 21.65 | 3054.94 | 18.52 |
| Ae331 | *Ac. echinatior* | lab | abdomens | 18.27 | 22.18 | 1487.71 | 11.67 |
| Ae484 | *Ac. echinatior* | lab | abdomens | 17.05 | 21.58 | 3130.37 | 19.68 |
| Ao482 | *Ac. octospinosus* | lab | abdomens | 17.23 | 21.25 | 2804.97 | 26.24 |
| Ao616 | *Ac. octospinosus* | lab | abdomens | 17.71 | 21.71 | 2093.22 | 17.58 |
| Ao492 | *Ac. octospinosus* | lab | abdomens | 17.4 | 21.37 | 2528.77 | 23.64 |
| 177613 | *T. cornetzi* | lab | abdomens | 19.2 | 25.03 | 843.79 | 0.97 |
| 5/2015 | *T. cornetzi* | lab | abdomens | 25.85 | 25.44 | 14.63 | 0.68 |
| Tcorn_l1 | *T. cornetzi* | lab | abdomens | 21.72 | 25.33 | 181.51 | 0.75 |
| Sam_3m2 | *S. amabilis* | lab | abdomens | 23.7 | 25.24 | 54.27 | 0.81 |
| TMI140519-13 | *S. amabilis* | lab | abdomens | 21.52 | 23.96 | 205.05 | 2.47 |
| J130504-04 | *S. amabilis* | lab | abdomens | 23.81 | 24.92 | 50.75 | 1.07 |
| Tz_l2 | *T. zeteki* | lab | abdomens | 26.83 | 25.51 | 8.05 | 0.64 |
| Tz_l1 | *T. zeteki* | lab | abdomens | 25.44 | 24.36 | 18.78 | 1.75 |
| 140515-01 | *T. zeteki* | lab | abdomens | 26.72 | 25.39 | 8.61 | 0.71 |
| 190523-07 | *Ap. dentigerum* | lab | abdomens | 23.16 | 24.83 | 75.43 | 1.16 |
| 150517-03 | *Ap. dentigerum* | lab | abdomens | 27.8 | 25.42 | 4.45 | 0.69 |
| Ace_f3 | *At. cephalotes* | field | dissected tissues | 35.21 | 32.53 | 0.05 | <0.01 |
| Ace_l1 | *At. cephalotes* | lab | dissected tissues | 19.95 | 23.98 | 535.73 | 2.44 |
| Ace_l2 | *At. cephalotes* | lab | dissected tissues | 20.56 | 23.97 | 368.20 | 2.45 |
| Ac_l1 | *At. colombica* | lab | dissected tissues | 21.26 | 22.91 | 241.01 | 6.21 |
| Ac_l2 | *At. colombica* | lab | dissected tissues | 20.24 | 23.52 | 448.90 | 3.63 |
| Ac_l3 | *At. colombica* | lab | dissected tissues | 20.48 | 22.94 | 387.79 | 6.02 |
| Aech_3m1 | *Ac. echinatior* | lab | dissected tissues | 18.13 | 22.07 | 1625.23 | 12.84 |
| Aech_f1 | *Ac. echinatior* | field | dissected tissues | 23.99 | 22.31 | 45.61 | 10.47 |
| Aech_f2 | *Ac. echinatior* | field | dissected tissues | 20.94 | 22.38 | 292.94 | 9.80 |
| Aoct_3m2 | *Ac. octospinosus* | lab | dissected tissues | 20.86 | 21.53 | 306.65 | 20.65 |
| Aoct_f1 | *Ac. octospinosus* | field | dissected tissues | 19.74 | 21.80 | 608.92 | 16.25 |
| Aoct_l2 | *Ac. octospinosus* | lab | dissected tissues | 16.58 | 22.09 | 4182.00 | 12.62 |
| Adent_3m1 | *Ap. dentigerum* | lab | dissected tissues | 29.93 | 25.57 | 1.22 | 0.61 |
| Adent_3m2 | *Ap. dentigerum* | lab | dissected tissues | 29.44 | 25.38 | 1.64 | 0.72 |
| Adent_f1 | *Ap. dentigerum* | field | dissected tissues | 31.36 | 26.28 | 0.51 | 0.33 |
| Ccost_3m1 | *C. costatus* | lab | abdomens | 26.91 | 26.29 | 7.66 | 0.32 |
| Ccost_f2 | *C. costatus* | field | abdomens | 28.63 | 26.25 | 2.69 | 0.34 |
| Ccost_l3 | *C. costatus* | lab | abdomens | 24.03 | 24.57 | 44.38 | 1.46 |
| Clong_3m1 | *C. longiscapus* | lab | abdomens | 23.00 | 24.88 | 83.42 | 1.12 |
| Clong_3m2 | *C. longiscapus* | lab | abdomens | 24.46 | 24.83 | 34.14 | 1.16 |
| Clong_f1 | *C. longiscapus* | field | abdomens | 23.01 | 25.02 | 82.91 | 0.99 |
| Medn_l2 | *M. ednaella* | lab | abdomens | 34.35 | 31.20 | 0.08 | <0.01 |
| Medn_f3 | *M. ednaella* | field | abdomens | 28.44 | 25.09 | 3.02 | 0.93 |
| rMedn_3m1 | *M. ednaella* | lab | abdomens | 31.87 | 30.91 | 0.37 | 0.01 |
| Msmi_3m1 | *M. smithii* | lab | abdomens | 32.34 | 28.81 | 0.28 | 0.04 |
| Msmi_f3 | *M. smithii* | field | abdomens | 29.10 | 26.27 | 2.02 | 0.33 |
| Msmi_l2 | *M. smithii* | lab | abdomens | 28.56 | 24.31 | 2.81 | 1.83 |
| Sam_3m1 | *S. amabilis* | lab | dissected tissues | 22.26 | 24.19 | 130.98 | 2.03 |
| Sam_f1 | *S. amabilis* | field | dissected tissues | 22.20 | 24.75 | 135.86 | 1.25 |
| Sam_l2 | *S. amabilis* | lab | dissected tissues | 33.95 | 25.99 | 0.10 | 0.42 |
| Tcorn_3m2 | *T. cornetzi* | lab | dissected tissues | 21.55 | 26.45 | 201.95 | 0.28 |
| Tcorn_f1 | *T. cornetzi* | field | dissected tissues | 20.18 | 24.73 | 465.63 | 1.27 |
| Tcorn_f2 | *T. cornetzi* | field | dissected tissues | 22.02 | 25.46 | 151.16 | 0.67 |
| Tz_3m1 | *T. zeteki* | lab | dissected tissues | 23.86 | 25.39 | 49.38 | 0.71 |
| Tz_3m3 | *T. zeteki* | lab | dissected tissues | 28.77 | 24.87 | 2.47 | 1.12 |
| Tz_f3 | *T. zeteki* | field | dissected tissues | 25.20 | 23.92 | 21.74 | 2.57 |

**Table S6: Differential representation of bacterial classes and OTUs in the abdomens of fungus-growing ants with and without cuticular Actinobacteria and on either side of the phylogenetic ‘A’ transition, using the 84 samples generated with standard PCR producing 2099 OTUs after rarefaction.** The first set of DESeq analyses (at both bacterial class-level and OTU-level) addressed whether there were significant statistical contrasts in relative OTU abundances across the ‘A’ transition identified in our study, which produced the top histogram of Fig. 3 (Table A below). The second set of DESeq analyses examined the contrast between ants with and without cuticular Actinobacteria (Table B below), which produced the bottom histogram in Fig. 3. Each of the analyses was repeated using the 48 bacterial classes (the top parts of Tables A and B) instead of the 2099 OTUs (the bottom parts of Tables A and B). Columns from left to right provide: bacterial classes or OTUs, estimated relative abundances in the entire dataset afterDESeq2 normalization (baseMean), effect sizes (fold changes), and corresponding standard errors relative to the group means compared (both along the X-axis and Y-axis of Fig. 3). The final columns give the z-values calculated by the negative binomial model in DESeq2, the p-values and the adjusted p-values after correction for multiple comparisons. Significant contrasts are presented with bold-face print. Of the 18 most abundant OTUs, which we always present in tables A and B, 13 had significant differential representations across one of the two partitions. Additional non-abundant OTUs that showed significant differential representations are presented with an asterisk towards the right. The four major classes are listed for the bacterial class-level analyses (Mollicutes, α-Proteobacteria, Actinobacteria and γ-Proteobacteria) plus any additional classes that showed significant differential representation.

| **Table A (Basal vs Evolutionarily derived attines)** | | | | | | |  |
| --- | --- | --- | --- | --- | --- | --- | --- |
| **Class** | **baseMean** | **effect size** | **SE** | **z-value** | **p-value** | **adjusted p-value** |  |
| **Actinobacteria** | **1411.54** | **4.97** | **0.47** | **10.54** | **<0.01** | **<0.01** |  |
| **Alphaproteobacteria** | **51326.07** | **-3.15** | **0.57** | **-5.48** | **<0.01** | **<0.01** |  |
| Mollicutes | 58159.89 | -1.45 | 0.63 | -2.31 | 0.02 | 0.12 |  |
| Gammaproteobacteria | 731.70 | 0.34 | 0.63 | 0.54 | 0.59 | 0.75 |  |
| **Sphingobacteria** | **218.84** | **5.96** | **0.61** | **9.74** | **<0.01** | **<0.01** | * |
| **Betaproteobacteria** | **31.38** | **1.80** | **0.34** | **5.24** | **<0.01** | **<0.01** | * |
| **Clostridia** | **13.07** | **3.16** | **0.66** | **4.79** | **<0.01** | **<0.01** | * |
| **OTU** |  |  |  |  |  |  |  |
| ***EntAcro1*** | **14680.66** | **-8.43** | **0.81** | **-10.43** | **<0.01** | **<0.01** |  |
| *WolAcro1* | 7725.23 | -1.50 | 1.00 | -1.50 | 0.13 | 0.51 |  |
| *EntAcro10* | 12625.58 | -0.48 | 1.03 | -0.47 | 0.64 | 0.89 |  |
| *RhiAcro1* | 11081.04 | -2.27 | 0.92 | -2.45 | 0.01 | 0.08 |  |
| ***ActAcro1*** | **1792.64** | **7.09** | **0.82** | **8.67** | **<0.01** | **<0.01** |  |
| ***Entom6*** | **5224.18** | **14.09** | **1.33** | **10.63** | **<0.01** | **<0.01** |  |
| *Enter7* | 29.00 | 1.88 | 0.72 | 2.62 | 0.01 | 0.06 |  |
| ***Rhizo8*** | **961.91** | **12.58** | **1.31** | **9.64** | **<0.01** | **<0.01** |  |
| ***Xanth9*** | **581.92** | **10.87** | **1.48** | **7.35** | **<0.01** | **<0.01** |  |
| ***Pseud10*** | **21.09** | **2.53** | **0.66** | **3.84** | **<0.01** | **<0.01** | ***** |
| *Rhizo11* | 1.35 | -2.95 | 1.86 | -1.59 | 0.11 | 0.45 |  |
| ***Burkh12*** | **20.41** | **2.83** | **0.60** | **4.75** | **<0.01** | **<0.01** | ***** |
| *EntAcro2* | 0.46 | -2.28 | 1.47 | -1.55 | 0.12 | 0.47 |  |
| ***Actin14*** | **29.01** | **9.25** | **1.50** | **6.15** | **<0.01** | **<0.01** |  |
| *Actin15* | 7.11 | 0.49 | 1.10 | 0.44 | 0.66 | 0.89 |  |
| ***Rhizo19*** | **56.41** | **11.24** | **1.68** | **6.70** | **<0.01** | **<0.01** |  |
| ***Actin20*** | **171.82** | **8.54** | **1.34** | **6.38** | **<0.01** | **<0.01** | ***** |
| ***Xanth21*** | **45.54** | **-5.79** | **1.34** | **-4.31** | **<0.01** | **<0.01** | ***** |
| ***Actin22*** | **20.67** | **6.36** | **1.14** | **5.56** | **<0.01** | **<0.01** | ***** |
| ***Sphin23*** | **170.77** | **12.91** | **2.01** | **6.42** | **<0.01** | **<0.01** |  |
| ***Burkh25*** | **3.96** | **3.60** | **1.21** | **2.97** | **<0.01** | **0.03** | ***** |
| *Solir27* | 0.06 | -0.17 | 2.92 | -0.06 | 0.95 | NA |  |
| ***Rhizo30*** | **89.56** | **10.71** | **1.70** | **6.29** | **<0.01** | **<0.01** |  |
| ***Actin31*** | **4.02** | **4.61** | **1.60** | **2.88** | **<0.01** | **0.03** | ***** |
| ***Flavo33*** | **10.08** | **4.04** | **1.38** | **2.93** | **<0.01** | **0.03** | ***** |
| *Rhizo35* | 1.01 | 2.66 | 1.89 | 1.41 | 0.16 | 0.56 |  |
| ***Burkh36*** | **3.40** | **2.68** | **0.96** | **2.79** | **0.01** | **0.04** | ***** |
| ***Rhizo38*** | **4.08** | **4.79** | **1.63** | **2.93** | **<0.01** | **0.03** | ***** |
| ***Rhizo40*** | **4.06** | **3.25** | **1.11** | **2.92** | **<0.01** | **0.03** | ***** |
| ***Sphin41*** | **32.71** | **25.86** | **1.88** | **13.77** | **<0.01** | **<0.01** | ***** |
| ***Rhodo286*** | **1.37** | **-4.23** | **1.50** | **-2.83** | **<0.01** | **0.03** | ***** |
|  |  |  |  |  |  |  |  |
| **Table B (With and without cuticular Actinobacteria)** | | | | | | |  |
| **Class** | **baseMean** | **effect size** | **SE** | **z-value** | **p-value** | **adjusted p-value** |  |
| **Alphaproteobacteria** | **48235.24** | **3.04** | **0.64** | **4.76** | **<0.01** | **<0.01** |  |
| Actinobacteria | 1262.06 | 1.82 | 0.63 | 2.91 | <0.01 | 0.08 |  |
| Mollicutes | 58159.89 | -1.37 | 0.68 | -2.02 | 0.04 | 0.39 |  |
| Gammaproteobacteria | 343.77 | 0.65 | 0.61 | 1.07 | 0.29 | 0.98 |  |
| **OTU** |  |  |  |  |  |  |  |
| *EntAcro1* | 14850.13 | -0.44 | 1.07 | -0.41 | 0.68 | 1.00 |  |
| ***WolAcro1*** | **7725.23** | **10.46** | **0.94** | **11.10** | **<0.01** | **<0.01** |  |
| *EntAcro10* | 12625.58 | -2.03 | 1.09 | -1.85 | 0.06 | 1.00 |  |
| *RhiAcro1* | 11081.04 | 2.30 | 1.00 | 2.29 | 0.02 | 1.00 |  |
| *ActAcro1* | 1613.95 | 2.04 | 1.05 | 1.95 | 0.05 | 1.00 |  |
| ***Entom6*** | **212.36** | **9.18** | **1.56** | **5.90** | **<0.01** | **<0.01** |  |
| *Enter7* | 78.17 | 2.01 | 0.91 | 2.20 | 0.03 | 1.00 |  |
| *Rhizo8* | 227.20 | 0.85 | 1.72 | 0.49 | 0.62 | 1.00 |  |
| *Xanth9* | 448.25 | -1.17 | 1.93 | -0.61 | 0.54 | 1.00 |  |
| ***Rhizo11*** | **1388.03** | **-14.85** | **1.78** | **-8.35** | **<0.01** | **<0.01** |  |
| *EntAcro2* | 0.46 | 0.51 | 1.61 | 0.32 | 0.75 | 1.00 |  |
| *Actin14* | 2.89 | -2.97 | 1.81 | -1.65 | 0.10 | 1.00 |  |
| *Actin15* | 7.11 | 3.85 | 1.15 | 3.33 | <0.01 | 0.30 |  |
| *Rhizo19* | 14.11 | 5.97 | 2.18 | 2.74 | 0.01 | 1.00 |  |
| ***Actin20*** | **64.18** | **6.70** | **1.55** | **4.31** | **<0.01** | **0.01** | ***** |
| *Sphin23* | 15.85 | 0.75 | 2.82 | 0.26 | 0.79 | 1.00 |  |
| ***Solir27*** | **62.90** | **-11.02** | **2.78** | **-3.96** | **<0.01** | **0.03** |  |
| *Rhizo30* | 78.40 | -4.23 | 2.10 | -2.01 | 0.04 | 1.00 |  |
| *Rhizo35* | 0.62 | 0.00 | 2.17 | 0.00 | 1.00 | 1.00 |  |
| ***Clost64*** | **6.90** | **21.16** | **2.87** | **7.37** | **<0.01** | **<0.01** | ***** |

**Table S7: Results of applying Bayesian Markov chain Monte Carlo generalized linear mixed models to examine the effects of sampling environment and the presence/absence of cuticular Actinobacteria on the four measures of alpha diversity and inequality of the abdominal bacterial communities associated with 16 attine ant species (A-D) and a similar analysis for overall bacterial abundance (E).** Models were run using the *R* package *MCMCglmm*, with the phylogenetic tree given in Fig. S4 included as a random effect. Main effects included were the presence/absence of visible cuticular Actinobacteria (A), the environment from which the ant colony was sampled (E), and the statistical interaction between these predictors (A×E). For sampling environment, comparisons were normally made between samples collected in the field and those collected from colonies kept in the laboratory for ca. 3 month (Field v Lab), and between colonies kept in the laboratory for ca. 3 months and for more than two years (Short v Long). For bacterial titer, only lab (both short-term and long-term) and field colonies were compared. For each response variables (**A**: OTU richness, **B**: PD, **C**: OTU diversity, **D**: inequality, and **E**: bacterial titers) we used five models comparing different combinations of main effects to select the best model based on the Akaike weights calculated from the AICc values. The results for each set of comparisons (**A**-**E**) are presented in two tables: the first one presenting the output coefficients for each of the five models and the second one giving the AICc values used to select the best model. Only the best model is discussed in the main text (given at the top of the second table and highlighted with bold-face print in the first table). The logLik = log likelihood and P-values reported in the main text are those for each term in the simplest model containing that term.

**A. Models for OTU richness (*log_e_S_obs_*)**

| **Model** | **Term** | **Posterior mean** | **Lower 95% CI** | **Upper 95% CI** | **Effective**  **samples** | **p** |
| --- | --- | --- | --- | --- | --- | --- |
| 1 | (Intercept) | 1.513 | 0.936 | 2.100 | 9270 | <0.001 |
|  | E (Field v Lab) | 0.239 | -0.190 | 0.677 | 9700 | 0.278 |
|  | E (short v long) | 0.033 | -0.393 | 0.451 | 9700 | 0.873 |
|  | A | -0.020 | -0.493 | 0.452 | 9362 | 0.929 |
|  | A×E (Field v Lab) | 0.034 | -0.452 | 0.546 | 9700 | 0.884 |
|  | A×E (short v long) | 0.122 | -0.354 | 0.608 | 9700 | 0.606 |
| 2 | (Intercept) | 1.462 | 0.955 | 1.940 | 9354 | <0.001 |
|  | E (Field v Lab) | 0.273 | 0.078 | 0.477 | 9700 | 0.011 |
|  | E (short v long) | 0.125 | -0.071 | 0.328 | 9700 | 0.212 |
|  | A | 0.047 | -0.240 | 0.376 | 9700 | 0.759 |
| **3** | **(Intercept)** | **1.486** | **1.037** | **1.934** | **9700** | **<0.001** |
|  | **E (Field v Lab)** | **0.270** | **0.072** | **0.467** | **9700** | **0.007** |
|  | **E (short v long)** | **0.122** | **-0.073** | **0.323** | **9700** | **0.222** |
| 4 | (Intercept) | 1.626 | 1.147 | 2.107 | 9700 | <0.001 |
|  | A | 0.026 | -0.293 | 0.352 | 9700 | 0.873 |
| 5 | (Intercept) | 1.636 | 1.214 | 2.077 | 9700 | <0.001 |

**Model selection table.** Models sorted by Akaike weight

| **Model** | **(Int)** | **Terms** | **df** | **logLik** | **AICc** | **ΔAICc** | **Akaike weight** |
| --- | --- | --- | --- | --- | --- | --- | --- |
| 3 | 1.486 | E | 5 | -29.945 | 70.7 | - | 0.507 |
| 5 | 1.636 | - | 3 | -32.848 | 72.0 | 1.34 | 0.260 |
| 2 | 1.462 | A + E | 6 | -30.026 | 73.1 | 2.48 | 0.147 |
| 4 | 1.626 | A | 4 | -32.906 | 74.3 | 3.66 | 0.081 |
| 1 | 1.513 | A + E + A×E | 8 | -31.032 | 80.0 | 9.32 | 0.005 |

**B. Models for OTU phylogenetic diversity (*log_e_* Faith’s *PD*)**

| **Model** | **Term** | **Posterior mean** | **Lower 95% CI** | **Upper 95% CI** | **Effective**  **samples** | **p** |
| --- | --- | --- | --- | --- | --- | --- |
| 1 | (Intercept) | 2.408 | 1.509 | 3.274 | 9700 | <0.0001 |
|  | E (Field v Lab) | 0.319 | -0.225 | 0.892 | 9700 | 0.2540 |
|  | E (short v long) | -0.394 | -0.946 | 0.171 | 9700 | 0.1691 |
|  | A | -0.608 | -1.336 | 0.076 | 9700 | 0.0866 |
|  | A×E (Field v Lab) | 0.182 | -0.492 | 0.908 | 9700 | 0.6126 |
|  | A×E (short v long) | 0.760 | 0.067 | 1.468 | 9700 | 0.0365 |
| 2 | (Intercept) | 2.179 | 1.346 | 3.018 | 9700 | <0.0001 |
|  | E (Field v Lab) | 0.455 | 0.099 | 0.784 | 9700 | 0.0078 |
|  | E (short v long) | 0.075 | -0.279 | 0.415 | 9700 | 0.6695 |
|  | A | -0.252 | -0.784 | 0.270 | 9700 | 0.3332 |
| **3** | **(Intercept)** | **2.042** | **1.320** | **2.811** | **9411** | **<0.0001** |
|  | **E (Field v Lab)** | **0.466** | **0.110** | **0.800** | **9700** | **0.0109** |
|  | **E (short v long)** | **0.077** | **-0.277** | **0.422** | **9700** | **0.6600** |
| 4 | (Intercept) | 2.412 | 1.570 | 3.231 | 9700 | 0.0002 |
|  | A | -0.291 | -0.837 | 0.283 | 9700 | 0.2748 |
| 5 | (Intercept) | 2.247 | 1.523 | 3.021 | 9700 | <0.0001 |

**Model selection table.** Models sorted by Akaike weight

| **Model** | **(Int)** | **Terms** | **df** | **logLik** | **AICc** | **ΔAICc** | **Akaike weight** |
| --- | --- | --- | --- | --- | --- | --- | --- |
| 3 | 2.042 | E | 5 | -115.307 | 241.2 | - | 0.451 |
| 2 | 2.179 | A + E | 6 | -115.045 | 242.9 | 1.72 | 0.190 |
| 5 | 2.247 | - | 3 | -118.483 | 243.2 | 1.99 | 0.166 |
| 1 | 2.408 | A + E + A×E | 8 | -113.218 | 243.9 | 2.7 | 0.117 |
| 4 | 2.412 | A | 4 | -118.192 | 244.8 | 3.57 | 0.076 |

**C. Models for OTU diversity (Shannon entropy *H*)**

| **Model** | **Term** | **Posterior mean** | **Lower 95% CI** | **Upper 95% CI** | **Effective**  **samples** | **p** |
| --- | --- | --- | --- | --- | --- | --- |
| 1 | (Intercept) | 0.645 | -0.455 | 1.765 | 3685 | 0.226 |
|  | E (Field v Lab) | 0.612 | -0.305 | 1.552 | 9700 | 0.188 |
|  | E (short v long) | 0.591 | -0.308 | 1.531 | 1422 | 0.207 |
|  | A | 0.387 | -0.603 | 1.340 | 4289 | 0.437 |
|  | A×E (Field v Lab) | -0.436 | -1.443 | 0.668 | 9700 | 0.410 |
|  | A×E (short v long) | -0.294 | -1.291 | 0.809 | 1293 | 0.589 |
| 2 | (Intercept) | 0.969 | 0.018 | 1.999 | 9700 | 0.052 |
|  | E (Field v Lab) | 0.259 | -0.157 | 0.650 | 9700 | 0.213 |
|  | E (short v long) | 0.351 | -0.061 | 0.770 | 9700 | 0.100 |
|  | A | 0.104 | -0.526 | 0.719 | 9700 | 0.710 |
| 3 | (Intercept) | 1.025 | 0.183 | 1.889 | 9700 | 0.025 |
|  | E (Field v Lab) | 0.256 | -0.169 | 0.669 | 9700 | 0.224 |
|  | E (short v long) | 0.350 | -0.058 | 0.751 | 9700 | 0.087 |
| 4 | (Intercept) | 1.219 | 0.245 | 2.192 | 9700 | 0.018 |
|  | A | 0.045 | -0.592 | 0.668 | 9700 | 0.875 |
| **5** | **(Intercept)** | **1.232** | **0.378** | **2.071** | **9704** | **0.009** |

**Model selection table.** Models sorted by Akaike weight

| **Model** | **(Int)** | **Terms** | **df** | **logLik** | **AICc** | **ΔAICc** | **Akaike weight** |
| --- | --- | --- | --- | --- | --- | --- | --- |
| 5 | 1.232 | - | 3 | -91.611 | 189.5 | - | 0.644 |
| 4 | 1.219 | A | 4 | -91.459 | 191.4 | 1.90 | 0.249 |
| 3 | 1.025 | E | 5 | -91.502 | 193.8 | 4.25 | 0.077 |
| 2 | 0.969 | A + E | 6 | -91.299 | 195.7 | 6.17 | 0.030 |
| 1 | 0.645 | A + E + A×E | 8 | -95.519 | 209.0 | 19.44 | 0.000 |

**D. Models for OTU inequality (Theil entropy *T*)**

| **Model** | **Term** | **Posterior mean** | **Lower 95% CI** | **Upper 95% CI** | **Effective**  **samples** | **p** |
| --- | --- | --- | --- | --- | --- | --- |
| 1 | (Intercept) | 2.776 | 1.663 | 3.888 | 8990 | <0.001 |
|  | E (Field v Lab) | -0.063 | -0.956 | 0.795 | 9700 | 0.888 |
|  | E (short v long) | -0.573 | -1.409 | 0.300 | 10810 | 0.180 |
|  | A | -0.483 | -1.403 | 0.453 | 9700 | 0.305 |
|  | A×E (Field v Lab) | 0.529 | -0.473 | 1.497 | 9700 | 0.291 |
|  | A×E (short v long) | 0.652 | -0.264 | 1.667 | 9700 | 0.179 |
| 2 | (Intercept) | 2.366 | 1.445 | 3.273 | 9700 | <0.001 |
|  | E (Field v Lab) | 0.369 | -0.056 | 0.750 | 9925 | 0.074 |
|  | E (short v long) | -0.072 | -0.463 | 0.343 | 9700 | 0.720 |
|  | A | -0.007 | -0.583 | 0.563 | 9700 | 0.979 |
| 3 | (Intercept) | 2.370 | 1.565 | 3.179 | 9291 | <0.001 |
|  | E (Field v Lab) | 0.368 | -0.030 | 0.776 | 9700 | 0.070 |
|  | E (short v long) | -0.071 | -0.462 | 0.337 | 9700 | 0.712 |
| 4 | (Intercept) | 2.510 | 1.659 | 3.353 | 9700 | <0.001 |
|  | A | -0.001 | -0.588 | 0.588 | 9313 | 0.987 |
| **5** | **(Intercept)** | **2.511** | **1.723** | **3.287** | **9700** | **<0.001** |

**Model selection table.** Models sorted by Akaike weight

| **Model** | **(Int)** | **Terms** | **df** | **logLik** | **AICc** | **ΔAICc** | **Akaike weight** |
| --- | --- | --- | --- | --- | --- | --- | --- |
| 5 | 2.511 | - | 3 | -91.602 | 189.5 | 0.00 | 0.410 |
| 3 | 2.370 | E | 5 | -89.498 | 189.8 | 0.26 | 0.360 |
| 4 | 2.510 | A | 4 | -91.740 | 192.0 | 2.48 | 0.119 |
| 2 | 2.366 | A + E | 6 | -89.624 | 192.3 | 2.84 | 0.099 |
| 1 | 2.776 | A + E + A×E | 8 | -89.338 | 196.6 | 7.09 | 0.012 |

**E. Models for bacterial titers (Fold-increase relative to *Ap. dentigerum*)**

| **Model** | **Term** | **Posterior mean** | **Lower 95% CI** | **Upper 95% CI** | **Effective**  **samples** | **p** |
| --- | --- | --- | --- | --- | --- | --- |
| 1 | (Intercept) | -1.469 | -4.725 | 2.157 | 1871.9 | 0.366 |
|  | E | 1.880 | -0.908 | 4.977 | 231.6 | 0.177 |
|  | A | 1.204 | -2.936 | 5.170 | 586.6 | 0.553 |
|  | A×E | -1.227 | -5.204 | 2.389 | 986.2 | 0.513 |
| 2 | (Intercept) | -0.840 | -4.052 | 2.312 | 9700 | 0.551 |
|  | E | 1.058 | -0.727 | 2.859 | 7811 | 0.226 |
|  | A | -0.045 | -1.972 | 2.000 | 9700 | 0.978 |
| 3 | (Intercept) | -0.883 | -3.558 | 2.042 | 8910 | 0.490 |
|  | E (Field v Lab) | 1.105 | -0.655 | 2.867 | 5352 | 0.209 |
| 4 | (Intercept) | 0.028 | -3.109 | 2.949 | 9700 | 0.982 |
|  | A | -0.040 | -2.030 | 2.130 | 10067 | 0.980 |
| 5 | (Intercept) | -0.013 | -2.580 | 2.637 | 8350 | 0.984 |

**Model selection table.** Models sorted by Akaike weight

| **Model** | **(Int)** | **Terms** | **df** | **logLik** | **AICc** | **ΔAICc** | **Akaike weight** |
| --- | --- | --- | --- | --- | --- | --- | --- |
| 5 | 2.042 | - | 3 | -61.037 | 128.8 | - | 0.629 |
| 4 | 2.179 | A | 4 | -60.753 | 130.8 | 1.99 | 0.232 |
| 3 | 2.247 | E | 4 | -61.603 | 132.5 | 3.69 | 0.099 |
| 2 | 2.408 | A + E | 5 | -61.154 | 134.4 | 5.53 | 0.040 |
| 1 | 2.412 | A + E + A×E | 6 | -63.909 | 142.8 | 13.97 | 0.001 |

**Table S8: HOMOVAs examining differences in the overall variability of abdominal bacterial communities (beta diversity) of Panamanian attine ants across the phylogenetic ‘A’ partition and the presence/absence of cuticular Actinobacteria categories, using either the 18 OTUs (A) or the 2099 abundant OTUs (B) in analyses based on three different distance methods (Bray-Curtis, weighted Unifrac, and unweighted Unifrac).** From top to bottom and for each sub-table within A and B, we provide: Pairwise comparisons of phylogenetic groups on either side of the ‘A’ transition that was identified in our study and in a recent phylogenetic study using ancestral state reconstructions (Branstetter et al., 2017), which occurs within the polyphyletic *Trachymyrmex* grade. To obtain a statistical interaction term (below/beyond ‘A’ x presence/absence of cuticular Actinobacteria), the phylogenetically basal and derived groups were further split in species with or without visible cuticular Actinobacteria. Tables list from left to right: the groups compared (X-axis and Y-axis in Fig. 4), the observed B values estimated by the Bartlett's test for homogeneity of variances, the corresponding P-values, and the variances (SS) within each of the compared groups. Significant contrasts are given in bold-face print.

| **A: 18 dominant OTUs** | | | | |
| --- | --- | --- | --- | --- |
| **BRAY-Curtis distances** | **BValue** | **P - value** | **SSwithinX** | **SSwithinY** |
| Attines with cuticular Actinobacteria *versus* attines without | 0.0333 | 0.313 | 0.3955 | 0.3717 |
| **Evolutionarily derived attines *versus* basal attines** | **1.2494** | **<0.001*** | **0.2767** | **0.3938** |
| Evolutionarily derived attines with cuticular Actinobacteria *versus* derived attines without cuticular Actinobacteria | 0.3454 | 0.137 | 0.3003 | 0.2236 |
| Evolutionarily derived attines without cuticular Actinobacteria *versus* basal attines without cuticular Actinobacteria | 0.2087 | 0.018 | 0.3003 | 0.3941 |
| **Evolutionarily derived attines with cuticular Actinobacteria *versus* basal attines with cuticular Actinobacteria** | **2.2194** | **<0.001*** | **0.2236** | **0.3963** |
| Basal attines with cuticular Actinobacteria *versus* basal attines without cuticular Actinobacteria | 0.0101 | 0.93 | 0.3941 | 0.3963 |
|  |  |  |  |  |
| **Weighted Unifrac distances** | **BValue** | **P - value** | **SSwithinX** | **SSwithinY** |
| Attines with cuticular Actinobacteria *versus* attines without | 0.0027 | 0.878 | 0.2447 | 0.2404 |
| **Evolutionarily derived attines *versus* basal attines** | **1.8054** | **<0.001*** | **0.1787** | **0.2732** |
| Evolutionarily derived attines with cuticular Actinobacteria *versus* derived attines without cuticular Actinobacteria | 0.2233 | 0.419 | 0.1824 | 0.1438 |
| Evolutionarily derived attines without cuticular Actinobacteria *versus* basal attines without cuticular Actinobacteria | 0.3725 | 0.175 | 0.1824 | 0.2625 |
| **Evolutionarily derived attines with cuticular Actinobacteria *versus* basal attines with cuticular Actinobacteria** | **2.9459** | **<0.001*** | **0.1438** | **0.2784** |
| Basal attines with cuticular Actinobacteria *versus* basal attines without cuticular Actinobacteria | 0.0148 | 0.583 | 0.2625 | 0.2784 |
|  |  |  |  |  |

| **Unweighted Unifrac distances** | **BValue** | **P - value** | **SSwithinX** | **SSwithinY** |
| --- | --- | --- | --- | --- |
| Attines with cuticular Actinobacteria *versus* attines without | 0.2636 | 0.194 | 0.1379 | 0.1159 |
| **Evolutionarily derived attines *versus* basal attines** | **0.9884** | **0.018*** | **0.0915** | **0.1252** |
| Evolutionarily derived attines with cuticular Actinobacteria *versus* derived attines without cuticular Actinobacteria | 0.1668 | 0.358 | 0.1029 | 0.0837 |
| Evolutionarily derived attines without cuticular Actinobacteria *versus* basal attines without cuticular Actinobacteria | 0.3338 | 0.065 | 0.1029 | 0.1452 |
| Evolutionarily derived attines with cuticular Actinobacteria *versus* basal attines with cuticular Actinobacteria | 0.5633 | 0.127 | 0.0837 | 0.1116 |
| Basal attines with cuticular Actinobacteria *versus* basal attines without cuticular Actinobacteria | 0.3083 | 0.208 | 0.1452 | 0.1116 |

| **B: All 2099 OTUs** | | | | |
| --- | --- | --- | --- | --- |
| **Bray-Curtis dissimilarities** | **BValue** | **P - value** | **SSwithinX** | **SSwithinY** |
| Attines with cuticular Actinobacteria *versus* attines without | 0.0351 | 0.278 | 0.3988 | 0.3742 |
| **Evolutionarily derived attines *versus* basal attines** | **1.3091** | **<0.001*** | **0.2785** | **0.3997** |
| Evolutionarily derived attines with cuticular Actinobacteria *versus* derived attines without cuticular Actinobacteria | 0.3289 | 0.142 | 0.3014 | 0.2260 |
| Evolutionarily derived attines without cuticular Actinobacteria *versus* basal attines without cuticular Actinobacteria | 0.2224 | 0.024 | 0.3014 | 0.3990 |
| **Evolutionarily derived attines with cuticular Actinobacteria *versus* basal attines with** cuticular Actinobacteria | **2.2412** | **<0.001*** | **0.2260** | **0.4016** |
| Basal attines with cuticular Actinobacteria *versus* basal attines without cuticular Actinobacteria | 0.0002 | 0.918 | 0.3990 | 0.4016 |
|  |  |  |  |  |
| **Weighted Unifrac distances** | **BValue** | **P - value** | **SSwithinX** | **SSwithinY** |
| Attines with cuticular Actinobacteria *versus* attines without | 0.3523 | 0.064 | 0.1079 | 0.0883 |
| **Evolutionarily derived attines *versus*** **basal attines** | **2.1910** | **<0.001*** | **0.0657** | **0.1050** |
| Evolutionarily derived attines with cuticular Actinobacteria *versus* derived attines without cuticular Actinobacteria | 1.2438 | 0.055 | 0.0804 | 0.0464 |
| Evolutionarily derived attines without cuticular Actinobacteria *versus* basal attines without cuticular Actinobacteria | 0.3032 | 0.205 | 0.0804 | 0.1117 |
| **Evolutionarily derived attines with cuticular Actinobacteria *versus* basal attines with cuticular Actinobacteria** | **4.1545** | **<0.001*** | **0.0464** | **0.1019** |
| Basal attines with cuticular Actinobacteria *versus* basal attines without cuticular Actinobacteria | 0.0367 | 0.409 | 0.1117 | 0.1019 |
|  |  |  |  |  |

| **Unweighted Unifrac distances** | **BValue** | **P - value** | **SSwithinX** | **SSwithinY** |
| --- | --- | --- | --- | --- |
| **Attines with cuticular Actinobacteria *versus* attines without** | **0.1700** | **0.022*** | **0.3130** | **0.2722** |
| **Evolutionarily derived attines *versus* basal attines** | **0.4797** | **<0.001*** | **0.2326** | **0.2893** |
| Evolutionarily derived attines with cuticular Actinobacteria *versus* derived attines without cuticular Actinobacteria | 0.3816 | <0.019 | 0.2819 | 0.2069 |
| Evolutionarily derived attines without cuticular Actinobacteria *versus* basal attines without cuticular Actinobacteria | 0.0403 | 0.151 | 0.2819 | 0.3176 |
| **Evolutionarily derived attines with cuticular Actinobacteria *versus* basal attines with cuticular Actinobacteria** | **0.5281** | **<0.001*** | **0.2069** | **0.2731** |
| Basal attines with cuticular Actinobacteria *versus* basal attines without cuticular Actinobacteria | 0.1000 | 0.041 | 0.3176 | 0.2731 |

**Table S9: Results of a linear Discriminant Analysis between attine species based on 2-dimensional NMDS scores of pairwise distances between samples, subdivided according to the quadrants in Figure 4.** Quadrants are: Lower Left (LL), Evolutionarily basal ants without visible cuticular Actinobacteria; Lower Right (LR), Evolutionarily basal ants with visible cuticular Actinobacteria; Upper Left (UL), Evolutionarily derived ants without visible cuticular Actinobacteria; Upper right (UR), Evolutionarily derived ants with visible cuticular Actinobacteria. Analyses were carried out separately for the 18 dominant bacterial OTUs and for all 2099 bacterial OTUs, using three different pairwise distance measures, Bray-Curtis dissimilarity and Unweighted and Weighted UniFrac distances between samples. Discriminatory ability is summarized as the percentage of colony-level samples that were assigned to the correct attine species, and the associated Entropy *R^2^* value (a measure of discriminatory power, where 1 = perfect discrimination, 0 = no discriminatory ability.)

| Distance Measure | | 18 dominant OTUs | | All 2099 OTUs | |
| --- | --- | --- | --- | --- | --- |
|  | Quadrant | % Correctly classified | Entropy *R^2^* | % Correctly classified | Entropy *R^2^* |
| Bray-Curtis | |  |  |  |  |
|  | LL | 92.9 | 0.593 | 71.4 | 0.255 |
|  | LR | 82.8 | 0.805 | 82.8 | 0.653 |
|  | UL | 66.7 | 0.405 | 58.3 | 0.244 |
|  | UR | 58.6 | 0.372 | 41.4 | 0.286 |
| Unweighted UniFrac | |  |  |  |  |
|  | LL | 92.9 | 0.639 | 50.0 | 0.132 |
|  | LR | 55.2 | 0.233 | 37.9 | 0.187 |
|  | UL | 41.7 | 0.313 | 41.7 | 0.028 |
|  | UR | 44.8 | 0.209 | 24.1 | 0.065 |
| Weighted UniFrac | |  |  |  |  |
|  | LL | 85.7 | 0.678 | 78.6 | 0.283 |
|  | LR | 58.6 | 0.321 | 62.1 | 0.349 |
|  | UL | 66.7 | 0.322 | 66.7 | 0.335 |
|  | UR | 58.6 | 0.403 | 55.2 | 0.266 |
